# Microbial metabolites in tumor epigenetic regulation

**DOI:** 10.1002/imt2.70115

**Published:** 2026-03-13

**Authors:** Wangzheqi Zhang, Haoling Zhang, Yan Liao, Shuya Jiang, Haolong Zhang, Chenwei Huang, Zhijing Song, Rui Zhao, Ping Lu, Xiaohong Kang, Eryan Kong, Yalin Zhu, Wei Wang, Xuesong Liu, Yisheng Chen, Zhiwen Luo, Zhaoyu Li, Di Wang, Na Xing, Yadong Guo, Jingjing Zhang, Xiaojing Wang, Wenyi Wang, Mowaffaq Adam, Bakiah Shaharuddin, Muhamad Yusri Musa, Doblin Sandai, Chenglong Zhu, Aimin Jiang, Peng Luo, Zhijie Zhao, Zui Zou

**Affiliations:** ^1^ Department of Anesthesiology Changhai Hospital, Naval Medical University Shanghai China; ^2^ School of Anesthesiology, Naval Medical University Shanghai China; ^3^ Department of Biomedical Sciences, Advanced Medical and Dental Institute Universiti Sains Malaysia Penang Malaysia; ^4^ Third Department of Hepatic Surgery The Third Affiliated Hospital of Naval Medical University Shanghai China; ^5^ The First Affiliated Hospital of Henan Medical University Weihui China; ^6^ Faculty of Psychology Naval Medical University Shanghai China; ^7^ Gansu University of Chinese Medicine Lanzhou China; ^8^ Department of Oncology The First Affiliated Hospital of Henan Medical University Weihui China; ^9^ The First Affiliated Hospital of Xinxiang Medical University Weihui China; ^10^ Department of Anesthesiology Eastern Theater Command Naval Hospital Zhoushan China; ^11^ Basic Medical College, Gansu University of Chinese Medicine Lanzhou China; ^12^ Fujian Medical University Fuzhou China; ^13^ Fujian Key Laboratory of Toxicant and Drug Toxicology Medical College, Ningde Normal University Ningde China; ^14^ Department of Sports Medicine Huashan Hospital, Fudan University Shanghai China; ^15^ Department of Anesthesiology, Pain and Perioperative Medicine The First Affiliated Hospital of Zhengzhou University Zhengzhou China; ^16^ Department of Urology Shanghai Tenth People's Hospital, Tongji University Shanghai China; ^17^ Fuwai Yunnan Hospital, Chinese Academy of Medical Sciences Kunming China; ^18^ Yunnan Provincial Cardiovascular Clinical Medical Center Kunming China; ^19^ Yunnan Provincial Cardiovascular Clinical Medical Research Center Kunming China; ^20^ Department of Rheumatology and Immunology Tongren Hospital, School of Medicine, Shanghai Jiao Tong University Shanghai China; ^21^ Juntendo University Tokyo Japan; ^22^ State University of New York at Old Westbury Long Island New York USA; ^23^ Department of Clinical Medicine, Advanced Medical and Dental Institute Universiti Sains Malaysia Penang Malaysia; ^24^ Department of Urology Changhai Hospital, Naval Medical University Shanghai China; ^25^ Department of Urology The First Naval Hospital of Southern Theater Command Zhanjiang China; ^26^ Department of Oncology Zhujiang Hospital, Southern Medical University Guangzhou China; ^27^ Donghai County People's Hospital, Kangda College of Nanjing Medical University Lianyungang China; ^28^ Department of Plastic and Reconstructive Surgery Shanghai Ninth People's Hospital, Shanghai Jiao Tong University School of Medicine Shanghai China

**Keywords:** bidirectional feedback loop, epigenetic modulation, microbial metabolites, precision oncology, tumor epigenetics, tumor microenvironment

## Abstract

With an increasing global cancer burden, the regulatory function of the human microbiome and its metabolites in tumor epigenetics has garnered significant interest. Microbial metabolites are not merely passive byproducts but serve as signaling molecules and epigenetic modulators, contributing to tumor progression through multiple overlapping pathways. Short‐chain fatty acids (SCFAs) such as butyrate directly inhibit histone deacetylases to reactivate tumor suppressor genes, while secondary bile acids (BAs) induce gene silencing via DNA methylation remodeling by altering the FXR/TGR5 signaling pathway. Folate and vitamin B12 serve as substrates for DNA and histone methylation through one‐carbon metabolism. A complex bidirectional feedback loop exists between microbial metabolism and tumor epigenetics: reprogramming driven by hypoxia or oncogenes alters metabolite flux, generating molecules such as lactate and succinate that not only remodel chromatin and the tumor microenvironment (TME) but also selectively promote the growth of metabolically adapted microbial species, thereby reinforcing epigenetic dysregulation. Despite growing mechanistic insights, establishing causality and correlating spatiotemporal dynamics and dose responses within the highly heterogeneous TME remain major challenges. Data integration across multi‐omics remains limited by methodological and computational constraints. Resolving these issues will be critical for understanding the microbe–metabolite–epigenetic axis and advancing personalized precision oncology.

## Highlights

- Microbial metabolites, such as butyrate and bile acids, regulate tumor progression by modifying histone deacetylases and DNA methylation.
- Microbial metabolites modulate immune cell functions, influencing tumor progression and enhancing the effectiveness of immune therapies.
- Tumor‐induced metabolic changes promote specific microbes, which reinforce epigenetic dysregulation, creating a bidirectional feedback loop.
- Microbial‐derived vitamins and amino acids regulate DNA and histone methylation, impacting tumor suppressor genes and metabolic pathways.

## Graphical abstract

The gut microbiome modulates tumor epigenetic regulation through bioactive metabolites derived from dietary substrates. Microbiota‐produced SCFAs, secondary BAs, one‐carbon metabolites, and tryptophan‐derived ligands regulate histone acetylation, DNA methylation, and chromatin remodeling via HDAC, DNMT, AhR, and metabolic cofactor‐dependent pathways. These epigenetic alterations reshape tumor metabolism, microenvironmental heterogeneity, and tumor suppressor gene activity. Conversely, tumor hypoxia, oncogenic signaling, and metabolic stress alter microbial composition and function, forming a bidirectional feedback loop that promotes tumor progression, metastasis, and therapy resistance. This microbiome–epigenetic axis reveals key mechanistic challenges and highlights opportunities for precision oncology through multi‐omics profiling and microbiome‐targeted therapeutic strategies. Abbreviations: AhR, aryl hydrocarbon receptor; BAs, bile acid(s); DNMT, DNA methyltransferase(s); HDAC, histone deacetylase(s); SCFAs, short‐chain fatty acid(s); TSG, tumor suppressor gene(s).

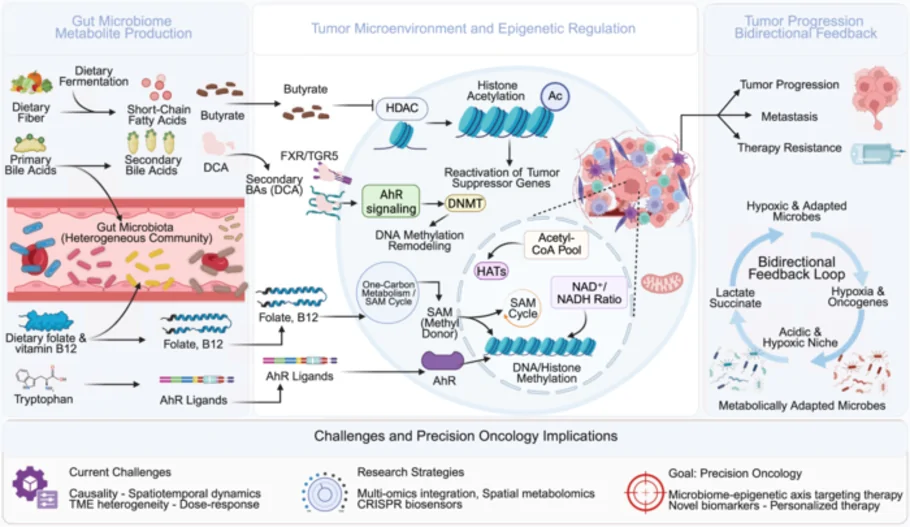

## INTRODUCTION

As global cancer incidence and mortality rise annually, the need to move beyond traditional oncology doctrine paradigms has become increasingly evident [1]. Notably, recent research has focused on the human microbiome, a community of bacteria, fungi, viruses, and their genetic material. Once viewed primarily as harmless symbionts, these microorganisms are now recognized as active regulators of host physiology and pathology. Through their metabolic activities, the microbiota produce a vast array of small‐molecule metabolites that have gained considerable attention for their role in tumor initiation and progression. Beyond supplying energy and structural precursors to tumor cells, microbiota‐derived metabolites also act as signaling molecules and epigenetic modulators. They can affect host gene expression, signal transduction, metabolic pathways, and the immune microenvironment, thereby regulating tumor cell proliferation, apoptosis, and migration [2, 3].

The direct interplay between microbial metabolites and the host's epigenetic machinery is a relatively new and highly important area of study in cancer biology. A large body of evidence suggests that microbial metabolites can directly or indirectly regulate host epigenetic enzymes, primarily through three mechanisms. First, they act as regulators of enzyme activity. For example, SCFAs generated through microbial fermentation of dietary fiber—especially butyrate—are well‐known inhibitors of class I and class II histone deacetylases (HDACs). By altering chromatin structure, these metabolites can reactivate silenced tumor suppressor genes (TSGs). Second, they function as activators of signaling pathways. Secondary BAs, such as deoxycholic acid (DCA), modulate DNA methylation patterns in intestinal epithelial cells via the farnesoid X receptor (FXR)/Takeda G protein‑coupled receptor 5 (TGR5) signaling pathway, leading to hypermethylation‐mediated TSG silencing and promotion of malignant progression [4, 5]. Third, they act as substrates or cofactors for epigenetic modifications. Metabolites derived from amino acids, such as tryptophan, regulate histone methylation through the aryl hydrocarbon receptor (AhR), while microbially synthesized vitamins such as folate and vitamin B12 provide essential cofactors for one‐carbon metabolism. This directly influences the bioavailability of S‐adenosyl‐L‐methionine (SAM), the universal methyl donor, thereby globally modulating DNA and histone methylation. In total, these metabolites regulate epigenetic processes through three key metabolic centers—the acetyl‐CoA pool [6], the SAM cycle, and the NAD^+^/NADH ratio. These hubs modulate the activity of histone acetyltransferases (HATs), DNA/histone methylation, and NAD‐dependent enzymes, respectively, forming an integrated regulatory network through which the microbiota influences host epigenetics.

Epigenetics offers crucial insight into how tumor cells undergo adaptive evolution in response to both internal and external stress. Unlike the relatively conserved genome sequence, the epigenome manifests high plasticity, allowing tumor cells to undergo frequent and reversible modifications. These include DNA methylation changes, histone post‐translational modifications (PTMs), non‐coding RNA (ncRNA) regulation, and remodeling of the three‐dimensional chromatin structure. Such adaptations help maintain proliferative signaling, evade apoptosis, and promote therapy resistance, posing great challenges for early diagnosis and precision therapy [7]. For example, hypermethylation of CpG islands (CGIs) in TSG promoters, coupled with global hypomethylation, serves as both a hallmark and a potential biomarker for early cancer diagnosis and prognosis [8, 9].

Epigenetic regulation in tumors constitutes a multi‐layered and dynamically evolving network, covering core mechanisms such as DNA modification, histone modification, ncRNA‐mediated regulation, and three‐dimensional chromatin remodeling. These processes jointly determine the transcriptional state of genes and play a key role in tumor initiation and progression. It is worth noting that microbial metabolites within the TME can interfere with these epigenetic mechanisms directly or indirectly—either by regulating the activity of epigenetic enzymes or by altering substrate accessibility—therefore profoundly reshaping the tumor epigenome, promoting malignant progression, and leading to therapy resistance. Notably, the interaction between microbial metabolites and tumor epigenetics is not a simple one‐way process but forms a complex bidirectional feedback loop. On the one hand, epigenetic reprogramming in tumor cells driven by factors such as hypoxia and oncogene activation can trigger metabolic remodeling, leading to the accumulation of metabolites such as lactate and succinate. These metabolites not only reinforce the epigenetic state of the tumor but also reshape the microenvironment niche. On the other hand, the resulting acidic and hypoxic TME exerts selective pressure on the resident microbial community, promoting the growth of acid‐tolerant and anaerobic strains. Ultimately, metabolites produced by these microorganisms further exacerbate or maintain tumor‐associated epigenetic dysregulation, thereby promoting continuous tumor evolution. Therefore, the self‐reinforcing cycle of “tumor epigenetics–microenvironmental metabolism–microbiome” constitutes a key mechanism driving cancer progression.

Although significant progress has been made, dissecting this complex interplay remains challenging. A major difficulty lies in establishing causality and achieving spatiotemporal resolution. The high heterogeneity and dynamic nature of the TME complicate efforts to prove causal links and elucidate the spatiotemporal relationships between specific microbial metabolites and epigenetic events. Recently, CRISPR‐Cas13‐based biosensors have been developed to detect specific metabolites in real time with spatiotemporal resolution within the TME, which may provide direct evidence for causality.

Another difficulty involves dose dependency and host variation. The biological effects of metabolites are generally nonlinear and dose‐dependent, with low and high concentrations often exerting distinct or even opposing effects. Moreover, these effects may be influenced by the genetic background of the host. Integrative approaches combining spatial metabolomics with single‐cell epigenomics could provide valuable insights into such dose‐ and context‐dependent phenomena.

A further challenge lies in technical integration and data interpretation. Effectively integrating multi‐source, heterogeneous omics data and extracting biologically meaningful networks remain major difficulties in bioinformatics. Developing advanced machine learning (ML) and artificial intelligence (AI) algorithms to establish predictive models of these interactions will be essential.

Elucidating the molecular mechanisms through which microbial metabolites regulate tumor epigenetics and defining their clinical significance constitute a new frontier in oncology. In this review, we systematically summarize the recent advances within the overarching framework of “microbiome‐ and metabolite‐driven epigenetic regulation in cancer.” Specifically, we first classify key microbial metabolites implicated in tumor regulation and outline their biosynthetic pathways and functional features. Next, we analyze how these metabolites remodel the epigenetic landscape through mechanisms involving DNA methylation, histone modifications, ncRNA regulation, and chromatin architecture. Subsequently, we discuss how tumor epigenetic states feedback to modulate microbial communities and their metabolic outputs. Finally, we highlight research strategies based on multi‐omics integration and advanced experimental models that may help translate insights into novel biomarkers and targeted therapies centered on the microbiome–epigenetics axis. By addressing tumor heterogeneity and therapy resistance, progress in this field is expected to open new avenues for precision oncology.

## CLASSIFICATION AND FUNCTIONAL CHARACTERISTICS OF MICROBIAL METABOLITES

Microorganisms generate a diverse array of small‐molecule metabolites through the fermentation of undigested host substrates and their intrinsic metabolic activities. These metabolites modulate host physiology via three primary pathways: supplying energy, transmitting signals, and directly participating in epigenetic modifications. Consequently, they exert profound regulatory effects on host metabolic enzyme activity, chromatin state, and the immune microenvironment. The diversity and functional heterogeneity of microbial metabolites form a complex regulatory network that interlinks metabolism, epigenetics, immunity, and microbiota. This network offers new insights into tumor initiation, progression, and therapeutic resistance while providing a theoretical foundation for developing combined interventions that target both the microbiome and epigenetic regulation. Various microbial taxa—including bacteria, actinomycetes, and fungi—produce structurally and functionally diverse metabolites through their characteristic biosynthetic pathways. These metabolites participate not only in energy metabolism and signal transduction but also serve as substrates or modulators of epigenetic modifications, thereby influencing tumorigenesis and progression. To illustrate their origins and categories more clearly, Figure 1 presents a classification and overview of representative metabolites derived from distinct microbial sources.

**Figure 1:**
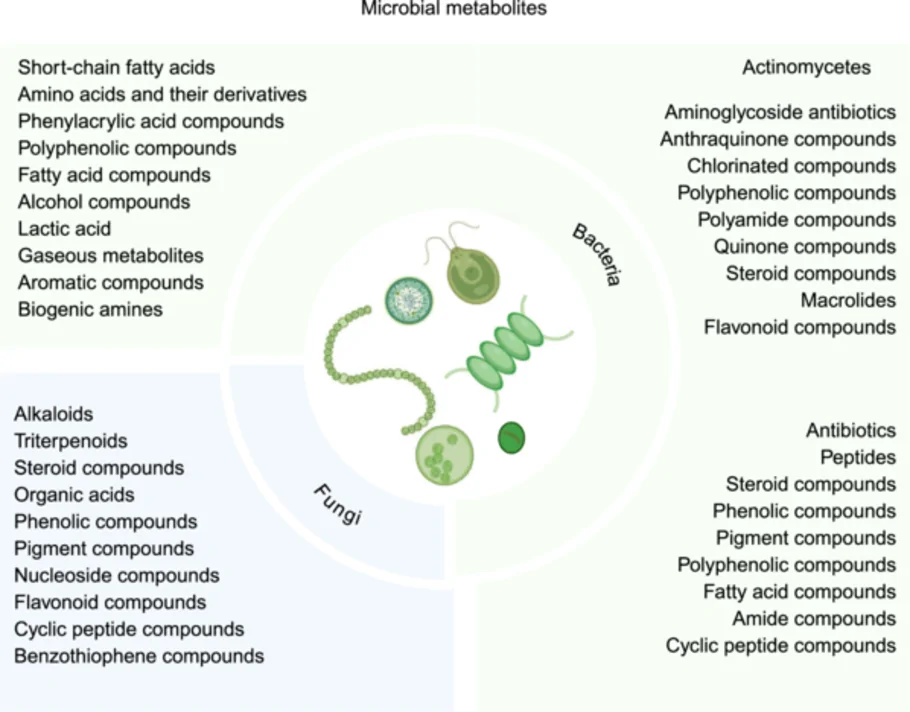
Classification and overview of metabolite types from different microbial sources (bacteria, actinomycetes, fungi). The classification covers microbial metabolites derived from bacteria, actinomycetes, and fungi. Bacterial metabolites encompass SCFAs, amino acid derivatives, lactic acid, gaseous metabolites, and aromatic compounds. Actinomycetes are known to produce a diverse array of bioactive compounds, including aminoglycoside antibiotics, macrolides, flavonoids, and steroid derivatives. Fungal metabolites include alkaloids, triterpenoids, nucleoside compounds, flavonoids, and cyclic peptides. Collectively, these metabolites constitute a structurally and functionally diverse pool of bioactive molecules that serve as substrates, cofactors, or regulators of host epigenetic modifications, thereby influencing tumor progression. Note: Actinomycetes (phylum Actinobacteria) are a distinct group of Gram‐positive bacteria. They are presented separately here to emphasize their unique contribution to the diversity and bioactivity of microbial secondary metabolites. Color coding indicates microbial origin: green, bacteria; blue, fungi. SCFA, short‐chain fatty acid.

### Chemical structure‐based classification system

Metabolites can be classified according to their chemical scaffolds—such as lipids, carbohydrates, amino acid derivatives, nucleotides, vitamin cofactors, organic acids, and alcohols. Rather than presenting a static inventory, we view this classification through the lens of how structurally diverse metabolites converge on common epigenetic regulators, including histone‐modifying enzymes, DNA methylation machinery, and ncRNA pathways. Each class of metabolites has the potential to modulate chromatin accessibility and gene transcription within the TME in a context‐specific manner. The subsections below provide representative examples and summarize their epigenetic roles and translational relevance (Table 1).

**Table 1:** Mechanism of function and clinical transformation strategy of tumor‐related intestinal microbial metabolites.

Name of metabolite	Microbial origin	Core metabolic pathways	TME regulation mechanism	Clinical translational value	Related tumor types	Treatment strategy	References
Acetate	Gut microbiota short‐chain fatty acids (SCFAs)	Fermentation of dietary fiber	Relieves energy imbalance; decreased in cancer cachexia	Biomarker of cachexia	pancreatic ductal adenocarcinoma (PDAC), lung cancer (LuCa), breast cancer (BrCa), ovarian cancer (OvCa)	SCFA supplementation and microbiota modulation	[10]
Butyrate	*Clostridium butyricum*	SCFA fermentation	Inhibits Wnt/β‐catenin pathway; activates GPR43/109 A	Anti‐proliferative, cancer prevention	colorectal cancer (CRC)	Probiotic supplementation, butyrate administration	[11]
SCFAs	*Lactobacillus pentosus* and other probiotics	Fermentation of dietary fiber	Promotes apoptosis; inhibits PI3K/Akt pathway	Apoptosis inducer against CRC	CRC	SCFA supplementation, probiotic intervention	[12]
Lactate	Product of tumor cell glycolysis	Lactate‐dependent histone lactylation	H3K18la promotes PC progression	Prognostic biomarker and therapeutic target	PDAC	Glycolysis/lactate dehydrogenase A (LDHA) inhibition	[13]
Lactate	Tumor cell hypoxic glycolysis	Lactate metabolism linked to hypoxia pathways	Induces immune exhaustion and dysfunction	Prognostic predictor and tool for immunotherapy assessment	CRC	Immune checkpoint blockade combined with optimized chemotherapy	[14]
d‐lactate	Gut microbiota–derived metabolite	Regulates PI3K/AKT signaling and macrophage polarization	Promotes tumor‐associated macrophages (TAMs) transition from M2 to M1 phenotype	Enhances efficacy of anti‐CD47 immunotherapy	hepatocellular carcinoma (HCC)	Nanodelivery combined with immunotherapy	[15]
Lactate	Gut microbiota–derived metabolite	Protein lysine lactylation (Kla) modification	Promotes immune evasion and metabolic reprogramming	Predictor of CRC prognosis	CRC	Targeting lactate metabolism combined with immunotherapy	[16]
Trimethylamine N‐oxide (TMAO)	*Clostridiales* genus	Choline metabolism producing TMAO	Activates ER stress–induced pyroptosis	Enhances immunotherapy efficacy	triple‐negative breast cancer (TNBC)	Exploiting TMAO to potentiate immunotherapy	[17]
TMAO	Gut microbial CutC‐producing bacteria	TMA → TMAO metabolic pathway	Activates TAMs and effector T cells	Enhances efficacy of immune checkpoint blockade (ICB)	PDAC, melanoma	TMAO combined with anti‐PD‐1/Tim‐3 therapy	[18]
Valerate and butyrate	Gut microbiota–derived SCFAs	mTOR signaling modulation and histone deacetylases (HDACs) inhibition	Enhances cytotoxic T lymphocytes (CTLs) and chimeric antigen receptor T‐cell activity (CAR‐T cell activity)	Improves efficacy of cellular immunotherapy	Melanoma, PDAC	SCFA treatment of CTLs or CAR‐T cells	[19]
Lactate	Gut microbiota‐derived metabolite	Warburg effect/glycolysis	Induces immunosuppression and regulatory T cells (Tregs) accumulation	Provides insight into immunotherapy resistance	non‐small cell lung cancer (NSCLC)	Anti‐APOC2K70‐lac combined with PD‐1 blockade	[20]
indole‐3‐carboxaldehyde (ICA)	*Lactobacillus gallinarum*	IDO1/Kynurenine/aryl hydrocarbon receptor (AhR) signaling axis	Inhibits Treg differentiation and enhances CD8⁺ T‐cell function	Potential application to improve anti‐PD‐1 efficacy	CRC	ICA or *L. gallinarum* combined with anti‐PD‐1 therapy	[21]
L‐KYN	gastric cancer (GCa) cell‐derived metabolites	IDO–tryptophan metabolic pathway	Induces ferroptosis of NK cells	Explains NK cell loss and prognosis in GCa	GCa	GPX4‐overexpressing NK cell therapy	[22]
Kynurenine (Kyn)	Tumor cell‐derived metabolites	Tryptophan–Kynurenine/AhR signaling	Upregulates Siglec‐15 to suppress CD8⁺ T cells	Immune evasion mechanism and therapeutic target	HNSCC	siSiglec‐15 nanoparticles combined with anti‐PD‐1 therapy	[23]
L‐KYN	Tumor IDO/TDO‐derived metabolites	Tryptophan–Kynurenine/AhR signaling	Induces Tregs and tolerogenic myeloid cells	Predictor of immunosuppression and therapeutic resistance	IDO/TDO‐overexpressing tumors	AhR inhibition combined with PD‐1 therapy	[24]
Lactate	Tumor cell‐derived metabolites	monocarboxylate transporter 4 (MCT4)‐mediated lactate transport	Acidification increases → reactive oxygen species (ROS)/NF‐κB → CXCL9/10 alterations	MCT4 as a predictive biomarker for immunotherapy	HCC	MCT4 inhibition combined with PD‐1 therapy	[25]
Lactate	Tumor cell‐derived metabolites	Lactate‐induced NUPR1 expression	Promotes M2 polarization → PD‐L1/SIRPα upregulation → T‐cell exhaustion	Predictor of immunotherapy response	HCC	NUPR1 inhibition combined with PD‐1 therapy	[26]
Succinate	Mitochondrial TCA cycle metabolites	TCA	Regulates immune cell infiltration	Prognostic indicator and predictor of immunotherapy response	CRC	Targeting SDH and related metabolic interventions	[27]
Butyrate	Gut probiotic fermentation products	Cellulose/pectin fermentation metabolism	Enhances CD8⁺ T‐cell infiltration	Improves efficacy of anti‐PD‐1 immunotherapy	CRC	Pectin dietary supplementation to modulate gut microbiota	[28]
Butyrate	Gut microbiota	SCFA metabolism and chromatin remodeling	Upregulates CXCL11 and promotes NK cell infiltration	Prognostic biomarker and immunotherapy sensitizer	HCC	Enhancement of NK cell–based immunotherapy	[29]
SCFAs	Gut microbiota	Inhibition of histone deacetylation	Enhances CD8⁺ T‐cell activation	Subtype‐dependent immunotherapy potential	CRC	SCFA intervention combined with immunotherapy	[30]
Bile acids (BAs)	Hepatic synthesis and metabolism	FXR/YAP1 BA signaling pathway	FXR upregulation suppresses YAP1‐driven carcinogenesis	Prognostic biomarker and potential therapeutic target	HCC	Targeting FXR, DHA‐based intervention	[31]
SCFAs	Gut microbiota in CRPC	Toll‐like receptor 3 (TLR3)‐mediated autophagy/NF‐κB/MAPK signaling	Recruits M2 macrophages to promote invasion	CCL20 as a prognostic biomarker	prostate cancer (PCa)	Modulation of SCFA‐producing gut microbiota	[32]

Abbreviations: AhR, aryl hydrocarbon receptor; BAs, bile acids; BrCa, breast cancer; CAR‐T, chimeric antigen receptor T cells; CRC, colorectal cancer; CTLs, cytotoxic T lymphocytes; GCa, gastric cancer; HCC, hepatocellular carcinoma; HDACs, histone deacetylases; ICA, indole‐3‐carboxaldehyde; ICB, immune checkpoint blockade; Kla, lysine lactylation; LDHA, lactate dehydrogenase A; LuCa, lung cancer; MCT4, monocarboxylate transporter 4; NSCLC, non‐small cell lung cancer; OvCa, ovarian cancer; PCa, prostate cancer; PDAC, pancreatic ductal adenocarcinoma; ROS, reactive oxygen species; SCFAs, short‐chain fatty acids; TAMs, tumor‐associated macrophages; TLR3, Toll‐like receptor 3; TMAO, trimethylamine N‐oxide; TNBC, triple‐negative breast cancer; Tregs, regulatory T cells.

#### Lipids and their derivatives

Lipid‐derived metabolites, especially SCFAs produced through microbial fermentation of dietary fiber, represent a key metabolic interface between the microbiota and host epigenetic regulation. Butyrate, acetate, and propionate function as signaling molecules that inhibit class I and III HDACs and activate G‑protein‑coupled receptors (GPR41/43) to induce interleukin‑22 (IL‑22) secretion and maintain mucosal homeostasis [33, 34, 35]. Conversely, butyrate—widely recognized as a classical HDAC inhibitor—also suppresses the Wnt/β‐catenin signaling pathway by disrupting β‐catenin complexes and restoring differentiation markers in tumors [36, 37]. Microbial dysbiosis or reduced butyrate availability disrupts this regulatory axis and promotes oncogenic activation, whereas exogenous butyrate supplementation may help reverse tumor stemness.

Besides HDAC inhibition, SCFAs regulate cytokine production, neutrophil migration, and the generation of neutrophil extracellular traps (NETs), thereby alleviating inflammation and preserving epithelial barrier integrity [38, 39]. This integrates metabolic, immune, and epigenetic regulation to maintain intestinal and oncologic homeostasis. SCFAs derived from different microbial species and metabolic pathways exhibit distinct functional profiles, as summarized in Table 2.

**Table 2:** Classification, metabolic pathway and functional characteristics of microbial metabolites.

Categories	Name of metabolite	Metabolic pathways	Key functional characteristics	Associated microorganisms/Environment	References
Organic acids	Lactic acid	Excess glucose fermentation	Causes lactic acidosis, suppresses macrophage function	Enriched *Streptococcaceae*, *Enterobacteriaceae* in TB	[40]
Fermentation metabolites	1,2‐propanediol, lactic acid, acetic acid	HMO fermentation	Antimicrobial activity, Bacterial cross‐feeding	*Bifidobacterium*	[41]
Fermentation product	Butyrate	Regulation of H3K27ac and TCR signaling	Enhancing the efficacy of anti‐PD‐1 therapy	Gut microbiota	[42]
Gut metabolites	Amino acids and related metabolites	Amino acid metabolism	Metabolites associated with host health	Firmicutes	[43]
Amino acid metabolites	Alanine	Alanine metabolic pathway	Activating AMPK and improving glucose tolerance	Mouse hepatocytes	[44]
Microbial‐associated metabolite	sodium butyrate (NaB)	Butyric acid fermentation pathway	Inhibits *H. pylori* virulence	Anaerobic bacteria, intestinal microbes	[45]
Microbial metabolites	Tryptophan metabolites	tryptophan metabolism	AhR activation	Pectin‐modulated gut microbiota	[46]
Organic acid metabolites	Citric acid	TCA cycle, glyoxylate cycle	Citric acid influences energy production and regulates cellular metabolism, while also reducing levels of cellular respiration and the capacity of bacteria to withstand oxidative stress.	Various bacterial pathogens	[47]
Tryptophan metabolites	Indole‐3‐lactic acid (ILA)	Tryptophan metabolic pathway	Regulation of YAP and Nrf2, alleviation of oxidative stress	*Lactobacillus murinus* and gut environment	[48]
Nitrogen metabolism	Ammonia	TGF‐β signaling pathway	Disrupts TGF‐β, induces inflammation	Obesity‐associated gut microbiota	[49]
SCFAs	Butyrate, Propionate	fatty acid metabolism	Prediction of low FODMAP diet efficacy	Human gut environment	[50]
Tryptophan metabolite	Indole‐3‐acetic acid (IAA)	Tryptophan metabolism	Activates hepatic AhR, improves metabolism	*Blautia coccoides*	[51]
Tryptophan metabolites	IAA	Tryptophan metabolism	Stress response, Diurnal rhythm regulation	Rhythmic tryptophan‐metabolizing bacteria	[52, 53]
Tryptophan metabolites	Indole, 2‐oxindole, IAA, Kynurenic acid	Tryptophan catabolism	Activation of AhR to maintain gut homeostasis	Gut symbiotic microbiota	[54]
Secondary metabolite	Secondary BAs	Microbial BA metabolism	Increase bile hydrophobicity, promote cholesterol absorption	Gut microbiota	[55]
BA metabolites	Deoxycholic acid (DCA), lithocholic acid (LCA)	Enterohepatic BA cycling	Promoting weaning from PN	Gut microbiota	[56]
Organic acids and their derivatives	Lactate, l‐Lac‐Phe, l‐Lac‐Val	Lactylation of amino acids	Regulation of satiety	Gut environment	[57]
Amino acids and their derivatives	Tryptophan, polyamine, glutathione (GSH)	Nicotinamide adenine dinucleotide (NAD), amino acid, one‐carbon, phospholipid metabolism	Maintaining nitrogen homeostasis	Gut microbiota	[58]
SCFAs	Butyrate	Acyl‐CoA metabolism	Butyrate influences the growth adaptability of *Bacteroides*	*Bacteroides*	[59]
Fatty acids and amino acid derivatives	Fatty acylcarnitines; tryptophan derivatives	Energy and amino acid metabolism	Regulation of energy metabolism	*Ruthenibacterium lactatiformans*	[60]
Amino acid metabolites	Arginine, tryptophan	Arginine metabolism, tryptophan metabolism	Appetite regulation via gut‐brain axis	*Akkermansia*, *Bacteroides*	[61]
Indole derivatives	Indole‐3‐propionate (IPA), ILA	Tryptophan conversion from ILA to IPA	Anti‐inflammatory, maintenance of gut barrier	*Enterocloster aldenensis*	[62]
SCFAs	Butyrate, acetate, propionate	Microbial fermentation of dietary fiber	Improvement of gut health	Gut microbiota	[63]
BAs, SCFAs	Taurine BA, acetate, butyrate, propionate	BA enterohepatic circulation, microbial fermentation	BAs influence lipid absorption, SCFAs decrease	Gut microbiota	[64]
SCFA	Sodium acetate, NaB, sodium propionate	SCFA metabolism	Antidepressant effects, epigenetic regulation	Gut bacterial communities	[65]
Amino acid derivative	Kynurenine	Tryptophan‐to‐kynurenine pathway	Positively correlated with infant weight and length	Human CMV positive breast milk	[66]
Energy metabolites, redox metabolites	Pyruvate, lactate, NAD/NADP, GSH	Glycolysis	Accumulation, suggesting enhanced aerobic glycolysis	CRC HCT‐116 cells	[67]
Fermentation metabolites	Caproate, lactate, valerate, succinate, isobutyrate, isovalerate	Proteolytic and saccharolytic fermentation	No significant link with insulin resistance	Gut microbiota fermentation	[68]

Abbreviations: DCA, deoxycholic acid; GSH, glutathione; IAA, indole‐3‐acetic acid; ILA, indole‐3‐lactic acid; IPA, indole‐3‐propionate; LCA, lithocholic acid; NaB, sodium butyrate; NAD, nicotinamide adenine dinucleotide.

#### Carbohydrates and their derivatives

Microbial metabolites, ranging from monosaccharides to polysaccharides, exhibit a hierarchical regulatory influence on host immunity and chromatin states. Lactate produced by *Lactobacillus* regulates immune signaling via GPR81 [69]. Oligosaccharides secreted by microbial enzymes modulate C‐type lectin receptors like dectin‐1, inducing signaling cascades that remodel histone acetylation and methylation [70]. Polysaccharide A exerts immunostimulatory effects by activating Toll‑like receptor 2 (TLR2), thereby promoting regulatory T‑cell (Treg) differentiation and anti‐inflammatory gene expression. Furthermore, certain sugar derivatives influence nuclear O‐GlcNAcylation by regulating the availability of the cofactor UDP‐GlcNAc, establishing a direct link between carbohydrate metabolism and transcriptional regulation [71, 72].

#### Amino acids and their derivatives

Microbial metabolism of amino acids produces metabolites with epigenetic activity. Polyamines—such as putrescine, spermidine, and spermine—interact electrostatically with DNA and histone tails, impacting nucleosome stability and chromatin compaction. Their biosynthesis consumes SAM, thereby reducing the SAM/S‐Adenosylhomocysteine (SAH) ratio and the cellular global methylation potential, which contributes to DNA hypomethylation and genomic instability in several cancers [73, 74, 75, 76, 77]. Additionally, derivatives of aromatic amino acids, notably indoles, engage the AhR within the TME, stimulating the recruitment of histone‐modifying enzymes and linking microbial metabolism to epigenetic regulation.

#### Nucleotides and their derivatives

Microbial nucleic acid metabolites, including free nucleosides/nucleotides, cyclic dinucleotides (CDNs), and NAD⁺ precursors, function as signaling molecules that link microbial activity to host epigenetic states. Adenosine suppresses the function of effector T cells through the A2A receptor and induces histone O‐adenosylation. Bacterial CDNs can stimulate the upstream signaling mediator stimulator of interferon genes (STING), thereby modulating histone H3 lysine 27 (H3K27) acetylation and methylation at tumor‐suppressor loci. Microbial NAD⁺ precursors, including nicotinamide (NAM) and nicotinamide riboside (NR), can modulate the activity of NAD⁺‐dependent enzymes such as sirtuins (SIRTs) and poly (ADP‐ribose) polymerases (PARPs). These enzymes play key roles in chromatin organization, DNA repair, and metabolic reprogramming [78, 79, 80, 81, 82]. Collectively, such metabolites bridge nucleotide metabolism with the regulatory functions of epigenetic enzymes.

#### Vitamin derivatives

Vitamin derivatives critically support one‐carbon metabolism by regulating the availability and utilization of methyl donors within the SAM cycle [83]. Folate (vitamin B9) donates methyl groups for DNA and histone methylation, thereby supporting genomic integrity. However, excess folate may promote hypermethylation of TSG promoters [84, 85]. Vitamin B12, synthesized by *Bifidobacterium* and *Lactobacillus*, facilitates the conversion of homocysteine to methionine and helps maintain H3K36me [86]. Metformin‐induced vitamin B12 deficiency can disrupt one‐carbon metabolism, leading to methylation abnormalities dependent on B12 status [87]. Furthermore, pyridoxine (vitamin B6) and niacin (vitamin B3) link amino acid metabolism with NAD^+^‐dependent SIRT activity [88, 89, 90]. Thus, vitamin‑mediated regulation of cofactor availability directly modulates epigenetic homeostasis (Table 3).

**Table 3:** The role of vitamins in cancer epigenetics, including fat‐soluble and water‐soluble vitamins.

Nutrients/category	Anti‐inflammatory effects	Pro‐inflammatory effects	References
Folic acid (water‐soluble, B vitamin)	QDPR repairs THF oxidative damage	Methotrexate induces widespread folate degradation	[91]
Vitamin A (13‐*cis*‐RA)	Promotes apoptosis and inhibits tumor progression	Resistance occurs, weakening the anticancer effect	[92]
Vitamin A	Regulates ROS and Myc, maintaining stem cell homeostasis	In its absence, stem cell depletion and instability occur	[93]
Vitamin B2 (Riboflavin)	Enhances DDP sensitivity in cancer stem cells (CSCs), promoting mitochondrial damage and apoptosis	Riboflavin accumulation in CSCs leads to resistance, promoting tumor survival	[94]
Vitamin B3 (Niacin)	Supplementing NAD^+^ improves mitochondrial metabolism and alleviates cachexia	NAD^+^ depletion leads to mitochondrial dysfunction in muscle	[95]
Vitamin B6 (Pyridoxine)	Inhibiting PNPO/PDXK reduces tumor volume and invasiveness	PNPO/PDXK activity promotes tumor progression	[96]
Vitamin B6	Adequate PLP reduces the risk of CRC	Deficiency is associated with oxidative stress and inflammation	[97]
Vitamin C (l‐ascorbic acid, water‐soluble)	Promotes DNA demethylation to enhance drug efficacy	Increases ROS‐induced cell apoptosis	[98]
Vitamin D (Fat‐soluble)	Promotes osteocyte metabolism and homeostasis	Phosphate synergistically upregulates FGF23	[99]
Vitamin D	vitamin D receptor (VDR) activation inhibits the inflammatory response	In its absence, VDR signaling is weakened	[100]
Vitamin D	Alleviates the severity of colitis and reduces IL‐6 levels	In its absence, inflammation intensifies and promotes tumor growth	[101]
Vitamin D	VDR upregulates METTL14, reducing inflammation and tumor burden	VDR deficiency leads to decreased METTL14, promoting cancer progression	[102]
Vitamin E (α‐tocopherol)	Low dose promotes cell proliferation and protection	High dose induces ROS and DNA damage	[103]
Vitamin K2 (Fat‐soluble)	Induces AMPK activation to inhibit mTORC1	Promotes glycolysis, triggering metabolic stress	[104]

Abbreviations: CSCs, cancer stem cells; VDR, vitamin D receptor.

#### Organic acids

Lactate and acetate are two highly abundant organic acids that exert both metabolic and epigenetic effects. Lactate serves as a precursor for histone lysine lactylation (Kla), particularly at histone H3 lysine 18 (H3K18la). This modification induces the expression of wound‐healing and angiogenic genes and promotes M2‐type macrophage polarization [13, 105]. Acetate is converted to acetyl‐CoA by acetyl‐CoA synthetase and serves as a substrate for HAT activity, promoting histone H3/H4 acetylation and transcriptional activation [106, 107, 108]. Through these mechanisms—Kla and acetylation—these simple metabolites dynamically remodel chromatin and integrate immune–metabolic crosstalk within the TME, as summarized in Table 4.

**Table 4:** Major mechanisms and profiles of the antitumor effects of microbial metabolites.

Metabolites	Structural formula	Derive from	Mechanisms	Cancer type	Conditions	Source of evidence	References
Tyrosol	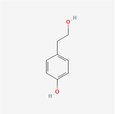	Gut microbiota	Inhibits NF‐κB/hypoxia‐inducible factor 1α (HIF‐1α)	CRC	In vivo and in vitro tumor models	Mouse models, Cell experiments	[109]
IPA	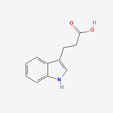	Tryptophan metabolism by gut bacteria	Enhances CD8^+^ T cell stemness via H3K27ac	Melanoma, BrCa, CRC	αPD‐1 immunotherapy	Preclinical pan‐cancer models	[110]
Isovaleric acid	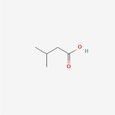	Gut microbial metabolism of leucine (BCFA)	Isovaleric acid activates FFAR3, promoting IL‐17 secretion, affecting intestinal microbiota, improving inflammation, and stimulating antitumor activity	colitis‐associated cancer (CAC)	AOM/DSS‐induced experimental colitis and CAC	In situ carcinoma mouse model	[111]
Humic acid	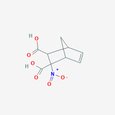	Humic substances (complex organic mixtures)	Humic acid promotes tumor progression through ROS, GSH, MMP‐2/9, and VEGF‐A	cervical cancer	Low concentration, co‐exposure with arsenic trioxide	In vitro cell assays and in vivo tumor model in NOD/SCID mice	[112]
Butyrate	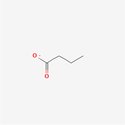	*Clostridium butyricum*	Inhibition of Wnt signaling, regulation of microbiota	CRC	High‐fat diet (HFD)‐induced intestinal tumor	Mouse model, clinical samples	[11]
Dihydroxybenzoic acid	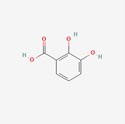	Naturally occurring benzoic acid derivatives	Benzoic acid has been shown to inhibit HDACs, thereby delaying the growth of cancer cells (CCs)	BrCa, CRC	CC culture model	Molecular docking and cell‐based assays	[113]
Butyrate	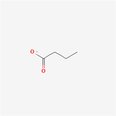	Gut microbiota fermentation of dietary fiber	Targets pyruvate kinase M2 (PKM2), metabolic reprogramming	CRC	HCT‐116 cells	DARTS quantitative proteomics, metabolomics	[114]
Propionate	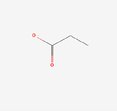	*Faecalibaculum rodentium*	Enhance Tpex‐cell expansion via histone acetylation	OvCa	Mannose supplementation	Mouse models, metabolomics analysis	[115]
Vitamin B3	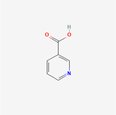	Dietary intake, microbiota‐modulated	Activates GPR109A/β‐Arrestin, inhibits NF‐κB, boosts CD8^+^ T cell antitumor immunity	HCC	Mouse model	Nutritional epidemiology, mouse models	[116]
Fulvic acid	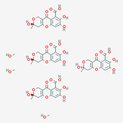	Humic substances (environment‐derived)	Inhibition of adhesion molecule expression	CRC	Co‐treatment with FA and resistin	Cell adhesion assay, NF‐κB, ICAM‐1/VCAM‐1 expression	[117]
Urolithin C	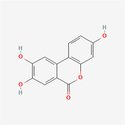	Gut‐microbial metabolism of Ellagic Acid	Inhibits AKT/mTOR signaling pathway	CRC	In vitro cells, subcutaneous tumor model	In vitro and in vivo experimental data	[118]
Isobutyric acid	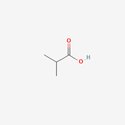	SCFA	Increased PD‐L1 expression, suppression of CD8^+^ T cells	CRC	In vitro CRC cells, mouse model	WB, RT‐qPCR, flow cytometry	[119]
Trimethylamine *N*‐oxide	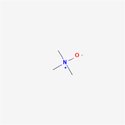	Gut microbiota–dependent choline metabolism via CutC enzyme	Activates type I IFN pathway	PDAC	Immune checkpoint blockade	Mouse models and patient data	[18]
Butyrate	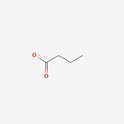	Gut microbiota	Inhibits Akt/ERK signaling	CRC	In vitro cell models	Wound healing assay, Transwell assay	[120]
3,4‐Dihydroxyphenylacetic acid	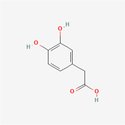	Gut microbial metabolism of cranberry proanthocyanidin	Modulate microRNA (miRNA) to reverse pro‐inflammatory responses	Gut‐related cancers	IL‐1β‐stimulated human intestinal epithelial cells	Differentiated Caco‐2BBe1 cell experiments	[121]
Butyrate	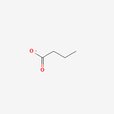	*Roseburia intestinalis*	Activate cytotoxic CD8^+^ T cells	CRC	Enhanced anti‐PD‐1 efficacy, mouse models	Patient stool analysis, CRC mouse models	[122]
l‐lactate	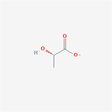	Tumor‐resident *Lactobacillus iners*	Lactate rewires metabolism, induces resistance	CESC	Chemoradiation resistance	Patient microbiome assays, in vitro assays	[123]
Rapamycin	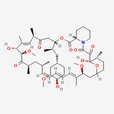	*Streptomyces* sp. (microbial secondary metabolite)	Inhibits mTOR‐FABP4 pathway	Hemangioma	In vitro HemECs	RT‐PCR, WB, BrdU, siRNA	[124, 125]
ILA	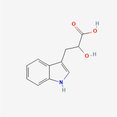	*Lactobacillus gallinarum*	Promote CRC apoptosis	CRC	In vitro & in vivo models	Murine models & cell, organoid assays	[126]
Indolepropionic acid	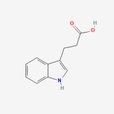	Gut microbiota (e.g., *Clostridium* spp.)	3‐Indolepropionic acid activates the AhR‐PXR pathway, induces an oxidative response, and inhibits the migration and invasion of tumor cells.	BrCa	BC cells and mouse model	Mouse model	[127]
Propionic acid	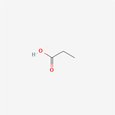	Gut microbiota (e.g., *Bacteroides*)	ROS induction, autophagy via NF‐κB and AKT/mTOR inhibition	Cervical cancer	HeLa cells	Flow cytometry	[128]
P‐coumaric acid	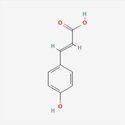	Plants, vegetables, fruits	Downregulates Grp78, induces ER stress apoptosis	CRC	DMH‐induced in vitro and in vivo models	In vitro and in vivo experiments	[129]
NaB	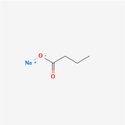	Gut microbial metabolism	Apoptosis induction, ROS inhibition	BrCa	MCF7, MDA‐MB‐231 cell lines	Proteomics, flow cytometry, ROS assay	[130]
Urolithin B	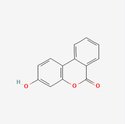	Gut microbiota metabolites	Regulates mitochondrial function and apoptosis	Lymphoma	Human lymphoma cell line HKB‐11	Proteomics, flow cytometry, ROS quantification	[131]
Butyrate	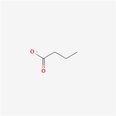	Gut microbial metabolism	Regulates calcium homeostasis, induces ROS	HCC	Sorafenib therapy, nanoparticle delivery	Clinical samples, gas chromatography–mass spectrometry (GC–MS), gene knockdown	[132]
Amphotericin B	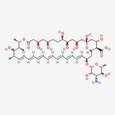	Conventional drug (microbial origin)	Induces immunogenic cell death (ICD)	Leukemic blasts	Sub‐toxic to toxic concentrations in vitro	Tumor cell lines, mouse experiments	[133, 134]
Acetic acid	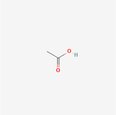	*B. thetaiotaomicron*	Modulate macrophage polarization via acetyl‐CoA carboxylase 1 (ACC1) histone acetylation	HCC	HCC recurrence, tumor growth inhibition	curcumin inhibition confirmed	[135]
4‐hydroxybenzeneacetic acid (4‐HPA)	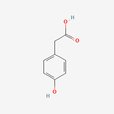	Gut microbiota metabolism	Activates JAK2/STAT3‐CXCL3 pathway	CRC	immunotherapy resistance	Mouse models, human CRC tissues	[136]

Abbreviations: 4‐HPA, 4‐hydroxybenzeneacetic acid; ACC1, acetyl‐CoA carboxylase 1; CAC, colitis‐associated cancer; CCs, cancer cells; GC–MS, gas chromatography–mass spectrometry; HFD, high‐fat diet; HIF‐1α, hypoxia‐inducible factor 1α; ICD, immunogenic cell death; miRNA, microRNA; PKM2, pyruvate kinase M2.

#### Alcohols

Microbial fermentation produces alcohols like ethanol and butanol, which impact tumor epigenetics by altering redox balance, substrate availability, and generating genotoxic intermediates. Alcohol metabolism depletes cellular NAD⁺, thereby inhibiting SIRT and PARP activity and impairing DNA repair capacity [137, 138]. Ethanol can be converted to acetyl‐CoA via acetate, providing substrates for HAT‑mediated acetylation and activating transcription programs that promote tumor growth [139, 140]. On the other hand, its metabolite acetaldehyde induces DNA adducts and mutagenesis, which are strongly associated with head‐and‐neck and esophageal cancer [141, 142]. Thus, microbial alcohols can act both as epigenetic modulators and genotoxins, driving tumor evolution through distinct mechanisms, as categorized in Table 5.

**Table 5:** The main mechanisms and conditions of microbial metabolites in pro‐tumor effects.

Metabolites	Structural formula	Derive from	Mechanisms	Cancer type	Conditions	Level of evidence	References
Putrescine	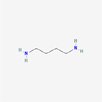	ODC synthesis of polyamines	Promotes proliferation, inhibits apoptosis	OvCa	ODC upregulation	Cell and animal experiments	[143]
Ornithine	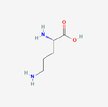	UC metabolites	Polyamine synthesis precursors	CRC	LVBU upregulation	Cell and PDX experiments	[144]
Malonate	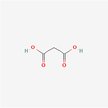	TCA cycle‐related off‐pathway metabolite	High‐dose apoptosis promotion, low‐dose autophagy promotion	Neuroblastoma (NB)	Dose‐dependent treatment	Cell experiments	[145]
Muramyl dipeptide	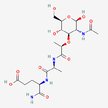	Gut microbiota‐derived bacterial cell wall component	RIP2‐mediated inflammation, nuclear autophagy‐induced DNA damage	HCC	Gut microbiota dysbiosis	Human samples, gene knockout mouse model validation	[146]
Lactate	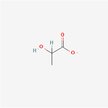	Tumor metabolism	Lactylation stabilizes DCBLD1, activates pentose phosphate pathway (PPP)	CESC	High lactate environment	In vitro and in vivo experiments	[147]
Butyrate	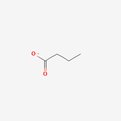	Intratumor microbiome	Epigenetic regulation of H19, inducing M2 polarization	LuCa	Early postoperative recurrence	Mouse model	[148]
Spermidine	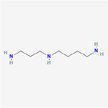	AMD1 catalyzes polyamine synthesis	eIF5A deacetylation promotes TCF4 translation	Basal‐like BrCa	AMD1 overexpression	Cell and animal experiments	[149]
Lactate	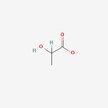	Tumor metabolism	Activates SREBP2, drives tolerogenic DCs	Melanoma	High lactate TME	In vitro and in vivo evidence	[150]
Isovalerate	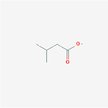	CRC‐associated gut microbiota metabolites	Activation of Tph2 promotes 5‐HT synthesis	CRC	Microbial enrichment	Cell and animal experiments	[151]
Lactate	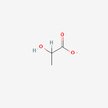	Glycolytic products, secreted by tumor cells	Activation of GPR81 inhibits antigen presentation and T cell‐infiltration	BrCa	MMTV‐PyMT and xenograft models	Mouse in vivo experiments	[69]
Citraconate	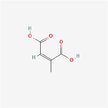	Fecal metabolite	Inhibiting ferroptosis via NRF2 pathway	CRC	Cell growth and liver metastasis	In vitro and in vivo experiments	[152]
Lactate	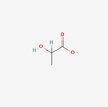	LDHA catalyzes glycolytic products	Activation of GPR81 → CX3CL1 → Treg infiltration, immune suppression	GCa	High glycolytic TME	Cell and humanized mouse experiments	[153]
TMAO	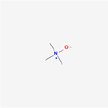	Gut microbiota metabolites of choline/carnitine	Promote CRC cell proliferation and VEGFA‐mediated angiogenesis	CRC	Choline‐fed mice + cell experiments	Cell and mouse experiments	[154]
Lactate	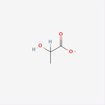	Tumor cells	GPR65 induces HMGB1 release via the cAMP/PKA/CREB signaling pathway, promoting TAM‐mediated tumor promotion	GCa	TAM sensing lactate in TME	In vitro and in vivo models	[155]
DCA	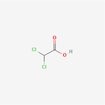	HFD‐induced BA elevation	Activation of NLRP3 inflammasome, disruption of barrier, promotion of M2 polarization	CRC	HFD + Apcmin/^+^ model	Cell and mouse experiments	[156]
Lactate	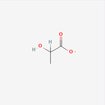	*F. nucleatum*	GLUT1‐driven glycolysis	Oral Squamous Cell Carcinoma	Tumor‐resident *F. nucleatum*	Preclinical, clinical correlation	[157]
DCA	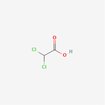	BA metabolites	Activation of TGR5 → JAK2/STAT3 → promotion of migration and invasion	NSCLC	Elevated serum DCA in patients	Cell and histological studies	[158]
Butyrate	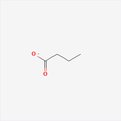	Gut Bacteria	Inhibition of M1 macrophage polarization and NLRP3 pathway	CAC	HFD	In vitro and in vivo evidence	[159]
Lactate	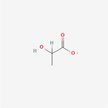	TME	H3K18 lactylation mediates immune suppression via the VCAM1‐AKT‐mTOR‐CXCL1 pathway	GC	High lactate levels in TME	In vitro and in vivo study	[160]
Lactate	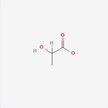	Tumor metabolism	Lactylation promotes ubiquitin‐independent cGAS degradation	LuCa	High lactate environment	In vitro and in vivo study	[161]
2‐hydroxyglutarate (2‐HG)	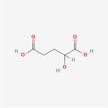	IPMN patient‐derived tumor bacteria	Induction of DNA damage; activation of carcinogenic metabolism	PDAC	Hypoxic TME	Ex vivo 3D spheroid infection	[162]
Lactate	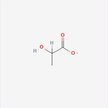	tumor cell glycolysis	Upregulates CA9, maintains pH, suppresses ROS	PC	Lactate accumulation drives CA9 expression	Cell and xenograft mouse models	[163]
Tyramine	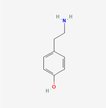	Gut bacterial metabolism	DNA damage, cell proliferation, inflammation	CRC	Post‐Roux‐en‐Y gastric bypass surgery	In vitro and in vivo experimental verification	[164]
Indoles	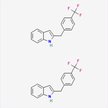	*Lactobacillus* metabolism of dietary tryptophan	Activation of AhR in TAMs, promoting immunosuppressive phenotype, inhibiting CD8⁺ T cells	PDAC	Presence of dietary tryptophan	Animal and clinical patient evidence	[165]
Lactate	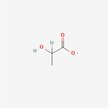	LuCa cells	Activates CAFs and recruits TAMs	LuCa	High lactate environment	In vivo and in vitro experiments	[166]
IPA	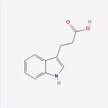	*Fusobacterium nucleatum*	Activation of AhR drives M2 polarization	CRC	In vitro co‐culture and xenograft	Cell and animal experiments	[167]
Lactate	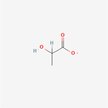	BrCa cells	Promotes c‐Myc via histone lactylation	BrCa	Enhanced glycolysis	Cell lines, mutant experiments	[168]
Trimethylamine N‐oxide	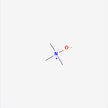	Gut microbiota	Activation of p38/HMOX1 promotes proliferation and migration	PCa	High‐choline diet	In vivo and in vitro experiments	[169]
Succinate	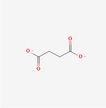	CCs	Activation of SUCNR1/PI3K/HIF‐1α signaling axis	LuCa	TME, elevated serum succinate	In vitro, in vivo, patient data	[170]
BAs	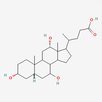	Gut microbiota	Suppress CD8^+^ T cells via oxidative	HCC	High BA environment	Human HCC samples	[171]

Abbreviations: 2‐HG, 2‐hydroxyglutarate; PPP, pentose phosphate pathway.

In summary, these chemically diverse metabolites converge on common epigenetic regulators—including HDACs, DNA methyltransferases (DNMTs), HATs, and AhR—while exerting context‐dependent effects across tumor types. The resulting integrated influence establishes a layered metabolic‐epigenetic framework that links microbial ecology with tumor evolution and therapy (see Section 2.4 for cross‑category integration).

### Microbial promotion of biosynthetic and metabolic pathways

The functions of microbial metabolites depend on their biosynthetic origins and the metabolic pathways of the producing microbes. Diverse bacterial genera, equipped with distinct enzymatic systems, convert macromolecular substrates—such as dietary carbohydrates, amino acids, and lipids—into small signaling molecules with epigenetic activity. Within the TME, these metabolites serve as key substrates and regulatory factors in epigenetic regulation.

The gut microbiota is intricately connected to the host metabolic network through complex metabolic pathways. This interaction influences the production of several important metabolites, such as SCFAs, amino acids, and BAs, and participates in host energy metabolism and signal transduction. Representative metabolic pathways of the gut microbiota and their integration with the host metabolic circuit are illustrated in Figure 2.

**Figure 2:**
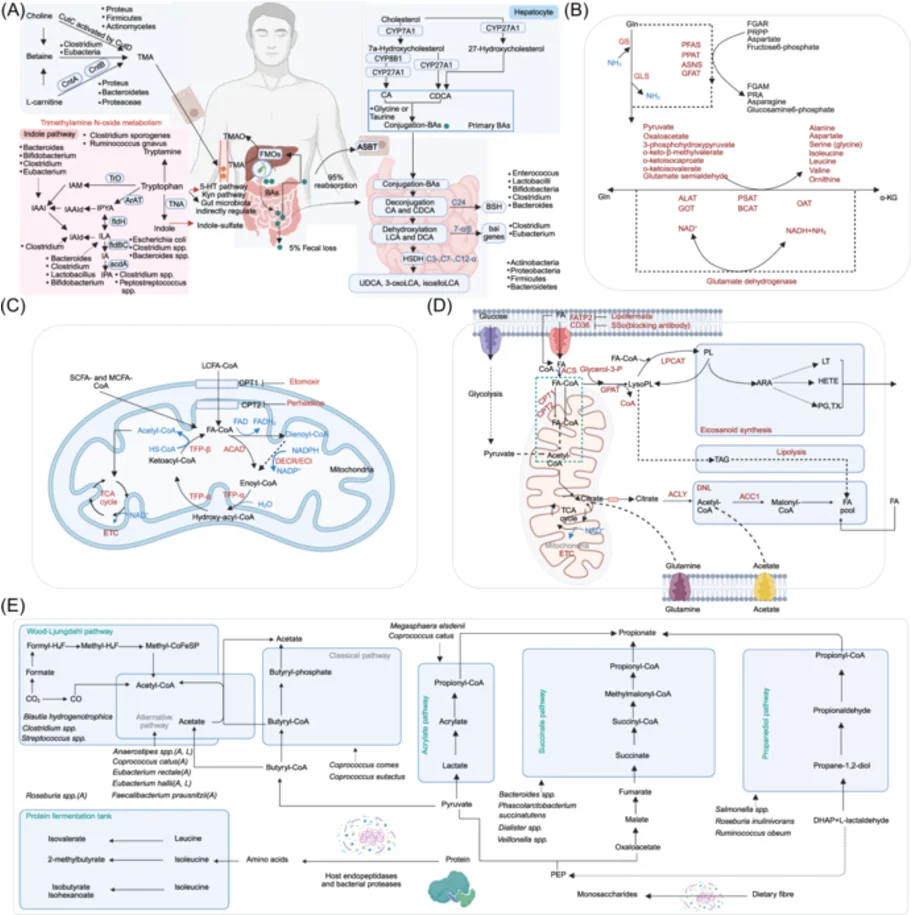
The generation pathways of intestinal microbiota metabolites and their interactions with the host metabolic network. This schematic illustrates the co‐metabolic network between the intestinal microbiota and the host, highlighting major microbially derived metabolites and their integration into host metabolic pathways. The diagram includes microbial pathways involved in TMA, BAs, and tryptophan metabolism, as well as host‐microbe interactions in amino acid metabolism, fatty acid utilization, and mitochondrial energy production. In addition, the fermentation pathways of SCFAs and protein‐derived metabolites within intestinal microorganisms are depicted. (A) Microbial and host metabolic pathways involving choline, BAs, and tryptophan‐derived metabolites along the gut‐liver axis. (B) Glutamine‐α‐KG‐centered amino acid and nitrogen metabolism linking microbial inputs with host metabolic fluxes. (C) Mitochondrial fatty acid β‐oxidation and its role in cellular energy metabolism. (D) Host lipid metabolic networks, including fatty acid uptake (inhibited by SSo, a blocking antibody targeting fatty acid transport), de novo lipogenesis, and eicosanoid synthesis. (E) Microbial fermentation pathways of dietary carbohydrates and proteins leading to SCFA production. BA, bile acid; CA, cholic acid; CDCA, chenodeoxycholic acid; DCA, deoxycholic acid; Gln, glutamine; LCA, lithocholic acid; SSO, sulfo‐*N*‐succinimidyl oleate; TMA, trimethylamine; TMAO, trimethylamine *N*‐oxide; UDCA, ursodeoxycholic acid; α‐KG, α‐ketoglutarate.

#### Carbohydrate fermentation metabolism

Carbohydrate fermentation starts with the microbial uptake and phosphorylation of dietary fibers, oligosaccharides, and monosaccharides. The carbohydrates then enter the Embden–Meyerhof–Parnas pathway, catalyzed by hexokinase and phosphofructokinase (PFK), finally resulting in the formation of pyruvate. Various bacterial genera proceed via distinct metabolic branches according to their enzymatic repertoire: (a) Homolactic fermentation: Most typical microorganisms, such as *Lactobacillus acidophilus* and *Lactococcus lactis*, further reduce pyruvate to lactate via lactate dehydrogenase (LDH). Lactate can be used as a substrate for histone lactylation, thereby modulating tumor‐cell gene expression [172]. (b) Mixed‐acid fermentation: Enterobacteriaceae (e.g., *Escherichia coli*) and Clostridiaceae (e.g., *Clostridium butyricum*) convert pyruvate into various SCFAs, such as acetate, butyrate, and ethanol [173]. Among them, acetate acts as a precursor for histone acetylation, whereas butyrate maintains chromatin accessibility through HDAC inhibition [174, 175]. (c) Propionate synthesis: Bacteria such as *Prevotella* produce propionate via the succinate or acrylate pathway. Besides functioning as a signaling molecule, propionate may affect histone propionylation levels by regulating valerate metabolism. Given that fermentation products are detectable in bodily fluids and tissues and may serve as potential tumor biomarkers, we summarize their characteristics in Table 6.

**Table 6:** Microbial metabolites as biomarkers for cancer diagnosis and prognosis evaluation.

Cancer	Sample type	Detection method	Sample size (example)	Biomarkers	Analytical methods	Diagnostic accuracy (%)	References
BrCa	Serum	GC–MS	150	Lactic acid	Analyzed by metabolomics	85%	[176, 177]
CRC	Fecal samples	16S rRNA gene sequencing (microbiota); HS‐SPME–GC×GC–TOFMS (VOC profiling)	117 samples for microbiota analysis; 28 samples for VOC analysis	Microbial biomarkers: Proteobacteria ↑, *Parabacteroides distasonis* ↑, *Lachnospiraceae* spp. ↓; Metabolic biomarkers: isobutyric acid ↑, methyl butyrate ↑, methyl propionate ↑, 2‐hexanone ↑, 2‐pentanone ↑	Microbiome analysis; VOC analysis; binomial logistic regression (association analysis)	/	[178]
HCC	Serum	¹H‐nuclear magnetic resonance (NMR) spectroscopy	64	Glucose, lactate, lipids, alanine, glutamine (Gln), 1‐methylhistidine, lysine, valine, tyrosine	Multivariate data analysis (including OPLS‐DA)	Translation close to 100%	[179]
NSCLC	Serum	UHPLC–Q‐TOF mass spectrometry	97	Multiple differential metabolites	Metabolomics data analysis (PCA, OPLS‐DA) and survival analysis	/	[180, 181]
GCa	Serum	HPLC–ESI/Q‐TOFMS	125 GCa patients + 38 healthy control	2,4‐Hexadienoic acid, dodecanoic acid 4‐methylphenyl ester, butanoic	Metabolomic analysis	/	[182]
ESCA	Serum	liquid chromatography–mass spectrometry (LC–MS)	450 patients with ESCA and 588 controls (total *n* = 1038)	15‐Metabolite predictive model	UPLC–MS/MS + bioinformatics analysis	>89%	[183, 184]
OvCa	Plasma	Multiplex immunoassay	47 cases + 179 controls (total *n* = 226)	CA125, HE4, glycoprotein	ROC curve, Mann–Whitney test, linear interpolation	CA125 area under the curve (AUC) = 0.957 (95% CI: 0.918–0.997)	[185]
PCa	Feces	16S rRNA gene sequencing	Discovery: 72 PCa + 42 controls; Validation: 24 PCa + 14 controls	*Rikenellaceae*, Alistipes, *Lachnospira* (high‐risk PCa); FMPI index (based on 18 OTUs)	LEfSe, LASSO regression, ROC curve	FMPI AUC = 0.85 (discovery); 0.81 (validation)	[186]
Bladder cancer (BLCA)	Urine	HS‐SPME–GC–MS	109	17 significantly altered volatile compounds	HS‐SPME–GC–MS analysis	80%	[187]
Kidney cancer (KCa)	Serum	NMR	Total *n* = 149 (104 training + 22 validation + 23 post‐operative assessment)	Cluster of 7 metabolites (including lactate)	NMR‐based metabolomics with self‐organizing map (SOM) analysis	/	[188]
Brain cancer (BrC)	Plasma	LC–MS	50	SCFAs (butyrate) and neutral lipid/cholesterol ratio	LC–MS analysis	/	[189]
Melanoma	Cell lines (WM115 and WM2664)	NMR	Sample size: *n* = 10 (10 replicates per cell line)	Acetylcholine, choline, inosine, guanosine; hypoxanthine, myo‐inositol, glutamate, multiple organic acids, purine/pyrimidine metabolites, amino acids	NMR + UHPLC‐HRMS/MS	/	[190]
Cervical cancer	Plasma	UPLC‐QTOF‐MS	226	AMP, aspartate, glutamate, hypoxanthine, lactate, proline, pyroglutamate	UPLC‐QTOF‐MS + multivariate statistics + MetaboAnalyst	AUC > 0.8	[191]
OvCa	Plasma, ovarian tissue, urine	GC–MS	17 OvCa patients	Lactate, malate, GSH, linoleic acid	GC–MS	AUC > 0.90	[192]
HCC	Serum	NMR	200	Propionic acid	NMR analysis	85%	[193]
PDAC	Plasma	LC–MS；GC–MS	400	Glutamate, choline, 1,5‐anhydro‐d‐glucitol, betaine, methylguanidine	LC–MS test	/	[194]
CRC	Serum	ELISA	200	TC + HDL + CEA + CA19‐9	ELISA test	/	[195]
GCa	Urine	GC–MS	Training set: 94; Validation set: 199 (total *n* = 293)	17 metabolites (10 amino acids + 4 organic metabolites)	GC–MS combined with OPLS‐DA, S‐adenosyl‐L‐methionine (SAM), Mann–Whitney *U* test, and ROC curve analysis	/	[196]
CRC	Urine	NMR	95	Lactate and 15 other metabolites (total 16)	NMR analysis combined with OPLS‐DA and multiple analyses	AUC: 0.933 (87.5%, 91.3%)	[197]
BrCa	blood	LC–MS	1947 participants (including screening, training, and validation cohorts)	Propionic acid	LC–MS analysis	AUC 0.834	[198, 199]

Abbreviations: AUC, area under the curve; BLCA, bladder cancer; BrC, brain cancer; Gln, glutamine; KCa, kidney cancer; LC–MS, liquid chromatography–mass spectrometry; NMR, nuclear magnetic resonance; SAM, S‐adenosyl‐L‐methionine.

Collectively, these microbial metabolic processes determine the concentration and ratio of typical epigenetic regulators such as SCFAs and lactate within the TME. These metabolites supply carbon sources that fuel host epigenetic regulation. In the next section, we will discuss the metabolism of protein and lipid substrates and their impact on tumor epigenetics.

#### Amino acid metabolic network

Gut microbiota metabolize amino acids to produce diverse epigenetic signaling molecules. The specific enzymatic repertoire of each bacterial genus defines its role in the metabolic network.

Transamination: *Bacteroides thetaiotaomicron*, for example, converts methionine and glycine into corresponding α‐keto acids, which enter the tricarboxylic acid (TCA) cycle to provide intermediates for further metabolite synthesis and energy production [200]. Decarboxylation: *Enterococcus faecalis* and *Lactobacillus reuteri* decarboxylate ornithine, lysine, and arginine to generate polyamines such as putrescine, spermidine, and spermine, which regulate epigenetic modifications and cell‐cycle progression [201]. Aromatic amino acid metabolism: *Clostridium sporogenes* and *Peptostreptococcus anaerobius* metabolize tryptophan into indole derivatives that activate nuclear receptors like the AhR, influencing immune regulation and chromatin remodeling [202, 203]. Branched‐chain amino acid (BCAA) metabolism: Species such as *Faecalibacterium prausnitzii* utilize BCAAs to produce branched‐chain SCFAs (e.g., isobutyrate, isovalerate), which modulate acetyl‐CoA synthesis and HDAC activity [204, 205]. Glutamate metabolism: *Bifidobacterium longum* converts glutamate to γ‑aminobutyric acid (GABA), which can affect transcription factor activity through host GABA receptors. The byproduct α‐ketoglutarate (α‐KG) affects ten‑eleven translocation (TET) enzyme activity, thereby mediating DNA demethylation [206].

Through the coordinated activities of these bacterial genera, amino acids are transformed into a spectrum of small signaling molecules. These metabolites function not only as metabolic substrates but also globally regulate tumor epigenetics by modulating histone modifications and DNA methylation, as summarized in Table 7. In the next section, we will focus on the biosynthetic pathways of lipid substrates and their regulation of tumor epigenetics.

**Table 7:** Role of key metabolic intermediates in tumor epigenetics.

Metabolites	Source	Metabolic pathways	Epigenetic roles	Tumor type in question	References
Acetyl‐CoA	ATP citrate lyase (ACLY) catalyzes the production of acetyl‐CoA	Glucose/citrate metabolism	Regulation of H3K27 acetylation	Glioblastoma (GBM)	[207]
BA	Gut microbiota‐derived bile acids	NF‐κB/SUMO3/β‐Catenin pathway	Promote histone acetylation	PDAC	[208]
α‐ketoglutarate (α‐KG)	AMPK‐dependent production	TCA cycle metabolism	Promote ten‐eleven translocation 2 (TET2), inhibit DNA methylation	CRC	[209]
Lactate	Glycolytic products	MCT1/MCT4‐mediated lactate transport	DNA methylation silencing of MCT1 promoter	Therapy‐resistant tumor cells	[210]
Lactate	CAFs	Mitochondrial metabolism, lipid metabolism	Regulation of histone acetylation	PCa	[211]
Methionine/SAM	Skeletal muscle (cancer cachexia)	One‐carbon metabolism, methionine cycle	Regulation of DNA methylation	Cachexia‐related muscle wasting	[212]
SAM	PCK1‐mediated gluconeogenesis pathway	Serine biosynthesis pathway	H3K9 trimethylation of tumor suppressor genes (TSGs)	HCC	[213]
Acetyl‐CoA	HCC Cells	Acetyl‐CoA metabolism	H3 acetylation upregulates CXCL1	HCC	[214]
α‐KG	p53‐reactivated PDAC cells	TCA cycle, oxidative metabolism	Promote 5‐hydroxymethylcytosine (5hmC), chromatin remodeling	PDAC	[215]
Succinic acid	KRAS mutant CRC cells	Glutaminolysis	DNA hyper‐methylation, decreased 5hmC	CRC	[216]
Acetyl‐CoA, fatty acids	O‐linked N‐acetylglucosamine transferase (OGT) glycosylation‐activated ACLY	PI3Kβ–OGT–ACLY metabolic axis	Enhance histone H3 acetylation, promote transcription	GBM	[217]
Butyrate	*Faecalibacterium prausnitzii*	Gut microbiota metabolites	Enhance histone acetylation, inhibit JAK/STAT	Natural killer T (NKT) cell lymphoma	[218]
Lactate	Accumulation in the TME	Lactate metabolism pathway	Histone H3K18 lactylation upregulates TNFR2	MPE	[219]
Acetyl‐CoA	ANKRD22‐SLC25A1 promotes citrate transport	Lipid metabolism reprogramming pathway	H3K27ac enhances transcription of cell cycle and cytoskeletal genes	NPC	[220]
α‐KG	Glutaminolysis	Glutaminolysis → α‐KG production pathway	KDM4C demethylation reduces H3K9me3 to regulate cFLIP	PDAC	[221]
D‐2‐HG	Produced by IDH1/2 mutations	Abnormal isocitrate → α‐KG metabolism	Inhibit TET enzyme activity, affect DNA methylation	Myeloid tumors	[222]
2‐HG	Generated by IDH1/2 mutations	Abnormal metabolism of α‐KG → 2‐HG	Induce DNA/histone hyper‐methylation, loss of acetylation	Glioma	[223]
Acetyl‐CoA	PDP1 activates PDH to generate acetyl‐CoA	PDP1/PDH/HIF‐1 metabolic axis	Maintain H3 acetylation, promote HIF target gene transcription	Various cancers (hypoxia‐related)	[224]
Acetyl‐CoA	BCAT2 regulates branched‐chain amino acids (BCAA) metabolism to generate acetyl‐CoA	BCAA metabolism → lipid synthesis pathway	P300‐mediated H3 acetylation promotes fatty acid synthase (FASN)/ACLY transcription	Melanoma	[225]
Butyrate	Dietary fiber fermentation products	Increased TCA cycle, decreased methionine metabolism	HDAC inhibition, CpG demethylation, regulate DNA methyltransferases (DNMTs)/TET	CRC	[226]
SAM	Methionine cycle production	One‐carbon metabolism/methyl donor pathway	Regulate H3K4me3 and H3K27me3 methylation	PCa	[227]
Lactic acid	Glycolytic products	Glycolysis pathway	DNA methylation changes	LuCa	[228]
Acetyl‐CoA	Fatty acid β‐oxidation	FAO/lipid metabolism	Decrease in histone acetylation	HCC	[229]
α‐KG	Cell permeability supplementation	Mitochondrial metabolism, lipid synthesis	Alter DNA methylation profile	TME (Treg‐related)	[230]
Fumarate	FH deficiency accumulation	NAD⁺ synthesis, DNA repair	Inhibit TET, leading to DNA hyper‐methylation	HLRCC	[231]
Acetyl‐CoA	Pyruvate oxidation	Mitochondrial metabolism/glycolysis	Decrease in histone acetylation	NB	[232]
NaB	Gut microbiota fermentation	Mitochondrial autophagy/apoptosis	Increased histone acetylation via HDAC inhibition	CRC	[233]
Lactate	Hypoxic metabolite	Glycolysis/LDHA pathway	H2B K58 lactylation	HCC	[234]

Abbreviations: 5hmC, 5‐hydroxymethylcytosine; ACLY, ATP citrate lyase; BCAA, branched‐chain amino acids; DNMT, DNA methyltransferase; FASN, fatty acid synthase; GBM, glioblastoma; NB, neuroblastoma; NKT, natural killer T; OGT, O‐linked *N*‐acetylglucosamine transferase; TET2, ten‐eleven translocation 2; TSGs, tumor suppressor genes; α‐KG, α‐ketoglutarate.

#### Lipid metabolic pathways

Microorganisms can convert dietary and host lipids into lipid mediators with epigenetic regulatory activities.

Triglyceride and phospholipid hydrolysis: *Bacteroides fragilis* expresses triglyceride lipase and phospholipase, hydrolyzing lipids into free fatty acids and glycerol [235]. Fatty acid derivative production: *Clostridium scindens* and *Eubacterium hallii* utilize hydrogenases and cytochrome P450 systems to produce conjugated linoleic acid and hydroxy fatty acids, which activate the peroxisome proliferator‑activated receptor (PPAR) pathway and modulate chromatin acetylation/deacetylation. Acetyl‐CoA and valeryl‐CoA synthesis: *Lactobacillus plantarum* and *Megasphaera elsdenii* employ lactobacillic acid β‐oxidation enzymes to generate acetyl‑CoA and valeryl‑CoA, serving as substrates for histone acetylation and valerylation, respectively [236, 237]. Cholesterol conversion: *Klebsiella pneumoniae* and *Prevotella intermedia* metabolize cholesterol into microbial sterols and steroid acids that activate the liver X receptor (LXR) and FXR nuclear receptor pathways, thereby regulating DNMT activity and gene‐promoter methylation [238].

These lipid metabolic pathways exhibit complex cross‐talk that coordinately regulates host immunity, metabolism, and epigenetics (for an integrated model of microbial–epigenetic interactions, Table 8). Microbial lipid metabolites are structurally diverse and functionally specific, acting as donors for acetyl, valeroyl, and acyl groups, as well as as ligands for nuclear receptors. They finely regulate tumor‐cell fate and therapy resistance.

**Table 8:** Epigenetics of tumor‐interactions between microorganisms and their epigenetic mechanisms regulating the microbial flora and TME.

Mechanism	Key molecules/enzymes	Interaction mechanism	Gut flora regulation effect	TME effect	Molecular pathways	Involves tumor type	References
Epigenetic modification	*DNMT1*, enhancer of zeste homolog 2 (EZH2), H3K27me3, AP‐2α (TFAP2A), RUNX3	Epigenetic modifications directly regulate immune checkpoint molecules and T‐ cell function	Promotes beneficial microbiota while inhibiting pathogenic taxa	Regulates T‐cell infiltration, function, and exhaustion	Epigenetic modifications upregulate PD‐L1 expression, promoting immune evasion	Hodgkin lymphoma (classical HL, cHL) and solid tumors were studied in mouse models: CT26, B16F10, GL261, and Glioma.	[239, 240]
DNA methylation	4‐Hydroxybenzoic acid (4‐HB), lactic acid, glutamic acid; Genes (PI16, FCRLA, LSP1, MT1M, RELN, PLCB1, IGSF9)	Microbial metabolites regulate gene expression through DNA methylation	*Fusobacterium* ↑ → 4‐HB ↓; *Prevotella* 2 ↓; Halomonadaceae ↑; *Paeniclostridium* ↓ → Lactic acid ↑	Downregulation of immune‐related gene expression, promotes tumorigenesis	Butyrate precursor metabolites regulate epigenetic regulation and gene expression	CRC	[241]
Histone acetylation	Histone acetyltransferases (HATs) and HDACs	Histone acetylation affects immune infiltration and regulates immune system activity	Modulates microbial community stability	The high HA score group is characterized as an immunosuppressive subtype, with high infiltration of immunosuppressive cells [e.g., Tregs and myeloid‐derived suppressor cells (MDSCs)]	Histone acetylation patterns are associated with malignant cancer pathways (e.g., chromatin remodeling, DNA repair)	HCC	[242, 243]
Microbiome‐immune system interactions	*Bifidobacterium longum*, *Enterococcus faecium*, *Collinsella* spp	Specific commensal consortia enhance antitumor T‐cell responses and the efficacy of immune checkpoint inhibitors (ICIs)	Gut microbiota in responders to immunotherapy augment antitumor immunity	Increased intratumoral infiltration of antigen‐specific CD8⁺ T cells sensitizes tumors to PD‐L1 blockade	Enhancement of systemic and intratumoral T‐cell immunity	Melanoma	[244]
Protein phosphorylation	CDK4/6, RB, NF‐κB p65, PD‐L1	Phosphorylation affects immune evasion and regulates cell proliferation	RB–NF‐κB axis regulates tumor immune evasion	Promotes tumor immune evasion and tumor progression	Phosphorylation regulates NF‐κB activity and PD‐L1 transcription, affecting tumor metabolism and immune evasion	CRC, LuCa, GCa, PCa	[245, 246]
SUMOylation	SRGs; TAK‐981 (E1 inhibitor)	SUMOScore associated with immune checkpoint expression and immune evasion; TAK‐981 activates antitumor immunity via interferon signaling	SUMOScore associated with TME and immune function; TAK‐981 transiently reduces B cells but activates T cells and NK cells	SUMOScore associated with TME, immune function, checkpoint expression, and immune evasion; TAK‐981 modulates TME by inhibiting SUMOylation	SUMOylation is closely linked to immune processes, with the low‐risk group being enriched in immune pathways; TAK‐981 induces interferon signaling, causes G2/M arrest, and activates antitumor immunity	CESC; PDAC	[247, 248]
Microbial modulation	*Lachnospiraceae*, *Bifidobacteriaceae*	Gut microbiota influences antitumor immunity by regulating DC activation and IL‐12 production	Antibiotic treatment or specific microbiota loss disrupt stability and reduce therapeutic response	Regulation of CD4⁺ T‐cell and DC recruitment/activation in the TME	Microbiota–DC–IL‐12–immune cell activation pathway	HER2‐positive BrCa	[249]
ncRNA Regulation	NEAT1, EZH2, PBX1; MALAT1, miR‐92a‐3p, ADAM10； DCA, PMCA	Exosomal long non‐coding RNAs (lncRNAs), specifically NEAT1 and MALAT1, inhibit NK cell activity through the regulation of target genes or proteases; microbial metabolite DCA compromises CD8⁺ T‐cell function by disrupting calcium signaling, thereby facilitating immune evasion	Gut bacteria with DCA biosynthesis genes produce DCA, negatively regulating CD8⁺ T‐cell effector function	Alters NK cell cytotoxicity, influencing immune evasion and tumor progression	NEAT1/EZH2/PBX1 axis; MALAT1/miR‐92a‐3p/ADAM10 axis; DCA/PMCA/NFAT calcium signaling pathway	Multiple myeloma (MM) NB, CRC	[5, 250, 251]
Microbial metabolites	Microbial metabolites (e.g., indole); host signaling molecules (AhR, IFN‐γ, IL‐2)	Microbial metabolites affect immune evasion and regulate immune cell function	Promotes beneficial microbiota growth	Remodeling the TME to suppress tumor progression and overcome immune evasion	AhR signaling; glycerophospholipid and sphingolipid metabolism; IFN‐γ and IL‐2 signaling	CRC; PDAC	[165, 252]
Microbiome–T cell immune regulation	IFN‐γ, IL‐17A, IL‐10	Microbiota depletion enhances antitumor T‐cell responses and suppresses immunosuppressive T cells	Microbiota depletion suppresses tumor growth by enhancing T‐cell function	Alters TME via metabolism, affecting tumor development	Microbiome modulates CD8⁺ T‐cell activity to influence immunotherapy response	CRC, GCa, BrCa, Melanoma, NSCLC, KCa, HCC	[253, 254]
Microbe‐immune evasion regulation	CCR1, PD‐L1, lipopolysaccharide (LPS), MAPK (phosphorylated form)	Microbial metabolites influence immune response via metabolic pathways	Microbial metabolites enhance immune evasion	Persistent microbial metabolite stimulation induces tumorigenesis and remodels immune microenvironment	Microbes regulate tumor proliferation by affecting immune system response	ESCA	[255]
Microbe‐immune system interaction	Immune checkpoint molecules and signaling mediators	Immune cell metabolic changes modulate TME	Microbes influence immune evasion mechanisms	Microbes alter immune evasion, regulating tumor proliferation and growth	Microbiota interact with immune system at mucosal interfaces, presenting antigens to lymphocytes and modulating the TME	CRC	[256, 257]
Microbe‐TME regulation	Gastric microbiota regulate Tregs and pDCs through low‐abundance genera (e.g., *Stenotrophomonas*, *Selenomonas*), influencing immune evasion	Immune evasion alters tumor growth via epigenetic mechanisms	Microbe‐tumor cell interaction affects immune response	Microbial metabolites influence immune evasion via Tregs and pDCs, regulating tumor cell growth	Microbial metabolites modulate macrophage polarization and NK‐DC crosstalk, affecting immune evasion	GCa; BP; MC38; TUBO	[258, 259]
Bacterial epigenetic modification	Host DNMT	Host‐bacteria epigenetic interaction	Enrichment of *Fusobacterium nucleatum* (*F. nucleatum*) and *Hungatella hathewayi* (*H. hathewayi*) promotes hypermethylation of TSG promoters	Bacterial epigenetic modifications alter tumor‐associated inflammation and tumor cell expression	Upregulation of DNMT expression and activity induces hypermethylation of TSG promoters (e.g., APC, CDX2, EYA4)	CRC	[260]
Microbe–host metabolic interactions	Tryptophan metabolites	Microbial metabolites regulate host immunometabolism via tryptophan and glycerophospholipid pathways	Modulates abundance of beneficial bacteria (e.g., *Lactobacillus*, *Bifidobacterium*, *Faecalibacterium*)	Affects PD‐L1 expression and tumor cell growth	Tryptophan and glycerophospholipid metabolism jointly regulate microbe–host interactions, influencing immune tolerance and tumor resistance	BrCa	[261, 262]
Viral epigenetic regulation	Epstein‐Barr virus (EBV) latent membrane protein 1 (LMP1)	Viral proteins regulate host DNA methylation	Viruses promote transmission and HCC progression via epigenetic reprogramming of metabolic pathways (e.g., glycolysis/gluconeogenesis)	Viral epigenetic modifications promote tumor immune evasion and growth	Viral epigenetic regulation reshapes host chromatin states; independently, HMGB2 regulates Tpex cell differentiation and function through epigenetic and transcriptional programming	HCC	[263, 264]
Microbial DNA hydroxymethylation	TET enzymes	Microbial metabolites affect TET enzyme activity	Promotes ILC1 differentiation and suppresses TET1 expression	Regulates ILC1 differentiation and intestinal inflammatory homeostasis	TET1‐mediated hydroxymethylation of the Tgfbr1 promoter regulates the TGF‐β signaling pathway	GCa	[265, 266]
UDCA degrades TGF‐β1 via autophagy to suppress Treg cells	UDCA, TGF‐β1, LC3B, autophagy inhibitor Baf‐A1	UDCA partially substitutes cholesterol to form immunoregulatory ULNPs encapsulating cRNAIL‐2F	ULNPs‐cRNAIL‐2F@G enable local sustained release, avoiding systemic exposure	Reduces TGF‐β1, increases CD8⁺ T/Treg ratio, reverses immunosuppressive microenvironment	TGF‐β1 autophagic degradation pathway; IL‐2/CD8⁺ T‐cell activation pathway	Melanoma, GBM	[267, 268]
Microbial SCFAs (valerate/butyrate) mediate HDAC inhibition and mTOR activation	Valerate, Butyrate, Class I HDACs	Microbial metabolites (valerate/butyrate) inhibit HDACs and activate the mTOR pathway	Megasphaera massiliensis produces valerate, with abundance correlated to SCFA levels	Enhances CD8⁺ T‐cell effector function (IFN‐γ, TNF‐α) and antitumor reactivity, improving the immunosuppressive microenvironment	HDAC inhibition and mTOR–S6 pathway activation	Melanoma; PDAC	[19]
Microbiota–metabolite–immunotherapy synergy axis	Butyrate	Gut microbiota‐derived butyrate optimizes cytotoxic T‐cell function via epigenetic and signaling mechanisms, enhancing sensitivity to PD‐1 blockade	*Akkermansia*, *Faecalibacterium*	CD8⁺ T cell–dependent enhancement of anti–PD‐1 efficacy, leading to tumor regression and prolonged survival; effect independent of CD4⁺ or γδ T cells	Microbiota–immune interactions, immune checkpoint blockade	NSCLC; Melanoma	[42]
Microbial metabolite–p53 interactions	Mutant p53, Gallic acid	Gut microbiota‐derived gallic acid determines the functional outcome of mutants p53	Production of gallic acid as a microbial metabolite	In proximal gut: suppresses tumorigenesis (in germ‐free or GA‐depleted conditions); promotes malignant phenotype (with microbiota or GA present)	Mutant p53 disrupts WNT signaling by blocking TCF4–chromatin binding; gallic acid restores TCF4–chromatin interaction and WNT hyperactivation	WNT‐driven intestinal cancer (e.g., Apc Min/^+^ or Csnk1a1‐deficient mouse models)	[269]
X‐chromosome inactivation (XCI) in alveolar type 2 cells (AT2s) lacks canonical Xist RNA clouds and heterochromatin modifications	Xist lncRNA	XCI escape alters expression of X‐linked genes (e.g., ACE2), influencing lung physiology and pathology	XIST downregulation enhances stemness and plasticity of ovarian CSCs	XIST loss promotes enrichment of epithelial‐ and mesenchymal‐like CSCs	X inactivation affects sex‐specific tumor traits, stemness, and plasticity	BrCa, OvCa	[270, 271]
Low‐CpG Transposon Silencing	LINE‐1 promoter methylation/de novo DNA methylation	Low CpG modification suppresses TLR9‐mediated transposon recognition and inflammatory response	Reduces plasmid‐induced inflammation	Inhibits tumor growth and increases CD4⁺ CAR‐T cells and TILs	Transposons enhance CD4^+^ CAR‐T cell infiltration, suppressing tumor growth	CRC	[272, 273]
Microbe‐mediated miRNA loss and autophagy activation	TLR4, MYD88, miR‐18a, miR‐4802, ULK1, ATG7	*Fusobacterium nucleatum* acts on host cells via the TLR4/MYD88 pathway	Enrichment of opportunistic pathogens such as *F. nucleatum*	Induces autophagy and promotes chemotherapy resistance	TLR4/MYD88 signaling, miRNA regulation, autophagy pathways	CRC	[274]

Abbreviations: 4‐HB, 4‐Hydroxybenzoic acid; cHL, classical Hodgkin lymphoma; EBV, epstein‐Barr virus; Ezh2, enhancer of zeste homolog 2; HATs, histone acetyltransferases; ICIs, immune checkpoint inhibitors; lncRNAs, long non‐coding RNAs; LMP1, latent membrane protein 1; LPS, lipopolysaccharide; MDSCs, myeloid‐derived suppressor cells; MM, multiple myeloma.

In the one‑carbon cycle, the metabolism of serine, glycine, and methionine generates SAM, which directly drives the methylation of DNA and histones. These metabolic substrates not only establish chromatin modification patterns but also closely associate with tumor‐cell proliferation and survival through signaling pathways like PI3K–AKT–mTOR (Figure 3).

**Figure 3:**
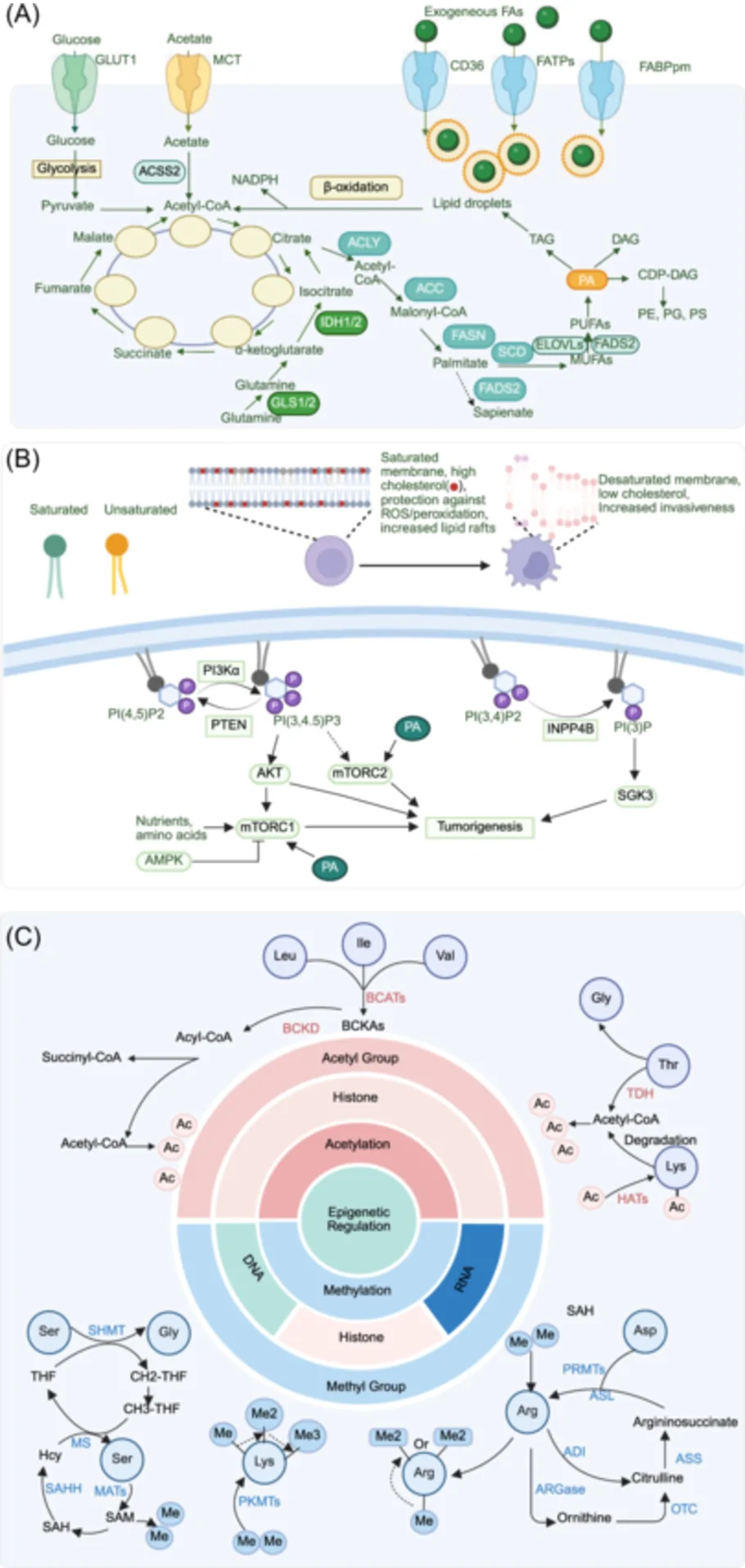
Metabolic pathways provide substrates and regulatory inputs for epigenetic modifications and signaling pathways. (A) Acetyl and methyl groups derived from carbohydrate and lipid metabolism supply essential substrates during tumorigenesis. Metabolic pathways not only generate substrates but also regulate epigenetic modifications and oncogenic signaling. Carbohydrate and lipid metabolism produce acetyl‐CoA, which provides acetyl groups for histone acetylation and supports fatty acid synthesis. (B) These epigenetic modifications converge with signaling networks, including the PI3K–AKT–mTOR pathway and the energy‐sensing AMPK pathway, which is depicted as a nutrient‐responsive regulator that counterbalances mTOR activity, to regulate cell proliferation, survival, and differentiation. In the upper left corner, different colors are used to represent saturated fatty acids and unsaturated fatty acids, indicating that the physical and chemical properties of these two types are significantly different. (C) Amino acid metabolism, particularly involving serine and glycine, supplies one‐carbon units required for SAM‐dependent DNA and histone methylation. Acetyl‐CoA, acetyl coenzyme A; AKT, protein kinase B; AMPK, AMP‐activated protein kinase; mTOR, mechanistic target of rapamycin; PI3K, phosphoinositide 3‐kinase; SAH, S‐adenosyl‐L‐homocysteine; SAM, S‐adenosyl‐L‐methionine.

#### Nucleotide metabolic pathways

Microorganisms can synthesize a variety of small molecules involved in nucleotide metabolism, significantly influencing host immunity and epigenetics. Mechanisms linking nucleotide signaling to innate immune pathways can improve sensitivity to immunotherapy.

Purine nucleotide release: *Streptococcus sanguinis* secretes exogenous nucleotidases that degrade ATP and guanosine triphosphate into nucleosides. These nucleosides act on the adenosine A2A receptor to regulate T‐cell function and chromatin accessibility [275]. CDN synthesis: *Listeria monocytogenes* and *Vibrio cholerae* possess diguanylate cyclases that synthesize c‐di‐GMP and c‐di‐AMP. These pathogen‐associated molecular patterns activate the STING pathway and induce histone modifications such as trimethylation of histone H3 lysine 27 (H3K27me3) [276, 277]. NAD^+^ cycle and derivative regulation: *Bifidobacterium adolescentis* and *Lactobacillus brevis* synthesize NAM, nicotinamide mononucleotide, and related molecules or inhibit their degradation, thereby regulating host SIRT and PARP activity and influencing histone acetylation and ADP‐ribosylation [278].

Given that nucleotide metabolism modulates immune‑cell response thresholds and participates in chromatin remodeling, DNA repair, and aging pathways, it is likely involved in tumor epigenetic processes, as illustrated in this study. Metabolites from different origins, such as lactate, SCFAs, acetate, BAs, and succinate, bridge metabolic supply with epigenetic modification, regulating DNA methylation, histone acetylation, and ncRNA networks. These metabolites not only induce and drive specific tumor types but also modulate systemic homeostasis via axes such as the gut‐brain axis, and immune–metabolic axis (Figure 4).

**Figure 4:**
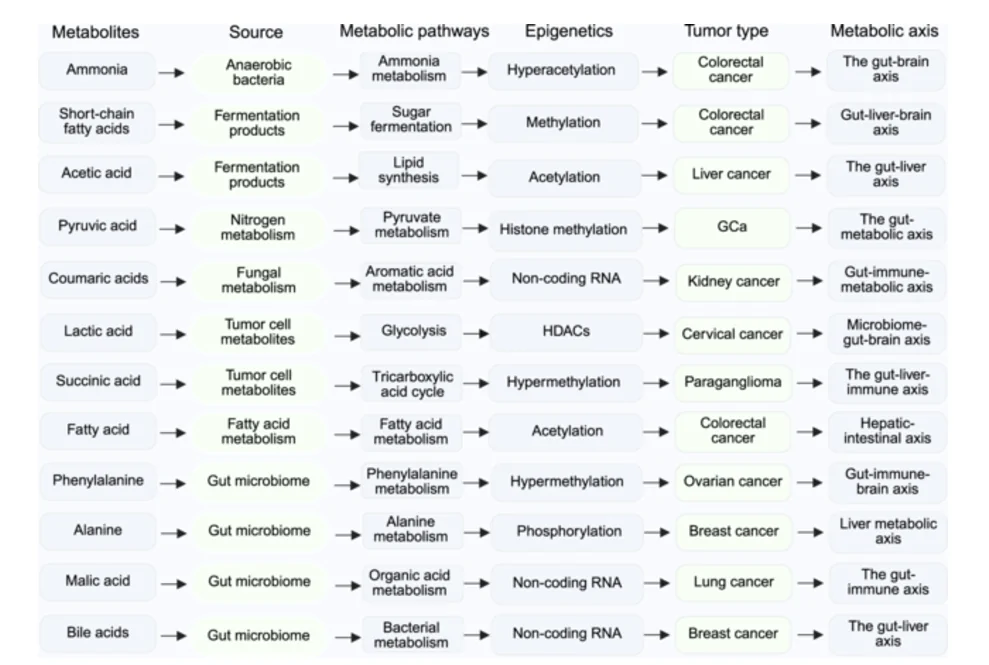
Representative microbially and tumor‐derived metabolites, their metabolic origins, associated epigenetic modifications, tumor types, and systemic metabolic axes. Various metabolites derived from tumor cells, gut microbiota, or fermentation‐related pathways link metabolism to epigenetic remodeling and tumorigenesis. Lactic acid, SCFAs, acetic acid, pyruvic acid, and ammonia are associated with histone acetylation or methylation through glycolytic, fermentation, lipid, or nitrogen‐related metabolic pathways, contributing to the development of cervical, colon, liver, and GCa. In addition, BAs, succinic acid, fatty acids, phenylalanine, alanine, malic acid, and coumaric acid are involved in DNA and histone modifications or the regulation of ncRNAs, thereby contributing to the pathogenesis of breast, colorectal, ovarian, lung, and kidney cancers. These metabolites act through systemic metabolic axes, including the microbiome–gut–brain, gut–liver, gut–immune, and gut–metabolic axes, highlighting their integrative roles in epigenetic regulation and tumor progression. GCa, gastric cancer; ncRNA(s), non‐coding RNA(s).

### Functional characteristics of microbial metabolites in tumors

Microbial metabolites are not merely metabolic substrates or cofactors; they actively modulate tumor signal transduction, metabolic reprogramming, and the immune microenvironment via multiple molecular mechanisms (Figure 5). For example, secondary BAs and SCFAs regulate tumor signaling via the Wnt/β‐catenin and PI3K/AKT/mTOR pathways. Lactate influences energy metabolism through monocarboxylate transporters (MCTs) such as M2 and M4. Indole derivatives and related metabolites can induce oxidative stress and endoplasmic reticulum stress. Additionally, other microbial metabolites shape tumor‐cell fate decisions by remodeling the immune microenvironment and altering the immune regulatory landscape.

**Figure 5:**
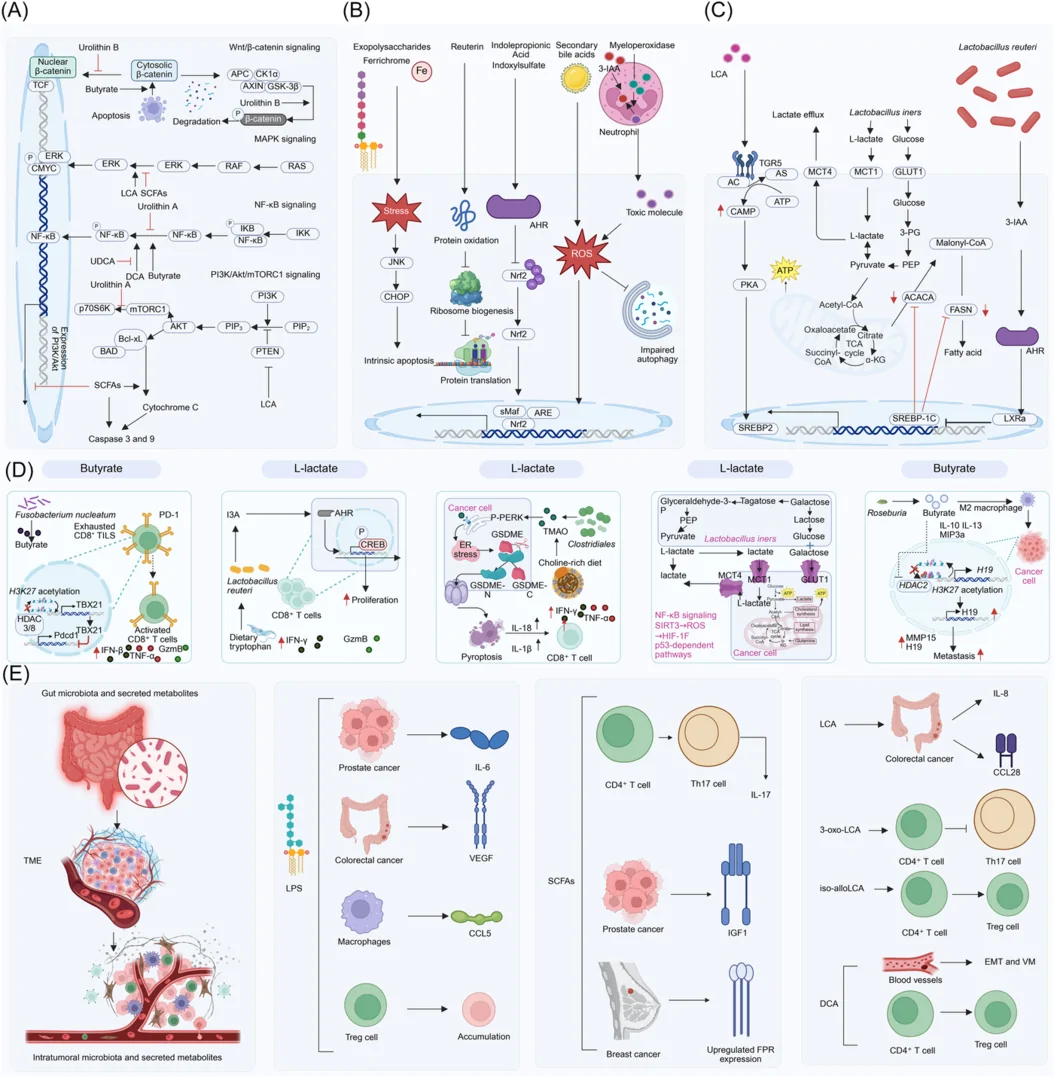
Intestinal microbiota and their metabolites regulate tumor signaling, metabolic reprogramming, and the immune microenvironment. (A) Secondary BAs (LCA/DCA) and SCFAs (butyrate) modulate canonical pathways such as Wnt/β‐catenin, MAPK/ERK, NF‐κB, and PI3K–AKT–mTOR, influencing apoptosis and proliferation. (B) Exopolysaccharides, reuterin, indole derivatives, and secondary bile acids activate JNK, protein oxidation pathways, AhR/Nrf2‐mediated transcriptional activation via ARE, and ROS signaling, respectively, thereby triggering oxidative stress responses. (C) Lactic acid secreted by *Lactobacillus* or cancer cells regulates metabolic reprogramming via MCT‐mediated transport and cAMP/PKA‐CREB signaling, thereby influencing mitochondrial oxidative metabolism through COX5. (D) At the cellular level, butyrate enhances CD8^+^ T cell effector functions through histone deacetylase inhibition, while lactic acid rewires tumor metabolism and inflammasome activity. (E) Gut and intratumoral microbiota collaboratively remodel the TME, modulating immune cell polarization (Th17/Treg), cytokine networks, angiogenesis, and metastasis. AhR, aryl hydrocarbon receptor; COX5, cytochrome c oxidase subunit 5; EPS, exopolysaccharides; HDACs, histone deacetylases; MCTs, monocarboxylate transporters; NF‐κB, nuclear factor kappa B; Nrf2, nuclear factor erythroid 2–related factor 2; ROS, reactive oxygen species; TME, tumor microenvironment; Tregs, regulatory T cells.

#### Epigenetic enzyme regulation

Microbial metabolites can remodel chromatin states and gene expression in tumor cells by directly or indirectly regulating epigenetic enzymes. Their regulatory mechanisms include the following types: a. Direct inhibition/activation: SCFAs such as butyrate and propionate can enter the nucleus and competitively inhibit HDAC activity. This leads to increased histone acetylation and transcriptional activation of classical TSGs such as *p21*, *BAX*, and *CDH1*. On the other side, butyrate works as an HDAC inhibitor (HDACi) to indirectly promote the assembly and activity of HATs by elevating global histone acetylation, thereby modulating the acetylation of genes involved in apoptosis and differentiation [279]. b. Allosteric regulation or interaction: Polyamines like spermidine can interact with histone methyltransferases (HMTs) or DNMTs, inducing conformational changes that modulate their enzymatic activity toward substrates. This fine‐tunes target‐gene expression by altering histone H3 lysine 4 trimethylation (H3K4me3) levels and the methylation status of promoter regions [280]. c. Cofactor or competitive inhibitor: BA derivatives can act as cofactors or competitive inhibitors of TET demethylases, thereby regulating the conversion of 5‐methylcytosine (5mC) to 5‐hydroxymethylcytosine (5hmC). This process plays an important role in the derepression of epithelial–mesenchymal transition (EMT)‐related genes [281]. d. Supply of substrates or precursors: NAM, synthesized by gut microbes, serves as a precursor of NAD⁺ and fine‐tunes the activity of SIRT family deacetylases. This regulates the acetylation status of target genes involved in the cell cycle and DNA repair, influencing genomic stability and therapy resistance [282].

In summary, microbial metabolites precisely modulate the activity of epigenetic enzymes through diverse regulatory pathways, thereby shaping tumor phenotypes and therapeutic responses.

#### Chromatin remodeling capacity

Microbial metabolites can not only directly modulate epigenetic enzyme activity but also affect the assembly and function of chromatin remodeling complexes through direct or indirect regulation, thereby altering nucleosome positioning and chromatin accessibility. For instance, SCFAs like butyrate can inhibit HDAC activity and increase histone acetylation levels, which in turn enhances the recruitment of chromatin remodelers like switch/sucrose non‑fermentable (SWI/SNF) to target gene promoters. Increased histone acetylation loosens chromatin structure at promoters of pro‐apoptotic and differentiation‑related genes, facilitating their transcription. On the contrary, when the concentration of butyrate is high, at high concentrations, butyrate may promote the activity of ISWI‑family complexes, accelerating nucleosome assembly and repressing the expression of proliferation‑associated genes [283].

Polyamine metabolites like spermidine and spermine can neutralize the negative charges of DNA and histone tails and therefore enhance the stability of nucleosomes within promoter regions, silenced loci, and heterochromatin domains. Such stabilization not only protects chromatin from external perturbations but also provides binding sites for substrate‐recognition proteins of CHD family chromatin remodeling enzymes, facilitating dynamic interconversion between transcriptionally active and repressive genomic regions [5, 284, 285, 286].

In addition, lactate can serve as a donor for Kla, mediating lactylation at specific histone residues like H3K18. Histone lactylation marks create open chromatin states, mainly in regulatory regions of inflammatory‐response and tissue‐repair genes. These marks facilitate the recruitment of bromodomain‐containing transcriptional activators or alleviate transcriptional repression by modulating higher‐order chromatin architecture, which in turn promotes the expression of IL‐10 and other immune‐regulatory factors through lactate‐driven signaling [287, 288]. In tumor models, histone lactylation is closely related to the metabolic state of tumors and confers transcriptional plasticity and adaptability to tumor and immune cells under microenvironmental stresses like hypoxia and lactate accumulation.

Through substrate provision, enzyme activity regulation, and cooperation with chromatin remodelers, metabolites secreted by tumor‐associated microbiota govern the spatial reorganization and functional partitioning of chromatin. This represents a key mechanism for reshaping the tumor epigenetic landscape [289, 290, 291, 292, 293, 294]. The aberrant histone modification profiles frequently observed across tumor types and their relationships with chromatin remodeling are summarized in Table 9.

**Table 9:** Overall patterns of abnormal histone modifications in cancer.

Type of modification	Modified sites	Function/mechanism	The role in cancer	Relevant cancer types	References
Lactylation	H3K18	Activation of gene transcription (e.g., TTK, BUB1B), forming a positive feedback loop	High expression associated with poor prognosis in PDAC, forming glycolysis‐H3K18la‐TTK/BUB1B positive feedback promoting cancer	PDAC	[13]
Histone acetylation	H3K9ac and H3K14ac	Acetylation modification activates TNF‐α gene transcription	Induces apoptosis under the action of anticancer agents, inhibiting tumor progression	CRC	[295]
Methylation	H3K9 and H3R17	CARM1 catalyzes dimethylation, bidirectionally regulating gene transcription	High expression indicates poor prognosis and shorter survival in GCa patients, independent prognostic factor for survival	GCa	[296, 297]
Ubiquitination	H2AK119, H3K27	PRC1/2‐mediated gene silencing (e.g., Ptf1‐L), driving cell dedifferentiation	Epigenetic silencing of acinar cell fate genes, promoting ADM and tumor initiation	PDAC	[298]
Acetylation	H3K4, H3K27	Regulation of its levels by PI3K/Akt and Wnt/β‐catenin signaling pathways	Promotion of epithelial–mesenchymal transition (EMT) and cancer progression	GCa	[299]
Acetylation	Histone H3, Histone H4	SAHA induces overexpression, recruits to promoters of genes such as *p21*, regulating gene transcription	Increased expression during GCa development, positively correlates with GCa cell differentiation	GCa	[300]
Lactylation	H3K9la, H3K56la	Promotes tumorigenesis; can be inhibited by DML	Promotes HCC development, with abnormal levels associated with HCC progression	HCC	[301]
Methionine oxidation	M239	MSRA reduces the oxidized form of PKM2 M239; M239 oxidation maintains the active tetrameric state of PKM2	Promotes PC respiration, migration, and metastasis	PDAC	[302]
Serotonylation (H3Q5ser)	H3Q5	Promotes tumor progression via MYC signaling pathway	Drives HCC Progression	HCC	[303]
Desuccinylation	Thr758, Lys176	PPA2 dephosphorylates and activates NEDD4, promoting HIF‐1α ubiquitination degradation; SIRT5 desuccinylates PPA2, causing its dissociation from NEDD4	PPA2 inhibits CRC metastasis through HIF‐1α degradation	CRC	[304]
Serotonylation	H3K4me3Q5ser	Drives lipid metabolism reprogramming by upregulating SCD expression	Promotes the development and progression of PDAC	PDAC	[305]
Trimethylation	H3K27	Associated with EZH2 expression, correlates with overall survival in spinal cord glioma patients	H3K27me3 retention + EZH2 negativity indicates favorable prognosis, serves as a potential prognostic marker	Diffuse Spinal Cord Glioma	[306]
SUMOylation and Desumoylation	K154	SENP3 mediates desumoylation, stabilizing SIX1 protein; SUMOylation modulates protein stability	Promotes PC cell proliferation and migration, leading to metastatic malignant progression	PCa	[307]
Acetylation; Phosphorylation	Lys	Involved in the anti‐CRC effects of FK228, with crosstalk between acetylation and phosphorylation modifications	Mediates the antitumor activity of histone deacetylase inhibitors (HDACis)	CRC	[308]
Crotonylation	H3K27	LINC00922 binds SIRT3, preventing its binding to the ETS1 promoter, increasing H3K27cr to activate ETS1 transcription, regulating CAMs	Promotes epithelial cell migration and invasion, leading to CRC metastasis	CRC	[309]
Methylation (Trimethylation)	H3K27me3	Establish restricted chromatin states	Aberrant gene repression	Aggressive lymphomas	[310]
Histone crotonylation	Lys	BRD4 regulates histone crotonylation levels and androgen receptor signaling pathway	Promotes PC cell proliferation, migration, and invasion	PC	[311]
Ubiquitination‐deacetylation crossover	K526/K531	HDAC6 deacetylates AKAP12, promoting its ubiquitination and proteasomal degradation	HDAC6 promotes, AKAP12 inhibits CRC metastasis	CRC	[312]
Phosphorylation‐ubiquitination crossover	S166	TPI1‐AKT‐MDM2 complex enhances MDM2 phosphorylation, promoting p53 ubiquitination and degradation	Promotes BC cell growth, migration, and invasion, leading to poor prognosis	BrCa	[313]
Deubiquitination methylation	H2AK119ub; H3K36me2	PSMD14 deubiquitinates H2AK119, promoting NSD2‐mediated H3K36 dimethylation	Activates oncogene transcription, driving myeloma development	MM	[314]
Lactylation	H3K14la	Regulates gene expression and promotes NEDD4‐mediated PTEN degradation	Drives chemotherapy resistance and malignant tumor progression	HCC	[315]
Methylation	H3K27	EZH2 catalyzes H3K27me3 to inhibit LATS2 transcription	Promotes proliferation and invasion of lung adenocarcinoma cells and inhibits apoptosis	LuCa	[316]
Lactylation	H4K12	Lactylation of lysine can regulate key target proteins, affect signaling proteins and metabolic enzymes, and alter protein stability.	Positively correlated with poor prognosis and an independent prognostic marker	TNBC	[317]
Citrullination	Arg	Mediates transcriptional activation of hypoxia response genes	Promotes tumor growth and angiogenesis	BrCa, LuCa	[318]
Phosphorylation	H2AT120	Drives transcriptional repression and promotes cell proliferation	Promotes the initiation and progression of CRC	CRC	[319]
Ubiquitination	H2B	Upregulates CCNB1 to enhance H2B ubiquitination	Promotes tumor progression	ESCA	[320]
Phosphorylation	H3T11	Co‐activates SERPINE1 expression with H3K27ac	Promotes metastasis of TNBC	TNBC	[321]
Serotonylation and Citrullination	H3Q5, H3Arg	Triggers chromatin decondensation and promotes the formation of neutrophil extracellular traps (NETs)	Promotes hepatic metastasis of neuroendocrine carcinoma	Neuroendocrine pancreatic cancer (PDAC subtype), small cell LC, medullary thyroid cancer (TCa)	[322]

Abbreviations: EMT, epithelial–mesenchymal transition; HDACis, histone deacetylase inhibitors; NETs, neutrophil extracellular traps; PRC, Polycomb repressive complex; TCa, thyroid cancer.

#### Regulation of genomic stability

The relationship between microbial metabolites and genomic stability operates as a feedback loop. On one hand, microbial metabolites help maintain genomic stability through epigenetic modulation and the regulation of DNA repair factors. For example, butyrate—acting as a HDACi—induces chromatin relaxation and promotes the recruitment of DNA damage repair proteins such as γH2AX and RAD51 damage sites, thereby enhancing homologous recombination repair efficiency [323]. Conversely, disruption of the gut microbiota in cisplatin‐resistant ovarian cancer (OvCa) is associated with increased chemoresistance, which may stem from impaired DNA damage repair pathways [324].

Imbalances in gut microbiota and their metabolic byproducts can compromise genomic stability. Specific microorganisms convert dietary components into genotoxic agents or produce cytolytic compounds—including certain bacterial toxins—that directly induce DNA strand breaks and sequence alterations [325]. Furthermore, chronic inflammation caused by such dysbiosis raises levels of reactive oxygen and nitrogen species, promoting the accumulation of DNA lesions. In the context of liver pathologies, these dysbiosis‐associated signals might activate certain host receptors, thereby impairing autophagic clearance and attenuating DNA repair abilities. Collectively, these interconnected effects exacerbate genomic instability and drive the progression of neoplastic lesions [146]. Thus, microbial metabolites act as key environmental modulators of cellular genomic integrity, operating through the fine‑tuning of DNA repair pathways while simultaneously imposing persistent genotoxic stress.

In addition to preserving genomic stability, microbial metabolites coordinate a multilayered regulatory network that connects DNA integrity maintenance to immune‐epigenetic crosstalk. SCFAs, especially butyrate, inhibit class I HDACs and enhance histone H3 acetylation at promoters of immune‐effector genes. This elevates CD8^+^ T cell cytotoxicity and chimeric antigen receptor T‑cell (CAR‑T) activity, accompanied by increased interferon‑γ (IFN‑γ) and tumor necrosis factor‑α (TNF‑α) production [326]. Conversely, secondary BAs like DCA and lithocholic acid act as endogenous ligands for the FXR/TGR5 axis, inducing DNA methylation and histone modifications at *FOXP3* loci that preferentially drive Treg polarization [327]. These antagonistic yet complementary effects establish a dynamic metabolite–epigenetic–immune axis, which simultaneously upholds genomic stability and shapes immune programming within the TME in response to neoplastic progression.

#### Regulation of cell fate decisions

Microbial metabolites profoundly regulate the fate decisions of both tumor and immune cells through integrated epigenetic and signaling cascades. In tumor cells, these metabolites primarily modulate the balance between proliferation, differentiation, and death. In particular, the SCFA butyrate acts as a HDACi, enhancing histone acetylation and leading to cell‐cycle arrest and apoptosis. It also promotes the expression of differentiation‐related genes (e.g., *p21, p27*), thus forming a more mature phenotype in tumor‑cell subpopulations [328]. On the other hand, polyamine metabolites like spermidine can activate DNMTs and HMTs, leading to repressive methylation marks at specific gene promoters. This epigenetic silencing supports the maintenance and expansion of cancer stem cell (CSC)‐like properties [329].

Microbial metabolites play an important role in regulating the polarization and functional phenotypes of immune cells through epigenetic mechanisms and receptor‐mediated signaling. Polyamines, for example, promote dendritic cell development while simultaneously restraining excessive T‑cell activation, thereby moderating hyperactive immune responses. Moreover, lactate‑maintained histone Kla in macrophages helps balance the M1/M2 polarization states, which determine pro‐inflammatory versus anti‐inflammatory phenotypes and, in turn, indirectly shape the dynamic equilibrium of the TME [330].

T‑cell proliferation is suppressed by nucleotide‐derived metabolites including adenosine, which signals through the adenosine A2A receptor pathway. Concurrently, adenosine upregulates *FOXP3* expression in Tregs, thereby reinforcing their immunosuppressive function [331].

The collective action of microbial metabolites on cell fate involves the modulation of epigenetic enzyme activity, chromatin accessibility, and immune‐receptor signaling. Together, these alterations influence the complex mechanisms governing tumor and immune cell proliferation, differentiation, apoptosis and immune polarization. Interestingly, gut microbial metabolites can simultaneously promote anti‐tumor immunity and induce immunosuppressive states by acting on immunoregulatory pathways, thereby modulating the efficacy of immunotherapy. The molecular processes by which microbial metabolites control the tumor immune microenvironment and shape therapeutic responses are illustrated in Figure 6.

**Figure 6:**
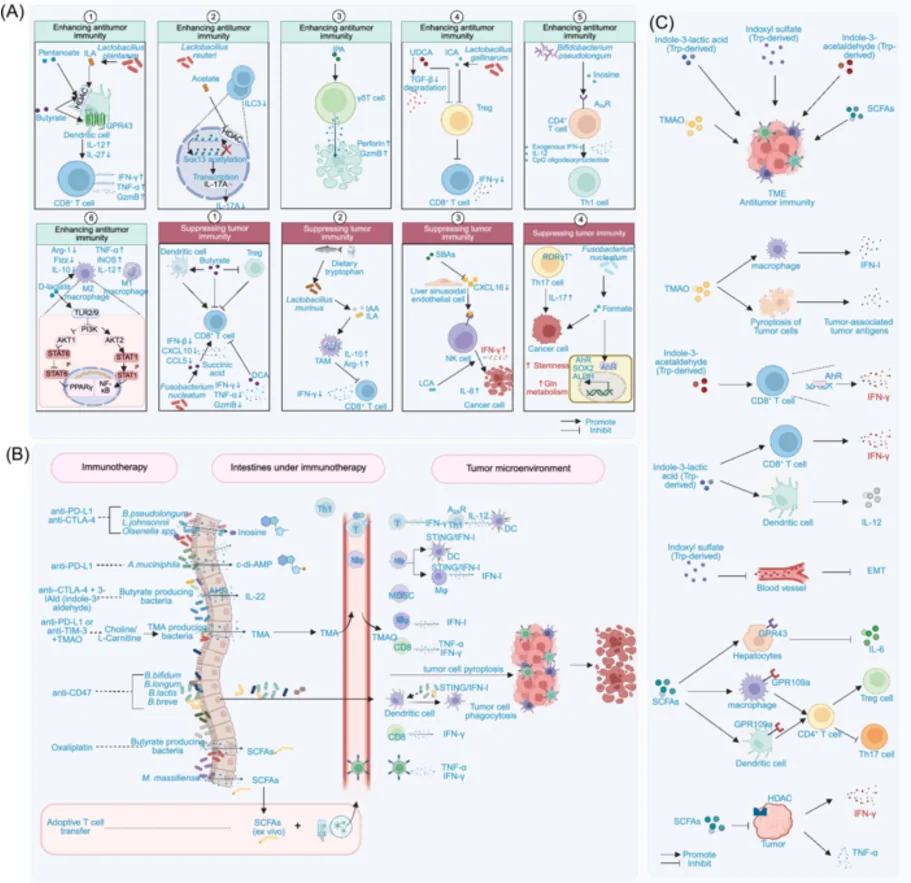
Multiple mechanisms by which intestinal microbiota and their metabolites regulate the tumor immune microenvironment and influence immunotherapy efficacy. Intestinal microbiota‐derived metabolites exert bidirectional regulatory effects on tumor immunity and immunosuppression. (A) This panel illustrates how microbial metabolites modulate antitumor immune responses, particularly affecting CD8^+^ T cells, regulatory T cells, and other immune cell subsets. (B) This panel depicts the interactions among immunotherapy, intestinal microbiota under immunotherapy conditions, and the TME, and indicates that the upstream signals or molecular mechanisms leading to STING/IFN‐I activation in the tumor microenvironment are currently not fully elucidated. (C) This panel summarizes the bidirectional effects of intestinal microbiota metabolites on tumor immunity and immunosuppressive pathways within the TME. A2aR, adenosine A2A receptor; AhR, aryl hydrocarbon receptor; Arg‐1, arginase‐1; EMT, epithelial–mesenchymal transition; Fizz, found in inflammatory zone (Fizz1/RELMα); GPR43, G protein‐coupled receptor 43; GPR109a, G protein‐coupled receptor 109A; IFN‐I, type I interferon; IFN‐γ, interferon gamma; iNOS, inducible nitric oxide synthase; MDSC, myeloid‐derived suppressor cell; STING, stimulator of interferon genes; TAM, tumor‐associated macrophage; TNF‐α, tumor necrosis factor alpha.

#### Integrated regulation of the Wnt/β‐catenin pathway by multiple microbial metabolites

Wnt/β‐catenin signaling cascade is a central oncogenic pathway in many cancers and a major convergence point for microbial metabolic signaling. Several categories of metabolites collectively modulate the activation threshold and downstream transcriptional programs of this pathway through layered mechanisms, including epigenetic modification, receptor‐mediated signaling, and protein‐stability regulation. SCFAs such as butyrate inhibit HDACs, increase acetylation at Wnt‐target‐gene promoters, and disrupt β‐catenin complexes, thereby suppressing signal transduction and tumor stemness—a mechanism consistent with potential tumor‐preventive actions [332]. On the other hand, secondary BAs, including DCA, stimulate the FXR/TGR5‐PI3K/AKT axis, promoting β‐catenin nuclear translocation and inducing the expression of oncogenes (e.g., *MYC* and *CCND1*). They also enhance EGFR phosphorylation and activate the RAS‐RAF‐ERK1/2 cascade, collectively driving cell proliferation and tumor progression [333]. Furthermore, under conditions of elevated glycolysis and hypoxia, lactate‐derived H3K18la opens chromatin conformation, facilitating β‐catenin‐dependent transcription and synergistically amplifying the expression of proliferation‐ and DNA‐repair‐related genes. This illustrates the epigenetic plasticity enabled by metabolic reprogramming.

Beyond their fundamental metabolic functions, amino acid‑derived metabolites significantly influence key signaling pathways. For instance, polyamines can remodel chromatin architecture, modulate epigenetic marking in regulatory zones, and amplify the activity of critical signaling hubs. When dysregulated, such modulation often cooperates with other malfunctioning pathways—such as the PTEN–PI3K–mTORC1 and RAS cascades—to collectively drive uncontrolled proliferation in susceptible cellular contexts [334]. On the flip side, derivatives such as indole‐3‐acetic acid (IAA) naturally bind to specific receptors, triggering the breakdown of these signaling molecules and thereby attenuating pathway output, which in experimental models restrains tumor growth [335]. Together, these interactions highlight how cellular processes balance between pro‑growth and growth‑restrictive signals, with disruption of this equilibrium favoring oncogenic progression.

Collectively, compounds derived from carbon sources (including SCFAs, lactate, and BAs) alongside substances originating from amino acids (such as polyamines and indole derivatives) create a two‐way control over the accumulation and activity of key signaling proteins. This is achieved through a tripartite mechanism involving “transcriptional regulation ↔ receptor‑mediated signaling ↔ post‑translational stability control.” This network of biochemical‐signaling interactions helps maintain the balance between pro‑growth and growth‑suppressive signals in the tumor environments, offering, thereby providing a unified framework for developing combination therapies. In other words, by elucidating these overlapping systems—how metabolites influence signaling proteins, and how receptor activities affect protein turnover—researchers gain clearer direction for designing multi‑target treatment approaches that can be tailored to individual patient characteristics.

### Crosstalk among microbial metabolites and its epigenetic implications

Microbial metabolites influence tumor biology not only through independent epigenetic mechanisms, but also through complex interactions and cross‐regulatory networks. Taking the Wnt/β‐catenin pathway as an example, SCFAs regulate HDAC activity, while BAs regulate DNA methylation. The synergy between these metabolites can either activate or inhibit the expression of tumor‐related genes. As a natural HDACi, sodium butyrate (NaB) has pioneered a new category of chemotherapeutic drugs in oncology. Studies have confirmed that when combined with traditional therapies, it can significantly enhance therapeutic effects and reduce toxicity [336]. In addition, synergistic interactions between amino‐acid‐derived and lipid‑derived metabolites can jointly regulate the tumor‑cell cycle progression, proliferative capacity, and metastatic potential through complementary mechanisms. The multiple roles of these metabolites in the same signaling pathway highlight both the complexity of microbiota‐driven epigenetic regulation and their promising translational potential in clinical research.

The synergistic and antagonistic interactions among microbial metabolites jointly construct a dynamic and complex “metabolism–epigenetic” network that regulates chromatin accessibility and transcriptional programs. A representative antagonistic relationship exists between SCFAs and secondary BAs: SCFAs such as butyrate act as natural HDACis, promoting chromatin relaxation and reactivating TSGs. In contrast, BAs such as DCA reshape the DNA methylation map through the FXR/TGR5 axis, promoting the formation of pro‐tumorigenic transcriptional programs. In various solid tumors, downregulation of FXR is often accompanied by abnormal DNA methylation and dysregulation of the RAS pathway, indicating a cross‐coupled regulatory relationship between BA signaling and the methylation network [337, 338]. Meanwhile, lactic acid further regulates chromatin accessibility in the acetylated matrix established by SCFAs. Enhanced glycolysis leads to lactate accumulation and elevated H3K18, which drives epigenetic activation of cell‐cycle and mitosis‐related genes, illustrating a flexible epigenetic regulatory pattern driven by metabolic reprogramming [13]. These phenomena are observed across various solid TMEs characterized by hypoxia and acidosis, and are not limited by tissue of origin.

At the methyl‐ and acetyl‐donor level, amino‑acid‑ and lipid‑derived metabolites (such as polyamines and indole derivatives) coordinate the balance between DNA/histone methylation and acetylation by regulating the SAM/SAH ratio and acetyl‐CoA flux [139]. Polyamine synthesis consumes substantial SAM, which not only reduces cellular methylation ability but also triggers genome‐wide hypomethylation. Conversely, SCFAs help maintain an open chromatin state by enhancing histone acetylation. This dynamic equilibrium allows tumor cells to flexibly switch between proliferative and differentiation programs in response to microenvironmental changes. In addition, folic acid and vitamin B12, as key cofactors in one‐carbon metabolism, can counteract metabolic antagonism by replenishing substrate pools, thereby supporting DNA methylation homeostasis. Long‐term B‐vitamin supplementation has been shown to influence folate and homocysteine levels, which in turn are closely associated with methylation changes in multiple development‑ and tumor‑related genes [339]. This ternary coupling among donor supply, acceptor availability, and enzyme activity constitutes the chemical basis of metabolite interactions.

Through mechanisms such as substrate supply, receptor‑mediated signaling, enzyme activity modulation, and feedback regulation, microbial metabolites work in synergy to jointly shape the epigenetic map of tumors, determining environment‐dependent gene expression and cell fate. This system‑level interplay determines the directionality and intensity of interactions among different metabolite categories, converts metabolic signals into implementable epigenetic instructions, and lays a theoretical foundation for multi‐target combination therapies and personalized treatment strategies.

## EPIGENETIC REGULATION IN TUMORS

Tumor epigenetic regulation constitutes a dynamically evolving, multi‐layered network involving interactions among various processes including DNA modification, histone modification, ncRNA‐mediated regulation, and three‑dimensional (3D) chromatin restructuring. Cancer‐related genes' transcriptional states are jointly determined by these processes and play key roles in tumor initiation and progression. Within the TME, microbial metabolites can interfere with epigenetic processes via direct or indirect regulation of epigenetic enzyme activity, as well as by modifying the accessibility of substrates. As a result, they reshape the tumor epigenome in a manner that promotes malignancy and contributes to therapy resistance.

### DNA methylation

DNA methylation is a classic epigenetic modification that primarily regulates gene expression by adding a methyl group to the fifth carbon of cytosine in CpG dinucleotides. This manipulation can regulate transcription accurately by modifying chromatin accessibility and promoter action. The balance of DNA methylation is dynamically maintained through the opposing actions of DNMTs and TET family demethylases [340].

#### Spatiotemporal regulation of the DNMT/TET enzyme system

In normal cells, DNMTs and the TET family function synergistically to construct a precisely regulated spatiotemporal DNA methylation system. DNMT1 is mainly responsible for maintaining the methylation patterns of the newly synthesized DNA strand during S‑phase, while *DNMT3A* and *DNMT3B* establish a brand‐new methylation map during embryonic development and cell differentiation. On the contrary, the TET family initiates DNA demethylation by catalyzing the oxidation of 5mC.

However, in tumor cells, this balance is often disrupted by gene mutations and microenvironmental perturbations. Microbial metabolites such as butyrate act as HDACi, promoting chromatin relaxation and thereby indirectly impairing the binding of DNMT to chromatin, which alters methylation patterns in gene regulatory regions [341]. Meanwhile, overexpression or mutation of *DNMT3A* and *DNMT3B* can lead to abnormal hypermethylation, keeping TSG promoters in a persistently silenced state. In addition, reduced activity or expression of demethylases such as *TET2* can impede the DNA demethylation pathway, thereby hindering the reactivation of TSGs. Most importantly, microbial metabolites will further aggravate the spatiotemporal imbalance between DNMT and TET activities. Secondary BAs, for example, can directly hinder the catalytic activity of TET enzymes. Dysbiosis‑induced alterations in the SAM/SAH ratio in one‐carbon metabolism limit methyl‑donor availability and reduce the efficiency of DNA methyl transfer [265]. Consequently, a dual abnormal pattern emerges: promoter‑specific hypermethylation alongside genome‑wide hypomethylation. This epigenetic dysregulation compromises proper gene function. Given these mechanisms, recent clinical studies have increasingly focused on epigenetic enzyme inhibitors and combination strategies. DNMT inhibitors (DNMTis), exemplified by 5‐azacitidine (5‐Aza), have already been reported to reverse abnormal methylation‐mediated silencing of TSGs and restore their expression in both solid tumors and hematological malignancies. It is worth mentioning that DNMTis can exert significant anti‐tumor effects—through induction of the DNA damage response (DDR) and chromatin relaxation—even in the presence of normal *DNMT1* expression [342]. The combination of epigenetic therapy with radiotherapy holds particular promise: co‑treatment with 5‐Aza and ionizing radiation can significantly enhance tumor cell apoptosis and radiosensitivity in non‐small cell lung cancer (NSCLC) models [343].

At present, metabolic treatment strategies are being actively explored. For the hypermethylation phenotype associated with *TET* gene inactivation, researchers are studying whether supplementing α‐KG can restore enzyme activity. In IDH‐mutated gliomas, accumulation of the oncometabolite 2‐hydroxyglutarate (2‐HG) inhibits TET activity, thereby driving the formation of glioma‐CGI methylator phenotype. Currently, targeted therapies such as IDH inhibitors and epigenetic drugs are under development [344]. In addition, regulating cofactors produced by the microbiota (such as folic acid and vitamin B12) may help rebalance methyl‑donor availability, offering potential for adjunctive nutritional intervention.

Epigenetic dysregulation of the DNMT/TET system, as a core driver of tumorigenesis, provides multiple therapeutic entry points. These include small‐molecule inhibitors, combination radiotherapy and chemotherapy, as well as interventions targeting the microbiota and metabolism. These methods are expected to play an increasingly important role in precision oncology. Abnormal DNA methylation is one of the most typical markers of cancer epigenetics, characterized by focal hypermethylation of TSG promoters alongside widespread genomic and repetitive‑sequence hypomethylation. This imbalance can silence TSGs and activate oncogenes (Figure 7).

**Figure 7:**
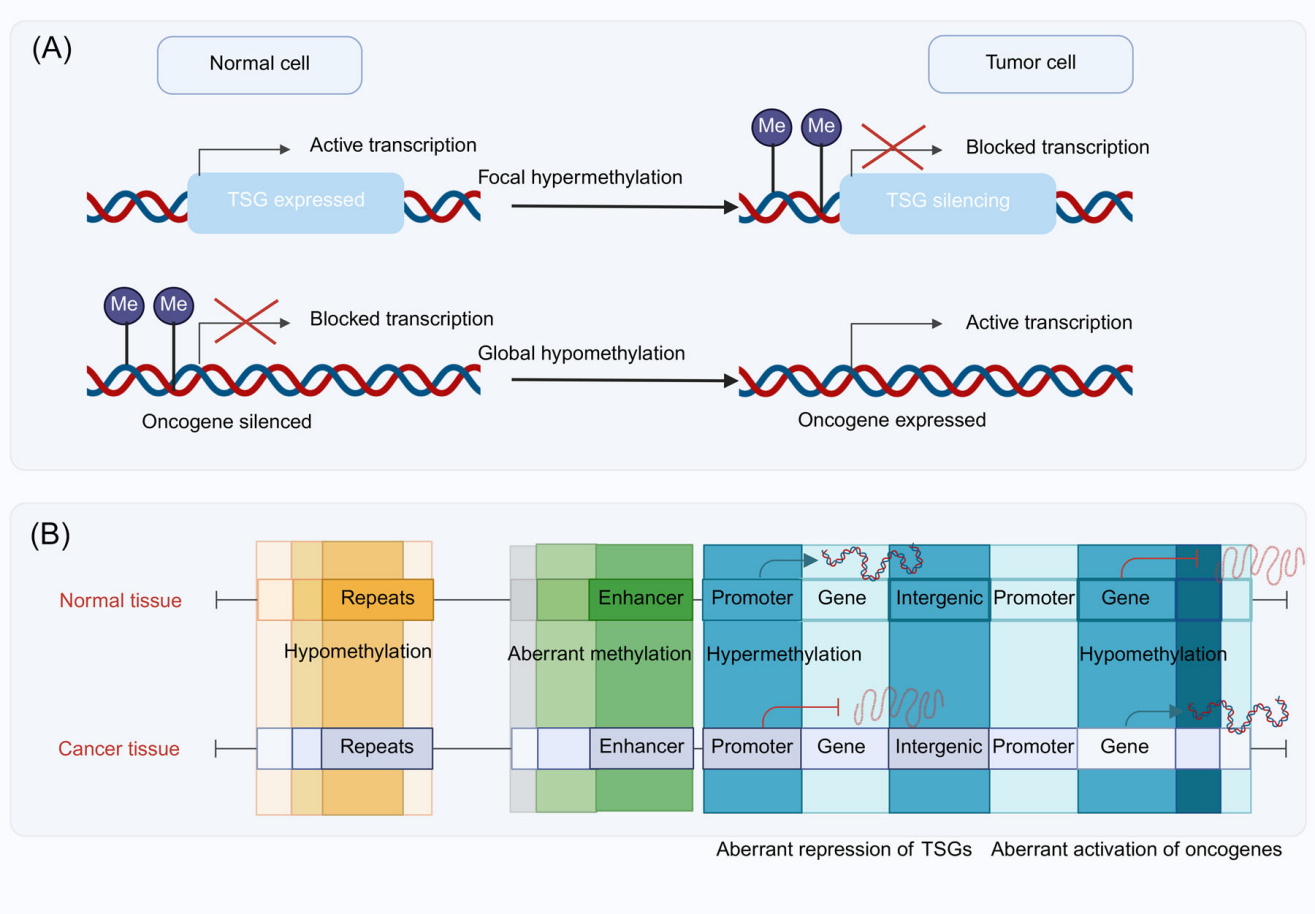
Dual dysregulation of DNA methylation in tumorigenesis. (A) In normal cells, TSGs are typically active, while oncogenes are kept in a silenced state. However, during tumorigenesis, focal promoter hypermethylation results in the silencing of TSGs, whereas global hypomethylation facilitates the activation of oncogene expression. (B) This panel compares DNA methylation landscapes across distinct genomic features, including repeat elements, enhancers, promoters, gene bodies, and intergenic regions. The upper track represents normal tissue and the lower track represents cancer tissue; shaded blocks depict relative DNA methylation levels, with higher levels indicating hypermethylation and lower levels indicating hypomethylation. Cancer tissue exhibits widespread hypomethylation of repeat elements and intergenic regions, accompanied by aberrant hypermethylation at promoter and enhancer regions. These changes contribute to inappropriate silencing of TSGs and aberrant activation of oncogene expression. Me, methylation; TSG, tumor suppressor gene.

#### Remodeling of CGI methylation landscapes

CGIs are highly enriched in the promoter regions of genes and serve as key regulatory “switches” for gene expression. In normal cells, promoter CGIs remain hypomethylated to maintain transcriptional activity, while the genome and repetitive sequences are generally hypermethylated to ensure genomic stability. However, in tumor cells, this methylation landscape has undergone extensive remodeling: CGIs in the promoters of a large number of TSGs become hypermethylated, leading to transcriptional silencing. Meanwhile, genome‑wide and repetitive‑sequence hypomethylation promotes increased in genomic instability.

Microbial metabolites significantly influence this remodeling process. Butyrate can modulate DNMT activity by altering the SAM/SAH ratio. Furthermore, BA derivatives are capable of directly impairing TET enzyme function. Altered levels of B vitamins, such as folic acid, influence the availability of methyl‐donor pools. The joint action of these factors generates spatiotemporal heterogeneity in CGI‐methylation and promotes the formation of the “CGI methylator phenotype (CIMP)”. CIMP is characterized by widespread, concurrent hypermethylation of specific CGIs across cancer types. Microorganisms play a key role in CIMP development. Earlier research indicated that specific bacterial taxa can interfere with CGI methylation, a process that can induce CIMP‑associated stemness in colorectal cancer (CRC) [345]. The heterogeneity of CIMP may help stratify cancers based on underlying driving mechanisms, thereby enabling more subtype‑specific treatment strategies [346, 347].

Clinical evidence in favor of CIMP as a biomarker across multiple cancer types has been validated in various studies and can be summarized at three levels: Diagnosis and risk assessment: Novel CIMP markers have been identified in microsatellite‐stable CRC [348]. Additionally, methylation patterns in CpG‐poor areas may serve as a monitoring markers for the risk of early CRC recurrence [349]. Treatment prognosis prediction: The promoter methylation status of O^6^‐methylguanine‐DNA methyltransferase (*MGMT*) in glioblastoma (GBM) reliably predicts response to temozolomide [350]. Recent clinical trials have also investigated the immunotherapy response of CIMP‑high tumors. Furthermore, in microsatellite instability‐high CRC, the CIMP‐high subgroup is associated with increased tumor‐infiltrating lymphocytes and overexpression of programmed death‑ligand 1 (PD‑L1), suggesting greater sensitivity to immunotherapy [351]. Prognostic stratification: Unique CIMP traits have been incorporated into prognostic stratification and therapeutic decision‐making for cholangiocarcinoma [352] and papillary thyroid carcinoma [353].

In CRC, hypermethylated tumors frequently show *MYC*‐driven overactivation of one‐carbon metabolism. The resultant enhancement of cell proliferation may be related to poor prognosis [354].

#### Epigenetic silencing mechanisms of transposable elements (TEs)

TEs, which make up about 45% of the human genome, pose a threat to genomic stability if dysregulated. In normal cells, these elements are tightly silenced by multiple mechanisms, such as DNA hypermethylation and inhibitory histone modifications (e.g., H3K9me3). Beyond these intrinsic pathways, silencing can also be regulated by microbial metabolites. For instance, folic acid and vitamin B12 can affect one‐carbon metabolism, thereby indirectly regulating the methylation efficiency of DNMTs at TE regions. On the other hand, tryptophan metabolites like indolepropionic acid may activate the AhR pathway, promoting heterochromatin formation at TEs.

Many epithelial‐derived malignant tumors manifest extensive genomic hypomethylation, as seen in CRC, oropharyngeal squamous cell carcinoma, OvCa, and esophageal squamous cell carcinoma. This hypomethylation leads to the reactivation of transposable elements, particularly long interspersed nuclear element‑1 (LINE‑1), resulting in new transposition events and increased chromosomal instability. Furthermore, SCFAs are thought to remodel chromatin at TE regions through HDAC inhibition and by interfering with the maintenance of DNA methylation and H3K9me3 [355]. Meanwhile, polyamine metabolites further modulate H3K9 methyltransferase activity, thereby influencing TE silencing [356].

Hypomethylation of LINE‐1 is especially important in early‐onset CRC [357]. LINE‐1 hypomethylation is also observed in oropharyngeal squamous cell carcinoma, where it is associated with poor prognosis [358]. Studies show that LINE‐1 insertion can inactivate important TSGs such as *RORA*, thereby LINE1 insertion [359]. Furthermore, LINE‐1 reactivation causes replication stress and DDRs [360], further exacerbating genomic instability in tumor cells. Overexpression of the LINE‐1 antisense promoter is also associated with TME remodeling, disease progression, and adverse clinical outcomes [361, 362]. On the contrary, during the malignant transformation of benign skin nevi, maintenance of methylation in repetitive sequences helps suppress malignancy [363].

Researchers have proposed the methylation level of LINE‐1 as a potential clinical biomarker for predicting the risk of metachronous CRC [364]. Research has demonstrated that expression of the LINE‐1 ORF1p protein may serve as a blood‐based biomarker for clear‐cell ovarian precancerous lesions [365]. In addition, a recent study confirmed that aberrant LINE‐1 activation drives tumor progression through the long non‐coding RNA (lncRNA) *CASC9* in esophageal squamous cell carcinoma [366].

Overall, whether this is the case remains to be determined. The causative roles of gut microbial metabolites will likely be clarified through animal models or further retrospective analysis of larger cohorts. A deeper understanding of this regulatory network may provide a theoretical foundation for transposon‐targeted strategies applicable to early cancer screening and future epigenetic interventions.

### Histone modifications

PTMs of histones, including acetylation and methylation, play an important role in regulating gene expression by altering chromatin conformation and promoting the recruitment of effector proteins. Microbial metabolites profoundly influence these dynamic modification processes. Histone modifications, including acetylation, methylation, phosphorylation, ubiquitination, ADP‐ribosylation, and SUMOylation, contribute substantially to tumor epigenetic regulation. By remodeling chromatin structure and modulating the recruitment of effector proteins, these modifications precisely regulate gene transcription and DNA repair (Figure 8).

**Figure 8:**
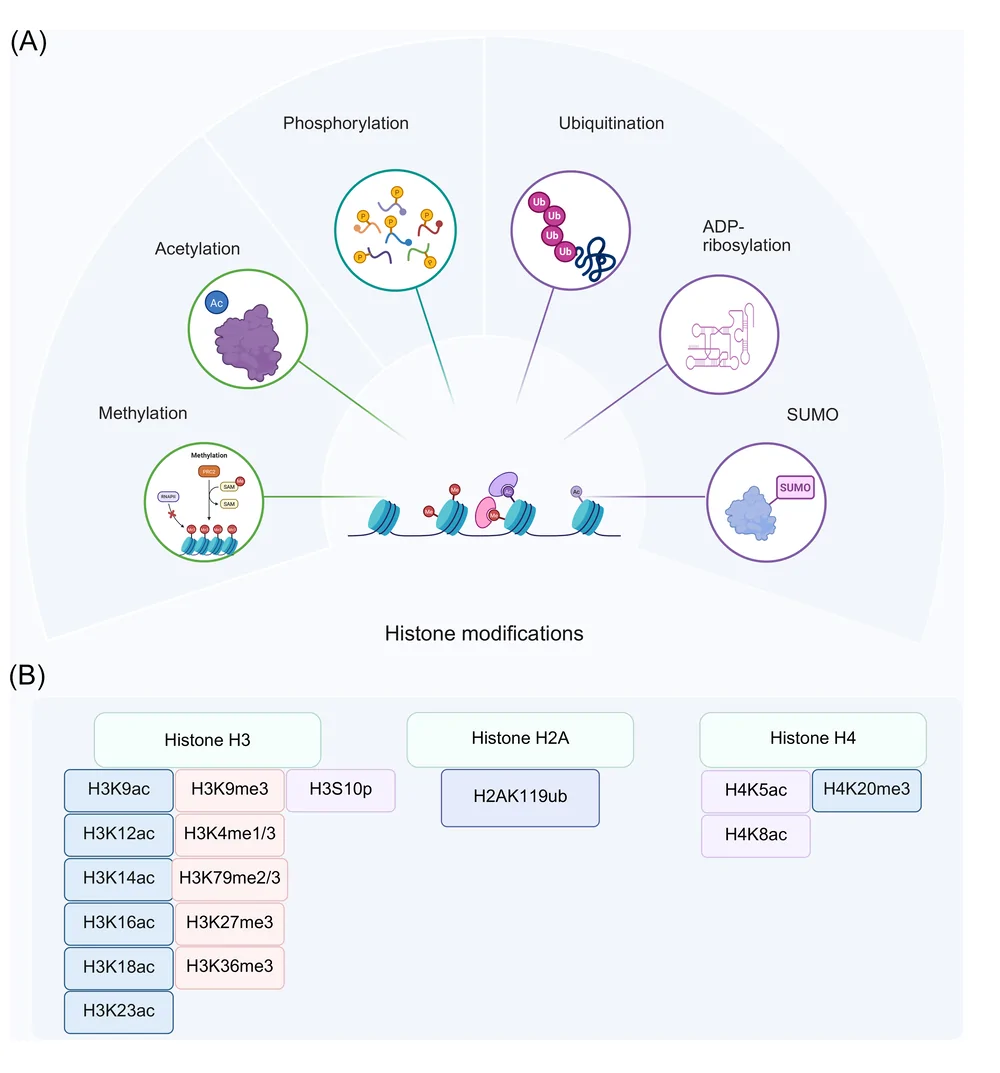
Overview of various PTM types and their main modification sites on different histone subunits. (A) This figure summarizes the primary types of PTMs, which include acetylation, methylation, phosphorylation, ubiquitination, ADP‐ribosylation, and sumoylation. (B) These modifications collectively regulate chromatin structure and gene expression. Representative modification sites include histone H3 (e.g., K9, K12, K14, K16, K18, K23, K27, K36, K79, S10), histone H2A (ubiquitination), and histone H4 (e.g., K5, K8, K20). These PTMs act as dynamic molecular switches that regulate transcription, DNA repair, and tumor progression. Color coding distinguishes specific histone modification types: In (A), distinct colors group modification categories (e.g., green for small chemical groups, purple for peptide conjugations). In (B), background shading indicates the specific type of modification on histone tails, particularly for H3: blue, acetylation sites; pink, methylation sites; purple, phosphorylation sites. PTM, post‐translational modification.

#### Acetylation dynamic balance

Histone acetylation is dynamically controlled by HATs and HDACs, which together shape the open or condensed state of chromatin. In tumor cells, this balance is often disrupted, typically manifesting as HDAC overactivity that silences TSGs, or as HAT inactivation that reduces the expression of differentiation‐promoting genes. Microbial metabolites play a key role in this imbalance. SCFAs such as butyrate act as natural HDACis and can reactivate silenced TSG networks. Furthermore, alterations in polyamine metabolites and acetyl‐CoA availability directly affect substrate accessibility for HATs. The synergistic interplay within this “microbial–metabolism–enzyme” system jointly determines the global histone acetylation state of tumor cells.

Histone acetylation patterns are closely related to tumor malignant pathways and microenvironmental characteristics [242]. Overexpression of *HBO1* can promote histone acetylation and the growth of NSCLC [367]; *NOC2L* promotes paclitaxel resistance in OvCa by inhibiting histone acetylation [368]. In addition to its histone‑specific role, the acetyltransferase *HAT1* can also promote tumor progression by acetylating the non‐histone *PGAM1* [369].

In addition, intracellular signaling pathways are closely related to histone acetylation. Defects in complex I controlled by Ca^2+^ can activate the PDP1‐PDH‐histone acetylation axis to induce endogenous radioresistance [370]. SIRT6 can inhibit the invasiveness of CRC cells by suppressing ATP‐citrate lyase (ACLY)‐dependent histone acetylation [371]. HDACis such as suberoylanilide hydroxamic acid may restore the decreased histone acetylation at the *hOAT2* locus in hepatocytes, thus enhancing sensitivity to 5‐fluorouracil (5‐FU) [372]. Mitochondrial dysfunction in CAR‑T cells can result from inhibition of histone acetylation, which impairs their anti‐tumor function [373]. *HAT1* also enhances BRD4 binding to the *PVT1* promoter, promoting gemcitabine resistance in pancreatic ductal adenocarcinoma (PDAC) [374]. Inhibitors of p300/CBP [375] and dual inhibitors of BET and HAT/p300 [376] can induce synthetic lethality or apoptosis, offering new precision‐treatment strategies for malignant tumors such as CRC.

Table 10 provides a comprehensive overview of representative epigenetic drugs targeting the acetylation/deacetylation axis, their mechanisms of action, and supporting research evidence.

**Table 10:** The role of chemically structured specific drugs in epigenetic regulation of tumors.

Chemical compound	Chemical structure characteristics	Mechanism of action	Epigenetic effects	Related tumor types	References
Decitabine	Cytosine analogue (2′‐deoxyribose)	Inhibition of DNMTs; Induces cellular senescence	Reverse DNA hypermethylation; upregulates CDKN1A/*CCND1*/CDKN2A; Induces senescence	Acute myeloid leukemia (AML), MDS, CRC	[377, 378]
Romidepsin	Cyclic tetrapeptide with a disulfide bond (pro‐drug)	1. Selective inhibition of Class I HDACs → Histone hyperacetylation → Altered gene expression → Apoptosis 2. Risk: Induction of EBV reactivation via histone acetylation	Enhanced histone acetylation	Cutaneous T cell lymphoma (CTCL), peripheral T‐cell lymphoma, extranodal NK/T‐cell lymphoma (ENKTL)	[379, 380]
Tazemetostat	Indole‐pyridine derivatives	EZH2 selective inhibitor	Reduce H3K27me3 levels, epigenetically suppress TFR2 expression, inhibit ferroptosis, and enhance sorafenib sensitivity	HCC, HTLV‐1‐Associated Diseases	[381, 382]
JQ1	Triazolobenzodiazepine core structure	BET bromodomain inhibitors (targeting BRD4)	In AML: Inhibition of BRD4 binding to the promoters of NFAT2 and *PDCD1* which encodes PD‐1, downregulates PD‐1 and Tim‐3 expression. In NUT carcinoma (NC) by inhibiting oncogene transcription (e.g., MYC) and reducing H3K27ac levels	NUT midline carcinoma (NMC), AML	[383, 384]
Ivosidenib	Isocitrate analogs (containing trifluoromethyl group)	IDH1 mutation enzyme Inhibitors	Reduce the accumulation of 2‐HG; however, it cannot correct the NADPH depletion and reduced carboxylation defects caused by mIDH1	IDH1 mutation in AML and cholangiocarcinoma	[385, 386]
Enasidenib	Quinazolinone derivatives	IDH2 mutation enzyme inhibitors; exert anti‐leukemic activity by inducing differentiation	Alleviate abnormal DNA methylation and promote differentiation of leukemia cells	IDH2 mutation in AML	[387, 388]
GSK126	Pyridine pyrimidine ketone skeleton	EZH2‐specific inhibitors	Blocking H3K27me3 modification, inducing the expression of B‐Cell differentiation and apoptosis‐related genes	DLBCL	[389]
Panobinostat	Hydroxamic acid + biphenyl structure	Broad‐spectrum HDACis (Class I/II/IV)	Synergizing with drugs (e.g., IAP antagonists or EZH2 Inhibitors), inactivating the non‐classical NF‐κB pathway, activating apoptotic pathways, inducing DNA damage (γH2A.X), and altering the transcriptome to induce apoptosis and overcome resistance	MM	[390, 391]
Valproic Acid	SCFAs (branched chain carboxylic acids)	HDACis; also act as upregulators of GM3 synthase gene (ST3GAL5), inhibiting EGFR phosphorylation and cell proliferation by increasing GM3 levels	Inhibiting HDAC, Increasing Histone acetylation; Inducing DNA hypermethylation at high concentrations over a short duration; causing DNA hypo‐methylation when combined with Temozolomide; Potentially inducing oxidative DNA damage (Increased 8‐oxo‐dG)	Glioma, epidermoid carcinoma	[392, 393]
EPZ‐5676	Purine analogues (fluorinated phenyl)	Dot1l histone methyltransferase (HMT) inhibitor	Block H3K79me2 modification and inhibit MLL fusion gene transcription	MLL rearranged leukemia	[394]
SGI‐110(Guadecitabine)	Dinucleotide (Decitabine linked to deoxyguanosine)	DNA Demethylating Agents	Reversing DNA hyper‐methylation of gene promoter CGIs in platinum‐resistant recurrent OvCa tumors (significantly reducing methylation levels)	OvCa	[395, 396]
Enasidenib (AG‐221/CC‐90007)	Quinazolinone structure	Selective inhibitor of mutant‐IDH2 enzymes	Reduces 2‐HG levels, reversing DNA and histone hypermethylation	Advanced myeloid malignancies, specifically relapsed or refractory AML with mutant‐IDH2	[397]
GSK3326595	tetrahydroisoquinoline (THIQ) derivatives (Lead compound)	Potent, selective, reversible PRMT5 inhibitor	Robust target engagement was achieved (specific histone methylation changes not detailed in clinical results)	MDS, CMML, AML	[398, 399]
LSD1 inhibitors (ORY‐1001)	Trans‐cyclopropyl group	Selective LSD1 (KDM1A) inhibitors	Inducers of Differentiation Biomarkers; Activating the NOTCH pathway, inhibiting neuroendocrine phenotype (ASCL1)	R/R AML(Iadademstat); SCLC(ORY‐1001)	[400, 401]
BIX‐01294	Quinazolinone derivatives	G9a HMT inhibitor (reducing H3K9me2 and H3K27me3 repressive marks)	Reducing H3K9me2 levels, upregulating the expression of autophagy‐related genes (LC3B, WIPI1), and differentiation‐related genes (GFAP, TUBB3) induce autophagy‐dependent differentiation of glioblastoma stem cells (GSCs)	GBM	[402, 403]
JIB‐04	Benzimidazole‐pyridone structure	KDM5A Inhibitors; Pharmacological blockers of Jumonji demethylases (Especially KDM5B)	Specifically Inducing the Accumulation of H3K4me3 at γH2AX Marked Sites, Impeding the recruitment of DNA repair factors; Blocking DNA damage repair	GBM; LuCa	[404, 405]
CUDC‐907	Hydroxamic acid + PI3K inhibitory structure	Dual inhibitors of PI3K and HDAC	Inhibiting HDAC activity leads to the downregulation of c‐Myc protein levels through the inhibiting of HDAC6. This process also results in the reduction of Akt activity; a decrease in the expression of BCL‐XL, MCL‐1, and NF‐κB p65, and ultimately induces cell apoptosis	MM; PDAC	[406, 407]
OTX015	Benzodiazepine‐based BET inhibitors	Selective inhibition of BRD2/3/4	Inducing BRD4 protein accumulation in the sAML model results in weaker apoptosis‐inducing effects and protein expression regulation compared to ARV‐825	NMC (Based on Compassionate use clinical evidence); Mentioned only as an experimental control in sAML	[408, 409]
SF1126	RGD Peptide‐conjugated compounds (targeting integrin αvβ3)	Dual Inhibition of PI3K and BRD4; inhibiting the binding of the BRD4 bromodomain to acetylated lysine residues on histone H3	Inhibiting the Epigenetic Reader Function of BRD4, thus Suppressing the expression of the MYCN gene and its associated transcriptional activity	NB, particularly the high‐risk subtype with MYCN amplification	[410]
Vorinostat	Hydroxamic acid derivatives	Pan‐HDACis, inhibiting the activity of class I and II HDACs (including HDAC1, 2, 3, and 6)	Globally increasing levels of histone acetylation, particularly the acetylation of histone H3K9, can lead to an “epigenetic sensitization” effect. This effect has the potential to activate or sensitize multiple signaling pathways, such as the Sonic Hedgehog (SHH) pathway, thereby creating opportunities for combination targeted therapy.	AML	[411]
I‐BET151	Acetylated histone mimetics (competitive inhibitors of BET protein binding to histones)	BET protein inhibitors, specifically those targeting bromodomain and extra‐terminal domain proteins, function by inhibiting the activity of BRD4 protein. This inhibition subsequently affects smoothened (Smo), leading to the suppression of the hedgehog (HH) signaling pathway	Inhibiting the binding of BRD4 to the Gli1 gene promoter region, thereby suppressing the transcriptional expression of downstream HH signaling pathway target genes (e.g., Gli1)	Medulloblastoma and other HH signaling pathway‐dependent cancers	[412]
Entinostat	Benzamide‐based HDACis	Selective HDAC3 inhibitors, when used in combination with BRAF/MEK inhibitors, such as Dabrafenib and Trametinib, synergistically inhibit ELK1 and ELK3 transcription factors. This combination broadly suppresses the expression of genes involved in DNA double‐strand breaks (DSBs) repair mechanisms, including homologous recombination (HR) and non‐homologous end joining (NHEJ)	Reducing histone deacetylation levels, altering chromatin conformation, and subsequently suppressing the transcription of DNA repair genes involved in the HR and NHEJ pathways	Melanoma, including BRAF, NRAS, NF1, or PTEN mutation subtypes	[413]
Olaparib	poly(ADP‐ribose) polymerases (PARP) inhibitors	PARP inhibitors inhibit DNA single‐strand break repair, leading to DNA replication stress and DSBs	In combination with radiotherapy, activation of the cGAS‐STING pathway induces the production of type I interferons and chemokines (CXCL9/10/11, CCL5). This process remodels the tumor immune microenvironment, enhances the recruitment and activation of CD8^+^ T cells, boosts the efficacy of ICIs, and generates distant effects	HCC (BRCA gene wild‐type)	[414]
CPI‐0610	A synthetic small‐molecule inhibitor targeting BET proteins (BRD2, 3, 4, and T) that binds to the tandem amino‐terminal bromodomains (BD1 and BD2)	Competes with acetylated histones for BET protein bromodomain binding, displacing them from chromatin and resulting in downregulation of the NF‐κB signaling pathway and oncogene transcription (e.g., MYC)	Inhibiting histone acetylation reader function, leading to a significant downregulation of mRNA expression of key pro‐inflammatory and pro‐survival genes (e.g., IL8, CCR1)	Lymphoma, including DLBCL, Follicular lymphoma, Hodgkin lymphoma, and others	[415]
DZNep	Adenosine analogues	S‐adenosyl‐L‐homocysteine (SAH) hydrolase inhibitors lead to the accumulation of SAH, which indirectly induces degradation of core PRC2 components, particularly EZH2	Reducing the protein levels of the HMT EZH2 (not mRNA), leading to a significant decrease in H3K27me3 levels	Chondrosarcoma, especially the high‐grade subtype	[416]

Abbreviations: AML, acute myeloid leukemia; CTCL, cutaneous T cell lymphoma; DSBs, double‐strand breaks; ENKTL, extranodal NK/T‐cell lymphoma; GSCs, glioblastoma stem cells; HH, hedgehog; HMT, histone methyltransferase; HR, homologous recombination; NHEJ, non‐homologous end joining; NMC, NUT midline carcinoma; PARPs, poly(ADP‐ribose) polymerases; SAH, S‐adenosyl‐L‐homocysteine; SHH, Sonic Hedgehog; Smo, Smoothened.

#### Methylation‐specific regulation

Protein methylation is tightly regulated by lysine methyltransferases (KMTs) and lysine demethylases (KDMs). The transcriptional outcome of methylation—activation or repression— depends on its specific site and degree. For instance, trimethylation of H3K4me3 is associated with transcriptional activation, whereas trimethylation of H3K9me3 and H3K27me3 typically represent repressive modifications. Consequently, the cell‐type‐specific epigenetic transcriptome is shaped by the dynamic balance between these activating and repressive marks.

Within the TME, microbial metabolites influence histone methylation through several distinct mechanisms. Direct regulation of methyltransferase activity is exemplified by polyamines, which enhance G9a‐catalyzed dimethylation of H3K9me2, thereby reinforcing TSG silencing [417]. Signaling‐mediated site‐specific modification can be influenced by tryptophan metabolites such as indolepropionic acid, which activates the AhR–mixed‐lineage leukemia complex and enriches the trimethylation of H3K4me3 mark in promoters of anti‐tumor genes [418]. Indirect activation of methyltransferase function occurs when SCFAs such as acetate and propionate increase enhancer of zeste homolog 2 (Ezh2) activity, promoting H3K27me3 and enhancing chromatin silencing in undifferentiated stem‐cell states [418]. Modulation of methyl‐donor supply via one‐carbon metabolism factors—for example, folate and vitamin B12—can alter the SAM/SAH ratio, ultimately affecting methylation signaling efficiency [419].

The regulatory mechanisms of histone methylation have been validated in several cancers. For instance, dysfunction of the *KDM6A* plays an important role in tumor initiation, progression, and therapy response. *KDM6A*, which removes the repressive trimethylation mark on H3K27me3, is thought to exert tumor‐suppressive effects [420]. Its functions and underlying mechanisms can be summarized as follows.

*KDM6A* deficiency promotes tumor progression and induces dependency on Ezh2. In esophageal squamous cell carcinoma, *KDM6A* mutations enhance chromatin silencing; targeting the H3K27 methyltransferase Ezh2 in this context increases radiosensitivit. In high‐grade bladder cancer (BLCA), loss of *KDM6A* stimulates tumor progression and enhances sensitivity to Ezh2 inhibitors [421]. Research on immunotherapy has demonstrated that *KDM6A* mutations may promote tumor immune escape, suggesting their potential as biomarkers for predicting response to immune checkpoint therapy [422].

*KDM6A* also takes part in the regulation of several signaling axes. In breast cancer (BrCa), *KDM6A* inhibits malignant progression by targeting the FOXA1‐KDM6A‐ARHGDIB axis. In gastric cancer (GCa), *KDM6A* cooperates with Ezh2 to regulate the SALL4/Wnt/β‐catenin pathway, influencing tumor stemness and drug resistance [423]. In gastrointestinal stromal tumors, *KDM6A* induces *SPARCL1* expression and inhibits the nuclear factor‐κB (NF‐κB) signaling pathway, thereby suppressing tumor proliferation and macrophage recruitment [424].

Combined strategies targeting *KDM6A* show therapeutic potential. In NSCLC, simultaneous inhibition of *KDM6A* and *KMT2B* significantly suppresses cancer stem‐cell proliferation [424]. In cisplatin‐resistant testicular cancer models, inhibiting the *KDM6A*/*KDM6B* axis represents a potential approach to reverse drug resistance [425]. In multiple myeloma, combining Ezh2 inhibitors with daratumumab can overcome resistance by regulating CD38/CD48 expression, a process mediated by *KDM6A* [426].

The mechanism of action of daratumumab involves targeting the transmembrane glycoprotein CD38, thereby mediating complement‐dependent cytotoxicity, antibody‐dependent cell‐mediated cytotoxicity, antibody‐dependent cellular phagocytosis, and directly inducing tumor‐cell apoptosis [426]. It can also eliminate CD38‐positive immunosuppressive cells, enhance cytotoxic T‐lymphocyte activation and IFN‐γ release, and reshape the tumor immune microenvironment. Activation of the downstream *MYC* signaling pathway may lead to metabolic and immune reprogramming, influencing immune efficacy and drug sensitivity. This immunomodulatory capacity can synergize with epigenetic regulators such as Ezh2 inhibitors to enhance anti‐tumor activity [427].

*KDM6A*, a key regulator in the histone demethylation system, exhibits diverse functions across tumor types. Its activity not only determines chromatin accessibility also contributes to stem‐cell maintenance, immune‐response regulation, and therapy‐sensitivity modulation. As an epigenetic modulator, *KDM6A* represents a promising target for future combination immunotherapy strategies.

#### Phosphorylation, ubiquitination, and chromatin remodeling

Histone phosphorylation acts as a rapid‑response switch that can promptly relay extracellular signals; microbial metabolites participate in this regulation. For example, indolepropionic acid can activate pro‐inflammatory genes via the AhR–mitogen‑activated protein kinase (AhR–MAPK) pathway, enhancing H3S10 phosphorylation through MSK1. SCFAs such as butyrate can enhance transcription pause mediated by H3S28 phosphorylation via modulation of PP2A activity [428]. Phosphorylation alterations interact with other modifications and play key roles in DNA damage repair. For example, the interplay between H2BY37ph and H2BK120ub upregulates the DDR [429], whereas ATM‐dependent phosphorylation of Bub3 helps maintain genomic integrity [430]. These dynamic protein modifications depend critically on the actions of specific enzymes [431, 432].

H2AK119ub and H2BK120ub, two major types of histone ubiquitination, are critical for gene silencing and transcriptional elongation. Microbially derived conjugated linoleic acid upregulates H2BK120ub via the PPARγ–RNF20 axis, mediating transcription of DDR‐related genes [433]. Excess polyamines can inhibit the deubiquitinating enzyme USP22, leading to accumulation of H2AK119ub at TSG promoters and maintaining gene silencing. In addition, the regulatory network of E3 ubiquitin ligases provides new targets for cancer treatment. For instance, RNF126 can modulate DNA damage response‐related radiosensitivity in triple‐negative breast cancer (TNBC) by regulating MRE11; inhibiting the cyclin‐dependent kinase 5 can promote PD‐L1 degradation via FBXO22, thereby enhancing immunotherapy synergy [434]; the deubiquitinating enzyme OTUD6A stabilizes TopBP1, contributing to therapy resistance in BrCa [435].

SUMOylation is equally crucial. SENP1 promotes DDR and chemotherapy resistance by de‐SUMOylating RNF168 [436]. Inhibition of the SUMOylation pathway affects the fate of prostate cancer (PCa) cells in *RINT1*‐deficient [437] and *KRAS*‐mutated [438] contexts, respectively, offering potential for novel combination therapy development.

### ncRNA regulatory layer

ncRNAs, including microRNAs (miRNAs), lncRNAs, and circular RNAs (circRNAs), play crucial roles in tumor epigenetic regulation. Their expression is controlled by DNA methylation and histone modifications, and they widely affect tumor initiation and progression by interacting with transcription factors, RNA‐binding proteins (RBPs), and target messenger RNAs (mRNAs) (Figure 9). For instance, the microbial metabolite butyrate can inhibit HDACs, thereby altering histone acetylation at promoters of long ncRNAs such as *HOTAIR*, subsequently regulating their expression and influencing downstream target genes [439]. These findings suggest that microbial metabolites can act as upstream regulators of ncRNA expression and epigenetic states, thereby linking microbiota‐derived metabolic signals to tumor‐cell phenotypes. Therefore, microbial metabolites can significantly reshape the tumor gene‐expression network through an “epigenetic modification–ncRNA–target gene” cascade, contributing to tumorigenesis and progression.

**Figure 9:**
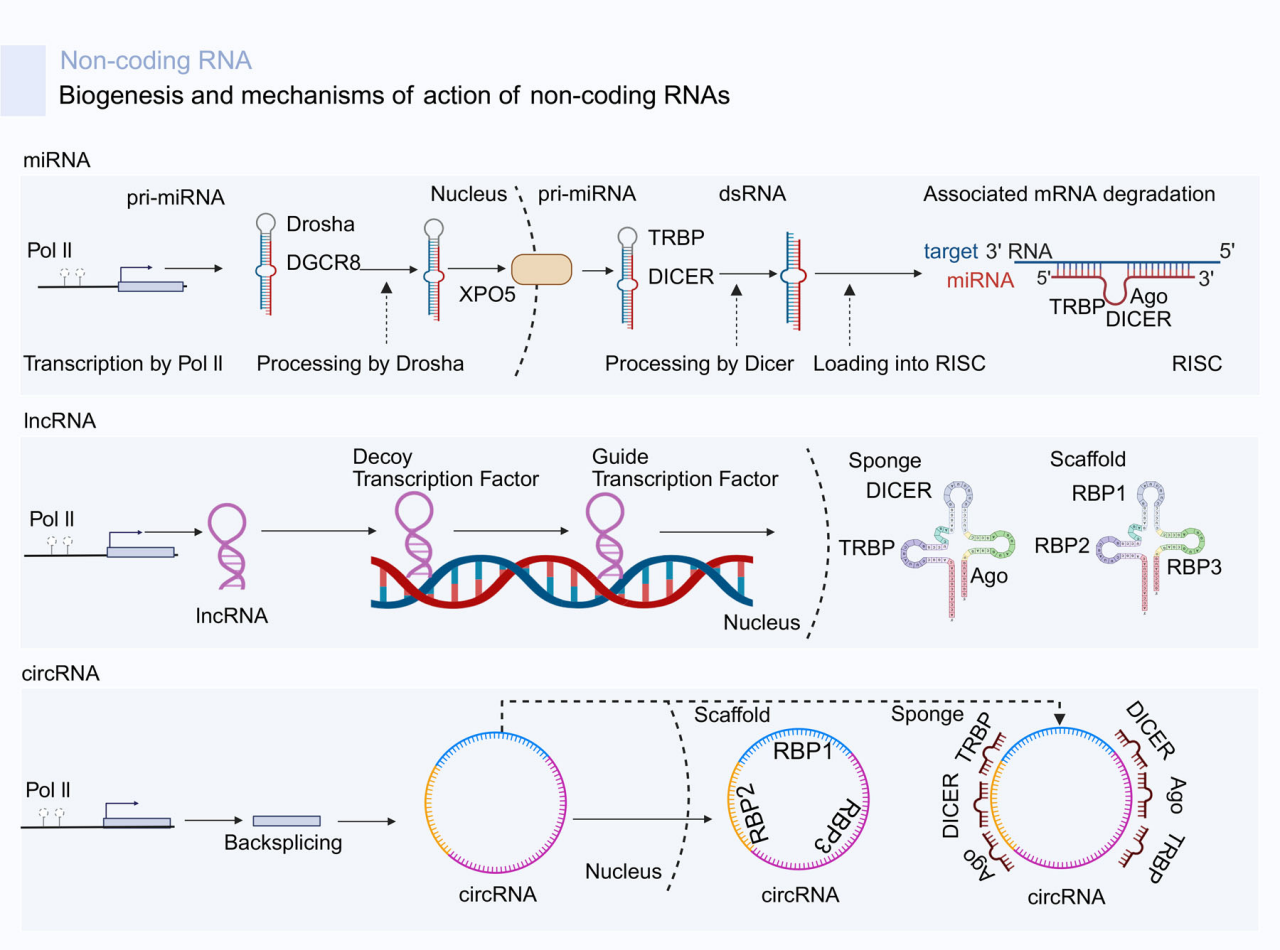
Biogenesis of ncRNAs and their mechanisms of action. miRNAs are transcribed by RNA polymerase II and subsequently processed by the Drosha–DGCR8 and Dicer–TRBP complexes. These miRNAs are then incorporated into the RISC, where they play a crucial role in regulating mRNA degradation and inhibiting translation. lncRNAs serve as decoys or guides for transcription factors, or act as scaffolds and sponges that interact with RBPs to modulate gene expression. circRNAs, which are generated through back‐splicing, function as sponges for miRNAs or RBPs and serve as scaffolds that regulate both transcriptional and post‐transcriptional processes. These ncRNAs play critical regulatory roles in tumor epigenetics and cellular homeostasis. Ago, Argonaute protein; circRNA(s), circular RNA(s); DGCR8, DiGeorge syndrome critical region 8; dsRNA, double‐stranded RNA; lncRNA(s), long non‐coding RNA(s); miRNA(s), microRNA(s); Pol II, RNA polymerase II; RBP(s), RNA‐binding protein(s); RISC, RNA‐induced silencing complex; TRBP, TAR RNA‐binding protein; XPO5, exportin 5.

#### miRNA

miRNAs are short ncRNAs that mainly regulate key biological processes—including cell proliferation, differentiation, migration, and apoptosis—by promoting target mRNA degradation or inhibiting its translation. Across various tumor types, downregulation of tumor‐suppressive miRNAs is often associated with promoter DNA hypermethylation. This epigenetic dysregulation mechanism is highly conserved among tumors of different tissue origins, providing a common basis for tumor progression and immune escape. Although many of these mechanisms are intrinsic to tumor cells, growing evidence indicates that microbial metabolites, particularly SCFAs, can modulate DNA methylation machinery, thereby indirectly shaping miRNA expression profiles in multiple cancers.

In myeloid malignancies, mechanistic studies show that IRAK1‐dependent inflammatory signaling, integrating NF‐κB pathways and epigenetic programs, is required to maintain leukemic stem/progenitor cell function and disease progression [440]. Similarly, in breast and PDACs, silencing of miRNAs such as *MIR145*, *MIR335*, and *MIR124* affects EMT and metastasis‐related signaling pathways [441, 442]. These tumor‐intrinsic observations highlight a conserved epigenetic vulnerability of miRNAs that may, in theory, be modulated by microbially derived metabolites targeting methyltransferases or histone‐modifying enzymes.

Specific miRNA‐target pathways operate across tumor types. For instance, in oral squamous cell carcinoma, hypermethylation of the *MIR34B/C* promoter is significantly associated with poor prognosis in high‐risk human papillomavirus (HPV)‐negative patients [443]. In nasopharyngeal carcinoma, hypermethylation of *MIR9‐1* can affect the Warburg metabolic pathway [444]. In osteosarcoma and lung adenocarcinoma (LUAD), DNA methylation regulates the expression of *MIR486‐5p* and *MIR148A‐3p*, thereby affecting cell migration and metabolic regulation [445, 446]. The highly conserved *MIR125A* is silenced in various solid tumors such as BrCa, liver cancer, and lung cancer (LuCa) due to promoter methylation. Restoring its expression can significantly inhibit autophagy and invasion, providing a new therapeutic target across multiple cancers [447]. Collectively, these findings illustrate how miRNA expression is tightly linked to epigenetic enzyme activity—activity that can be modulated by microbial metabolites such as butyrate, propionate, and acetate.

Overall, miRNA promoter methylation is a common epigenetic aberration across various cancers, functioning as a key node in regulating cell fate and tumor progression. Although different tumors may involve specific targets and signaling pathways, the strategy of silencing miRNA via DNA methylation to promote tumorigenesis or immune escape is highly universal and conserved. This mechanism provides a theoretical basis and rationale for the broad‐spectrum development of miRNA methylation‐targeted therapies. As short ncRNAs, miRNAs constitute an important layer of tumor epigenetic regulation. Their expression imbalance exhibits considerable tissue specificity across cancers: some miRNAs lose tumor‑suppressive functions due to promoter methylation or histone modification, whereas others exert oncogenic properties through aberrant upregulation. This “double‐edged sword” effect is evident in nearly all major cancer types (Figure 10). Given the capacity of microbial metabolites to modulate methylation and histone acetylation machinery, they may indirectly shape these tumor‐specific miRNA expression profiles, thereby providing mechanistic links between host‐microbiota metabolism and ncRNA biology.

**Figure 10:**
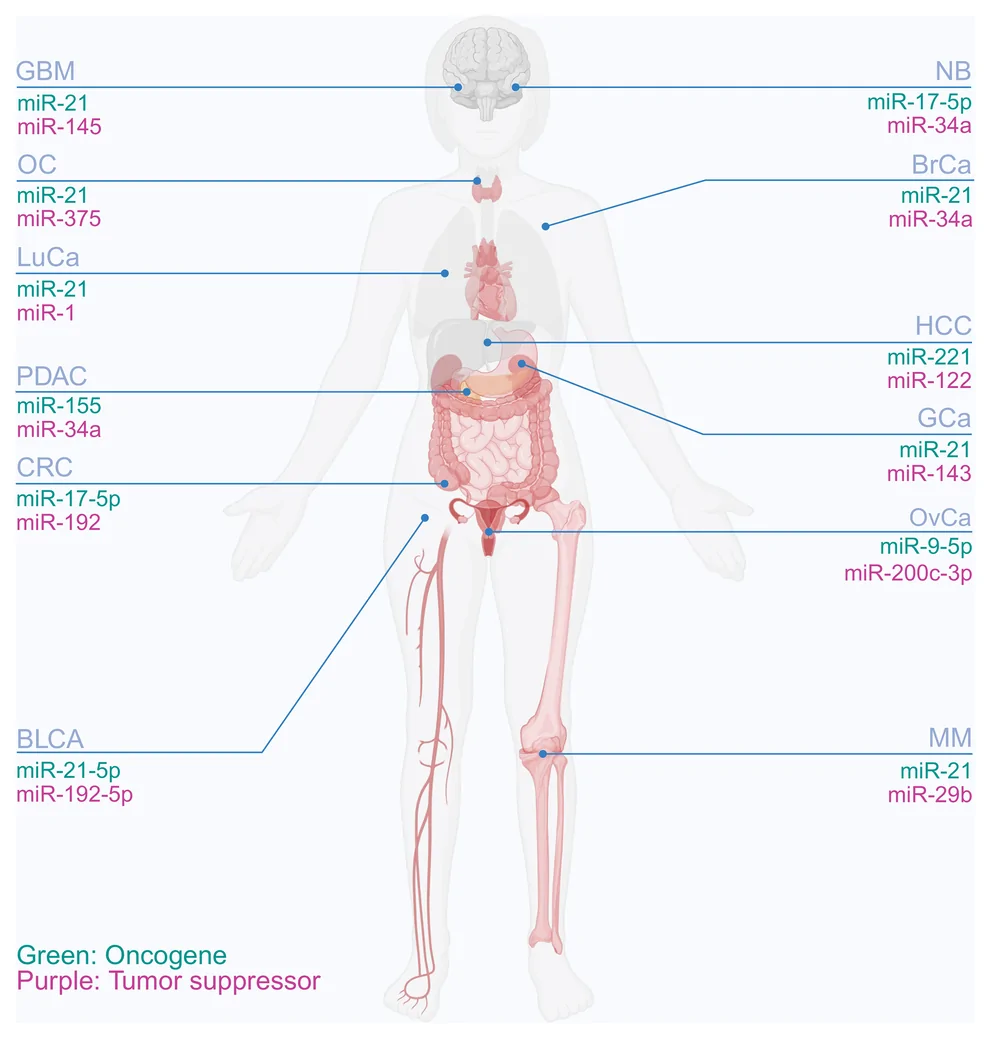
Representative oncogenic and tumor suppressor miRNAs implicated in different cancer types and their tissue distribution. miRNAs exhibit distinct oncogenic or tumor‐suppressive functions across human cancers depending on tissue context. Oncogenic miRNAs, such as miR‐21, miR‐17‐5p, miR‐155 and miR‐221, are frequently upregulated in various cancers, including GBM, lung, pancreatic, colorectal, hepatocellular, and BrCa, where they promote proliferation, survival, and metastasis. In contrast, tumor suppressor miRNAs, such as miR‐145, miR‐34a, miR‐192, miR‐128, and miR‐29b, are often reported to be silenced in glioma, BrCa, CRC, and MM, which impairs cell cycle control and apoptosis. GBM, a high‐grade subtype of glioma, is displayed separately to distinguish miRNA dysregulation associated with highly aggressive gliomas from that observed in gliomas more broadly. This spatial mapping highlights the dual roles of miRNAs in cancer development and emphasizes their potential as diagnostic and therapeutic targets. Color coding distinguishes molecular types and functional axes: Green indicates oncogenic miRNAs, whereas purple denotes tumor suppressor miRNAs. BrCa, breast cancer; BLCA, bladder cancer; CRC, colorectal cancer; GBM, glioblastoma; GCa, gastric cancer; HCC, hepatocellular carcinoma; LuCa, lung cancer; MM, multiple myeloma; NB, Neuroblastoma; OC, oral cancer; OvCa, ovarian cancer; PDAC, pancreatic cancer.

#### lncRNA

lncRNAs regulate gene expression through multiple mechanisms, including recruiting chromatin‐modifying complexes, functioning as molecular scaffolds, and acting as competing endogenous RNAs. Across diverse tumor types, lncRNA expression profiles are frequently dysregulated. Their core functions are usually mediated by epigenetic mechanisms, broadly influencing tumor initiation, progression, and therapy resistance. Since microbial metabolites can alter the activity of chromatin‐modifying enzymes, their effects may extend to lncRNA promoter accessibility and expression regulation, thereby adding a microbiota‐associated layer to lncRNA‐mediated oncogenic pathways. Table 11 summarizes key lncRNAs implicated in tumor epigenetic regulation. While these mechanisms have been largely characterized within tumor biology, the sensitivity of lncRNA promoters to epigenetic modification suggests that they represent critical downstream targets for microbiota‐derived metabolic signals.

**Table 11:** Key lncRNAs involved in tumor progression: Potential epigenetic targets for microbial metabolite regulation.

Cancer type	Oncogene	Oncogenic mechanism	Tumor suppressor	Tumor suppressor mechanism	References
BrCa	HOTAIR	PRC2‐mediated epigenetic silencing of TSGs	miR‐34c‐5p	Inhibits E2F1 expression	[448, 449]
NSCLC	MALAT1, NORAD	Regulate E2F1 to promote cell cycle progression	miR‐185‐5p	Inhibits MDM4 expression	[450, 451]
GCa	SNHG15	Targeted inhibition of miR‐506‐5p	miR‐506‐5p	Targeted inhibition by SNHG15	[452]
TCa	MFSD4A‐AS1	Regulates the VEGF/TGF‐β signaling pathway via miRNA sponging	miR‐30c‐2‐3p	Targeted inhibition of VEGF/TGFBR2	[453]
PDAC	LINC00657	Sponges miR‐520h and upregulates CKS1B	miR‐520h	Targeted inhibition of CKS1B expression	[454]
OvCa	NEAT1	Sponges miR‐34a‐5p and upregulates BCL2	miR‐34a‐5p	Targeted inhibition of BCL2 expression	[455]
Glioma	NEAT1	Sponges miR‐98‐5p, thereby upregulating BZW1 expression	miR‐98‐5p	Directly targets and inhibits BZW1 expression	[456]
Cervical cancer	AFAP1‐AS1	Upregulates EGFR expression by competitively binding miR‐7‐5p, promoting drug resistance	miR‐7‐5p	Directly targets and inhibits EGFR gene expression	[457]
ESCA	LINC00152	Sponges miR‐107 to upregulate RAB10	miR‐107	Targets Rab10 to inhibit tumor progression	[458]
TNBC	LUCAT1	Promotes proliferation and metastasis while inhibiting apoptosis	miR‐5702	Negatively regulates LUCAT1 to achieve anti‐cancer effects	[459]
Adult T‐cell leukemia (ATL)	ANRIL	Forms a complex with EZH2 and p65 to activate NF‐κB signaling	miR‐455‐3p	Inhibits the expression of oncogenes AK4 and RHOC	[460, 461]
Melanoma	BANCR	Promotes cell proliferation	miR‐218‐5p	Directly targets and inhibits the expression of the oncogene STAM2	[462, 463]
BLCA	UCA1	Sponges miR‐143 to upregulate HMGB1 and promote EMT	miR‐143	Targets HMGB1 to inhibit invasion and EMT	[464]
Endometrial cancer (ECa)	TUG1	Inhibits miR‐299 and miR‐34a‐5p	miR‐299/miR‐34a‐5p	Inactivated by inhibition from TUG1	[465]
MM	MALAT1	Forms a positive feedback loop with NRF1 to inhibit KEAP1 transcriptional activity	miR‐485‐5p	Directly targets and inhibits the expression of ABCB8	[466, 467]
Chordoma	lncRNA NONHSAT02477	Acts as a ceRNA to sponge miR‐1290 and upregulate Robo1	miR‐1290	Directly targets and inhibits the expression of the oncogene Robo1	[468]
GBM	LPP‐AS2	Acts as a ceRNA to sponge miR‐7‐5p and upregulate EGFR and c‐MYC	miR‐7‐5p	Directly targets and inhibits the expression of EGFR	[469]
HCC	LINC01234	Downregulates ASS1 expression and activates the mTOR signaling pathway	miR‐524‐5p	Targets and inhibits the expression of TUG1 and SIX1, suppressing glycolysis and invasion	[470, 471]
Cholangiocarcinoma	HOTTIP	Sponges miR‐637 to relieve its inhibitory effect on LASP1	miR‐637	Targets and downregulates LASP1, enhancing chemotherapy sensitivity	[472]
LuCa	LINC01133	Sponges miR‐30d‐5p to relieve its inhibitory effect on MARCKS	miR‐30d‐5p	Targets MARCKS, suppresses proliferation, and promotes apoptosis	[473]

Abbreviations: ATL, adult T‐cell leukemia; ECa, endometrial cancer.

On one hand, numerous lncRNAs influence signaling and transcriptional regulation by regulating chromatin modification states. For instance, *PRADX* recruits Ezh2 to activate the NF‐κB signaling pathway, promoting tumor progression in GBM and CRC [474]; *MALAT1* co‐localizes with polycomb repressive complex 2 (PRC2) at promoters of active genes to mediate transcriptional regulation in BrCa [475]; and *HOTAIR* enhances stem‑cell‑like properties and EMT in BrCa, PDAC, and LuCa by regulating the NF‐κB axis or inhibiting *MIR145‐5p* [476, 477].

On the other hand, lncRNAs play a key role in cellular metabolic reprogramming by influencing methylation patterns. For instance, *SNHG22* promotes hepatocellular carcinoma (HCC) cell proliferation by regulating the methylation level of the *MIR16‑5p* promoter [478]; similarly, *RBM5‐AS1* recruits PRC2 to inhibit *MIR132/212* expression, thereby facilitating cell‑cycle progression in LuCa [479]. In addition, *PRADX* inhibits *BLCAP* via H3K27me3, activates the signal transducer and activator of transcription 3 (STAT3) pathway, and accelerates metabolic reprogramming across multiple tumor types [480]. Because microbial metabolites such as SCFAs directly modulate histone modification states, they may influence the activity of these chromatin‐associated lncRNAs, thereby integrating microbiota‑derived metabolic signals into lncRNA‐driven oncogenic pathways.

Moreover, lncRNAs frequently exert anti‐tumor effects via epigenetic mechanisms, particularly through the regulation of transcriptional programs governing EMT and metastatic dissemination. For example, methylation‐mediated downregulation of *MIR200CHG* attenuates its inhibitory control over EMT and thereby promotes metastasis in GCa [481]. This highlights another potential avenue through which metabolite‐induced epigenetic remodeling could reactivate tumor‐suppressive lncRNAs to counteract cancer progression.

In summary, the paradigm of lncRNA‑mediated gene regulation through epigenetic mechanisms has been consistently validated across diverse tumor types. Although specific regulatory networks differ among cancers, lncRNAs have emerged as core regulatory molecules in multiple oncogenic pathways. By mediating chromatin conformation, recruiting enzyme complexes, regulating miRNA expression, and participating in metabolic reprogramming, lncRNAs provide key theoretical support for the future development of broad‐spectrum targeted therapies. Considering the established epigenetic activity of microbial metabolites, microbiota‐derived molecules may act as modulators of lncRNA expression and function, thereby bridging tumor epigenetics with host‐microbiota metabolic crosstalk.

#### circRNA

circRNAs are a class of structurally stable, non‐degradable ncRNAs widely present in eukaryotic cells. Their roles in tumors are mainly realized through three mechanisms: acting as miRNA sponges, regulating RBP activity, or participating in the recruitment of epigenetic enzyme complexes, thereby functioning as key nodes in gene‐expression regulation. Growing evidence indicates that circRNA expression and function are regulated by epigenetic mechanisms—including DNA methylation and RNA modifications—and play important roles across diverse tumor types. Based on their functional characteristics, circRNAs can be categorized as follows (Table 12 for a summary of key circRNAs involved in tumor progression). While direct evidence linking specific microbial strains to individual circRNAs is still emerging, circRNA biogenesis is tightly controlled by splicing factors and epigenetic marks that are known targets of microbial metabolites, suggesting they represent potential downstream effectors in the microbe‐cancer axis.

**Table 12:** Key circRNAs in tumor progression: Potential downstream targets for microbial epigenetic modulation.

Cancer type	Oncogene	Oncogenic mechanism	Tumor suppressor	Tumor suppressor mechanism	References
ICC	circPCNXL2	Binds STRAP to activate the ERK/MAPK pathway; sponges miR‐766‐3p, upregulating SRSF1	circUGP2	Activates the ADGRB1/p53 axis by sponging miR‐3191‐5p	[482, 483]
NSCLC	hsa_circ_0125356	Sponges miR‐582‐5p, upregulating FGF9 and activating the Wnt pathway, leading to gemcitabine resistance	miR‐582‐5p	Targets FGF9, inhibits the Wnt signaling pathway, and enhances chemotherapy sensitivity	[484]
NSCLC	hsa_circ_102179	Sponges miR‐330‐5p, upregulating HMGB3 expression	miR‐330‐5p	Targets HMGB3, suppresses proliferation and invasion	[485]
NSCLC	circVMP1	Sponges miR‐524‐5p, upregulating METTL3/SOX2	miR‐524‐5p	Targets METTL3/SOX2, suppresses progression and resistance	[486]
NSCLC	circHERC1	Sponges miR‐142‐3p, upregulating HMGB1	miR‐142‐3p	Targets and inhibits HMGB1 gene expression	[487]
NSCLC	circPPAPDC1A	Sponges miR‐30a‐3p, activating the IGF1R/PI3K/AKT/mTOR pathway	miR‐30a‐3p	Targeted inhibition of IGF1R expression	[488]
NSCLC	circMBOAT2	Mediates chronic stress to promote tumor cell proliferation, migration, and invasion	CTCF	Negatively regulates the expression of oncogenic circular RNA (circRNA) circMBOAT2	[489]
NSCLC	hsa_circ_0001666	Sponges miR‐1184/miR‐548I, upregulating AGO1 and activating the PI3K/AKT pathway	miR‐1184, miR‐548I	Targets AGO1 and inhibits the PI3K/AKT/mTOR signaling pathway	[490]
NSCLC	hsa_circ_0022383	Sponges miR‐495‐3p, enhancing KPNA2 expression	miR‐495‐3p	Targeting KPNA2 to inhibit tumor progression	[491]
NSCLC	circATXN7	Sponges miR‐7‐5p, upregulating PFN2 expression	miR‐7‐5p	Targeting PFN2 to inhibit tumor malignancy	[492]
NSCLC	hsa_circ_0017620	Sponges miR‐520a‐5p, upregulating KRT5 expression	miR‐520a‐5p	Targeting KRT5 to inhibit proliferation and metastasis	[493]
GBM	circMMD	Sponges miR‐15b‐5p, activating the Wnt/β‐catenin pathway	miR‐15b‐5p	Targeting and degrading FZD6 to suppress the Wnt signaling pathway	[494]
GBM	circFOXO3	Sponges miR‐138‐5p/miR‐432‐5p, upregulating NFAT5 and promoting tumor activity	miR‐138‐5p/432‐5p	Targeting NFAT5 to inhibit proliferation and invasion	[495]
Glioma	circPCMTD1	Sponges miR‐224‐5p, promoting tumor progression	miR‐224‐5p	Targeting mTOR to inhibit its translation	[496]
GBM	circPOLR2A	Sponges miR‐2113, upregulating POU3F2 and activating SOX9 transcription	miR‐2113	Targeting POU3F2 to inhibit downstream oncogenic signals	[497]
GBM	circFGFR1	Sponges miR‐224‐5p, upregulating CXCR4 expression	miR‐224‐5p	Suppresses tumor malignancy by inhibiting CXCR4 expression	[498]
GBM	circXPO1	Sponges miR‐7‐5p, promoting malignancy	miR‐7‐5p	Inhibiting tumor progression through negative regulation of RAF1	[499]
GBM	circFOXM1	As a miR‐577 molecular sponge, it relieves the inhibition on E2F5	miR‐577	Targeting and inhibiting the expression of E2F5	[500]
HCC	hsa_circ_0001955	Promotes the development of HCC through sponge‐mediated release of TRAF6 and MAPK11 from miR‐516a‐5p	miR‐516a‐5p	Directly targeting and inhibiting the expression of TRAF6 and MAPK11	[501]
HCC	circSEC. 24 A	As a miR‐421 molecular sponge, it relieves the inhibition on MMP3	miR‐421	Directly targeting and inhibiting the expression of MMP3	[502]
HCC	circPIAS1	As a miR‐455‐3p molecular sponge, it upregulates the NUPR1/FTH1 axis and inhibits ferroptosis	miR‐455‐3p	Targeting and inhibiting the expression of NUPR1	[503]
AML	circPVT1	As a miR‐455‐3p molecular sponge, it promotes the expression of MCL1	miR‐455‐3p	Targeting and inhibiting the expression of the MCL1 gene	[504]
T‐cell acute lymphoblastic leukemia	circPRKDC	Sponges miR‐653‐5p, relieving its inhibition of RELN	miR‐653‐5p	Targeting and inhibiting RELN and the PI3K/AKT/mTOR pathway	[505]
Chronic myeloid leukemia	circCRKL	Sponges miR‐877‐5p, upregulating BCR‐ABL expression	miR‐877‐5p	Targeted inhibition of BCR‐ABL gene expression	[506]
BrCa brain metastasis	circBCBM1	Sponges miR‐125a, upregulating BRD4 and MMP9 expression	miR‐125a	Targeted inhibition of BRD4 gene expression	[507]
CRC	hsa_circ_002144	Sponges miR‐615‐5p, relieving its inhibition of LARP1	circ_0007379	Acts as a scaffold to promote miR‐320a maturation, inhibiting RUNX1 expression	[508, 509]
Cervical cancer	circCDK6	Sponges miR‐449a, inhibiting its activity	circMAST1	Competitively binds to NAT10, inhibiting the ac4C modification of YAP mRNA and promoting its degradation	[510, 511]
KCa	circNUP98	Sponges miR‐567, relieving its inhibition of PRDX3	circAMOTL1L	Sponges miR‐92a‐2‐5p, relieving its inhibition of the TSG KLLN	[512, 513]
Cervical cancer	hsa_circ_0000119	Sponges miR‐433‐3p, relieving its inhibition of the oncogenic target PAK2	miR‐433‐3p	Directly targets and inhibits the expression of the oncogene PAK2	[514]
CRC	hsa_circ_0000231	Sponges miR‐502‐5p, upregulating MYO6 expression	miR‐502‐5p	Targets and inhibits the expression of MYO6 mRNA	[515]
CRC	hsa_circ_0126925	Inhibits TRIM21‐mediated ubiquitination and degradation of BCAT2, maintaining BCAA metabolism	TRIM21	Mediates the ubiquitination and degradation of BCAT2, limiting tumor growth	[516]
CRC	hsa_circ_0007718	Promotes CRC progression by modulating the miR‐1299/PSMC2 axis	miR‐1299	Targeted inhibition of PSMC2 mRNA expression	[517]
CRC	hsa_circ_0001313	Sponges miR‐338‐3p, inducing radioresistance	miR‐338‐3p	Negatively regulates circ_0001313, enhancing radiosensitivity	[518]
CRC	hsa_circ_0079480	Enhances cell proliferation, migration, and invasion by the miR‐498/ATP5E axis	miR‐498	Targeted inhibition of ATP5E mRNA expression	[519]
Cervical cancer	hsa_circ_0000118	Sponges miR‐211‐5p/miR‐377‐3p, maintaining AKT2 levels	miR‐211‐5p, miR‐377‐3p	Negatively regulate the expression of the AKT2 gene	[520]
CRC	hsa_circ_0000520	Sponges miR‐542‐3p, upregulating MYH9 expression to promote cancer	miR‐542‐3p	Targets and inhibits MYH9 mRNA expression	[521]
CRC	hsa_circ_0008234	Promotes CRC progression by modulating the miR‐338‐3p/ETS1 axis and the PI3K/AKT/mTOR signaling pathway	miR‐338‐3p	Targeted inhibition of ETS1 expression	[522]
CRC	hsa_circ_0128846	Promotes CRC tumorigenesis by sponging hsa‐miR‐1184, releasing AJUBA, and inactivating the Hippo/YAP signaling pathway	miR‐1184	Targeting AJUBA to activate the Hippo/YAP pathway	[523]
Cervical cancer	hsa_circ_400029	Sponges miR‐1285‐3p, relieving its inhibition of TLN1	miR‐1285‐3p	Directly targets TLN1 mRNA and inhibits its expression	[524]
CRC	hsa_circ_013666	Activates Treg‐mediated immune escape in CRC via the miR‐497/PD‐L1 pathway	miR‐497	Directly targets the 3′‐UTR of PD‐L1, inhibiting its expression	[525]

Abbreviation: circRNA, circular RNA.

In BLCA, for example, the demethylation of *circ_104797* enhances cisplatin sensitivity, illustrating its potential as an epigenetic intervention target [526]. In HCC, for instance, *circ‐CCT3*—regulated by N⁶‑methyladenosine (m⁶A) modification—promotes tumor progression via the *MIR378A‐3p‐FLT1* axis [527]. In GCa and BrCa, *circ‐RAB31* and c*ir‐BACH2* act as sponges for *MIR885‐5p* and *hsa‐miR‐944*, respectively, activating the PI3K/AKT and MAPK signaling pathways [528, 529]. circRNAs also take part in various cancer‐associated processes, including tumorigenesis and inflammation. For example, *hsa_circ_0000043* promotes malignant phenotypes by sequestering *MIR4492* in LuCa [530], while *circNTNG1* sponges MIR19B‐3p in clear‐cell renal cell carcinoma, thereby inhibiting EMT and suppressing cell migration [531]. circRNAs can also exert direct tumor‑suppressive effects, as illustrated in CRC where *circ_0003266* functions through the *MIR503‐5p‐PDCD4* axis, highlighting its potential as a new therapeutic target [532]. Given that circRNA biogenesis and function are strongly influenced by epigenetic marks—which microbial metabolites are known to modify—microbiota‐derived metabolic signals may shape circRNA‐mediated regulation in tumor biology.

In conclusion, circRNAs contribute to essential pathological processes across different tumor types by regulating miRNA networks, signaling pathways, and epigenetic modification. Their regulatory modes are highly conserved yet functionally diverse among cancers. circRNAs are not only promising as broad‐spectrum biomarkers but also offer a rich source of potential targets for future RNA‑based therapeutics and epigenetic intervention strategies. Importantly, the ability of microbial metabolites to alter chromatin modifications and RNA regulatory pathways suggests potential crosstalk between microbiota‐derived metabolic networks and circRNA biology, a connection that merits further investigation.

#### RBPs

RBPs represent a critical post‐transcriptional regulatory layer that is highly sensitive to cellular metabolic and epigenetic states. Microbial metabolites play a dual role in tumor epigenetic regulation by directly modulating epigenetic enzyme activity and reshaping cellular metabolic signaling pathways that broadly influence gene regulatory programs and their downstream regulatory layers [326]. Recent studies show that metabolite mixtures derived from *Candida albicans* disrupt energy metabolism and mitochondrial homeostasis in CRC cells, inhibiting proliferation, migration, and invasion in dose‑ and time‐dependent manners while inducing lipid peroxidation and cell death. These findings suggest that fungal metabolites, as complex and bidirectional regulators, participate in the interplay between tumor metabolism and epigenetics [533, 534].

Meanwhile, SCFAs—key products of intestinal microbial digestion of dietary fiber—act as classic HDACi. They can modify histone acetylation and chromatin conformation, and influence gene transcription and epigenetic status by modulating RBP translation and stability. SCFAs also serve as acyl donors for PTMs during chromatin remodeling. Furthermore, they interact with receptor‐mediated signaling and energy‐metabolism pathways, thereby integrating the dynamic crosstalk between the microbiota and host chromatin. Collectively, these processes reshape the epigenetic and transcriptional landscape in tumor cells via SCFAs [535, 536].

Recent studies suggest that microbial metabolites modulate ncRNA function by altering RBP activity, thereby establishing a post‑transcriptional regulatory layer [537]. NaB, for instance, enhances H3K27 acetylation, upregulates *LOH12CR2* expression, stabilizes the m^6^A methyltransferase METTL14, stimulates m^6^A modification of *SPC24* mRNA leading to its degradation, and thereby suppresses CRC initiation and progression [538]. Moreover, metabolites such as vitamin B12 can influence RNA stability and translational efficiency by affecting RBP recognition of m^6^A marks. Microbial factors—including SCFAs and vitamin B12—coordinate multi‐level epigenetic and post‐transcriptional networks that regulate RBP–ncRNA interactions, which in turn modulate tumor‑cell proliferation, apoptosis, and differentiation.

In addition to the regulation by specific metabolites, RBPs themselves occupy central nodes within the tumor epigenetic network. RBPs critically influence gene expression and play essential roles in cancer cell epigenetics. Members of the insulin‐like growth factor 2 mRNA‑binding protein (IGF2BP) family function as m^6^A readers and exhibit strong oncogenic activity, thereby contributing to m^6^A‐driven tumorigenesis and progression [539]. RBPs regulate gene expression through post‐transcriptional mechanisms that interface with various epigenetic alterations such as DNA methylation, histone modifications, and ncRNA‑mediated regulation [540]. Certain RBPs can participate in DNMT‑mediated chemical modifications at gene promoters. For example, HNRNPK recruits DNMT1 to methylate the *TRAF3IP2* promoter, repressing transcription and disrupting the equilibrium between TSGs and oncogenes, thereby promoting tumorigenesis [541]. Furthermore, RBPs can form functional complexes with epigenetic enzymes such as HDACs to regulate chromatin architecture and transcription. For instance, SUMOylated HDA‐1, an HDAC homolog, enhances its interaction with chromatin regulators such as MEP‐1 and other remodeling factors, promoting deacetylation and chromatin compaction to silence transcription through epigenetic control [542]. Collectively, these findings demonstrate that RBPs engage in complex interactions with multiple epigenetic modifiers, forming integrated transcriptional networks that confer dynamic plasticity to tumor‑cell gene‐expression programs.

Members of the RBP family perform diverse functions, many of which serve as key regulators of tumorigenesis and progression. Heterogeneous nuclear ribonucleoproteins (HNRNPs) are a major subclass of RBPs involved in RNA splicing, transport, translation, and degradation. Substantial evidence links HNRNPs to cancer initiation and therapy resistance [541]. During adaptive evolution, tumor cells dynamically reshape their epigenetic landscape—including DNA methylation and ncRNA function—through RBP‑mediated regulation, which in turn modulates cell proliferation, migration, and drug sensitivity. Furthermore, cooperation between RBPs and lncRNAs enables dynamic gene‐expression manipulation, remodels molecular functional profiles, and offers new avenues for studying tumor heterogeneity [543]. Such epigenetic reprogramming improves tumor‑cell survival and adaptability while providing novel mechanistic insights into therapy resistance, underscoring the multifaceted role of RBPs in malignant progression.

### Chromatin 3D structure remodeling

Structural studies of the 3D chromatin structure, particularly topologically associating domains (TADs), provide a spatial framework for understanding gene‑expression regulation. The CCCTC‑binding factor (CTCF) and cohesin complexes maintain TAD integrity by forming chromatin loops that bring enhancers and promoters into proximity, thereby activating or suppressing gene expression. In various tumors, deformation or disruption of TAD boundaries reorganizes chromatin looping, resulting in aberrant enhancer–promoter contacts that can inappropriately activate oncogenes or silence TSGs. This remodeling drives tumorigenesis, transcriptional reprogramming, and therapy resistance [544].

Altered chromatin topology plays a key role in regulating therapy sensitivity. PDAC cells utilize interactive enhancer hubs to form highly dynamic chromatin loops, enabling them to evade drug pressure. Research shows that targeting these enhancer structures can reverse chemotherapy resistance [545]. The unusual plasticity of chromatin configuration equips cancer cells with “adaptive survival strategies,” and inhibiting this plasticity may provide a critical approach to restore therapeutic response.

In PCa, the *LTF* enhancer RNA (eRNA) promotes chromatin loop formation at its target gene *LTF*, sustaining *LTF* expression and exerting tumor‐suppressive effects. Downregulation of this eRNA weakens *LTF* activation, thereby attenuating its restraint on tumor progression [546]. Likewise, in PCa, the transcription factor *MYC* disturbs CTCF‐mediated chromatin looping, resulting in aberrant oncogene activation and disruption of transcriptional homeostasis [547]. In hormone‑dependent tumors, *large tumor suppressor kinase 1 (LATS1)* regulates the selection of CTCF binding sites on chromatin, thereby shaping the gene‐expression program [548]. Collectively, transcription factors, eRNAs, and regulatory factors govern key oncogenic drivers through the regulation of chromatin folding, ultimately altering tumor phenotype.

Enhancer and super‐enhancer (SE) connectivity is crucial for signaling‑pathway regulation. Promoter‐enhancer remodeling activates subtype‐specific genes, sustaining signaling‑pathway expression in TNBC [549]. In LUAD, transforming growth factor‑β (TGF‑β) signaling activates the SE associated with *LINC01977*, promoting tumor invasiveness [550]. Another example of SE hijacking involves *RBBP7* activation, which recruits the nucleosome remodeling and deacetylase (NuRD) complex to enhance stemness and metastasis in BrCa cells [551]. The SE‐regulated long intergenic ncRNA *LINC00880* functions through the CDK1/peroxiredoxin 1 (PRDX1) axis to sustain malignant expression in LUAD [552]. SEs function as amplified hubs—core chromatin‐structural elements that spatially integrate multiple oncogenic signaling pathways.

The stability of chromatin 3D structure and associated genome‑defense mechanisms help prevent cancer in vertebrates. In normal cells, CTCF maintains the integrity of TAD borders and chromatin loops. Loss or mutation of CTCF impairs loop formation, leading to aberrant oncogene activation [553]. Conversely, tumors overexpressing *CTCFL*, a CTCF homolog, exhibit increased genomic instability and mutation risk. CTCF binding to HDACs is essential for chromatin compaction, which restricts nonspecific gene activation [554]. Under the influence of SEs, broad domains marked by H3K4me3 can be converted from tumor‑suppressive to oncogenic regions, highlighting SEs' vital role in chromatin plasticity and transcriptional reprogramming [555]. Thus, the 3D chromatin architecture not only organizes genomic space but also safeguards the genome and maintains cellular homeostasis through tumor‑suppressive pathways. Disruption of this spatial stability can be viewed as an “epigenetic mutation in the spatial dimension.”

In multiple tumor types, alteration or disruption of TAD boundaries causes aberrant enhancer‐promoter contacts. These ectopic interactions can activate oncogenes or silence TSGs, driving tumorigenesis and transcriptional reprogramming (Figure 11).

**Figure 11:**
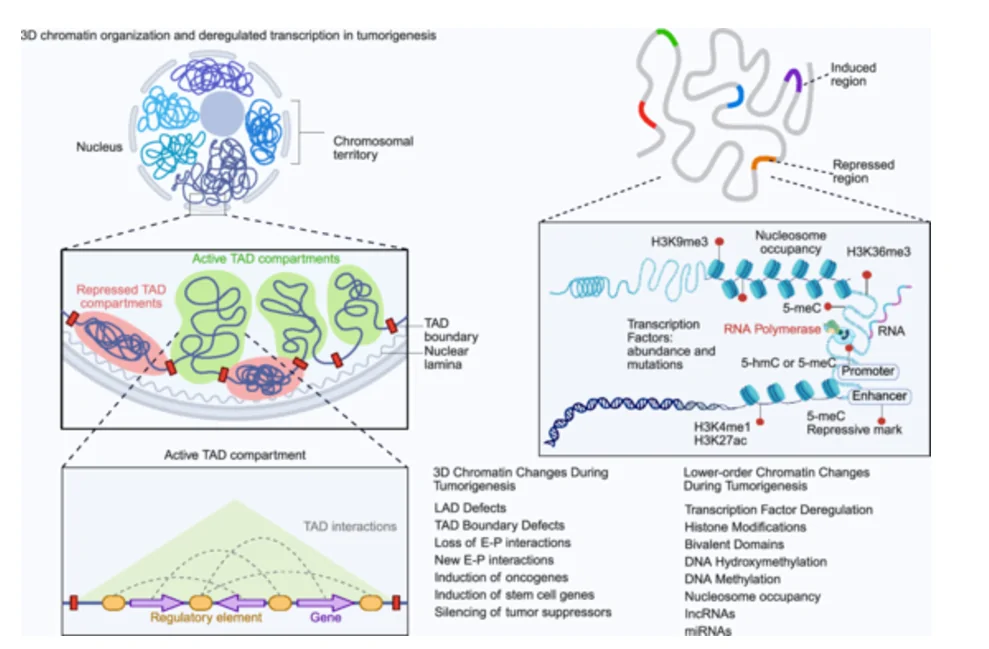
Three‐dimensional chromatin remodeling and transcriptional dysregulation mechanisms play a crucial role during tumorigenesis. Alterations in chromatin spatial organization throughout cancer development involve the disruption of TADs and LADs. These disruptions lead to abnormal enhancer–promoter interactions, resulting in oncogene activation and the silencing of TSGs. Lower‐order chromatin changes, including histone modifications, DNA methylation and hydroxymethylation, nucleosome occupancy, and ncRNA regulation, significantly contribute to transcriptional reprogramming. These multi‐level chromatin alterations reshape three‐dimensional genome architecture and drive malignant progression. LAD(s), lamina‐associated domain(s); TAD(s), topologically associating domain(s).

### RNA epigenetic modifications

m⁶A is the most abundant modification of ncRNAs. This modification profoundly influences RNA splicing, stability, translation efficiency, and subcellular localization. m⁶A is a reversible epitranscriptomic mark dynamically regulated by the coordinated activity of multiple “writers” like METTL3 and METTL14, “erasers” like fat mass and obesity‑associated protein (FTO) and alkB homolog 5 (ALKBH5), and “readers” including the YTHDFs.

Altered expression of m^6^A regulatory enzymes is commonly observed in tumors. In various cancer types, the balance of the m⁶A system is greatly disturbed. For example, the “writers” METTL3 strengthens the stability of proto‐oncogene mRNAs such as *SOX2*, thereby enhancing stemness and proliferation. Conversely, “erasers” such as FTO and ALKBH5 remove m⁶A marks oncogenic transcripts including *MYC* and *CD274*, maintaining their mRNA stability and driving cancer progression. Additionally, “readers” such as IGF2BP1 can bind m⁶A‑modified sites to stabilize key transcripts, sustaining specific tumor‐cell phenotypes [556]. Collectively, the m⁶A enzyme system constitutes a new “epitranscriptional regulatory layer” that systemically influences gene‑expression outcomes in tumor cells.

m^6^A modification regulates diverse biological processes in tumor cells, and its associated factors are pivotal in oncogenic regulation. For instance, METTL14 enhances the sensitivity of cervical squamous cell carcinoma (CESC) cells to sorafenib‐induced ferroptosis by suppressing FTH1 stability, illustrating its role in therapeutic sensitivity [557]. In signal‐pathway regulation, METTL3 regulates the tumor suppressor *LATS1* in BrCa, activating the YAP/TAZ cascade to drive proliferation [558]. In immune regulation, m^6^A‐mediated stabilization of PD‐L1 contributes to immune evasion and modulates immune‐cell infiltration [559]. Regarding tumor metabolic reprogramming, ALKBH5‐mediated m^6^A demethylation in PDAC is proposed to form a positive feedback loop with glycolysis under hypoxia, further accelerating tumor progression [560]. Thus, m⁶A modification is not a single‐factor regulator but a multifunctional component integrated into numerous oncogenic processes.

Microbial by‐products can influence the m^6^A modification landscape, thereby affecting mRNA transcription and translation. The activity of the “eraser” enzymes FTO and ALKBH5 depends notably on α‐KG, a core intermediate in the TCA cycle [561]. Consequently, microbial metabolites that alter host metabolic states—such as SCFAs like butyrate and propionate, and secondary BAs—can modulate α‐KG levels, perturb the TCA cycle, and affect the activity of RNA‐modifying enzymes. This may subsequently reshape the m^6^A landscape, influence RNA fate, and impact tumor phenotypes and therapeutic responses [562]. The “microbe–metabolism–RNA modification–tumor progression” axis thus establishes a new research direction, and RNA epigenetic modifications may serve as a breakthrough point for future metabolic interventions and epitranscriptomic therapies.

RNA epigenetic modifications represent a newly recognized regulatory layer that has been confirmed as important in the development of multiple cancers. Modifications like m⁶A, m¹A, m⁵C, and m⁷G govern RNA stability, translation efficiency, and immune visibility via the dynamic interplay of writers, erasers, and readers. Aberrations in these RNA modifications are closely associated with cell proliferation, invasion, immune evasion, and therapy sensitivity across diverse cancer types (Figure 12).

**Figure 12:**
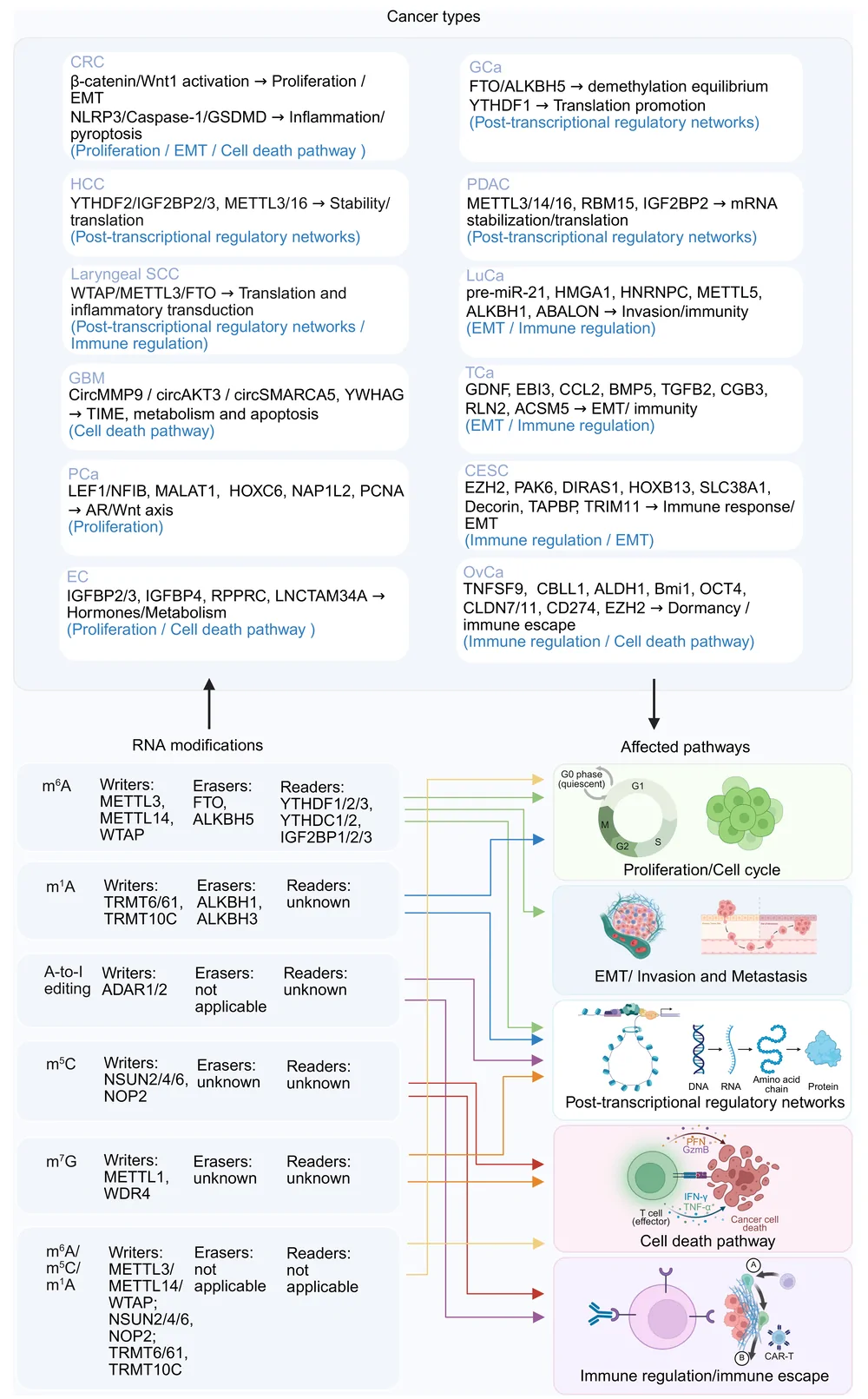
RNA modifications (m^6^A, m^1^A, m^5^C, m^7^G, A‐to‐I editing) and their roles in different cancer types and pathways. RNA modifications constitute a dynamic and reversible regulatory layer in cancer biology, mediated by specific writers, erasers, and readers that shape post‐transcriptional regulatory networks. Dysregulation of RNA modifications contributes to tumorigenesis by affecting RNA stability, translation, splicing, and immune recognition. In this schematic, RNA modifications are summarized across multiple cancer types and are associated with major functional pathways, including proliferation and cell‐cycle regulation, EMT and metastasis, cell death, and immune regulation or immune escape. For example, m^6^A modifications mediated by METTL3 and METTL14 are associated with enhanced proliferation and EMT in CRC and GCa, while demethylases such as FTO and ALKBH5 broadly influence mRNA stability and tumor–immune interactions. RNA modifications are associated with altered metabolism, apoptosis, and immune‐related pathways in GBM, PCa, and CESC. The pathway categories shown on the right summarize major biological processes that are commonly influenced by RNA modifications across cancer types. The connecting arrows indicate representative and non‐exclusive associations derived from accumulated studies, rather than strict one‐to‐one regulatory relationships between individual RNA modifications and specific pathways. CESC, cervical squamous cell carcinoma; EC, uterine cancer; m^1^A, N1‐methyladenosine; m^5^C, 5‐methylcytosine; m^6^A, N6‐methyladenosine; m^7^G, N7‐methylguanosine; TCa, thyroid cancer.

In summary, m^6^A is a key regulatory mechanism governing RNA fate, showing heightened dynamics and functional integration in tumor cells. By governing RNA stability, translation efficiency, and metabolic pathways, it sustains tumor‐cell proliferation, immune evasion, and therapy resistance. Its activity is importantly modulated by metabolic substrates, positioning it as a potential bridge between cellular metabolism and the tumor epitranscriptome. These insights provide a theoretical basis for developing therapies and regulatory strategies that target RNA modifications and the microbiome.

## MICROBIAL METABOLITES AND TUMOR EPIGENETICS

### Bacterial‐derived metabolites

#### Lipids and their derivatives

SCFAs are essential metabolites produced by anaerobic gut bacteria like *Clostridium* and *Prevotella* through the fermentation of dietary fiber. Key SCFAs include butyrate, propionate, acetate, and valerate. Through diverse epigenetic mechanisms, they influence both tumor cells and the immune system, establishing a microbiota‐mediated axis that regulates tumor biology.

SCFAs are crucial regulators of epigenetic processes, modulating chromatin structure and the expression of phenotype‑associated genes. A key epigenetic function of SCFAs is the inhibition of HDACs. Both butyrate and propionate can enter the nucleus to directly inhibit class I and II HDACs, elevating acetylation levels of histone H3 and H4. This promotes chromatin relaxation and activates TSGs like *p21* and *BAX*, leading to cell‑cycle arrest and apoptosis [563]. Butyrate has also been shown to activate the Kelch‑like ECH‑associated protein 1/nuclear factor erythroid 2‑related factor 2 (KEAP1/NRF2) pathway and modify CpG methylation profiles in CRC, thereby triggering epigenetic anti‐cancer programs [564]. In HCC models, butyrate promotes H3K27 methylation and increases DNA methylation at the *RACGAP1* and *RAC1* promoters, inhibiting the EMT process [565]. Beyond HDAC inhibition, SCFAs also serve as precursors of acetyl‐CoA, influencing the dynamic equilibrium among acetylation, lactylation, and methylation fluxes. By modulating the availability of multiple enzyme activities and substrates, SCFAs exert broad effects on chromatin architecture. Thus, SCFAs functionally interconnect metabolism, epigenetics, and gene regulation.

SCFAs reshape immune‑cell fate and exert anti‐tumor effects. For instance, butyrate and valerate enhance the effector function of cytotoxic T lymphocytes (CTLs) and CAR‐T cells by inhibiting class I HDACs and activating the mTOR pathway. This enhancement boosts the expression of CD25, IFN‐γ, TNF‐α, and other anti‐tumor mediators [326]. Pre‐treatment with these SCFAs has been shown to improve the efficacy of adoptive T‐cell therapy in melanoma and PDAC models. Butyrate, in particular, regulates multiple cancer‐suppressive processes. Under conditions of microbiome dysbiosis, it modulates autophagy and oxidative phosphorylation (OXPHOS), which is critical for immune metabolism and CRC cell survival [566]. In short, SCFAs are not merely metabolic by‐products but also serve as immune‐epigenetic remodelers that shape the anti‐tumor immune microenvironment through targeted enzyme modulation and metabolic reprogramming.

SCFAs also influence ncRNA networks by modulating miRNA‐cluster expression profiles. Butyrate upregulates the tumor‐suppressive miRNA clusters *MIR143* and *MIR145* in HCT‐116 CRC cells, which together inhibit tumor proliferation and metastasis [567]. Butyrate‑mediated alterations in histone acetylation and promoter accessibility indicate its regulatory potential at the ncRNA level, possibly driving miRNA induction. By shaping miRNA expression profiles, SCFAs establish an integrated regulatory axis that links metabolism, chromatin dynamics, and non‐coding networks, thereby amplifying their systemic anti‐tumor effects.

#### Carbohydrates and their derivatives

Microbial carbohydrate metabolites, including extracellular polysaccharides (EPS), modulate host immune responses and metabolic regulation. Beyond these roles, they influence epigenetic processes from tumor initiation through advanced progression. Their effects operate in a continuous, stage‑spanning manner.

EPS influence epigenetic regulation by modulating histone modifications through signaling pathways. Microbial EPS can engage host pattern‑recognition receptors such as TLR2/4 and C‐type lectin receptors, thereby activating the NF‐κB and STAT3 pathways. These signaling cascades recruit HATs such as p300/CBP, leading to H3K27ac and the formation of active chromatin domains. Concurrently, pro‐inflammatory cytokines including IL‐6 and TNF‐α are upregulated [568, 569]. Within the TME, this process fosters chronic inflammation, shapes immune responses, and facilitates adaptive remodeling. Thus, EPS establishes an epigenetic pathway that links exogenous carbohydrate signaling to alterations in host chromatin states via a “receptor recognition → signal transduction → chromatin remodeling” circuit.

EPS also exert anti‐tumor activity by inhibiting tumor‑cell proliferation, migration, and signaling pathways, as demonstrated in both in vitro and in vivo models. For instance, EPS364, derived from deep‐sea bacteria, suppresses the FGF19–FGFR4 signaling axis, thereby inhibiting the proliferation and adhesion of HCC cells [71]. Similarly, EPS11 interacts with collagen I to block adhesion and migration of extracellular‑matrix‑dependent tumor cells, significantly reducing lung metastasis [570]. EPS11 has also shown marked anti‐tumor efficacy in A549 cell‐derived xenograft models [571]. Thus, microbial carbohydrate derivatives not only modulate immune responses but also directly restrain tumor‐cell proliferation and migration by disrupting specific signaling pathways and adhesion mechanisms.

EPS further regulate immune responses and apoptosis, enhancing anti‐tumor outcomes. EPS produced by *Lactobacillus*, for instance, can activate CCR6^+^ CD8^+^ T cells in Peyer's patches, thereby modulating the tumor immune microenvironment and improving the efficacy of immune checkpoint inhibitors (ICIs) [572]. In a CRC animal model, EPS treatment reduced tumor volume and increased the expression of LC3 and caspase‑3, indicating enhanced autophagy and apoptosis [573]. Similarly, EPS from *Lactobacillus plantarum* activated the mitochondrial apoptosis pathway in HT‐29 CRC cells through upregulation of caspase‐3/9 and *BAX*, while inhibiting cell proliferation [574]. Thus, EPS can restrain tumor expansion by triggering mitochondrial‐mediated programmed cell death and simultaneously bolster antigen‐specific T‐cell responses, thereby coordinately targeting both immune and tumor cells.

#### Amino acids and their derivatives

Microbial‐derived metabolites, including amino‐acid derivatives, play a dual role in tumor epigenetic regulation. Tumor cells can exploit these compounds to fuel oncogenic metabolism and evade immune surveillance. Conversely, the same metabolites—produced through specific microbial metabolic pathways—can also function as epigenetic regulators that antagonize tumor progression. These opposing functions are best understood as interconnected elements within a unified narrative rather than as isolated mechanisms.

Tumor cells hijack amino‐acid metabolic pathways to fuel their metabolic reprogramming and epigenetic evasion, thereby driving progression. For example, in HCC, accumulated arginine interacts with RBM39, regulating the transcription of metabolic genes and establishing a positive‐feedback epigenetic loop that promotes tumor initiation [575]. In BrCa, ALKBH5 undergoes arginine methylation via PRMT6, which stabilizes LDHA mRNA, enhancing glycolysis and tumor growth [576].

Within immune‐suppressive and evasion mechanisms, tumor cells can promote chromatin condensation in T cells through arginine depletion, which downregulates ASS1 expression and promotes immune evasion [577]. Overproduction of ARG1 increases Treg infiltration into tumors, accelerating BrCa growth [578]. Under arginine‐deficient conditions, the AKT/RNF167 pathway promotes CASTOR1 degradation, activating the mTORC1 and enhancing tumor progression [579]. Additionally, CARM1 facilitates HIF1A binding promoters of stemness‐related genes, potentially driving the malignant phenotype of TNBC [580]. CARM1 also interacts with the NuRD complex, representing a potential therapeutic target [290]. Collectively, cancer cells subvert metabolism‐epigenetics crosstalk by hijacking amino‐acid fluxes and modifying epigenetic reprogramming, establishing an oncogenic program characterized by enhanced glycolysis, immune suppression, and mTOR activation.

Microbial metabolites can counteract tumorigenic programs and exert anti‐cancer effects through epigenetic regulation. Activation of the AhR by tryptophan metabolites or ligands (e.g., indolepropionic acid) enhances Ezh2‐mediated H3K27me3 while reducing HDAC activity, thereby increasing H3K9ac. AhR activation by these metabolites also downregulates immune‐suppressive molecules such as PD‐L1 and IDO1, improving immune recognition [581].

Arginine‐derived polyamines (e.g., spermidine) regulate the balance between HAT and HDAC activity, enabling bidirectional modulation of acetylation at both pro‐oncogenes and TSGs. This regulatory effect is dose‐dependent and context‐specific rather than absolute [582].

SAM, a derivative of the essential amino acid methionine, serves as the universal methyl donor for DNMTs. The cellular DNA methylation state is governed by the SAM/SAH ratio [583]. Moreover, SAM supplementation has been shown to enhance CD8^+^ T cell function and suppress tumor growth in a CRC model [584].

Microbial arginine metabolism regulates immune pathways—such as those involving *FOXP3* and *STAT1*—promoting immune activation and tumor suppression and highlighting its potential for immunometabolic intervention [585]. In summary, microbial‑derived metabolites function as enzyme substrates, signaling ligands, or regulatory factors, broadly influencing DNA methylation, histone modifications, and immune‐signaling outputs. They have demonstrated epigenetic‑modulating and tumor‐suppressive effects across diverse preclinical tumor models.

#### Nucleotides and their derivatives

Nucleotide‐derived compounds from microbial metabolism often display a “dual role” in tumor epigenetic regulation. Some molecules stimulate immune activation against tumors and promote epigenetic marks associated with anti‐cancer effects. On the other hand, other derivatives are co‐opted by tumor‐cell immune‐suppressive networks to boost epigenetic silencing and immune evasion.

Nucleotides and their microbial‐derived derivatives participate in epigenetic and immune activation for anti‐tumor regulation. Bacterial CDNs (e.g., c‐di‐AMP) activate the host STING pathway, inducing a type‐I interferon response and recruiting p300/CBP to enhance H3K27ac. This promotes immune‐related gene expression and strengthens anti‐tumor immunity [586]. NAD^+^ serves as a key substrate for the SIRT family of HDACs, which are influenced by both microbial and host metabolic processes. SIRT1 and SIRT2 deacetylate histone marks such as H3K9ac and H3K27ac, thereby modulating tumor suppressors including p53. NAD^+^ supplementation enhances SIRT‐mediated anti‐proliferative signaling, resulting in reduced tumor growth [587]. NAMPT upregulates α‐KG levels, boosting TET1 activity. TET1‐mediated demethylation of the *IRF1* promoter increases PD‐L1 expression in tumors, sensitizing them to ICIs [588]. Vitamin D3 promotes SIRT1 self‐deacetylation, facilitating H3K27 deacetylation and exerting anti‐proliferative effects [589]. Collectively, microbial nucleotide metabolites that engage pathways such as STING–p300, SIRT–NAD^+^, TET1–IRF1 enhance chromatin accessibility and immune‐gene expression, establishing an integrated “microbiome–metabolism–epigenetics–immune” axis for anti‐tumor defense.

Nucleotides and their microbial‐derived derivatives can be hijacked by the TME to induce epigenetic silencing and immune suppression, thereby promoting cancer progression. For instance, adenosine derived from ATP degradation activates the adenosine A2A receptor and the cAMP/PKA pathway, leading to Ezh2 activation. This increases the repressive histone mark H3K27me3 while reducing the active marks H3K4me1/2, thereby enhancing transcriptional silencing and immune evasion [590, 591]. Overexpression of NNMT in cancer‐associated fibroblasts (CAFs), induced by SAA, drives epigenetic reprogramming. This process recruits immune‐suppressive macrophages, forming a PD‐1/PD‐L1‐resistant tumor niche [592]. Although SIRT1 is a deacetylase, its activity is tightly regulated by fluctuating NAD^+^ levels. Depletion or inhibition of NAMPT impairs SIRT1‐mediated deacetylation of key targets such as H3K27ac, p53 and FOXO3a, compromising tumor‐suppressive functions [593]. Furthermore, metabolic signaling through GID8 and PCK1 via the glutamine (Gln)/PI3K–AKT axis promotes tumor proliferation [213, 594]. Additionally, YTHDF2 degrades TSG mRNAs through m^6^A‐dependent pathways, exacerbating transcriptional imbalance in PDAC [595]. In summary, nucleotide derivatives can lead to oncogenic epigenetic remodeling by elevating H3K27me3, silencing TSGs, and facilitating immune evasion through three main pathways: They are adenosine–Ezh2 axis, the NNMT–CAF axis, and NAD^+^ depletion. These alterations collectively render the TME more resistant to therapy.

#### Vitamin derivatives

Microbially synthesized B‐vitamin derivatives, especially vitamin B12 and folate, influence the epigenetic state of tumors through their roles in one‐carbon metabolism. The production and stability of the universal methyl donor SAM, a central output of one‐carbon metabolism, depend directly on the folate and methionine cycles.

An inverse correlation exists between vitamin B12 levels and DNA methylation in CRC. Vitamin B12 deficiency accelerates DNA methylation in CRC patients [596, 597]. Adequate B12 supports the epigenetic reprogramming of tissue‐repair genes [86]. Vitamin B12 is also recognized for modulating both global and gene‐specific DNA methylation, as well as histone modifications. Moreover, it influences imprinting gene expression [598]. Under conditions of dietary imbalance—such as low B12 and high folate—vitamin B12 can exert trans‐generational effects on gene expression by altering histone marks [598].

In mouse models, a high‐folate diet promotes HCC development, which is related to upregulation of MAT2A. This enzyme reprograms one‐carbon metabolism to support tumor growth. Additionally, IFN‐γ is capable of enhancing PD‐L1 expression through O‐GlcNAc modification of *MYC*, as it has been shown to regulate MTHFD2, a critical enzyme in the folate cycle [599].

It is noteworthy that folate exerts context‐dependent, bidirectional effects on DNA methylation. Folate supplementation can induce either hyper‐ or hypomethylation in CRC cells, with the direction and magnitude of change strongly influenced by the molecular and epigenetic characteristics of the cells [600]. In BrCa cells, SIRT4 senses folate levels and inhibits proliferation by promoting the deacetylation and degradation of MTHFD2 [1]. Dietary folate can also modulate the methylation of the *p16* gene and influence CRC risk [601]. In CRC, acetylation modifications of ALDH1L2 [602] and promoter methylation of *MIR375* [603] play crucial regulatory roles. In GCa, *MIR22* represses *MTHFD2* and *MTHFR*, leading to reduced endogenous SAM levels. This restoration of TSG expression—including *p16*— helps inhibit tumor growth [604].

The interaction of microbial‐derived B vitamins, amino acids, and diol metabolites with SAM‐dependent cycles constitutes a key mechanism for regulating DNA methylation density and imprinting stability across biological contexts.

#### Organic acids

Organic acids produced by microbial and host cells play an important role in the regulation of tumor immunity and progression through novel epigenetic mechanisms. Beyond the classical inhibition of HDACs, organic acids including lactate, succinate, fumarate, and itaconate act as substrates or allosteric regulators that directly participate in histone modifications.

Within the TME, ILA strengthens H3K27ac at the *IL12A* enhancer, enhancing dendritic‐cell function and activating the anti‐tumor activity of CD8^+^ T cells [605]. Elevated lactate levels can induce Kla. For instance, in therapy‐resistant CRC, H4K8la promotes autophagosome maturation, enabling tumor‐cell survival under hypoxia. In addition, as a direct SCFA, lactate enhances *MYC* transcription via histone acetylation, thereby promoting CSC states [606]; SCFAs also directly supply precursor molecules for histone acetylation [607]. In esophageal squamous cell carcinoma, a hypoxic, lactate‐rich environment further enhances H4K8la, elevating BMP7 transcription and driving tumor progression [608].

Succinate and fumarate are not only TCA cycle intermediates but also competitive inhibitors of the TET and JmjC domain‐containing demethylase families. Elevated succinate levels can profoundly reshape epigenetic landscapes and Wnt/β‐catenin signaling in *KRAS*‐mutant CRC, thereby enhancing tumor stemness and therapy resistance [216].

Macrophages produce itaconate, which selectively inhibits class I HDACs, including HDAC1, HDAC2, HDAC3, and HDAC8. The conversion of itaconate to itaconyl‐CoA depletes the cytosolic CoA pool, indirectly influencing CoA‐dependent acetyltransferase activities and potentially impacting histone butyrylation. This process enhances anti‐inflammatory gene expression. However, in HCC, macrophage‑derived itaconate can induce H3K4me3 on CD8^+^ T cells, upregulating the *EOMES* promoter and promoting CD8^+^ T cell exhaustion, thereby driving HCC progression [609]. In tumor‐associated macrophages (TAMs), OXCT1 promotes CD8^+^ T cell exhaustion through the succinate‐H3K4me3‐Arg1 axis [610].

Therefore, TCA cycle intermediates such as succinate and fumarate act as competitive inhibitors of TET and JmjC domain‑containing demethylases, restricting their activity. Furthermore, they deplete the pool of α‐KG. Altogether, these metabolites limit substrate availability and hinder the demethylation process. In addition, chromatin accessibility—and consequently the accessibility and expression of immune‑effector genes—is jointly influenced by these metabolites, the NAD^+^/FAD redox state, glutathione (GSH) synthesis capacity, and lactate‐triggered Kla levels. This intersection of metabolism and epigenetics represents a crucial regulatory node for remodeling the tumor immune microenvironment.

#### Alcohols

Alcohols derived from both microbiota and host metabolism—such as ethanol, acetaldehyde, and diols—play complex epigenetic roles in tumorigenesis through multiple mechanisms. These include modulation of histone acetylation, DNA methylation, and one‐carbon metabolism. While ethanol metabolism can unleash oncogene expression and sustain stemness, microbial‑derived phenolic alcohols and diols may exert epigenetic anti‑cancer activity, showcasing a form of “bidirectional regulation”.

Ethanol metabolism drives epigenetic activation. Ethanol oxidizes to acetaldehyde, which is subsequently converted to acetyl‐CoA, a key substrate for HAT‐mediated catalysis. This process significantly increases acetylation of histone H3K9 and histone H4K16, thus enhancing the activity of oncogenes like *MYC* [611]. In oral squamous cell carcinoma, upregulation of acetaldehyde‐induced acetylation, activation of the NFAT pathway, and enhanced aerobic glycolysis together increase the CSC pool and sustain stemness [612]. In addition, the increased supply of acetaldehyde and acetyl‐CoA, coupled with SCFAs‐derived carbon and NAD⁺/SIRT‐mediated deacetylation, establishes a “push‐pull mechanism” that defines the threshold and reversibility of oncogene transcriptional activity. This interplay holds substantial epigenetic significance in alcohol‐associated carcinogenesis.

Microbial‐derived phenolic alcohols, such as tyrosol produced by *Lactobacillus* species, have been implicated in host epigenetic modulation. In parallel, dietary polyphenols such as curcumin have been shown to inhibit DNMT1 and induce promoter‐specific demethylation of tumor suppressor genes, exemplified by DLC1 in BrCa models [613]. In models of alcoholic HCC, TNF‐α activated NF‐κB p65 recruits DNMT1 to the *PSD4* promoter, leading to DNMT1‐dependent hypermethylation and silencing of *PSD4*. This suppression, driven by NF‐κB activation, drives EMT and tumor progression; conversely, the restoration of *PSD4* expression inhibits HCC expansion [614]. Furthermore, diols fermented by specific anaerobic bacteria interfere with one‐carbon metabolism, reducing the SAM/SAH ratio and thus inhibiting HMT activity. This, in turn, limits the availability of methyl donors for oncogenes, illustrating another potential tumor‐suppressive regulatory mechanism [615].

#### Others

Besides the categories discussed, special metabolites such as hydrogen sulfide (H₂S) and 4‐acetylphenol sulfate (4‐EPS) also exhibit epigenetic regulatory functions and demonstrate anti‐cancer activity.

H₂S produced by sulfate‐reducing bacteria is genotoxic and may contribute to the progression of chronic intestinal diseases, including CRC [616]. Its intestinal effects are concentration‐dependent: at low levels, H₂S has a protective role, whereas high concentrations cause inflammation and tissue damage. H_2_S is also important in epigenetic regulation, particularly for Treg differentiation and function. It can S‐sulfhydrylate the nuclear transcription factor NF‐YB, which enhances its binding to the *TET1* and *TET2* promoters and sustains the expression of these DNA demethylases. Together with transforming TGF‐β and IL‐2 signaling, *TET1* and *TET2* are recruited to the *FOXP3* locus, where they catalyze the conversion of 5mC to 5hmC. This establishes a Treg‐specific hypomethylation pattern, stabilizing *FOXP3* expression and maintaining immune homeostasis [617]. The influence of H₂S on the TET axis and Treg homeostasis is further connected to the folate‐SAM cycle and NAD⁺/SIRT pathways, indicating that sulfur‐containing metabolism is a third important dimension that connects “metabolism–epigenetics–immunity.”

The gut microbiome metabolite 4‐EPS has selective anti‐cancer activity against CRC. In vitro studies reveal that 4‐EPS can upregulate *BAX* and downregulate *BCL2*, inducing G₂/M phase cell‐cycle arrest. Molecular simulations also reveal that 4‑EPS interacts strongly with HDAC isoforms, suggesting that its anti‐cancer effect likely involves HDAC inhibition. Notably, 4‐EPS does not cause significant toxicity to healthy colon epithelial cells, highlighting its potential as a promising therapeutic agent for CRC [618].

### Fungal‐derived metabolites

#### Mycotoxins

Mycotoxins are toxic metabolites produced by several fungi (e.g. *Aspergillus*, *Fusarium*, and *Penicillium*) during storage or fermentation of crops. These compounds are prevalent in grains, nuts, spices, and dairy products. They possess potent mutagenic and immunotoxic properties and are believed to promote tumorigenesis through epigenetic mechanisms. Ingestion usually takes place through the oral route, leading to accumulation in metabolically active tissues like the liver, kidney, and intestine. Their carcinogenic activity is characterized by considerable epigenetic targeting and exhibits distinct time‐ and tissue‐dependent patterns.

Aflatoxin B1 (AFB1) is considered one of the strongest carcinogens in food, causing cancer by modifying epigenetic levels in various biological systems. Studies have shown that AFB1 overexpresses DNMT1 to maintain the hypermethylation, or silencing, of the promoters of TSGs such as *p16INK4a*. In parallel, AFB1 acts as a functional ligand of the AhR, inducing its nuclear translocation and activating the AhR CYP axis responsible for xenobiotic metabolism, with potential implications for broader metabolic reprogramming [619]. Aberrant activation of AhR signaling has been broadly implicated in carcinogenic and immunomodulatory processes [620]. Moreover, AFB1 can interfere with core cellular processes. For instance, it can stop the normal cell cycle and proliferation, and boost apoptosis and oxidative stress. Abnormal histone modifications (e.g., H3K27me3, H3K9me3) and ncRNA remodeling underlie these effects. The multidimensional epigenetic toxic mechanisms of AFB1, a classic representative of fungal toxins, are shown in Figure 13.

**Figure 13:**
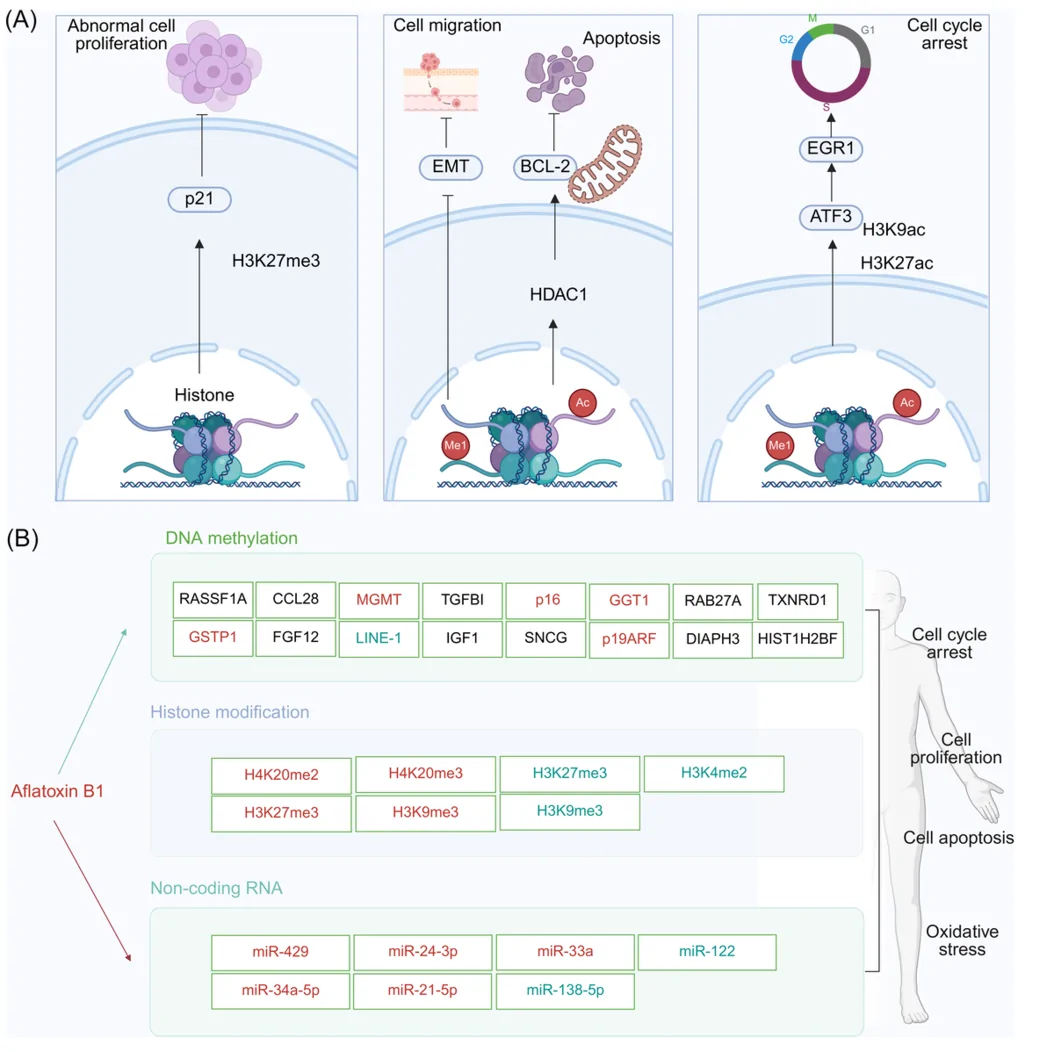
Aflatoxin B₁ mediates epigenetic mechanisms of cell cycle, proliferation, apoptosis and oxidative stress through DNA methylation, histone modification and ncRNA regulation. (A) Epigenetic changes induced by AFB1 significantly influence abnormal cell proliferation, apoptosis, and cell cycle arrest. For instance, the H3K27me3 modification suppresses the expression of p21, while HDAC1 upregulates BCL‐2, leading to inhibition of apoptosis. Additionally, histone acetylation, specifically H3K9ac and H3K27ac, modulates the activity of transcription factors such as EGR1 and ATF3. (B) AFB_1_ exerts its oncogenic potential through the DNA methylation of tumor suppressor genes and other cancer‐related loci (e.g., p16, RASSF1A, GSTP1), histone modification (e.g., H3K27me3, H3K9me3, H4K20me2), and dysregulation of ncRNA (e.g., miR‐24, miR‐34a, miR‐138‐1). Collectively, these mechanisms drive cell cycle arrest, aberrant proliferation, apoptosis, and oxidative stress. Color coding indicates the direction of regulation by Aflatoxin B_1_: In (A), red spheres denote epigenetic chemical groups (Ac, Acetylation; Me, Methylation). In (B), text colors differentiate expression or modification status: red, upregulated or hypermethylated; green, downregulated or hypomethylated. AFB_1_, aflatoxin B_1_.

The KDM5B is induced by ochratoxin A (OTA) and removes the activating epigenetic mark H3K4me3. This histone modification leads to chromatin compaction and the downregulation of tumor suppressor and DNA repair genes. In addition, by inhibiting the cellular repair of DNA double‐strand breaks, OTA induces chromosomal instability in renal tubular epithelial cells, further contributing to kidney cancer [621].

Zearalenone (ZEN), a mycotoxin structurally similar to estrogen, can bind to estrogen receptor alpha (ERα). The methyltransferase Ezh2, associated with Erα, is recruited to target genes in collaboration with ERα. This process catalyzes the accumulation of the repressive histone mark H3K27me3, facilitating the silencing of TSGs like *BRCA1*. The fungal toxin fumonisin B1 (FB1) affects the PTEN/PI3K/AKT signaling pathway and induces DNA damage [622]. Based on these findings, mycotoxins can drive tumorigenesis via several epigenetic mechanisms.

The epigenetic carcinogenic signals of mycotoxins do not act in isolation. They can engage in crosstalk with host one‐carbon metabolism, NAD^+^/SIRT pathways, the SCFAs‐HDAC axis, and other pathways to amplify or buffer their carcinogenic effects, depending on the tissue and timescale.

#### Yeast metabolites

During fermentation, yeast releases small‐molecule metabolites that, on the one hand, promote tumorigenesis through signaling pathways and, on the other hand, may exert anti‐tumor immunity in an epigenetic manner. Specifically, ergothioneine (EGT), GSH, and their derivatives regulate cell proliferation, metabolic reprogramming, and immune evasion, demonstrating both pro‐carcinogenic and anti‐cancer effects.

EGT is a sulfur‐containing amino acid derivative widespread in *Sepedonium pyriformis* and other microorganisms. It is a compound capable of preventing or blocking the action of harmful substances. EGT can either act as a catalyst for cellular damage, usually promoting carcinogenesis, or inhibit the growth of tumor cells. It inhibits the activity of the HAT p300, which activates oncogenes such as *c‐MYC*, thereby reducing the transcriptional levels of *c‐MYC*. Furthermore, it might prevent the establishment of epigenetic marks associated with cellular growth [623]. This blocking effect may interfere with the normal control mechanisms of cell growth in certain cancers, allowing tumor progression. The blockade of p300 not only hampers the transcription of oncogene but also triggers necroptosis via the SIRT3‐MLKL signaling axis in particular cancer cell types. This action is an important mechanism underlying the anti‐tumor effects of EGT [623]. Additionally, EGT suppresses PD‐L1 expression in TAMs, which may weaken the immune evasion mechanism, thus enhancing the immune response against the tumor [624].

GSH is an important antioxidant present in the reduced form within cells and is involved in many metabolic processes. GSH and its yeast fermentation by‐products can exhibit both tumorigenic and anti‐tumor effects. An increase in GSH concentration depletes SAM in one‐carbon metabolism, thus globally reducing DNA and histone methylation and resulting in a demethylated state. This process may unwittingly trigger the expression of some oncogenes and drive tumor cells towards an invasive phenotype. In a colitis‐associated cancer model, selenium‐enriched methionine prevents tumor development and decreases myeloid‐derived suppressor cells (MDSCs), highlighting the intricate role of GSH regulation in the TME [625]. Overconsumption of GSH may initiate ferroptosis, whereas its suppression might occur via the Wnt/NR2F2/GPX4 pathway and enhance chemoresistance [626]. Conversely, excessive GSH depletion may result in tumor cell death through ferroptosis, thus inhibiting tumor growth. In RAS‐mutated tumors, combined inhibition of NADPH oxidase and GSH synthesis has a synergistic lethal effect [627]. In PDAC models, cannabidiol induces tumor cell apoptosis by lowering GSH levels, suggesting that GSH depletion can boost tumor suppression by altering redox status and activating ferroptosis pathways. The GSH metabolic network, interacting with NAD^+^, SAM, and α‐KG pools, demonstrates context‐dependent epigenetic effects under different stress conditions, manifesting as a dual “chemotherapy resistance promotion‐ferroptosis induction” phenotype [626, 628]. This suggests that GSH may, in some contexts, orchestrate immune and metabolic responses to protect tumor cell viability, while in others, it promotes tumor suppression by inducing ferroptosis [629].

#### Filamentous fungal metabolites

The secondary metabolites of filamentous fungi modulate tumor cell activity by targeting histone‐modifying enzymes. Specifically, these fungal compounds and their precursors influence the action of JMJD3 and SUV39H1. They facilitate the establishment of different histone methylation marks in a complex regulatory apparatus, thereby enhancing the regulatory activity at the tumor cell‐system level. For example, penicillic acid, a metabolite from *Penicillium*, acts as an allosteric activator of the KDM6B. The reinforcement of JMJD3 catalytic activity by penicillic acid aids in removing the suppressive H3K27me3 mark from TSG promoters. This reactivates genes like *p21* and *GADD45A*, which then inhibit tumor cell proliferation [630, 631, 632, 633, 634].

Chaetocin, secreted by *Chaetomium fungi*, is an inhibitor of the H3K9 methyltransferase SUV39H1 and a potent inhibitor of the KMT5A. Chaetocin impairs the monomethylation of histone H4K20, thereby elevating DNA replication stress and chromatin instability. Consequently, the DDR response is activated, leading to cancer cell apoptosis [193].

Under nutrient‐deprived conditions, several fungal secondary metabolites induce the accumulation of ROS by targeting the GSH and thioredoxin systems, driving apoptosis in PDAC cells [635]. Fungal estrogens enhance the conversion of estrogen into the highly tumorigenic metabolite 4‐hydroxyestradiol [636]. ZEN promotes tumor progression through multiple mechanisms, including activation of the BEST4/AKT/ERK1/2 pathway [637], modulation of Rho/ROCK signaling [638], and protection against oxidative stress [639]. Furthermore, ZEN suppresses intestinal T‐cell activation via the 27‐HC/LXR/ApoE axis [640] and activates signaling pathways such as MAPK and Hippo‐YAP1 [641].

Fungal toxins, including gliotoxin and its derivatives, act as potent inhibitors of LSD1, demonstrating significant potential for the treatment of BrCas and GCas [642, 643].

Consequently, the diverse regulatory influences exerted by filamentous fungal metabolites on a range of enzymes, including LSD1, JMJD3, SUV39H1 and SETD8, combined with their dynamic in vivo interactions with host metabolic cofactor pools, orchestrate a network‐level reorganization of various histone marks such as H3K27me3, H3K9me3, and H4K20me, in the host.

#### Endophytic fungal products

Endophytic fungi are a major source of natural products and can produce various small molecules with epigenetic activity. These agents have specific effects on cancer cell stimulation, apoptosis, migration, and other processes by modulating epigenetic enzymes, transcription programs, and immune responses [644, 645].

Paclitaxel and its analogs are classic anticancer drugs that act as microtubule stabilizers to block cell division. BRD4 recognizes acetylated histones and activates the transcription of oncogenes such as *c‐MYC* [646]. Although paclitaxel does not directly target BRD4, paclitaxel‐induced alterations in chromatin structure and cellular stress signaling may secondarily affect BRD4 chromatin occupancy. Such changes can influence BRD4‐dependent transcriptional programs, leading to reduced expression of proliferative genes in certain cellular contexts [647].

A major group of endophytic fungal metabolites are cyclic peptides, particularly a class known as depsipeptides. These compounds specifically modulate the enzymatic activity of the KDM1A. LSD1 is a monomeric FAD‐dependent histone demethylase that removes the activating H3K4me1/2 marks and contributes to the epigenetic silencing of TSGs [648, 649]. Inhibition of LSD1 has been shown to increase H3K4me2 levels and reactivate aberrantly silenced genes, thereby restraining tumor cell proliferation and invasion [650]. Recent studies have further developed macrocyclic and peptide‐based LSD1 inhibitors as promising epigenetic therapeutics in cancer [651, 652]. Cyclic peptides induce the dissociation of LSD1 and BRD4 from their chromatin binding sites. Additionally, their binding to DNMT/TET, SIRT, and KAT/HDAC modules enables programmable epigenetic combination therapy.

Several compounds derived from endophytic fungi have demonstrated significant anticancer activity. However, potent bioactive compounds are also produced by endophytic bacteria. Notably, *Streptomyces sp*. LRE541, an endophytic actinobacterium rather than a fungus, exhibits strong anticancer effects. Its extracts induce S‐phase cell‐cycle arrest and apoptosis in a dose‐dependent manner, thereby inhibiting the proliferation of RKO and 7901 cancer cells [653]. Other compounds inhibit tumor growth by targeting critical signaling pathways, including EGFR/PI3K/Akt [654], JAK3/STAT [655], and PI3K/AKT/FoxO1 [656]. Notably, 8‐n‐butylnorbelladine induces apoptosis in Huh‐7 cells through the mitochondrial apoptosis pathway [657], while VDT targets mitochondrial OXPHOS, presenting a new therapeutic strategy for leukemia and lymphoma [658]. Additionally, extracts from *L. marginatus* endophytic fungi exhibit significant in vitro anticancer activity [659]. Fusaric acid E inhibits the growth of HT29 xenograft tumors, with Beclin‐1 identified as a potential target [660]. Furthermore, ascorbate exerts its anticancer effect by inducing immunogenic cell death [661].

### Virus‐induced host metabolites

Viruses alter the host cell's epigenome through the proteins they encode and the small molecules they regulate; this process is thought to facilitate tumor initiation and promotion. The regulation of viral infections is known to involve basic epigenetic processes, including changes in DNA methylation, histone modifications, and ncRNA expression.

#### DNA virus‐encoded products

DNA viruses encode essential oncogenic proteins that can reprogram the host epigenome. The NuRD complex remodels host chromatin through the MBD2/MBD3 component via the E6 and E7 oncogenes of HPV [662]. The E7 protein directly binds to the core PRC2 complex methyltransferase Ezh2, leading to elevated levels of H3K27me3 at TSG promoters (e.g., *p16*) and corresponding gene silencing [663, 664]. Moreover, cervical cancer can result from the upregulation of UHRF1 expression mediated by E6 and E7. This upregulation, along with an E6/E7‐induced increase in USP7 levels (which stabilizes UHRF1), leads to altered transcriptional CpG methylation at the TXNIP promoter, contributing to cervical cancer development [665]. Furthermore, HPV16 E2 has been shown to inhibit the promoter of the EMT master regulator *TWIST1*, which can improve prognosis [666].

The Epstein‐Barr virus (EBV) latent membrane protein 1 (LMP1) facilitates the nuclear translocation of DNMT3A, thereby enhancing its catalytic activity on the methylation of TSG promoters, such as that of *PTEN*. This process drives the progression of nasopharyngeal carcinoma and other malignancies. Concurrently, LMP1‐induced metabolic alterations, such as those involving α‐KG, disrupt the homeostasis of global DNA methylation and demethylation, which inhibits the generation of 5hmC and impacts the virus's lytic/latent switch [667]. Furthermore, EBV infection reprograms the epigenome by modifying chromatin accessibility [668], while epigenetic silencing mechanisms, including DNA methylation, are implicated in the regulation of viral gene expression during latency.

Hepatitis B virus (HBV) X protein (HBx) binds to and inhibits the NAD⁺‐dependent HDAC SIRT1. This interaction results in elevated levels of the acetylated histones H3K9ac and H4K16ac at pro‐inflammatory and pro‐proliferative gene promoters, enhancing their transcription. Simultaneously, it impairs DNA damage repair mechanisms, accelerating the malignant transformation of hepatocytes.

These viral proteins modulate the availability of SAM, NAD^+^, and α‐KG, enabling crosstalk between viral oncogenic signaling and host microbiome metabolites within overlapping pathways. Table 13 summarizes the evidence chain and mechanistic steps by which host metabolites induced by DNA tumor viruses influence epigenetic regulation.

**Table 13:** The role of virus‐related metabolites in tumor epigenetics.

Metabolite	Metabolic pathway	Epigenetic effects	Related tumor types	References
UDP‐GlcNAc	Hexosamine pathway	As a donor for O‐GlcNAc modification, it modifies the T378 site of the CHK2 protein under glucose starvation. Its rate‐limiting enzyme, GFAT, has been confirmed as a viable anti‐cancer target. Inhibition of GFAT can reduce O‐GlcNAcylation levels, suppress tumor growth, and induce tumor cell apoptosis.	HCC	[669, 670]
SAM	One‐carbon metabolism	As a methyl donor, it regulates DNA and histone methylation, particularly hypomethylating genes such as p16, PTEN, and RASSF1A. Metabolic reprogramming, driven by NNMT, leads to a reduction in histone methylation within the stroma. MeCP2‐regulated miR‐22 induces an endogenous SAM deficiency by targeting MTHFD2 and MTHFR.	GCa; OvCa	[604, 671]
Gln	Gln metabolism	Generates α‐KG and other TCA cycle intermediates through catabolism; provides essential γ‐nitrogen sources for purine and pyrimidine biosynthesis to support proliferation of virus‐transformed cells	Kaposi's sarcoma	[672, 673]
*N*‐(3‐aminopropyl)‐butane‐1,4‐diamine, spermidine	Polyamine biosynthesis	Stabilizes the YBX1 protein to inhibit autophagy. Polyamine depletion (e.g., using DFMO) suppresses global translation in BrCa cells, while spermidine supplementation can reverse this inhibition.	BrCa (particularly TNBC)	[674, 675]
Gamma‐aminobutyric acid	Glycolysis/Warburg effect	DNA methylation leads to low expression of ABAT in HCC	HCC	[676]
Lactate	Glycolysis/Warburg effect	Metabolic reprogramming characterized by lactate export, decreased oxygen consumption rate (OCR) and NAD^+^ levels, and increased ROS promotes cell survival and acquired resistance	Virus‐associated lymphoma	[677]
Cholesterol	Cholesterol synthesis	Inhibition of SREBP signaling activates the compensatory mTORC1‐pS6 pathway. Combined targeting of SREBP and mTORC1 synergistically suppresses lymphoma proliferation, cell cycle progression, and lipid raft formation.	B‐cell lymphoma	[678]
Acetyl‐CoA	Fatty acid β‐oxidation	Accumulated acetyl‐CoA epigenetically activates ACSL4 transcription, leading to increased fatty acid β‐oxidation activity and the maintenance of HCC cell stemness.	Leukemia; HCC	[679, 680]
dNTPs	Nucleotide de novo synthesis	Promotion of de novo nucleotide synthesis (by increasing R5P) alleviates DNA damage and genomic instability during liver injury, reduces inflammatory responses (TNF‐α, IL‐6), STAT3 signaling, liver steatosis, and fibrosis, and ultimately inhibits HCC development	HCC	[681]
5mC	One‐carbon metabolism	LINE‐1 and global DNA hypomethylation are associated with reduced in situ levels of methyl donor molecules (folate and SAM)	CRC	[682]
NADP	Tryptophan–kynurenine pathway	GLI1 and STAT3 cooperatively bind to the cis‐regulatory region of IDO1 and increase active chromatin marks at the gene level	Melanoma	[683]
NAD^+^	NAD^+^ salvage pathway	Activates SIRT1 deacetylase and inhibits NF‐κB inflammatory signaling	Lymphoma	[684]
Riboflavin (Vitamin B2)	Vitamin B2 metabolism	UPR (via XBP1s) upregulates TXNIP and activates the NLRP3 inflammasome, overcoming barriers to EBV lytic cycle replication Riboflavin deficiency induces endoplasmic reticulum stress and the CHOP pathway, leading to cell apoptosis	DLBCL; HCC	[685, 686]
2‐HG	α‐KG‐dependent demethylase inhibition	α‐KG‐dependent demethylase inhibition	EBV‐associated nasopharyngeal carcinoma and other head and neck squamous cell carcinoma subtypes	[687]
Fumarate	TCA cycle	Inhibition of TET demethylases (enhances DNA methylation)	EBV‐associated nasopharyngeal carcinoma (HNSCC subtype)	[688]
Itaconate	Citric acid cycle (corynebacterial branch)	Promotes succinate‐dependent histone H3K4 trimethylation	HCC	[609]
Serine	Serine/one‐carbon metabolism and methionine cycle	Maintains the methionine cycle by de novo ATP synthesis and provision of one‐carbon units, supporting DNA and RNA methylation; serine deficiency significantly reduces DNA and RNA methylation levels	CRC	[689]
PGE2	Arachidonic acid metabolism (prostaglandin pathway)	Upregulates the expression and activity of *DNMT3B*, leading to genomic DNA hypermethylation [e.g., promoter methylation of O6‐methylguanine‐DNA methyltransferase (MGMT), CNR1, and POT1 genes]	GCa (Gastric Mucosal Cancer)	[690]
Succinate	TCA cycle	Competitively inhibits α‐KG‐dependent dioxygenases (e.g., histone demethylases, TET DNA demethylases), leading to histone and DNA hypermethylation	Pheochromocytoma, Paraganglioma	[691, 692]
Fumarate	TCA cycle	Competitively inhibits α‐KG‐dependent dioxygenases (e.g., histone demethylases, TET DNA demethylases), resulting in histone and DNA hypermethylation	HLRCC	[691, 692]
Polyamines	Polyamine synthesis pathway	Inhibit the activity of histone lysine demethylases, such as LSD1, resulting in elevated levels of H3K4 methylation, which subsequently influences the epigenetic regulation of gene expression, exemplified by the upregulation of ID1	CSC population	[693]
Betaine	One‐carbon metabolism (methionine cycle)	Promotes SAM synthesis via betaine‐homocysteine methyltransferase (BHMT), specifically increasing N6‐methyladenosine (m^6^A) RNA methylation levels	HCC	[694]

Abbreviations: BHMT, betaine‐homocysteine methyltransferase; m^6^A, N6‐methyladenosine; MGMT, O^6^‐methylguanine‐DNA methyltransferase; OCR, oxygen consumption rate.

#### RNA virus effectors

The main protein of hepatitis C virus (HCV) modulates host epigenetics through a dual regulatory mechanism. It recruits HDACs and the PRC2 to the enhancer region of the *MIR122* gene, thereby inhibiting the transcription of this tumor‐suppressing miRNA and increasing H3K27 trimethylation while decreasing histone acetylation. On the other hand, the core protein interacts with DNMT1 to promote its binding to the promoters of Wnt signaling inhibitors like *DKK3*. This process leads to promoter hypermethylation and silencing of these inhibitors, which in turn activates pro‐oncogenic signals [695]. The HCV NS3 protein is also involved in multiple epigenetic regulatory pathways [696]. HCV infection leaves persistent scars on the host epigenome. Even after HCV clearance, epigenomic methylation changes in enhancer regions persist. However, these methylation marks can be reversed by combining demethylating agents with antiviral treatment [697]. HCV infections are highly adaptable and are associated with depletion of H4K20me3 levels [698]. Moreover, in vitro, the HCV core protein can induce CpG methylation profile alterations similar to those observed in patients [699].

LMP1 from EBV, HBx and core proteins from HBV and HCV, and E6/E7 from HPV are among the many tumor‐associated viral proteins that upregulate the activity of DNMTs. As a result, TSG promoters, such as that of *CDH1*, undergo methylation‐induced silencing. These viral proteins activate DNMT1 through various pathways, thus promoting tumorigenesis (Figure 14).

**Figure 14:**
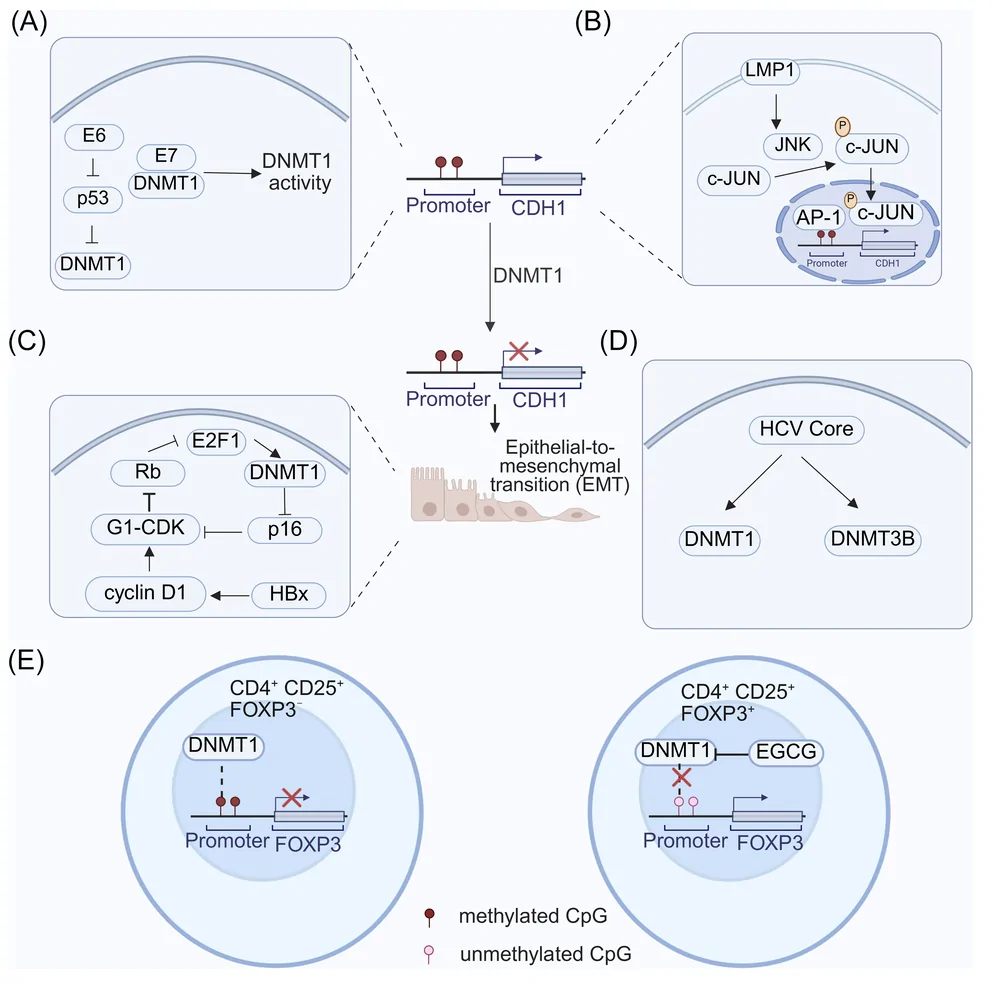
DNMT1‐mediated epigenetic repression of epithelial and immune regulatory genes. (A) HPV oncoproteins E6 and E7 upregulate *DNMT1* through the inactivation of p53, resulting in hypermethylation of the CDH1 promoter and subsequent transcriptional silencing. (B) EBV LMP1 activates the JNK/c‐JUN/AP‐1 pathway, enhancing *DNMT1*‐mediated *CDH1* repression. (C) HBV X protein (HBx) interacts with *DNMT1* and various cell cycle regulators including E2F1, p16, and cyclin D1, thereby promoting EMT. (D) HCV core protein stimulates *DNMT1* and *DNMT3B* activity, resulting in *CDH1* silencing. (E) *DNMT1*‐mediated methylation represses *FOXP3* expression in CD4^+^CD25^+^ T cells; however, treatment with EGCG can restore *FOXP3* expression, thereby highlighting a potential therapeutic strategy. DNMT1, DNA methyltransferase 1; DNMT3B, DNA methyltransferase 3B; EBV, Epstein–Barr virus; EGCG, epigallocatechin gallate; HBV, hepatitis B virus; HCV, hepatitis C virus; HPV, human papillomavirus.

#### Viral‐regulated small molecules

Viruses can indirectly regulate host epigenetics by producing small molecules or ncRNAs, thus establishing a virus‐host epigenetic regulatory network. Virus‐encoded enzymes can influence host metabolism. For example, viruses are capable of producing structural analogs of SAM that compete with DNMTs, resulting in global DNA hypomethylation. This triggers the expression of both antiviral and proviral replication genes. Moreover, virus‐encoded ncRNAs can directly modify the host's epigenetic machinery. For instance, the EBV‐encoded miRNA, such as miR‐BART11‐3p, targets chromatin remodeling factors, such as ARID1A, impairing its tumor‐suppressive function [700].

Treatment with dihydrotestosterone can enhance the transcription of EBV lytic genes by reducing the expression of DNMT3A, leading to genome‐wide DNA demethylation [701]. In the context of EBV‐associated GCa, the highly methylated enhancer‐derived miR‐129‐2‐3p upregulates crucial genes that promote the recruitment of immune cells [702]. Furthermore, the inhibition of EBV‐encoded miR‐BART6‐5p, when combined with decitabine, enhances the expression of miR‐29 in Burkitt lymphoma cell lines [703].

Chromatin remodeling by viral ncRNAs and cross‐module interactions arising from one‐carbon metabolism and the HDAC‐SIRT axis provide new connection points in the virus‐host epigenetic network.

### Chemically‐specific mechanisms

This section connects different metabolic categories through their chemical structures, aiming to show how various metabolites converge at the network level via shared enzymatic or cofactor pathways. It examines how the structural features of metabolites affect their interactions with key enzymes and cofactors, thereby imparting similar epigenetic effects across metabolite classes. By understanding the chemical specificity of these metabolites, we can identify convergent mechanisms underlying similar biological effects, such as the regulation of gene expression and tumor progression.

#### Carboxylic acids and their derivatives

SCFAs and other carboxylic acid metabolites can efficiently cross cell membranes due to their unique chemical structure. They are deprotonated at the low pH of the TME, which enables them to exert multiple epigenetic regulatory functions within the cell nucleus. The primary mechanisms include: (1) acting as direct inhibitors of HDACs, resulting in increased global histone acetylation levels; and (2) having their carboxyl groups interact with the phosphate backbone of nucleic acids, thereby loosening the nucleosome structure and increasing chromatin accessibility.

Based on this mechanism, NaB and the tea polyphenol epigallocatechin gallate (EGCG) can synergistically induce apoptosis in PDAC cells [704]. Pentanoic acid and butyrate enhance the antitumor activity of CTLs and CAR‐T cells through metabolic and epigenetic reprogramming [326]. In CRC HCT116 cells, NaB exerts antiproliferative effects by inhibiting the NRF2 pathway and demethylating the *KEAP1* promoter. TBTB demonstrates antitumor effects by altering histone acetylation through HDAC inhibition [563]. Butyrate‐containing structural lipids inhibit EMT in HCC by enhancing promoter methylation [565]. Additionally, NaB upregulates miR‐21, miR‐143, and miR‐145 in the HCT‐116 cell line [567]. 4‐PBA inhibits HCV replication by enhancing IFN‐α signaling through epigenetic modifications [705]. α‐KG promotes 5hmC modifications and upregulates PD‐L1 expression via the STAT1/3 pathway, thereby improving the efficacy of anti‐PD‐1 therapy [706]. Lastly, lactic acid activates *MYC* by increasing histone acetylation, which regulates the CSC state [606].

These metabolites coordinate with lipids and alcohols for carbon supply and HDAC inhibition, while also interacting with organic acids and amino acid metabolism through the α‐KG/TCA cycle node, thus creating a cross‐category regulatory axis governing acetylation and demethylation.

#### Amine compounds

The main amines (‐NH₂) metabolites involved in tumor epigenetics include polyamines. These compounds control tumor epigenetics in a variety of ways. One important mechanism is the competitive inhibition of MAT, also known as SAM synthetase. This inhibition prevents the formation of the methyl donor SAM, thereby restricting global DNA and histone methylation.

HDAC10 plays a crucial role in maintaining polyamine homeostasis by deacetylating N⁸‐acetylspermidine, which results in the release of free primary amines. The absence of HDAC10 has been shown to suppress tumor growth [707]. Histamine, a primary amine secreted by GSCs, activates endothelial cells via the H1 receptor, thereby promoting angiogenesis [708]. In BrCa, polyamine metabolites enhance the pro‐tumor polarization of TAMs through thymine DNA glycosylase‐mediated DNA demethylation [709]. Furthermore, overexpression of AMD1 increases spermidine production, driving the proliferation of basal‐like BrCa cells [149]. Polyamines also regulate the transcription of genes involved in cell proliferation by stimulating HAT such as GCN5 or CBP [710]. Elevated plasma levels of TMAO are associated with an increased risk of CRC [711]. It is noteworthy that the antihistamine ebastine has been identified as a new Ezh2 inhibitor that exerts anticancer effects by downregulating EZH2 protein levels and reducing H3K27me3 [712].

In tumors, primary amine metabolites like polyamines and histamine perform double duty as epigenetic regulators. They disrupt one‐carbon metabolism and consequential SAM generation, thereby affecting DNA and histone methylation. Furthermore, these compounds can also influence immune responses and the proliferation of tumor cells in complicated ways. These mechanisms are crucial features of tumor epigenetic regulation, warranting further investigation for their therapeutic targeting potential.

#### Steroids

Microorganisms, especially fungi, can oxidize a variety of sterols, including cholesterol, to form oxysterols. These molecules function as signaling ligands that activate nuclear receptors like LXR and the recently characterized L5R. Subsequently, these receptors recruit TET family DNA demethylases to gene promoters. This catalytic process oxidizes 5mC to 5hmC, potentially activating silenced pro‐inflammatory and lipid metabolism genes.

BAs are acidic organic compounds produced from cholesterol by hepatic enzymes. Alterations in BA metabolism in HCC models can drive tumor progression through epigenetic mechanisms. For instance, YAP inhibits the BA efflux gene *BSEP* by binding to TEAD transcription factors and recruiting HDAC1, thus epigenetically suppressing FXR signaling [713]. On the other hand, abnormal BA levels can hinder the recruitment of natural killer T cells and promote the polarization of M2‐like TAMs, which inhibits antitumor immunity and facilitates the progression of HCC [714]. Alcohol intake exacerbates the progression of HCC by disturbing BA homeostasis via multiple signaling pathways [615]. In addition, rationally designed paclitaxel‐phosphatidylcholine‐cholyl acid nanomicelles can strongly upregulate the TSG *SPARCL* by inhibiting DNMT activity. Subsequent promoter demethylation enhances the antitumor effects of this intervention [715].

The regulation of steroid and BA pathways can be modified epigenetically in a manner that depends on the carbon supply from lipids and SCFAs, the NAD^+^‐SIRT pathway, and the SAM cycle. These interactions establish a cross‐category network that facilitates demethylation events and regulates immune cell recruitment.

#### Heterocyclic compounds

Microbial metabolites with heterocyclic structures like pyrrole and indole act as specific regulators of epigenetic enzymes. Pyrrole derivatives like pyrrole‐2‐carboxylic acid can selectively bind to the active sites of JmjC domain‐containing demethylases such as JMJD3, inhibiting their demethylase activity by interfering with α‐KG binding to the Fe(II) cofactor. This leads to the restoration of gene silencing through the accumulation of the repressive histone mark H3K27me3 at the promoter of TSG. In addition, the nucleolar stress inducer actinomycin D can activate p53 and potentiate the action of ferroptosis inducers [716]. Azacytidine and decitabine inhibit DNA methylation by irreversibly blocking the action of DNMTs [717].

Indole and its derivatives are natural ligands of the AhR. Upon activation, the AhR/ARNT transcription complex recruits HATs, such as CBP/p300, leading to increased levels of H3K27ac and H3K9ac. Consequently, chromatin structure opens, activating the expression of target genes. Conversely, this complex can also recruit HDACs or DNMTs to some pro‐tumor genes, establishing a negative feedback loop. Research has shown that TSG activation by HDACis, such as trichostatin A, also alters the expression of pro‐ and anti‐apoptotic genes [718, 719]. In addition, by inhibiting *SLFN11* promoter methylation, these agents enhance tumor sensitivity to topoisomerase inhibitors [720]. Heterocyclic compounds interact with the α‐KG node, linking organic acid and NAD^+^ metabolism, thus forming a cross‐category demethylation‐acetylation regulatory pattern dependent on their specific chemical frameworks.

#### Polyphenols

Plant polyphenols such as quercetin, EGCG, and resveratrol (RSV), along with their microbial transformation products, can induce epigenetic regulatory effects through various mechanisms. For instance, they can activate the NAD⁺‐dependent HDAC SIRT6. This enzyme selectively removes the H3K56ac mark, an epigenetic modification associated with DNA damage repair and gene expression. By enhancing SIRT6 activity, flavonoids promote chromatin condensation at specific site, block the aberrant expression of DNA repair genes such as *BRCA1*, and decrease the transcription of proliferative genes like *c‐MYC*. These changes inhibit tumor growth and increase sensitivity to radiation and chemotherapy [721, 722, 723].

Further studies have shown that RSV regulates the interaction between MBD2 and the *BRCA1* promoter, resulting in elevated *BRCA1* expression and inhibited proliferation of BrCa cells [724]. In TNBC, RSV upregulates *CDH1* and *CDKN1A* while enhancing histone acetylation, thereby reducing cell migration [725]. In non‐melanoma skin cancer, green tea polyphenols activate TSGs by maintaining low levels of DNA methylation, which inhibits tumor growth [726]. In melanoma, fermented blueberry preparation, a polyphenol‐rich formulation, regulates miR‐200c and miR‐145 to modulate EMT, demonstrating chemopreventive potential [727]. Additionally, in BrCa, RSV can epigenetically alter the transcription of upstream oncogenes in the Hedgehog and Wnt pathways, thereby weakening core oncogenic signaling [728].

Polyphenols activate SIRTs, creating a synergistic interaction across various chemical and enzymatic pathways, including the carboxyl–NAD⁺ axis, the SAM cycle, and the HDAC inhibition module. This network‐level interaction amplifies antitumor signaling and increases sensitivity to chemotherapy.

In the previous section on chemically specific mechanisms, we focused on selected bacterial metabolites—such as SCFAs, BAs, and amino acid derivatives—and their modulation of epigenetic enzyme activity, receptor activation, and chromatin accessibility. However, within the native TME, these metabolites seldom act in isolation. Fungal metabolites, specifically secondary metabolites like aflatoxins, along with co‐metabolites produced by host‐associated microbes and fungi, have been shown to establish cross‐kingdom chemical interactions. These interactions further integrate one‐carbon metabolism, coenzyme dynamics, and immune signaling, thereby contributing to the formation of a higher‐order chemical–species–host network. This perspective proposes a transition from a source‐ or structure‐based classification toward mechanism‐oriented integration, aiming to achieve a more holistic and pathophysiologically actionable understanding of the microbial–metabolite–epigenetic interplay between specific fungi and bacteria from a co‐metabolic and interspecies perspective. This framework will be further elaborated in Section 4.5, using the regulation of host metabolic pathways by metabolites as an example.

### Microbial–host co‐metabolites

Building on the chemically specific mechanisms discussed above, this section adopts a more integrated view of microbe–host co‐metabolism by examining bacterial and fungal metabolites alongside host‐derived metabolites. This comparative framework highlights their cooperative and opposing roles in one‐carbon metabolism, supply of cofactor and substrate, and the joint regulation of immune and epigenetic processes.

Microorganisms and their hosts cooperate to achieve mutual regulation through shared metabolites. These co‐metabolites affect epigenetic regulation, thereby contributing to tumor initiation and progression or modulating immune responses. The next sections will focus on the relationship between gut microbiota and host liver/immune cells, and how these metabolites affect the TME through epigenetic mechanisms.

#### Gut microbiota‐liver metabolic axis

The gut microbiota and liver are closely linked through a co‐metabolic axis centered on BA metabolism, which significantly influences tumor epigenetics. Gut microbiota convert primary BAs into secondary BAs (e.g., DCA). Upon entering the liver via the portal vein, these secondary BAs activate receptors such as the FXR and TGR5 to regulate energy metabolism and directly inhibit HDAC activity. This inhibition leads to increased accumulation of H3K9ac and H3K27ac, thereby activating genes involved in energy utilization and programmed cell death.

The secondary BA, DCA, produced by the microbiota, can directly activate the mTOR pathway in hepatocytes, thereby promoting the progression of HCC driven by non‐alcoholic steatohepatitis [729]. Meanwhile, butyrate, a SCFA derived from gut microbial fermentation of dietary fibers, serves as an essential carbon source for histone acetylation in the host. Following colitis, levels of butyrate and the corresponding histone acetylation are reduced [730]. Conversely, clinical studies indicate that the serum of HCC patients exhibits a significantly lower ratio of secondary BAs. The reduction of secondary BAs caused by broad‐spectrum antibiotics, which worsens HCC, can be reversed by supplementation with glycochenodeoxycholic acid, resulting in a decreased tumor burden [731].

This metabolic axis links sterols (e.g., BAs) with carboxylic acids (e.g., SCFAs) and other organic acids (e.g., lactate and dihydroxycamphor precursors). It modulates HDAC activity, nuclear receptor signaling, and immune cell metabolic reprogramming, thus establishing a tripartite “metabolism–receptor–chromatin” regulatory module at the epigenetic level. The SAM/NAD⁺ hub that interconnects these pathways is further modulated by upstream alcohol and amino acid metabolism. Furthermore, these metabolites reciprocally reshape the gut microbiota structure, forming a complex network with both positive and negative feedback loops.

#### Microbiota‐T cell interaction

The gut microbiota promotes dendritic cells to secrete cytokines like IL‐6 and IL‐23 by means of indole derivatives, bacterial cell wall constituents, and other signaling molecules. These cytokines facilitate the differentiation of naïve CD4^+^ T cells into Th17 cells [732]. After differentiation and maturation, Th17 cells subsequently secrete a great deal of inflammatory cytokines, such as IL‐17A and IL‐22. The enrichment of H3K27ac and activation of inflammatory genes can be achieved through cytokine‐mediated activation or recruitment of HAT p300/CBP within the TME [733]. Additionally, IL‐17A can activate TAMs to induce TET2 expression. This facilitates the conversion of DNA 5mC sites to 5hmC, maintaining an open chromatin state at inflammatory gene loci.

Moreover, SCFAs such as butyrate can enhance anti‐tumor immunity by modulating the epigenetic state of CD8^+^ T cells. Butyrate inhibits IFN‐γ‐induced upregulation of PD‐L1 in CRC cells and promotes the acetylation of STAT1 at lysine residues. Furthermore, butyrate increases the enrichment of H3K27ac in the promoter regions of *PDCD1* and *CD28* in CD8^+^ T cells, leading to upregulation of PD‐1 and CD28 expression and thereby enhancing the efficacy of anti‐PD‐1 therapy [734]. Additionally, butyrate‐treated MDSCs can restore their immunosuppressive functions [735]. SCFAs also reprogram the metabolism and epigenomics of CTLs and CAR‐T cells by enhancing mTOR activity and inhibiting class I HDACs, which significantly boosts anti‐tumor activity [326]. These findings illustrate the multi‐layered epigenetic feedback network formed by Th17 cytokines and microbial metabolites, which profoundly influences the tumor immune microenvironment and the response to immunotherapy.

The microbiota‐T cell interaction node integrates various classes of signals, including indoles (heterocyclic compounds), carboxylic acids, amino acid derivatives, and organic acids. These metabolites exert synergistic amplification or cross‐regulation through shared transcription factors (such as STAT and NF‐κB) and epigenetic enzymes (including HAT/HDAC and TET/DNMT), thereby synchronizing immune responses with metabolic‐epigenetic states.

In summary, microbial metabolites from various chemical categories do not operate in isolation on tumor epigenetics. Instead, they establish a highly interconnected regulatory network centered on core co‐factor hubs and signaling nodes, including acetyl‐CoA, SAM/SAH ratios, NAD^+^/NADH, α‐KG/TCA cycle intermediates, and GSH/ROS balance. This network encompasses synergistic interactions in which compounds like SCFAs, alcohols, polyphenols, and sterols/BAs act as SIRT activators and HDAC inhibitors. Lactate along with SCFAs offer carbon and may exhibit either synergistic or competitive relationships in specific TMEs [736]. Regulatory mechanisms further involve one‐carbon metabolism, affecting amino acids, B‐vitamins, and polyamine‐linked amines, together with SAM‐related fungal and viral factors, which cooperatively regulate DNMTs. This creates complex cross‐class interactions, where even small changes in the SAM/SAH ratio can be greatly amplified by many metabolic inputs [737]. Additional mechanisms involve the NAD⁺/SIRT pathway, influenced by nucleotides, sterols, vitamins, and organic acids, which interacts with α‐KG‐dependent TET and JmjC‐domain enzyme families at both substrate and inhibitor levels. Immune‐epigenetic coupling arises when metabolites such as butyrate, indoles, TCA intermediates, organic acids, and sterols converge at signaling nodes—including STAT, NF‐κB, and p300/CBP/TET—to regulate the functional states of immune effectors such as CTLs, Tregs, Th17 cells, MDSCs, and non‐immune cells [165]. Redox‐chromatin homeostasis reflects the dynamic equilibrium among GSH, ROS, FAD/NAD^+,^ and the acetylation–deacetylation and demethylation–methylation cycles, which are intricately interconnected. Multiple metabolite classes can induce epigenetic reprogramming, particularly under conditions of oxidative stress.

Therefore, understanding how these cross‐class metabolites synergistically or antagonistically influence tumor epigenetics—through shared substrates, competitive enzyme binding, and co‐regulated transcription‐epigenetic nodes—is crucial for revealing the systems‑level dynamics of tumor initiation and progression. This insight is equally essential for designing multi‐pathway‐targeted combination therapies.

## EPIGENETIC FEEDBACK REGULATION OF TUMOR ON MICROORGANISMS AND METABOLISM

### Epigenetically driven regulation of host metabolic enzymes

Epigenetic mechanisms, including DNA methylation, histone modifications, and ncRNA regulation, play a pivotal role in modulating the expression and activity of key metabolic enzymes. These regulatory events allow tumor cells to adapt their metabolic programs to a dynamic microenvironment. Table 14 summarizes key metabolic enzymes regulated by these epigenetic mechanisms and their roles in cancer progression.

**Table 14:** Regulated metabolic enzymes in cancer.

Regulated metabolic enzymes	Mechanism of action	Role in tumors	Related tumor types	References
CYP27C1	Involvement in regulating the IGF‐1R/Akt/p53 signaling pathway is crucial in determining cellular sensitivity to various anticancer drugs, such as vincristine and picropodophyllin. The expression level of this pathway plays a significant role in modulating the effectiveness of these therapeutic agents	Inhibits tumor progression. Its low expression enhances proliferation, clonal formation, migration, and chemoresistance in LuCa cells	LuCa	[738]
hexokinase 2 (HK2)	Catalyzes the initial step of glucose metabolism, known as the committed step. Its deletion inhibits the production of glucose‐derived nucleotides and impairs glutamine carbon utilization in the salvage pathway	Essential for tumor initiation and maintenance, the continued expression of HK1 is insufficient to sustain the neoplastic phenotype of cancer cells when HK2 is deleted	LuCa, BrCa	[739]
LDHA	Catalyzes the final step of glycolysis, the conversion of pyruvate to lactate, a crucial process that exemplifies the Warburg effect, a hallmark of aerobic glycolysis	Plays the role of an oncogene. Its expression promotes PDAC cell growth, whereas knockdown significantly inhibits cell growth and tumorigenicity in vivo by activating apoptotic pathways	PDAC	[740]
GAPDH	Methylation modification at the R206 site enhances its function and inhibits mitochondrial activity	Promotes glycolysis, proliferation, and tumorigenesis, serving as a poor prognostic marker	CRC	[741]
G6PD	Activates the STAT3 signaling pathway and regulates the cellular redox state	Promotes proliferation, migration, and tumorigenesis in vivo, serving as a poor prognostic marker	LuCa	[742]
IDH1	The mutant catalyzes the production of R‐2HG, activating the miR‐182‐5p/CDKN2C signaling axis	Mutation enhances proliferation and inhibits apoptosis, driving tumorigenesis		[743]
ATP‐Citrate Lyase	Catalyzes the production of acetyl‐CoA, providing precursors for lipid synthesis	Silencing induces cell cycle arrest and apoptosis, dependent on lipid depletion conditions	Found in Multiple Cancers	[744]
FASN	Key enzyme catalyzing de novo lipogenesis	Promotes proliferation and metastasis via the mTOR/Gli1 pathway	GCa	[745]
Glutaminase 1 (GLS1)	Catalyzes glutamine metabolism, activating the AKT/GSK3β pathway	Promotes proliferation and is associated with poor prognosis in HCC cells	HCC	[746]
Fructose‐1,6‐Bisphosphatase A	Catalyzes glycolysis and inhibits AMPK, activating the YAP signaling pathway	Promotes proliferation and migration in CRC and is associated with poor prognosis	CRC	[747]
PFK‐2	Activates glycolysis and regulates fructose‐2,6‐bisphosphate levels	Promotes proliferation and cell cycle progression in KCa and is associated with poor prognosis	KCa	[748]
Acetyl‐CoA synthetase 2	Catalyzes the conversion of acetate to acetyl‐CoA	Provides carbon sources under hypoxic or lipid depletion conditions, promoting tumor growth	BrCa	[749]
3‐Phosphoglycerate Dehydrogenase	Catalyzes the first step of serine biosynthesis	Promotes tumor growth and progression in serine‐restricted environments	Melanoma, BrCa	[750]
SCD1	Catalyzes the synthesis of unsaturated fatty acids (rate‐limiting enzyme)	Promotes proliferation and invasion in LuCa and is associated with poor prognosis	LuCa	[751]
Enolase 1	Catalyzes glycolysis and functions as a plasminogen receptor, among other roles	Overexpressed in various cancers, promoting proliferation, migration, and poor prognosis	CRC, LuCa	[752]
6PGD	Transcriptionally regulated by TAp73, catalyzes the production of Ru5P and NADPH	Supports proliferation, antioxidant defense, and anti‐apoptosis, promoting tumorigenic growth	Found in Various Cancers	[753]
Citrate synthase	Catalyzes the first step of the citric acid cycle	Promotes proliferation, migration, and invasion	PCa	[754]
PSAT1	Involved in serine metabolism reprogramming	Promotes metastasis and induces treatment resistance	KCa	[755]
SHMT2	Catalyzes the rate‐limiting step of serine degradation and is regulated by desuccinylation	Drives cell proliferation and adapts to metabolic stress	Various Cancers	[756]
BCAT1	Catalyzes BCAA catabolism and is regulated by IDH/α‐KG	Promotes cell proliferation, invasion, and tumor growth	Glioma	[757]
NAMPT	Catalyzes the rate‐limiting step of the NAD⁺ salvage pathway	Promotes proliferation and migration via the Wnt/β‐catenin pathway	CRC	[758]

Abbreviations: GLS1, glutaminase 1; HK2, hexokinase 2.

#### DNA methylation–mediated metabolic reprogramming

DNA methylation is a stable, heritable epigenetic alteration that affects the expression of several metabolism‐related genes. It not only plays an initiating role in tumorigenesis but also exerts dynamic effects throughout tumor progression and therapeutic resistance, spanning the continuum from premalignant lesions to acquired drug resistance.

During initial tumor development, DNA methylation activates metabolic genes to facilitate the reprogramming required to meet the cellular demands of a growing tumor. Intratumoral microbes generate metabolites, including butyrate and DCA, which alter the availability of SAM, leading to systemic, genome‐wide shifts in DNA methylation. Promoter methylation changes of key metabolic enzyme‐encoding genes—including Hexokinase 2 (*HK2*), *PFK*, pyruvate kinase M2 (*PKM2*), fatty acid synthase (*FASN*), acetyl‐CoA carboxylase (*ACC*), and glutaminase 1 (*GLS1*)—regulate glycolytic flux, lipid synthesis, and Gln anaplerosis. Mutually, these pathways generate the energy and molecular building blocks necessary to sustain tumor growth [759]. Furthermore, CGIs near the binding sites of ESR1, FOXA1, and GATA3 are enriched with hypomethylation in early‐stage BrCa, enhancing the activation of these metabolic pathways to enable tumor initiation [760, 761, 762].

The regulatory effects of DNA methylation promote tumor proliferation and metastasis. In ER⁺ BrCa, ZEB1‐induced depletion of the *MIR497HG* lncRNA abolishes the inhibitory effects of miR‐497 and miR‐195 on the PI3K–AKT pathway, leading to tamoxifen resistance [763]. In thyroid cancer, increased methylation of the *ATF3* promoter impairs its tumor‐suppressive function and activates downstream oncogenes [764]. In HCC, *LDHB* silencing mediated by DNMT3A alters the immune microenvironment to promote disease progression [765, 766]. Collectively, these mechanisms enhance the invasiveness and therapy resistance of tumor cells.

DNA methylation is a central driver of drug resistance in tumor cells. Essentially, tumor cells depend on DNA methylation to adapt under therapeutic pressure and develop tolerance to chemotherapy and targeted agents. For instance, in HCC, tumor proliferation and metastasis depend on a ROS–FOXC1–cysteine metabolism–ROS positive feedback loop, which represents a potential therapeutic target [767]. Ongoing DNA methylation changes at this stage help tumor cells evade apoptosis and withstand drug stress, thereby reinforcing the resistant phenotype.

#### Histone modification‐mediated regulation of metabolic pathways

Unlike DNA methylation, which exerts a binary on–off effect, histone modifications provide a more dynamic and finely tuned regulatory mechanism, functioning akin to a “dimmer switch”. This allows metabolism‐related genes to adjust their transcription rapidly in response to both intracellular and extracellular signals, particularly those derived from metabolites of mixed origins.

During early tumorigenesis, histone modifications remodel chromatin structure to fine‐tune metabolic gene transcription and facilitate rapid cell proliferation. Microbial metabolites including SCFAs act as HDACis, increasing H3K9ac and H3K27ac levels. These modifications enhance the expression of genes governing glycolysis and lipogenesis [768, 769, 770], thereby providing tumor cells with the metabolic carbon source for early tumor growth.

Tumor growth is sustained by metabolic and epigenetic alterations that promote invasive behaviors. The activation of glycolytic pathways by ZEB1 drives tumor evolution toward a more aggressive neuroendocrine lineage in PCa. Loss of the SWI/SNF chromatin‑remodeling complex subunit ARID1A in LuCa increases cellular dependence on hyperactive glycolysis [771]. Through such processes, tumor cells can rapidly adapt to changing metabolic demands using during disease progression.

Histone modifications play key roles in maintaining the activity of essential metabolic pathways, enabling tumor cells to escape therapy and survive during the drug‐resistant stage. For instance, FASN is regulated by multiple signaling pathways that promote tumor cell proliferation and invasiveness, which are crucial for the development of therapeutic resistance [772, 773, 774, 775]. The continuation of such epigenetic regulation facilitates tumor cells survival during drug‐resistant phases.

#### ncRNA‐mediated regulation of enzyme activity

The ncRNA network provides an additional layer of fast and precise metabolic regulation at the post‐transcriptional level. This is especially important for tumor cells coping with acute stresses, such as those induced by therapeutic agents. For example, miRNA binding to the 3′‐untranslated region of target genes causes mRNA degradation or translational repression.

During early tumorigenesis, ncRNAs tightly regulate the expression of metabolism‐related genes through post‐transcriptional modifications, enabling tumor cells to adapt to environmental stress. For example, research found that miR‐96‐3p targets *SDHB*, which modulates the AKT/mTOR pathway and enhances metastasis in papillary thyroid carcinoma [776]. Likewise, in CRC, *SDHB* deficiency is essential for the oncogenic activity of miR‐142‐5p [777]. These ncRNA‐mediated pathway alterations modify metabolic flux to support the growth of nascent tumors.

During tumor progression, ncRNAs stabilize the activity of metabolic pathways, supporting tumor growth and proliferation. For instance, the ncRNA LINC01852 regulates alternative splicing of *PKM*, which represses CRC malignant transformation and chemoresistance [778]. By guiding tumor cells to optimize their metabolic activity, ncRNAs enable them to adapt to the continuously evolving TME.

#### Chromatin remodeling‐mediated regulation of metabolic pathways

Chromatin remodeling is the executive mechanism of epigenetic regulation. It determines the accessibility of transcription factors to regulatory elements of metabolic enzyme genes by directly changing DNA‑chromatin architecture. This physical layer of regulation guides essential phenotypic changes throughout tumor development.

Chromatin remodeling complexes regulate nucleosome positioning and chromatin accessibility, thereby directly controlling the transcription of metabolic enzyme genes. In PCa cells, Zeb1 binds to and activates the promoters of various glycolytic enzymes, enhancing glycolysis and driving these cells toward a more aggressive neuroendocrine lineage, illustrating its role in tumor progression and lineage plasticity. The ARID1A subunit of the SWI/SNF complex crucially affects tumor dependence on metabolic pathways. Loss of ARID1A renders LuCa cells reliant on hyperactive glycolysis, and inhibition of the SWI/SNF complex suppresses the growth of ARID1A‐deficient LuCa [771]. Furthermore, the Lyn/RUVBL1 complex enhances the metabolism of arachidonic acid and induces expressions of MMP9 and VEGF in a β‐catenin‐dependent way, facilitating CRC metastasis [779].

Therapeutic resistance is likewise influenced by chromatin remodelers. The ATP‐dependent remodeling factor BRG1 (SMARCA4) enhances HCC mitochondrial function by modulating TOMM40. It also drives the progression of precancerous pancreatic lesions in conjunction with SOX9, inferring that the BRG1‐associated axis may be therapeutic targets [780, 781]. In basal‐like BrCa, the transcription of *HK2* gets directly activated by the SOX4–SMARCA4 complex, which, in turn, sustains glycolysis and proliferation [782].

Together, these mechanisms highlight that chromatin remodelers act as core mediators, integrating upstream signals with metabolic pathways to enable tumors to adjust to the demands of different developmental and therapeutic stages.

#### PTMs in the regulation of metabolic enzyme activity

Detachment of an amino acid or peptide from its macromolecule, which is important for its metabolic function, is known as a PTM. Tumor cells must respond rapidly and with high fidelity to different internal and external signals, including microbial metabolite cues, necessitating such dynamic regulatory mechanisms.

During the early stages of tumorigenesis, PTMs regulate the activity of key metabolic enzymes to promote metabolic adaptation. For instance, O‐GlcNAc glycosylation of phosphoglycerate kinase 1 (PGK1) coordinates glycolysis with the TCA cycle. Inhibition of this modification significantly inhibits CRC cell proliferation [783]. These PTMs thus confer the metabolic flexibility necessary for tumor cell adaptation.

Mutations in genes encoding PTM‐remodeling enzymes also occur during tumor progression and lead to cancer‐causing alterations in the protein targets. When oxygen levels are lower than normal or when certain genes are activated, PGK1 gets a change that enables its mitochondrial translocation, where it reprograms cellular energy metabolism to promote tumorigenesis [784]. Acetylation of ACLY leads to a reprogramming of lipid metabolism and the activation of the cGAS–STING pathway, overcoming resistance to anti‐PD‐L1 therapy [785]. In LuCa with coexisting KRAS and LKB1 mutations, autophagy‐derived acetyl‐CoA enhances acetylation of the Snail transcription factor, promoting metastatic capacity [786]. In HCC, acetylation of ACLY at lysine 468, mediated by NAT10, has been identified as a novel driver of chemoresistance [787]. Essentially, these PTM‑driven mechanisms illustrate diverse strategies utilized across cancer types.

Tumor cells are capable of flexibly re‐programming metabolic pathways according to growth demands as well as environmental variations through multiple regulatory layers, including DNA methylation, histone modifications, ncRNAs, and PTMs. Microbial metabolites play a significant role in this process by influencing cancer development across its distinct phases—early, progressive, and therapy‐resistant. By modulating these phases, microbial metabolites represent promising novel therapeutic targets and provide valuable insights into the mechanisms of carcinogenesis.

### Tumor epigenetic‐driven remodeling of the TME and microbial metabolism

#### Hypoxia‐induced epigenetic–metabolic coupling

The progression of cancer is strongly driven by the activation of the transcription factor HIF‐1α in the hypoxic TME. In response to hypoxia, the activation of various metabolic pathways and epigenetic reprogramming results in the buildup of metabolic intermediates, thereby forming an integrated hypoxia–epigenetics–metabolism–microbiota coupling network.

Lactate, once dismissed as just a waste product, is now an important signal. It regulates the expression of genes related to glycolysis by lactylation. The development of such adaptive state leads to the accumulation of lactate, which in turn enhances histone lactylation, creating a positive feedback loop that promotes glycolysis and membrane lipid biosynthesis [788]. Similarly, α‐KG‐dependent demethylases, such as the TET and JmjC domain‐containing families, are competitively inhibited by succinate and fumarate. This inhibition leads to a hypermethylated genomic state that enhances the survival and invasion of tumor cells [691]. Moreover, microorganisms located in hypoxic areas produce high levels of SCFAs because of the tumor microbiota influence. These metabolites may inhibit HDACs or serve as acetyl donors, dynamically modulating epigenetic marks and influencing tumor metabolic adaptation [789].

The role of lactate extends beyond tumor cell metabolism. Lactate stimulates M2‐like macrophage polarization via the PKA/CREB signaling pathway, which contributes to immune evasion and tumor progression. This lactate‐mediated process is an important part of the hypoxia–lactate–immune evasion phenomenon, allowing tumor cells to survive and become more resistant to chemotherapy. HIF‐2α sustains BrCa stemness via the SOD2–mtROS pathway, and its inhibition has been shown to restore chemosensitivity [790]. Lactylation of APOC2 enhances its protein stability, promoting Treg recruitment and resistance to immunotherapy [20].

#### Regulation by the acidic microenvironment

The epigenetic landscape is modified by acidic TME, which ultimately alters metabolism genes transcription. This change improves tumor invasion and gives a proliferative advantage to acid‐resistant microbial types. The resulting shifts in microbial community composition and metabolite profiles act synergistically to promote tumor invasion.

Accumulated lactate enhances the activation of histone marks, including H3K9ac and H3K18la, thereby accelerating glycolytic flux and LDHA activity. This allows tumor cells to grow under low‐pH conditions and increases invasive capacity. At the same time, selective pressure from acidic microenvironment favors the expansion of acid‐resistant bacteria like *Bacteroides* and *Lactobacillus*. These microorganisms adapt to acidic conditions through mechanisms such as activation of proton pumps and resistance to F_0_F_1_‐ATPase inhibition [791].

In the context of NSCLC, lactate production is regulated by the miR‐7‐5p/*c‐MYC*/LDHA axis. Targeting LDHA lowers lactate production and inhibits metastasis [792]. In CRC models, acidic conditions combined with vitamin D receptor overexpression result in the downregulation of SOX2, which inhibits cancer stemness [793]. In summary, acidic stress could promote invasion and metastasis of tumors via epigenetic regulation of metabolic pathways. Tumor metabolic reprogramming requires coordinated enhancement of glycolysis, pentose phosphate pathway, TCA cycle, and fatty acid metabolism, as well as integration of transcription factor networks through signaling axes, such as ROS, HIF‐1α, and PI3K/AKT/mTOR. These mechanisms promote the aberrant activity of metabolism‐related genes and speed up cancer progression (Figure 15).

**Figure 15:**
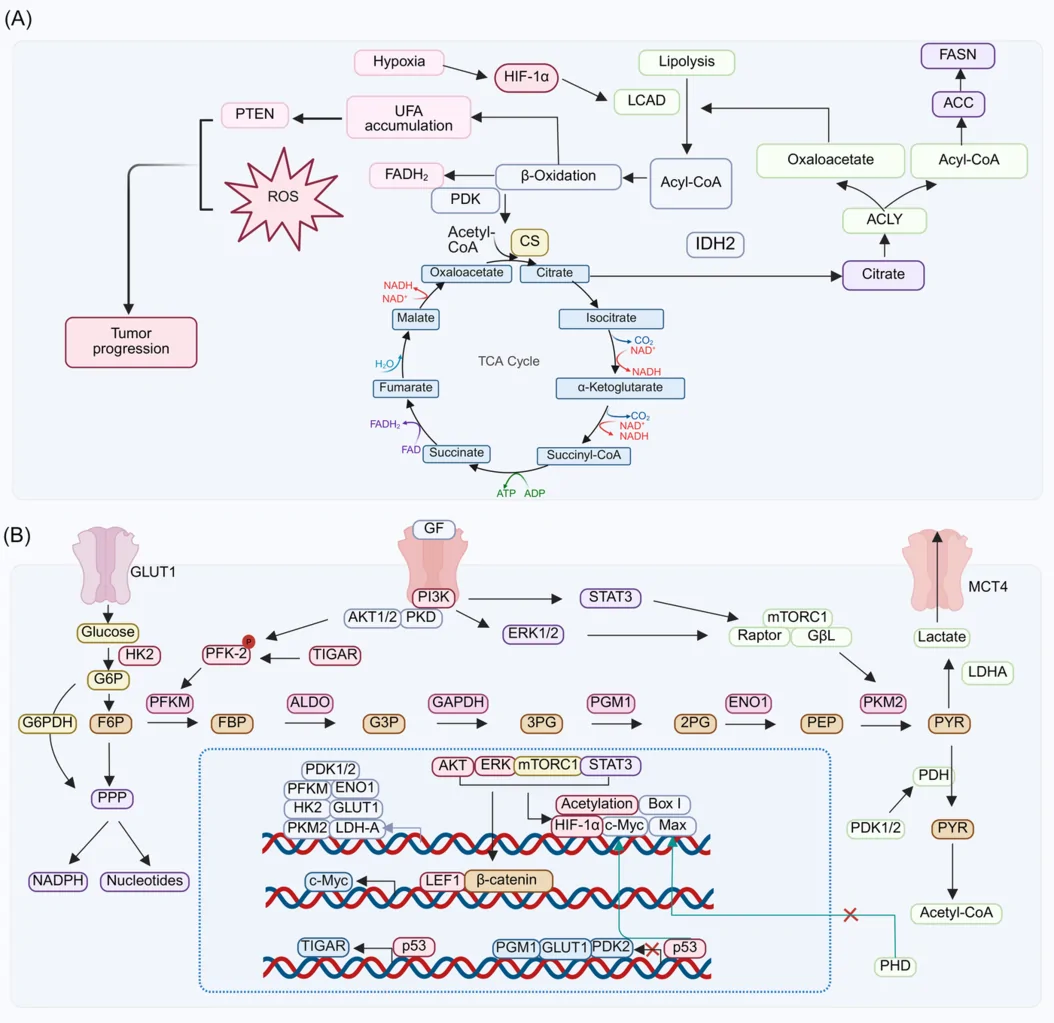
Cancer cell metabolic reprogramming: synergistic regulation of the glycolysis–PPP, TCA cycle, and fatty acid metabolism through signaling and transcription factor networks. (A) ROS and hypoxia induce alterations in fatty acid β‐oxidation and TCA cycle flux, activating HIF‐1α and downstream metabolic nodes, including ACL, ACC, and FASN, thereby sustaining tumor progression. (B) Enhanced glycolysis via GLUT1‐mediated uptake and lactate export through MCT4 converges with PI3K/AKT, ERK, and mTOR signaling cascades to reinforce transcriptional programs. Transcription factors, including HIF‐1α, c‐Myc, and β‐catenin, orchestrate the expression of metabolic enzymes (e.g., HK2, PKM2, and LDHA), creating a feed‐forward loop linking metabolic reprogramming to tumor growth and survival. Box I highlights a transcriptional regulatory module integrating oncogenic signaling pathways with transcription factors to control glycolytic gene expression. ACC, acetyl‐CoA carboxylase; FASN, fatty acid synthase; HIF‐1α, hypoxia‐inducible factor 1α; HK2, hexokinase 2; LDHA, lactate dehydrogenase A; PKM2, pyruvate kinase M2; PPP, pentose phosphate pathway.

#### Immunometabolic interactions and epigenetic regulation by microbial metabolites

To escape the immune response of the host, tumor cells upregulate the immune checkpoint molecules such as PD‐L1 and CTLA‐4 by the use of epigenetic modifications. Microbial metabolites help regulate the immune system functions. One example is that butyrate, which is a SCFA, inhibits the activity of HDAC, leading to enhanced PD‐1 expression in T cells due to increased H3K27 acetylation within the *PDCD1* promoter region. The phenomenon may enhance the exhaustion of T‐cells and evade the immune system [42]. The modulatory effect of tryptophan‐derived metabolites, particularly indole derivatives, on checkpoint expression has been demonstrated to be mediated by the AhR pathway through the reduction of histone methylation at the inhibitory receptors CTLA‐4 and LAG‐3. This process restores T cell effector functions [794]. Secondary BAs also lower PD‐1 expression in T cells and boost anti‐tumor immunity by targeting FXR/TGR5 signaling, which reduces DNA methylation at the *PDCD1* gene locus [795, 796]. Microbial metabolites directly affect the tumor immune response through complex epigenetic regulation. Studies have revealed that metabolites in the TME remodel immune checkpoint expression through epigenetic alterations, shaping the immune landscape and impacting the immunotherapy efficacy.

Ultimately, the native epigenetic state of tumor cells, the metabolic programming of immune cells, and the epigenetic modulation of immune checkpoints by microbial metabolites are integrated in a complex, dynamic, and mutually regulatory network of “epigenetics–metabolism–immunity”. The efficacy of immunotherapeutic interventions largely depends on this network.

### Epigenetic regulation of microbiota composition by tumors

#### Metabolite‐mediated niche remodeling

Niche remodeling refers to the process by which organisms (e.g., microbes or host cells) begin to change their ecological position and functional state in a given environment in response to metabolic, gene expression, or behavioral changes. This remodeling, driven by metabolite production and environmental fluctuations, reshapes interspecies interactions and the dynamic balance of the system. In the TME, tumor cells influence microbial distribution, function, and metabolism through metabolic reprogramming, immune modulation, and ecological alteration mechanisms. Tumor growth, invasion, and response to therapy are affected by these processes. Furthermore, these metabolites maintain their influence over the metabolic niches at every phase of tumor initiation, progression, and treatment.

During antitumor immunotherapy, pectin enhances the efficacy of anti‐PD‐1 therapy in CRC by regulating the gut microbial production of butyrate [28], highlighting how microbially derived metabolites can reshape the immune metabolic niche. In GBM, SCFAs stimulate glycolysis in TAMs, promoting their polarization toward the M1 phenotype and underscoring the therapeutic potential of the “gut–brain axis” in TAM‐targeted immunotherapy [797]. From a chemosensitization perspective, the oxaliplatin (Oxa)@HMI nanoplatform—a nanotechnology‐based drug delivery strategy that integrates Oxa with a molecularly imprinted polymer nanocarrier system—enhances the efficacy of chemotherapy in CRC by reshaping the tumor microbiota and activating antitumor immunity [798]. These findings further demonstrate that modulation of microbial composition and its metabolic outputs contributes to metabolic‐mediated niche remodeling.

These results show a closed‐loop regulatory network encompassing tumor cells, epigenetics, metabolism, and microbiota. Tumor cells undergo epigenetic changes that alter metabolic programs, leading to the secretion of metabolites that further change the TME and the associated microbial communities. The detrimental interplay creates a negative feedback loop that is difficult to break (Figure 16).

**Figure 16:**
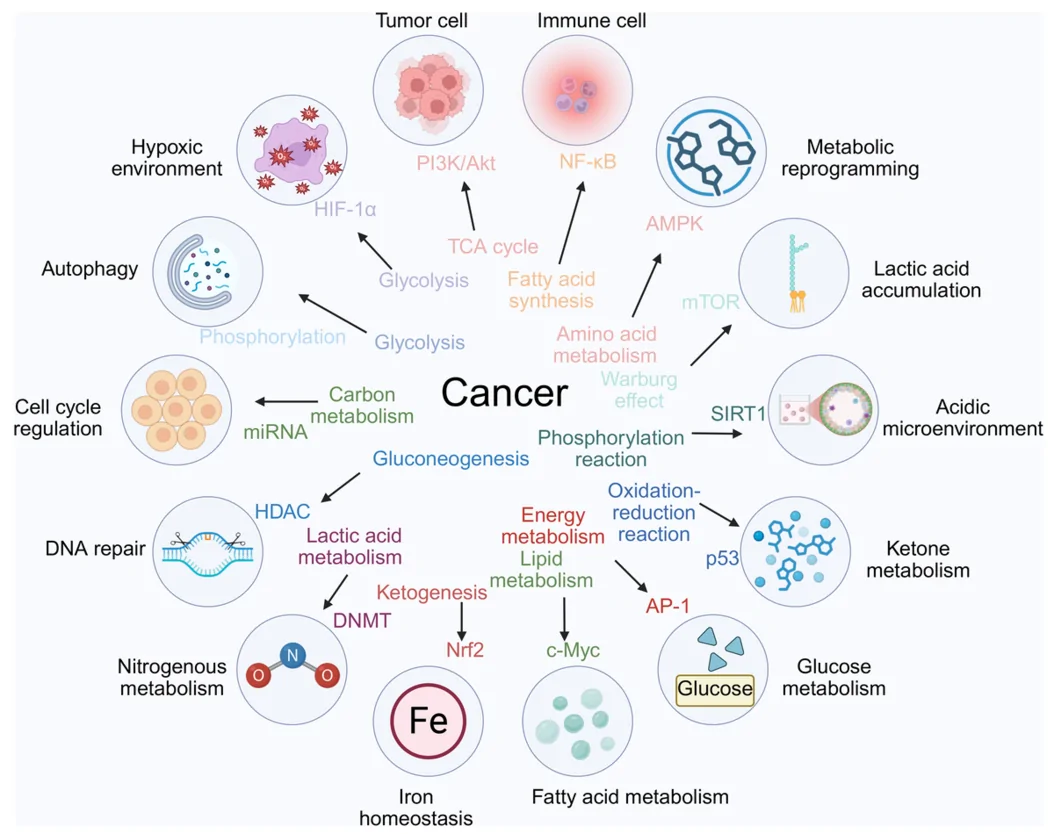
Integrated network of cancer metabolism, epigenetic regulation, and microenvironmental adaptation. Cancer progression is tightly regulated by a complex network that integrates metabolic pathways, epigenetic regulation, and responses to microenvironmental stress. Tumor and immune cells interact through reprogrammed pathways, including glycolysis, TCA cycle, fatty acid metabolism, ketone body synthesis, and amino acid metabolism. These pathways modulate chromatin modifiers such as HDACs, DNMTs, SIRT1, as well as transcription factors including HIF‐1α, NF‐κB, c‐Myc, AP‐1, Nrf2. This modulation influences critical cellular processes, including cell cycle regulation, DNA repair, autophagy, and immune adaptation. Additionally, the accumulation of lactic acid, alterations in iron metabolism, and the acidic TME further reinforce malignant phenotypes, establishing a feed‐forward loop of “metabolism–epigenetics–cancer” that sustains tumor growth and contributes to therapy resistance. DNMTs, DNA methyltransferases; SIRT1, sirtuin 1.

#### Epigenetic–immune axis in microbiome regulation

The immune microenvironment created by tumor epigenetic reprogramming plays a vital role in the microbial composition. A notable example is the formation of NETs. Blocking the *TGM2* gene or the serotonin transporter reduces histone modifications such as H3Q5ser and H3cit, which in turn leads to reduced NET formation and hepatic metastasis in different cancers [322].

The novel tripartite mechanism called ‘epigenetics–immunity–microbiota’ proposes an exciting approach for reprogramming the tumor‐associated microbiome by epigenetic or immune manipulation. Specifically, host epigenetic machinery acts as a “gatekeeper,” modulating the infiltration of immune cells that serve as primary sensors and regulators of the microbial community. For instance, the transcription factor EHF controls the migration of CXCR2‐positive neutrophils through the CXCL1‐CXCR2 axis. Significantly, the depletion of such neutrophils increases PDAC sensitivity to chemotherapy and immunotherapy [799]. This epigenetic regulation is critical because neutrophils are key effectors in shaping the spatial distribution and inflammatory response of the tumor microbiota. Similarly, in papillary thyroid carcinoma, METTL3 collaborates with YTHDF2 to modulate tumor‐associated neutrophil infiltration through the NF‐κB pathway [800]. This suggests that m^6^A RNA methylation dynamically reshapes the immune landscape, thereby creating a permissive or restrictive niche for specific microbial colonization. These examples illustrate how epigenetic alterations on the host side can indirectly but profoundly govern the rules of engagement for the tumor microbiome.

The gut microbiota affects the TME both locally, through the production of metabolites, and systemically, via epigenetic and immune signaling. Growing evidence supports this conclusion. Microbial metabolites can affect the immune microenvironment by directly influencing chromatin modifications and gene transcription. Plus, these metabolites may enter systemic circulation via the portal vein and BA enterohepatic circulation, resulting in disturbed metabolism–immunity homeostasis in the liver and throughout the organism (Figure 17).

**Figure 17:**
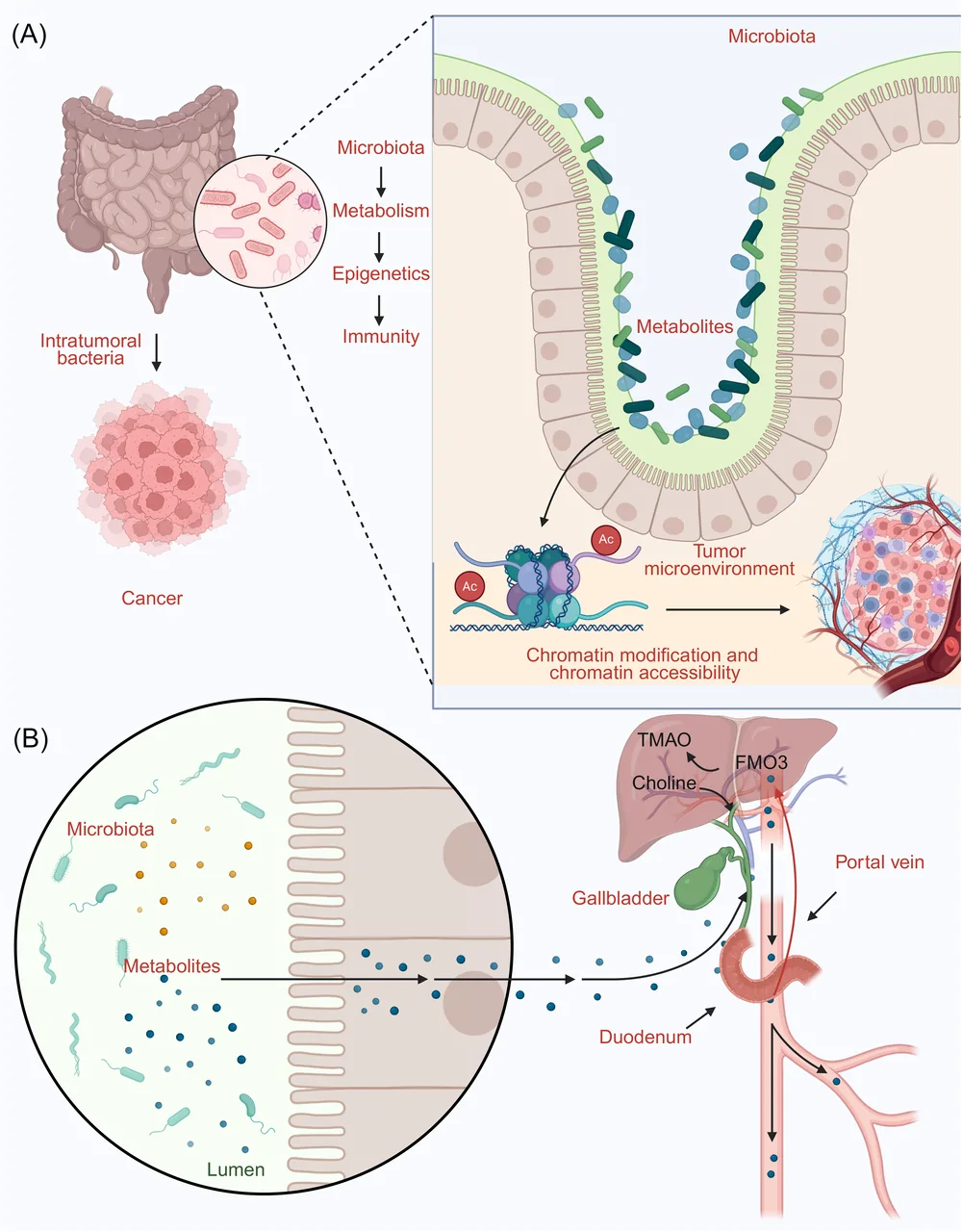
Intestinal microbiota affect the TME and systemic metabolic–immune regulation. (A) Microbiota‐derived metabolites modulate chromatin accessibility and epigenetic states within the TME, thereby linking microbial metabolism to immune regulation and cancer progression. Intratumoral bacteria and intestinal microbiota collaborate with host cells through metabolic and epigenetic pathways, thereby shaping tumor immunity. (B) Microbial metabolites produced in the gut, including TMAO and choline‐derived intermediates, enter the bloodstream through the portal vein and biliary system, thereby influencing systemic metabolism and immune responses beyond the intestinal tract. Ac, acetylation; FMO3, flavin‐containing monooxygenase 3; TME, tumor microenvironment.

#### Microbial epigenetic adaptation

In the TME's selective pressure, colonizing microbes actively improve their ability to survive and colonize via epigenetic mechanisms rather than passively adapting. This allows the microbes to build a strong symbiosis with the tumor cells. In response to acidic conditions, oxidative stress, and drug exposure, tumor cells release signals and metabolite via epigenetic reprogramming that causes epigenetic adaptation in the resident microbiota. For instance, the m^6^A methylation induced by *Helicobacter pylori* activates caspase‐8, which improves GCa sensitivity to 5‐FU [801]. Histone H3K18 lactylation promotes immune evasion in NSCLC through the POM121/*MYC*/PD‐L1 axis [802]. The loss of ASF1A facilitates M1 macrophage polarization and T cell activation, synergizing with anti‐PD‐1 therapy [803]. SATB2‐driven neural crest–mesenchymal programs enhance melanoma proliferation and therapeutic resistance [804]. Inhibition of glycolysis and B7‐H3 expression reactivates tumor‐infiltrating CD8^+^ T cells, strengthening the efficacy of anti‐PD‐1 therapy [805], while NPY overexpression combined with histone serotonylation drives malignant progression in brain cancers [135].

Microbes existing in the TME further strengthen their ecological power by employing different epigenetic mechanisms mediated by SCFAs, lactate, and miRNAs. These include interactions in which SCFAs and adenylate metabolites inhibit bacterial DNMTs, thereby reversing the methylation‐mediated silencing of acid‐resistance and antioxidant genes [806]. Bacterial histone‐like proteins undergo lactylation, a modification that relaxes chromatin structure and activates genes involved in adhesion and drug degradation [807]. Additionally, bacterial sRNA/CRISPR networks are reshuffled by exosomal miRNAs from a tumor, promoting an acetylation‐linked metabolic shift [808]. Furthermore, microbes regulate DNMTs activity via quorum‐sensing molecules, enhancing the expression of acid‐resistance and antioxidant genes to enhance their adaptation to the TME [809].

Tumor cells and microbial communities create a closed‐loop regulatory network that includes epigenetics, immunity, and metabolism through these processes. Metabolites such as SCFAs, indole derivatives, BAs, and organic acids are secreted by microbes in the gut as well as the intratumoral microbes. These substances affect the expression of immune checkpoint proteins, the differentiation of T cells, and epigenetic states, which all help remodel the tumor immune and metabolic landscapes (Figure 18).

**Figure 18:**
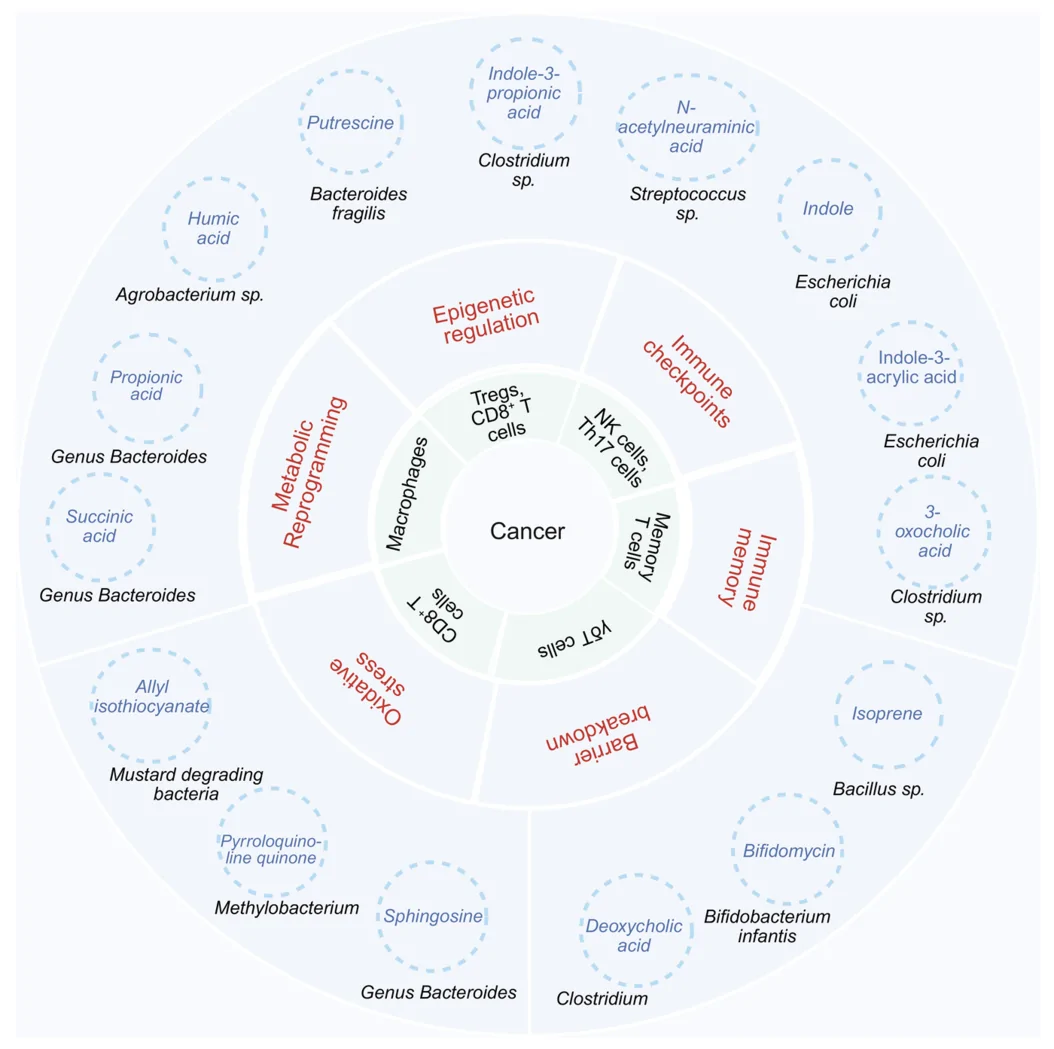
Microbial metabolites regulate cancer through multiple dimensions including epigenetic regulation, immune checkpoints, immune memory, barrier breakdown, oxidative stress, and metabolic reprogramming. Different microbial species and their metabolites shape the TME by influencing host immunity, metabolism, and epigenetic states. For instance, *Bacteroides* spp., *Clostridium* spp., and *Escherichia coli* produce metabolites such as propionic acid, succinic acid, indole derivatives, and 3‐oxocholic acid, which regulate immune checkpoints, Treg/Th17 balance, and chromatin accessibility. Other species including *Bifidobacterium infantis*, *Bacillus* spp., and *Methylobacterium* release BA derivatives, isoprene, or pyrroloquinoline quinone, contributing to the control of oxidative stress, the integrity of barriers, and metabolic reprogramming. These microbiota–metabolite–host interactions form an integrated network that influences tumor initiation, progression, and therapeutic responses. Color coding distinguishes molecular types and functional axes: light blue—SCFAs, organic acids, and polyamines; blue—indole and tryptophan‐derived metabolites; green—BAs and lipid‐related metabolites; purple—redox‐active metabolites and oxidative stress‐related cofactors.

This two‐way interaction helps in maintaining microbial homeostasis, while microbial metabolites and epigenetic signals engage in reciprocal regulation with the host. Ultimately, this constructs a multidimensional host–microbe–epigenetics network, revealing new therapeutic opportunities for microbiota‐based approaches, such as probiotics, fecal microbiota transplantation (FMT), and postbiotics, to remodel the tumor epigenetic state.

#### Hormone metabolite‐mediated microbiome–host interactions

The gut microbiota and the host epigenetic landscape in TME are remodeled by endogenous hormones and their microbiota‐derived metabolites through multilayered positive feedback loops. This interaction is particularly important in hormone‐dependent tumors, such as BrCa and OvCas. The gut microbiota, through β‐glucuronidase activity, hydrolyze conjugated estrogens (e.g., estrogen glucuronides) to release the active estrogens. Once back in host circulation, these active estrogens affect tumor cells by leading to histone modifications (H3K27ac and H3K9ac) and promoting DNA demethylation. This process induces the activation of ER‐target gene networks, resulting in tumor proliferation [810, 811].

In addition, the intestinal epithelial cells of the host are influenced by hormonal signals that alter their epigenetic state and secretory profile. This change enriches the gut microbial community with strains possessing androgen‐metabolizing enzyme activity. Some microbes that have high estrogen‐breaking power may also enhance local hormone processes. These changes in microbial composition, in turn, promote the growth of tumor cells. This positive feedback loop, referred to as the ‘tumor–microbiome–epigenetics' interaction, aids tumor immune evasion and therapy resistance, which is a new explanation for endocrine therapy resistance.

Notably, tumor‐derived exosomal miRNAs are also integral to regulating this loop. They exert their influence by impacting microbial metabolic networks like esterification and metabolite biosynthetic pathways, conferring microbial growth advantages. This feedback loop between the microbiota and host epigenetic and immune response development is generated through various metabolite‐mediated mechanisms.

### Bidirectional crosstalk between epigenetics and microbial metabolism

#### The SAM metabolic feedback loop

SAM, the principal methyl donor within cells, is tightly regulated by one‐carbon metabolic pathways, including the methionine and folate cycles. SAM donates methyl groups to DNA, histones, and RNA through methyltransferases, resulting in the production of SAH as a byproduct. SAH functions as a feedback inhibitor and, together with SAM, maintains the dynamic equilibrium of the cellular “methylation index” (the SAM/SAH ratio) [812]. In the TME, the availability of SAM is significantly influenced by tumor‐driven metabolic alterations, particularly through the regulation of key enzymes such as MAT2A and MTHFR [813]. Disruption of this balance reshapes genome‐wide methylation patterns and drives tumor metabolic reprogramming.

MAT2A, a core enzyme in the SAM metabolic feedback loop, catalyzes the biosynthesis of SAM. The loss or inhibition of MAT2A creates unique therapeutic vulnerabilities across various cancers. For instance, in tumors with *CDKN2A*/*MTAP* co‐deletions, the depletion of MAT2A selectively suppresses CRC proliferation, thereby identifying it as a promising therapeutic target [814]. In HCC models, the combined inhibition of MAT2A and GSK3 significantly suppresses tumor growth by disrupting methionine metabolism [815]. In CRC, MAT2A‐mediated methylation of *PDCD6* supports cell survival under low‐glucose conditions [816]. Collectively, the integration of MAT2A function with SAM homeostasis highlights novel strategies for targeting one‐carbon metabolism and restoring methylation equilibrium in cancer.

This study emphasizes the importance of the SAM metabolic feedback loop in connecting epigenetics to tumor metabolism and reveals new avenues for therapeutically targeting SAM metabolism and its downstream pathways.

#### Dynamic balance of acetyl‐CoA

Acetyl‐CoA links metabolism to epigenetic regulation in cells. Acetyl‐CoA is an important intermediate in the TCA cycle and lipid biosynthesis, providing the essential substrate for histone acetylation. Microbial metabolites such as butyrate and propionate are converted into acetyl‐CoA, which can then be regenerated in the nucleus via ACSS2 to fuel site‐specific histone acetylation. This metabolic–epigenetic bridge connects cellular metabolic state to transcriptional control [817].

KAT2A has recently been identified as a histone lactyltransferase that converts lactate into histone H3 lactylation marks, thereby activating signaling pathways such as Wnt/β‐catenin and NF‐κB. This activation promotes immune evasion and tumor progression [817]. Furthermore, a blocking peptide that disrupts the interactions between ACSS2 and KAT2A has been shown to synergize with anti‐PD‐1 therapy, enhancing antitumor efficacy. In BRAF inhibitor‐resistant melanoma, ACSS2 coordinates acetaldehyde metabolism with chromatin regulation, thereby maintaining acetyl‐CoA homeostasis [818]. In PDAC, ACSS2‐mediated production of acetyl‐CoA downregulates CA9 in an NF‐κB–dependent manner, which further enhances immune evasion [819].

Altogether, these mechanisms highlight the importance of acetyl‐CoA management in tumor metabolic reprogramming. Targeting key nodes such as ACSS2 and KAT2A, particularly in combination with immunotherapy, can provide effective strategies to overcome metabolic adaptation and therapeutic resistance.

#### TCA cycle metabolic axis

The TCA cycle is a central energy source for cells and a source of metabolites that function as epigenetic regulators. The activity of TET and JmjC domain‐containing histone demethylases, which remove methyl groups from DNA and histones, is strictly dependent on α‐KG as an essential cofactor. On the other hand, the accumulation of succinate and fumarate exerted competitive inhibition of these demethylases, leading to hypermethylation‐induced gene silencing [691, 820].

In CRC, upregulation of SLC25A22 promotes succinate accumulation, which activates the WNT/β‐catenin signaling pathway, enhances cancer stemness, and confers resistance to 5‐FU. Targeting this pathway represents a promising therapeutic strategy [216]. In ependymoma models, metformin disrupts metabolic reprogramming, restores H3K27me3 levels, and suppresses tumor growth, highlighting the therapeutic potential of combined metabolic and epigenetic targeting [768]. Butyrate has demonstrated preventive and antitumor activity in CRC by modulating KEAP1/NRF2 signaling [564]. In contrast, in HCC, the combination of pyruvate metabolism modulation with Gln‐restricted diets reveals novel metabolic vulnerabilities [821].

These present findings reveal that TCA cycle metabolites not only participate in energy metabolism but also regulate epigenetic mechanisms underlying malignant phenotypes, positioning them as promising, precise therapeutic targets.

#### Gln metabolic axis

Gln metabolism contributes to the TCA cycle by producing α‐KG and simultaneously acts as a source of this essential cofactor for TET and JmjC domain‐containing demethylases, which directly influences DNA and histone demethylation. Tumor‐driving mutations, such as H3.3K27M and mIDH1, exploit this pathway to maintain malignant epigenetic states [822].

In PDAC, variants of *SLC1A5* enhance Gln‐mediated ATP generation, conferring resistance to gemcitabine [823]. In melanoma, the ALDH1A3–ACSS2 axis coordinates the metabolism of acetaldehyde with chromatin regulation [818]. In NSCLC, the combined inhibition of GLS and CPT1 suppresses tumor proliferation, suggesting that increased dependency on Gln may represent a novel therapeutic vulnerability [824].

In general, the reprogramming of tumor metabolism provides not only energy and biosynthetic substrates, but also directly alters epigenetic machinery. Key regulatory metabolites include acetyl‐CoA, α‐KG, succinate, fumarate and NAD^+^. The combined activities of glycolysis, TCA cycle, Gln metabolism, fatty acid oxidation (FAO), and OXPHOS generate nucleotides, proteins, membranes, and energy while simultaneously modifying the epigenetic landscape (Figure 19).

**Figure 19:**
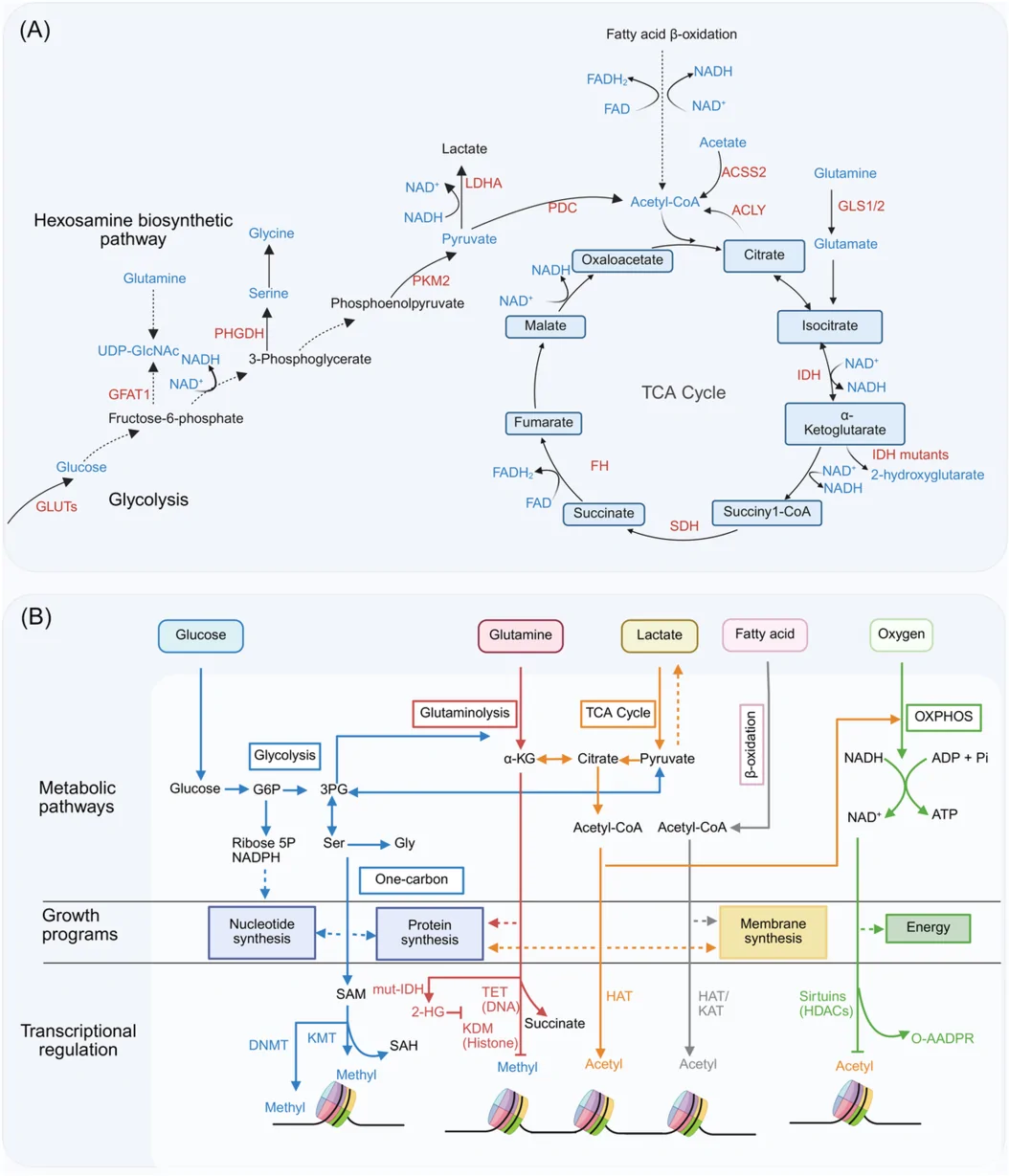
Main metabolic pathways of tumor cells provide substrates and regulate transcriptional programs for growth and epigenetics. (A) The primary metabolic pathways of tumor cells, including glycolysis, the TCA cycle, Gln metabolism, fatty acid β‐oxidation, and OXPHOS, dynamically supply metabolites such as acetyl‐CoA, α‐KG, succinate, fumarate, and NAD^+^. These intermediates not only support cell growth but also function as cofactors or regulators of epigenetic enzymes. (B) Metabolic reprogramming channels glucose, Gln, lactate, fatty acids, and oxygen into growth programs, including nucleotide, protein, and membrane synthesis, as well as energy production. Simultaneously, metabolic intermediates directly regulate DNA/histone methylation and acetylation through various enzymes such as DNMTs, TET, KDMs, HATs, and HDACs, thereby linking metabolism with epigenetic regulation and tumor progression. Color coding distinguishes molecular types and functional axes: In (A), text colors denote: red, metabolic enzymes; blue, metabolites and cofactors. In (B), color coding tracks specific metabolic‐epigenetic linkages: blue, glucose and one‐carbon metabolism linked to methylation; red, glutamine metabolism linked to demethylation regulation; orange, acetyl‐CoA pathways linked to acetylation; green, bioenergetics and redox state linked to deacetylation. HAT(s), histone acetyltransferase(s); KDM(s), lysine demethylase(s); NAD, nicotinamide adenine dinucleotide; OXPHOS, oxidative phosphorylation; TET, ten‐eleven translocation.

### Epigenetic drugs–microbiota interactions

#### Effects of DNMTis

The combination of microbial metabolites and DNMTis can alter the tumor immune environment. DNMTis like 5‐Aza can reverse aberrant genomic hypermethylation and reactivate TSGs [825], a process in which the gut microbiota plays an essential role.

Initially, metabolites from mycobiota can “prime” DNMTi effects. The SCFAs can act as natural HDACis, promoting histone acetylation, which in turn facilitates DNA demethylation. SCFAs also alter the SAM/SAH ratio to further induce demethylation. SAM levels in the host are also regulated by microbially generated folate and niacin, one‐carbon metabolism intermediates that influence DNMT activity [825].

Moreover, DNMTis treatment can reciprocally reshape gut microbial composition, often enriching for butyrate‐producing bacteria. This establishes a “host–microbiota–epigenetic” interaction network that ultimately leads to an alteration of the tumor immune microenvironment [826, 827].

This interaction has different therapeutic potentials in different stages of tumor development. During the preventive stage, administration of a *BSp* and *Ash* combination was shown to modulate the signaling pathways, epigenetic regulation, and gut microbiota, preventing ER⁻ BrCa [828]. Involvement of gut microbiota in mediating CD4^+^ T cell regulation is closely connected to sensitivity to neoadjuvant chemotherapy, suggesting the microbiome may serve as a predictive biomarker for therapeutic response [829]. MDSCs make use of autocrine IL‐6 signaling to hijack the STAT3‐DNMT epigenetic circuit, silencing TNF‐α to overcome immune suppression. This inhibits TNF‐α‐induced and RIP1‐dependent necroptosis, thereby sustaining the survival and accumulation of MDSCs. According to these findings, inhibition of IL‐6 expression or activity could have therapeutic value in obstructing MDSC persistence in TME [830].

#### Metabolic reprogramming by HDACis

HDACis can change gene expression by modifying histone acetylation and impacting the metabolism pathway. HDACis like vorinostat can upregulate important enzymes in glycolysis as well as lipid synthesis pathways—including HK2, LDHA, and FASN—thus altering the metabolic profile of tumor cells. Simultaneously, these inhibitors restore mitochondrial respiratory chain activity and redistribute metabolic intermediates such as acetyl‐CoA and α‐KG by targeting mitochondrial sirtuins such as SIRT3 and SIRT5. The combined application of HDACis and FAO inhibitors has been shown to extend survival in animal models of PDAC [831]. The glycolysis–H3K18la–TTK/BUB1B positive feedback loop exacerbates functional impairment in PDAC [13].

Metabolic alterations can serve as biomarkers for repurposing HDACis. The activation of NRF2 sensitizes LUAD cells to class I HDACis, highlighting their therapeutic potential in solid tumors [832]. In liver fibrosis models, inhibition of LDH impedes Gln metabolism via the lactate/HDAC/*c‐MYC* signaling axis, identifying a novel therapeutic target [833]. In TNBC, metformin enhances the antitumor efficacy of HDACis by blocking FGFR4 signaling and disrupting metabolic reprogramming [834].

Furthermore, the combination of epigenetic targeting with HDAC inhibition may unveil novel therapeutic strategies. In ervical cancer, the expression of *SLC5A8*, which is suppressed by promoter hypermethylation, can be partially restored by HDACi treatment [835]. In neuroblastoma (NB), HDACis upregulate the sialyltransferases ST3GAL5 and ST8SIA1, thereby promoting ganglioside GD2 biosynthesis and suggesting potential synergy with anti‐GD2 immunotherapy [836].

The modulation of endogenous SCFA activity by HDACis sets up a feedback loop connecting epigenetics, metabolism, and the microbiota. This cycle amplifies tumor metabolic reprogramming and additionally facilitates the activation of metabolism‑associated transcription factors such as PPARγ and PGC‐1α. Through these mechanisms, adaptive change in TME occurs, which provides the rationale for combining HDAC inhibition with the metabolism‐targeting therapies. Table 15 provides a summary of representative paradigms and evidence of synergistic effects of metabolite and epigenetic drug combinations and anti‐tumor strategies.

**Table 15:** Application of apparent drug‐microbial metabolite combination therapy in cancer.

Epigenetic modulators	Microbial metabolites	Combined mechanisms of action	Regulation of TME	Clinical translational potential	Associated tumor types	References
ACSS2; KAT2A	Lactate	Blocking the ACSS2–KAT2A interaction combined with immune checkpoint inhibition	Upregulates PD‐L1 expression and mediates immune evasion	ACSS2–KAT2A blocking peptide combined with anti‐PD‐1 therapy	GBM	[817]
SFN, GE, NaB	NaB (SCFA)	Synergistic inhibition of DNMTs, HDACs, HMTs, and HATs	Suppresses proliferation and induces apoptosis/necrosis	Nutrient‐based combination therapy for BrCa	BrCa cell line models	[837]
HDAC inhibition	SCFAs	GPR41/AhR/HIF‐1α promotes IL‐22 production	Enhances IL‐22 to maintain intestinal homeostasis	SCFA supplementation improves intestinal inflammation	CRC	[33]
Valproate, Vorinostat (HDACis)	Propionate, Butyrate	Promote H3K23 acetylation/propionylation	Enhance active chromatin accessibility	Epigenetic therapeutic potential and mutation‐targeted strategy	Multiple cancers (including BRPF1‐mutant tumors)	[838]
HDACis TSA, NaB	NaB, SCFA	Promote histone acetylation related to ATP2A3 transcription	Regulates calcium homeostasis, affecting proliferation and apoptosis	Epigenetic drugs with therapeutic potential via calcium signaling modulation	BrCa, GCa, CRC, LuCa	[839]
NaBu (HDACis activity)	NaB, SCFA	TRAF6‐dependent regulation of TXNIP ubiquitination	Enhances E‐cadherin to suppress migration and proliferation	Combination targeting of TRAF6/TXNIP to inhibit NSCLC progression	NSCLC	[840]
Trichostatin A (HDACi)	Butyrate (SCFA)	HDAC inhibition enhances histone acetylation	Suppresses mast cell inflammatory cytokine release	Inflammation‐associated cancers	Combination therapy strategy targeting inflammation‐associated cancers	[841]
HDACis	SCFAs (acetate, propionate, butyrate)	HDAC/NF‐κB inhibition and PPARγ activation	Regulates intestinal barrier drug transporters	Optimization of oral drug exposure and therapeutic efficacy	CRC	[842]
HDAC inhibitory effect (acetate salts)	Acetate	HDAC inhibition and miRNA regulation	Suppresses proliferation, invasion, and angiogenesis	Epigenetic drug discovery for GBM	GBM	[843]
*DNMT3A;* MBD4; 5‐azacitidine	Acetate (via ALDH2‐mediated acetaldehyde metabolism)	ALDH2 upregulation and MAPK inhibition	Suppresses EMT and promotes apoptosis	ALDH2 as a prognostic biomarker in bone metastasis	LuCa bone metastasis	[844]
HDACis	SCFAs (butyrate, propionate, acetate)	SCFAs synergize with AhR ligand activation	Enhance Treg induction and suppress inflammation	Improve intestinal inflammation and cancer prevention/treatment	CRC	[845]
NSUN2 (m5C methyltransferase), YBX1 (m5C reader protein)	l‐lactate	Activates the HIF‐1α/ENO1/AKT/PI3K pathway	Promotes PDO growth without altering drug resistance	Optimization of PDO culture for precision medicine	HCC, PDAC	[846, 847]
BET inhibitors (JQ1, OTX015, ABBV‐075)	l‐kynurenine (tryptophan metabolite)	BET inhibition reduces IDO1 expression and its metabolites	Blocks IDO1‐mediated immune evasion pathway	BET inhibitors combined with IDO1 inhibitors show therapeutic potential	IDO1‐positive tumors	[848]
BET inhibitor JQ1	Tryptophan/kynurenine pathway	IDO inhibition, BET blockade and DOX‐induced ICD	Enhances CD8^+^ T cell activation and suppresses Treg cells	Multi‐drug nanodelivery significantly suppresses tumor growth and prolongs survival	Melanoma	[849]
HDACi Vorinostat	LCA	Epigenetic regulation by VOR + anti‐proliferative effect of LCA in synergy	Enhances cytotoxicity via FA receptor targeting	FA–PEG–cholesterol nanocapsules improve drug delivery efficacy	BrCa	[850]
BET inhibitors	Succinate accumulation	BET inhibition alleviates metabolic vulnerability caused by SDHB deficiency	Enhances glycolysis dependence and impacts energy metabolism	Provides a novel targeted therapeutic option for SDHB‐mutant tumors	CRC	[851]
T3E inhibitor	NaB	Demethylation and histone acetylation upregulates DKK1	Inhibits Wnt signaling and promotes apoptosis	T3E shows potential as an anti‐mesothelioma agent	Malignant mesothelioma	[852]
HDACi Trichostatin A (TSA)	Butyrate	Downregulation of miRNAs and upregulation of p21 inhibit proliferation	Suppresses adenoma cell cycle progression	Preventive potential in early CRC	Colorectal adenoma	[853]

#### Epigenetic drug‐mediated metabolic interference

Epigenetic drugs can modify the epigenetic landscape of tumor cells, thereby increasing their sensitivity to conventional and immunotherapeutic interventions [854, 855]. These agents not only affect host cells but also reshape the metabolism and epigenetic state of gut microbiota, significantly influencing drug efficacy, toxicity, and resistance. For instance, HDAC1 and HDAC6 are crucial in the growth and invasiveness of *IDH1*‐mutant gliomas; their inhibition can partially reverse the gene dysregulation induced by *IDH1* mutation [856]. In CRC, *SULF1* ^*+*^ CAFs play a crucial role in promoting angiogenesis and tumor progression, while also serving as biomarkers for response to HDACis [857]. Oncolytic virotherapy may impair the recruitment of immune cells and reduce the efficacy of chemotherapy; however, the activation of TLR3 can restore pathway function and enhance the effectiveness of 5‐FU. This suggests that preserving virome integrity could be a promising adjunct strategy for chemotherapy in CRC [858]. Therefore, epigenetic regulation can indirectly influence therapeutic efficacy and drug resistance by modulating microbial metabolic activity [859, 860].

The interactions among diet, pharmaceuticals, and microbiota exert profound effects on drug metabolism. For instance, soybean saponins, when metabolized by gut microbes, can induce hepatic drug‐metabolizing enzymes, consequently reducing the plasma concentrations and anticancer activity of PI3K inhibitors. Conversely, a high‐carbohydrate, low‐phytochemical diet has been shown to synergize with PI3K inhibition in suppressing tumor growth [861]. In addition, microbiota‐derived metabolites such as tyrosol exert antitumor effects by inhibiting the HIF‐1α/NF‐κB signaling pathway [862], while 3‐methylxanthine may potentiate OvCa chemotherapy through a dopamine D1 receptor–dependent mechanism [863].

Exogenous epigenetic agents (DNMT and HDACis) modify metabolic enzymes of the host via epigenetic regulation. Besides, they modify microbial metabolic functions as well as epigenetic states, which affects drug pharmacokinetics and resistance. Additionally, epigenetic changes in microbes can quickly regulate efflux pumps and degradative enzymes to rapidly evolve resistant strains. A study on *Escherichia coli* has shown that the knock‐out of *dam* affects bacterial growth. Moreover, the *dam* knock‐out has also been linked to antibiotic resistance via the multidrug efflux pump AcrAD [864].

The use of drugs to interfere with epigenetic metabolic processes, involves interactions among the host, microbiome, and metabolites. This has important implications for the optimization of combination strategies in cancer.

## RESEARCH STRATEGIES

### Multi‐omics integration strategies

#### Integrated metabolomics–epigenomics analysis

Metabolic reprogramming and epigenetic dysregulation in tumors are closely intertwined. Key metabolic intermediates, such as α‐KG and succinate produced in the TCA cycle, can directly act as substrates or inhibitors of epigenetic enzymes, thereby modulating the epigenetic landscape and influencing gene expression in tumor cells. DNA methylation, a fundamental epigenetic hallmark, is characterized in tumors by the coexistence of promoter hypermethylation of TSGs and genome‐wide hypomethylation [865]; such features are commonly observed in *IDH*‐mutant brain tumors [866], *KRAS*‐mutant CRC [216], and breast CSCs [867]. These differentially methylated regions display pronounced tumor heterogeneity [868] and can promote oncogenesis or immune evasion by activating alternative promoters, thereby increasing transcript diversity [691]. For example, in pediatric GBM, hypermethylation of the *TET1* promoter is closely associated with malignant progression of the disease [869].

To mechanistically understand this process, the “metabolic supply‐enzyme activity‐chromatin state‐cell fate” axis can serve as a conceptual framework. Compounds like α‐KG cooperate with epigenetic enzymes (e.g. DNMTs, TETs) to maintain a balance in the dynamics of DNA methylation; disruption of this equilibrium contributes to tumor initiation and progression [870, 871, 872]. Additionally, metabolic intermediates such as α‐KG and acetyl‐CoA have been shown to remodel the TME via immune polarization, tumor cell migration, tissue repair and oncogenesis [873, 874]. Consequently, the enzymes metabolic enzymes such as ACC, citrate lyase, and *IDH* which are functionally implicated in both metabolism and disease progression, have emerged as important therapeutic targets. In the context of *IDH* mutations, supplementation with α‐KG analogs is proposed to restore normal demethylation activity and inhibit tumor progression [875, 876, 877, 878].

Building upon this central regulatory axis of metabolism, epigenetics, and cell fate, we propose a causality‐oriented multi‐omics integration framework comprising three dimensions. The first is the metabolomic dimension. The quantity of metabolites derived from both the host and the microbiota can be assessed using techniques such as liquid chromatography–mass spectrometry (LC–MS/MS), gas chromatography–mass spectrometry (GC–MS) as well as nuclear magnetic resonance (NMR) [879, 880], while the incorporation of temporal and spatial metabolomic mapping further enhances this analysis [881]. The second is the epigenomic dimension. This involves high‐resolution characterization of DNA methylation and hydroxymethylation via bisulfite sequencing (Bisulfite‐seq/oxBS‐seq) [882], histone modification profiling using ChIP‐seq/CUT& Tag [883], and application of single‐cell technologies to localize cell populations influenced by metabolic states [884]. The third is data integration and causal inference. This step integrates metabolic, epigenetic, and transcriptomic datasets through the application of O2PLS and MOFA methodologies, followed by causal inference performed utilizing dynamic Bayesian networks and Granger causality analysis [885, 886]. Finally, the inferred causal effects are finally validated using organoid systems and murine models, thus establishing a closed‐loop framework that verifies the sequential relationship from ‘metabolic supply’ to ‘enzyme activity,’ ‘chromatin configuration,’ and ultimately ‘cell fate.’.

#### Single‐cell spatial epimetabolomics

Single‐cell technologies are critical for understanding tumor heterogeneity and drug resistance. According to researchers, approaches that integrate spatial resolution with multi‐omics dimensions significantly improve the systematic assessment of host‐microbiome interactions. These methods enable the concurrent analysis of the spatial localization of microbes and the epigenetic states of the host. Transcriptomic, metabolomic, and chromatin profiling data can also be integrated to build regulatory networks across cell types and spatial domains. The specific taxa present in the bacteria of the murine intestine exhibit strong spatial coupling with immune gene expression in the host. This means that metabolites produced by microbes can alter the epigenetic state of the host through gene transfer and/or biochemical pathways [887].

Spatial multi‐omics methods offer a number of benefits, such as high spatial resolution, strong integrative capacity, and enhanced causal inference power. They provide efficient tools to decipher the interactions within the TME. Future studies will integrate spatial metabolite imaging with single‐cell epigenomic profiling to create more precise integrative models of “microbial metabolism‐host epigenetics‐cell fate” to develop therapeutic strategies for cancer therapy.

#### Microbial genomes–host epigenome associations

The microbial genome possesses a substantial metabolic potential, enabling the production of molecules that directly influence host epigenetics, including SCFAs, secondary BAs, and polyphenol‐derived metabolites. These metabolites alter the host epigenetic landscape by modulating HDACs, activating nuclear receptors, or regulating mitochondrial autophagy, thereby governing critical processes such as immune function, lipid metabolism, and Treg differentiation [888, 889, 890, 891, 892, 893, 894]. Conversely, alterations in the host epigenetic state—such as methylation of the *TLR4* promoter or changes in H3K27me3 levels—can influence the colonization and abundance of specific microbial taxa, thereby establishing a bidirectional regulatory loop [895].

To elucidate the thoughts regarding the complex interplay between host and microbes, metagenomic sequencing can be coupled with epigenomic profiling. For example, METABOLIC (Microbiome, 2022) offers functional annotations of microbial genomes, metabolic pathway reconstruction, and association studies with host epigenetic targets. This method builds cascade networks linking microbial functional genes, metabolic pathways and epigenetic targets [896]. Data from different scientific disciplines are used in the integration process. Cross‐omics data integration clarifies the relationship of the microbes with the host and helps to identify new therapeutic targets.

#### Case studies of multi‐omics integration

Research on PDAC shows that integration of spatial transcriptomics with 16S rRNA sequencing revealed considerable overlap between regions enriched for histone acetylation (H3K9ac) and specific intratumoral microbes (*Pseudomonas*). This study found that the process of “local bacterial enrichment → metabolite secretion → host epigenetic modification” takes place [897]. A further inquiry integrating host metagenomics, metabolomics, and transcriptomics of the host revealed a systemic correlation between serum butyrate concentrations and the epigenetic activation of TSGs in the host [218].

These case studies demonstrate the powerful capability of multi‐omics integration to elucidate the mechanisms of microbe–host interactions, providing new concepts and methodologies to the study of TMEs.

### Experimental model construction

#### Humanized microbiota mouse models

Humanized microbiota mouse models are established by transplanting human fecal microbiota into germ‐free mice, providing a critical in vivo platform for investigating human‐relevant microbial communities and their systemic effects on the host. These models are capable of stably maintaining a “human‐derived” microbial community structure [898] and have been successfully applied to simulate complex ecosystems such as the vaginal microbiota [899]. Moreover, they effectively recapitulate the activation of the host immune system by microbes without provoking severe inflammatory responses. This characteristic makes them an ideal model for studying the homeostasis and dysregulation of the tumor immune microenvironment [900, 901, 902].

Nonetheless, several limitations persist. Differences among donors and community drift may hinder reproducibility, while species differences between murine and human immune systems may affect translational relevance. The composition and stability of microbial colonization also greatly depend on external factors such as diet and cage effects.

#### Tumor organoid–microbe co‐culture

Organoids are self‐organizing three‐dimensional culture systems that closely mimic tissue architecture and function in vitro, making them highly suitable for studying host‐microbe interactions. This model facilitates the investigation of how microbes and their metabolites regulate DNA damage, signal pathway activation, and inflammatory responses in host cells [903, 904, 905]. In particular, patient‐derived organoids offer a valuable platform for exploring individualized therapeutic responses [906].

However, several limitations persist. Organoids lack blood vessels, nerves, and a full immune system. Moreover, the time frame for co‐culturing with anaerobic bacteria is limited, which may constrain microbial growth and toxin release. The origin of the extracellular matrices and the composition of the culture media influence the metabolic states and, accordingly, the reproducibility of the results.

#### CRISPR–Cas‐mediated metabolite editing

CRISPR–Cas genome editing enables precise targeting of specific genes and serves as a powerful tool for elucidating the causal relationships between microbial metabolites and tumor epigenetics. By applying this technology to organoids or primary cells, tumor models with defined genetic backgrounds can be rapidly constructed [907, 908, 909], facilitating the validation of key molecules within the microbe‐metabolite‐epigenetic interaction network.

Although CRISPR–Cas holds great potential for metabolite editing, several obstacles remain, including efficient delivery, editing fidelity, off‐target effects, and *in situ* editing feasibility. The outcomes of gene editing can be further validated using organoid systems or humanized mouse models. Their role in systemic immunity, drug metabolism, and microenvironmental remodeling can also be assessed.

### Other biological approaches

In addition, emerging biological and computational strategies provide complementary perspectives. Detailed analyses of various cell types within the TME—such as the functional heterogeneity of CAFs [910], the dynamic changes of circulating tumor cells [911], and the interaction mechanisms of immune cells [912]—have laid the groundwork for understanding complex cellular networks. More importantly, AI and ML are emerging as central strategies for the integrative analysis of high‐dimensional multi‐omics data. These approaches can be applied to non‐invasive cancer detection and the prediction of therapeutic responses [913], offering critical tools for identifying novel biomarkers from complex datasets.

## CLINICAL TRANSLATION AND THERAPEUTIC STRATEGIES

### Development of diagnostic biomarkers

#### Microbial metabolite fingerprinting

Microbial metabolite fingerprinting can capture thousands of metabolite variations along the “microbe–metabolite–host” axis, thereby comprehensively depicting tumor‐associated metabolic states. These fingerprints arise from the disturbance of metabolic networks and perturbation of the immune microenvironment caused by microbial dysbiosis and may represent a new source of biomarkers for early diagnosis, molecular subtyping, and therapeutic response.

Typically, high‐throughput platforms such as LC–MS/MS, GC–MS, or NMR are used to generate metabolite fingerprints, from various biospecimens including plasma, feces, and urine. Research has shown that early‐onset and late‐onset CRC have significantly different microbial‐metabolite interactions, allowing patients to be accurately stratified based on metabolite fingerprints [914]. The combination of fecal microbiota and BAs has been validated for predicting immunotherapy response in HCC [915]. The secondary BA DCA has been ascertained to enhance BrCa cell proliferation [916]. Certain metabolites like HMDB0240606, along with bilirubin and l‐tyrosine, can serve as diagnostic biomarkers for HCC [917].

In data analysis, researchers are applying AI and ML techniques to metabolite fingerprints. Support vector machines (SVM), random forests (RF), and deep learning algorithms are utilized to identify predictive markers of ICI response within metabolite fingerprints. Moreover, multimodal models that merge metabolomic and epigenetic information (e.g., DNA methylation) are now being developed as “metabolite–epigenetic” predictive frameworks, which help enhance the prediction of response to DNMT and HDACis [132, 918, 919, 920, 921, 922].

These approaches have tremendous potential, yet they face formidable challenges in translation. Non‐standardized technical platforms and sample preprocessing lead to poor data comparability. Further, AI models are hindered by “black‐box” interpretability issues and insufficient generalizability. High‐quality multicentric, prospective clinical validation, comprehensive annotation databases, and elucidation of mechanistic causal pathways are still lacking. There is an urgent need for coherent standardized workflows, multi‐ethnic reference databases, and extensive clinical validation studies to promote clinical translation.

#### Epigenetic tag combinations

Combinations of epigenetic tags (DNA methylation, histone marks, chromatin accessibility, and ncRNA expression) allow the creation of multidimensional “epigenomic maps.” Integrated signatures can thus serve as highly sensitive biomarkers and prognostic indicators with a wide range of applications, including early cancer screening, assessment of therapeutic efficacy and prediction of resistance.

Innovative technologies like bisulfite sequencing, ChIPmentation, and ATAC‐seq enable identification of promoter‐specific methylation and histone modifications such as H3K27ac. Elevated concentrations of circulating nucleosomal H3.1 in non‐Hodgkin lymphoma patients can serve as an early diagnostic marker [923]. Moreover, ML models are now used by researchers to optimize the analysis of multidimensional epigenetic datasets to capture subtle changes. EpiScore emerged as an epiproteomic indicator that, unlike many other epigenetic signatures, is predictive across disease states [924]. The integrated analysis of 5mC and 5hmC signals in cfDNA in PDAC has markedly improved the efficacy of early detection [925]. In addition, monitoring changes in epigenetic alterations such as *DNMT1* and H3K9me3 pre‐ and post‐treatment can provide useful information about treatment responses and the development of resistance.

Yet, various barriers impede clinical translation. Technological platform interoperability and sample selection bias affect reproducibility, while the low interpretability of ML models and their “black‐box” nature hinder clinical adoption. Further translational barriers are related to data privacy governance and reimbursement pathways. These challenges can be addressed through algorithm standardization, validation across platforms, co‐development of a universal data and quality‐control system, and collaboration between the industry and clinical enterprises.

#### Microbe–host interaction biomarkers

This kind of biomarker utilizes microbes' signals and immune or epigenetic responses of the host, enabling two‐dimensional “signal–response” recognition. These biomarkers may be useful for early cancer screening, prediction of immunotherapy efficacy, and monitoring of therapeutic dynamics.

Microbial signals include circulating free bacterial DNA (cfbDNA), epigenetically modified exosomes, and metabolites; host response signals encompass immune factors such as IL‐6 and PD‐L1, as well as the epigenetic states of immune cells (e.g., H3K27ac in macrophages). Significant studies have established that microbial signatures associated with *Parvimonas micra* are enriched in CRC patients and can serve as potential non‐invasive biomarkers for early CRC detection [926]. Immune cells interacting with tumor cells are greatly influenced by biochemicals, affecting T‐cell infiltration and immunotherapy response in BrCa [927]. According to research, *Fusobacterium nucleatum* increases CRC invasiveness via the AhR pathway [928]. The presence of *Veillonella parvula* in the lower airways has been linked to the reduced efficacy of ICI in LuCa [929]. In the tumor tissues, *Propionibacterium* and *Streptococcus* perform an immunosuppressive and immune‐activating function, respectively [930]. Also, dietary factors may affect precancerous states by enriching a particular taxon, *Flavonifractor plautii* [931].

The development of biomarkers for these interactions remains exploratory, despite their promise. Current limitations include the weak signal intensity of microbial signals, contamination, high heterogeneity of host responses, and challenges in specificity validation. Moreover, high‐throughput single‐cell technologies are costly and sophisticated platforms that pose difficulties for data integration. Future strategies for improvement may rely on synchronous detection methods based on digital PCR and single‐cell omics, as well as multi‐omics AI modeling. This will improve the signal‐to‐noise ratio and enhance clinical interpretability.

### Therapeutic strategies

#### Precision modulation with probiotics/prebiotics

Interventions with probiotics and prebiotics can reshape the gut microbiota and its metabolomic landscape (especially the production of SCFAs like butyrate), thereby synergistically modulating tumor‐associated epigenetic phenomena. These interventions help decrease treatment‐related toxicity, improve immune efficacy, and enhance the effectiveness of radio‐chemotherapy and epigenetic drugs. Mechanistically, they elevate SCFA levels, such as butyrate, to inhibit HDACs and promote histone acetylation [888], and stabilize the gut microbiome through combined “bacteria+substrate” strategies, thereby contributing to the restoration of epigenetic homeostasis [932].

Several intervention formats exist, such as functional strain supplementations, targeted‐prebiotics and other synbiotic formulations. For example, patients with CRC have shown fewer postoperative complications when administered synbiotics perioperatively [933]. Probiotics help repair intestinal injury caused by radiotherapy [934]. Microbiome analyses have shown an association between *Streptococcaceae* abundance and survival in cholangiocarcinoma [935]. CRC models demonstrate that synbiotics engineered with SynCon can suppress pro‐inflammatory taxa while also retaining butyrate‐producing bacteria [936]. Inulin and pectin are prebiotics that help generate NR precursors, thereby activating the NAD^+^–SIRT pathway. Jointly with probiotics, these interventions may induce a “metabolic positive feedback loop” that upregulates acetylation‐based regulation.

Despite their potential, the clinical application of probiotic and prebiotic treatments faces various problems. Strain variability, differences in colonization efficiency, and bioavailability issues hinder reproducibility. Most clinical trials are observational and provide low‐level evidence. Moreover, standardized strain combinations or optimized formulations have yet to be established, and a clear regulatory framework for classification and quality control is still lacking. Future microbiome diagnostics will be an essential part of clinical implementation. Likewise, nanodelivery systems could optimize intervention efficiency. Above all, large‐scale data “gut–epigenetic” networks will play an important role in dose timing and optimization.

#### Metabolite prodrug design

The innovative strategy of transforming microbial metabolites, including butyrate, into “prodrugs” and delivering them using nanoplatforms enables more precise epigenetic modulation. This approach increases the availability of metabolites in the TME without causing systemic toxicity. Targeted delivery can exploit tumor‐specific characteristics, such as lower pH and higher ROS levels. Controlled release improves the local effectiveness while minimizing exposure to normal tissues. In addition, antitumor effects may be enhanced by combining these prodrugs with epigenetic or chemotherapeutic agents.

Prodrug strategies frequently employ nanoscale delivery systems like PLGA, liposomes, or stimuli‐responsive self‐assembled scaffolds. For instance, camptothecin prodrugs modified with biotin or butyrate encapsulated in nanoparticles featuring RGD peptides have significantly improved tumor targeting [937]. A dual‐delivery system co‐loading butyrate and sorafenib was shown to trigger ferroptosis and induce antitumor immunity in HCC [938]. Moreover, other types of delivery systems including exosomes, PEGylated conjugates, GSH‐responsive platforms and “Trojan horse” carriers have been validated in preclinical studies [939, 940, 941, 942].

Although promising, there are several challenges to this strategy. The enhanced permeability and retention (EPR) effect in different tumors varies widely among different tumors, limiting targeting efficacy. Moreover, scalable manufacturing methods and GLP‐compliant toxicological tests are still under development. The lack of a robust in vitro–in vivo correlation (IVIVC) system and human safety data has hampered clinical translation. To address these issues, future work should focus on standardizing nanoplatform development, incorporating pharmacokinetic studies, and designing closed‐loop delivery systems informed by clinical feedback to accelerate translation into therapies.

#### Epigenetic drug–microbiome combination therapy

Combining epigenetic drugs with microbiota‐based therapies represents a promising strategy for cancer treatment. This approach aims to relieve epigenetic repression and subsequently regulate immune pathways induced by the microbiome, thereby exerting additive or synergistic antitumor responses. Key mechanisms include: (1) activation by DNMTis and HDACis, which increase tumor immunogenicity through ERV and interferon pathways [943, 944]; (2) inhibition of HDACs and promotion of histone acetylation by SCFA metabolites such as butyrate [945, 946]; and (3) activation of immune cells to reverse “cold” tumor phenotypes.

This method has a lot of potential in studies. In HCC, the efficacy of PD‐1 blockade was significantly boosted by supplementation with acetate or *Lactobacillus reuteri* [946]. In CRC, combining azacitidine with butyrate or probiotics further enhanced the expression of immune‐related genes [341]. Moreover, further research highlights that FMT can reverse tumor immunosuppression and strengthen ICI response rates [947]. Importantly, butyrate deficiency in gut is associated with high levels of SULF1⁺ CAFs, which is a marker of sensitivity to HDACis [857]. Furthermore, the toxicities induced by Oxa were mitigated by microbiome interventions [948]. Moreover, DNMTis and HDACis, whether used alone or in combination, significantly inhibit the proliferation of various solid tumors by modulating apoptosis‐related genes such as *BAX* and *Bcl‐2* [718].

Several early‐phase clinical studies have begun to explore the feasibility of “microbiota–epigenetic interventions” in cancer therapy. A phase I trial (NCT03341143, *n* = 15) first evaluated the potential of FMT combined with anti–PD‐1 therapy in patients with PD‐1–refractory melanoma. Some patients regained immune responsiveness, with three achieving partial or complete remission [949]. Subsequently, a multicenter phase I study applied healthy‐donor FMT combined with PD‐1 inhibitors as a first‐line regimen in previously untreated patients with advanced melanoma. The combination was generally well tolerated, and clear signs of disease control and objective responses were observed [950]. In addition, a randomized phase I clinical trial incorporating the probiotic strain *Clostridium butyricum* CBM588 into a nivolumab plus ipilimumab regimen, resulting in a significant improvement in progression‐free survival (12.7 vs. 2.5 months) without additional toxicity [951].

Though clinical results have been promising, there are significant shortcomings. Most investigations are single‐arm, early‐phase trials with small cohorts and short follow‐up, preventing definitive conclusions regarding long‐term efficacy or safety. Microbial translocation, excessive immune activation, and differences between individuals in their genetics, diet, and their commencement microbiota can muddle matters. The lack of standardized controls for donor screening, metabolite quantification, and product quality hinders comparability. Upcoming randomized, multicenter trials must verify efficacy, define clinical endpoints, and integrate metabolomic and epigenomic profiling to identify responders and inform targeted therapeutic intervention.

### Personalized medicine applications

#### Microbial subtyping for therapeutic decision‐making

The microbial subtyping uses metagenomic and multi‐omics data to validate models of functional subtypes of patients' gut microbiota. Examples include butyrate‐producing–enriched, proinflammatory, or TMAO‐dominant types. This differentiation provides a very valid biological rationale for focusing epigenetic therapy, immunotherapy and others. The composition of metabolites and inflammatory character can be influenced by microbial profiles that lead to epigenetic alteration in the host, immune activation and therapy response.

The use of high‐throughput sequencing, 16S/metagenomic clustering and AI subtype recognition enables precision therapy. The microbiome's evolving changes during treatment can function as early predictors of nCCRT effects in the context of locally advanced rectal cancer (LARC) [952]. Research studies show that predictive models using *Akkermansia muciniphila* characteristics in melanoma and GCa have high accuracy in predicting ICI response, with an area under the curve (AUC) value of more than 0.9 [953, 954]. The microbial variation among dMMR and pMMR CRC is notable and suggests the need for specific tailored therapies. FMT has demonstrated potential in NSCLC, slowing the progression of disease and boosting immune response [955, 956]. Moreover, the transplant of an old gut microbiome has improved immunotherapy response in old mice [957]. Additionally, the microbial metabolite methylglyoxal (MG) shows radiomodulatory and immunogenic activity [958].

Real‐time microbial subtyping offers the potential for rapid treatment modification; however, problems still exist, such as the absence of standard nomenclatures, functional annotation schemes, dietary and medication‐induced rapid turnover of the microbiome, high testing costs, and the need for extensive randomized controlled trials (RCT) to help subtype‐driven decision‐making. Future directions should focus on the generation of stable and cost‐effective microbial detection platforms and the construction of integrative frameworks that link “microbial subtype → drug response → therapeutic algorithm” to achieve truly subtype‐driven personalized treatment.

#### Epigenetic response prediction

By incorporating epigenetic information and microbial metabolic states into layered predictive models, it is possible to identify patient populations responsive to therapy in advance, monitor efficacy in real‐time, and make changes as necessary. The approach will lead to “reactive–adaptive” therapy strategies.

Microbiota‐induced epigenetic reprogramming in tumors not only shapes oncogenic processes but also offers actionable biomarkers for response prediction. At baseline, tumor molecular features can aid patient stratification. Clinical epigenetic therapy studies indicate that baseline tumor molecular characteristics related to drug response, such as HR23B expression, are associated with differential sensitivity to HDACis, underscoring the value of biomarker‐guided patient selection [959]. ML models further enhance the construction of composite response scores by integrating epigenomic, metabolomic, transcriptomic, and microbiome datasets. For example, in NSCLC, DNMTi‐mediated epigenetic remodeling improves γδ T cell cytotoxicity, thereby providing a rationale for combination immunotherapy [960]. Dynamic monitoring introduces an additional dimension: alterations in 5mC/5hmC or H3K27ac levels can be utilized to track treatment responses; shifts in SCFA or TMAO levels may be associated with probiotic or dietary interventions; and soluble PD‐1 or circulating miRNAs can act as liquid biopsy markers for real‐time response assessment [961].

However, predicting epigenetic response using multi‐omics integration and high‐dimensional modeling is a significant challenge. Common pitfalls in algorithms are feature leakage and overfitting; biomarker heterogeneity requires multicenter standardization and proper quality control; the integration of predictive models into clinical workflows is limited by challenges in data governance. Consequently, it is crucial to develop a complete end‐to‐end closed‐loop system for response monitoring and feedback to enhance the translational feasibility and clinical applicability of this predictive system.

#### Dynamic monitoring systems

To facilitate a real‐time connection between epigenetic changes and microbial metabolites, a multilayered monitoring system integrating liquid biopsy, single‐cell omics, and spatial imaging is needed. This integration will assist in diagnosis, medication adjustment, and treatment.

Blood samples can be used for DNA methylation analysis by WGBS, RRBS or EPIC arrays. PBMCs can be used for ChIP‐seq profiling of histone modifications and fecal/urine samples for tracking microbiota composition and metabolite spectra [962, 963, 964]. CUT&Tag and CUT&RUN techniques offer high‐resolution detection of histone modifications [965]; DESI‐MSI imaging enables spatial mapping of metabolites, including SCFAs and TMAO; and highly sensitive LC–MS platforms permit absolute quantification [966]. Integrative algorithms such as MOFA and DIABLO extract cross‐omics covariation patterns at the data analysis and application level [967, 968]. Interactive cloud platforms and dashboards enable real‐time visualization, while deep learning architectures like LSTM support trend prediction and personalized intervention recommendations [969].

A modular monitoring framework could significantly enhance the timeliness and accuracy of oncology care powered by microbiome–epigenetic interactions. However, challenges remain, including non‐standardized sampling, high costs, and the complexity of integrating data into clinical practice. In the future, such systems should be integrated into the long‐term management of cancer. This will be supported by health insurance and telehealth networks for timely implementation.

#### Dietary and environmental modifiers of the microbiome–epigenetic axis

Integrating dietary and environmental exposures adjustable inputs into longitudinal monitoring systems can stabilize subtype classification and enhance response prediction because they rapidly alter microbial diversity and metabolism. The host's diet and environment are as important as genetics in generating the tumor‐associated microbiome and metabolic profile. Different food types contribute to the formation of SCFAs, notably butyrate, which can impact HDAC activity. This allows the reactivation of TSGs. High‐fat diet (HFD) causes bacterial imbalance, leading to reduced SCFA production, an increase in pro‐inflammatory taxa, and disrupted DNA methylation. Treatments with butyrate inhibit M1 macrophage polarization, experiments show evidence for this. The inhibition of HDAC activity and the reduction of various pro‐inflammatory mediators are mechanistically linked to this process [159].

### Clinical trials and translational perspectives

The research into the microbiome–metabolite–epigenetic axis has shifted from mechanistic studies to clinical applications, revealing a privileged role for microbial composition and metabolites in the epigenetic regulation of the host. The connection between basics and practice of medicine is a well known phenomenon in literature.

#### Clinical evidence of Microbiome–Epigenetic interactions

The shifts in composition and metabolic activity of cancer‐relevant microbes of diverse cancers co‐occur with epigenetic changes. The abnormal DNA methylation, histone modifications, and ncRNA regulation events. When there is imbalance in the gut microbiota, it leads to lower levels of SCFAs. This in turn weakens the protective effects of SCFAs because they start losing their ability to suppress inflammation, regulate immune homeostasis, and maintain the integrity of the mucosa. Overall, this results in the disturbed epigenetic stability mediated by DNA methylation [970]. On the other hand, high fiber diets could aid in the recovery of butyrate‐producing bacteria and partially reverse the abnormal methylation patterns to improve the immune microenvironment.

Beyond SCFAs, disordered BA metabolism and abnormal proportions of *Clostridium* species have been strongly associated with *MGMT* promoter methylation and CIMP, suggesting that microbial metabolites may modulate the activities of DNMTs and TET enzymes to maintain the dynamic balance of DNA methylation [971]. Moreover, SCFAs have significant activity in HDAC, apart from crotonylation and other PTMs of histones. This helps in linking the metabolic states to chromatin reorganization. Butyrate, as a canonical HDAC inhibitor, selectively inhibits the activity of HDAC1, downregulates short‐chain acyl‐CoA dehydrogenase (SCAD), and then further enhances its epigenetic effects through a cutback in its oxidative metabolism [972].

Changes in plasma or fecal metabolites, including acetate, propionate, and secondary BAs, are tightly associated with the efficacy of epigenetic therapy and may serve as potential biomarkers for disease monitoring. The findings highlight how the microbial metabolic state is an important external force for tumor epigenetic dysregulation and help in its precision stratification.

#### Interventional clinical trials and translational strategies

Clinical strategies focused on the microbiome metabolite epigenetic network have become deeper and deeper with time. A number of adjustable targets and translational opportunities are present. The metabolic and epigenetic pathways coordination may result from nutritional and microecological intervention (high fiber diet or prebiotic supplementation) mediated butyrate increase, inhibition of HDAC activity, activation of TSGs, and enhancement of immune microenvironment.

At the pharmacological level, DNMTis (e.g., azacitidine, decitabine) and HDAC inhibitors (e.g., vorinostat, belinostat) show significant clinical benefit in multiple cancers [973]. For example, the combination of azacitidine or decitabine with the *BCL‐2* inhibitor venetoclax improves the outcomes in Acute myeloid leukemia (AML), while vorinostat inhibits EMT and proliferation in cervical cancer via the ubiquitin‐conjugating enzyme *UBE2C* [974, 975]. Additionally, gut microbiota diversity influences the metabolism, efficacy, and toxicity of drugs; strategies such as FMT or probiotic supplementation can improve drug sensitivity and tolerance.

Research in immunotherapy has increased the clinical relevance of microbiome–epigenetic interactions. The gut microbiota influences the response of patients to PD‐1/PD‐L1 blockade therapy [976]. The microbial metabolite butyrate can directly enhance histone acetylation (H3K27ac/H3K9ac) at promoters of key genes in CD8^+^ T cells, promoting immune‐related transcriptional activation and improving their effector functions, including cytokine production and antitumor activity [42]. IPA enhances the level of H3K27ac in Tcf7 SE regions, maintaining CD8^+^ T cell stemness and giving rise to progenitor exhausted T cells (Tpex), thus enhancing antitumor immune responses [110, 977]. At the same time, epigenetic modulators can induce a “proinflammatory phenotype” that synergizes with ICIs and offers rational mechanistic combinations.

#### Future directions and clinical design considerations

The future translational research should lead to a continuous chain of evidence from mechanism to population. Clinical trial designs should incorporate epigenetic biomarkers (e.g., CIMP status, *MGMT* or *LINE‐1* methylation) and metabolic signatures (including SCFA levels, BA spectra, and aromatic amino acid metabolites) to identify patient subgroups with distinct “microbiome–epigenetic subtypes,” thereby enabling precision stratification and targeted interventions. Microbiome, immune factors, and therapeutic responses of locally advanced rectal cancer patients have to be studied through integrated analyses using their dynamic changes to find biological correlates and predictive models for nCCRT outcomes [954]. Multi‐omics monitoring over time and cross‐omics network analyses further show associations between dynamic microbial metabolism and host epigenetic states during treatment. The gut microbiome, for example, plays an important modulatory role on the proteomic and metabolomic changes brought about by FMT or anti‐PD‐1 therapy [949]. The combination of metagenomic, methylomic, metabolomic, and immunomic data allows for the mapping of the regulatory cascade linking metabolic signaling, epigenetic remodeling, and clinical response to enable mechanistic elucidation and reproducible validation of therapeutic heterogeneity.

Combinatorial strategies in clinical translation based on these insights and with epigenetic therapy are microbiota modulation with prebiotics, optimized diet or metabolite supplementation. Strategies aimed at enhancing therapeutic efficacy, restoring immune balance, and reducing toxicity, have been identified to modulate several key metabolites like butyrate and secondary BAs to amplify and prolong treatment responses. Moreover, polyphenolic compounds, through their antioxidant activity and inhibitory effects on *DNMT1*, exhibit potential influence in epigenetic regulation [978]. Dietary patterns enriched in polyphenols may modulate DNA methylation‐based “epigenetic aging,” representing an emerging “epigenetic diet” strategy with preventive and therapeutic promise [979].

Standardized lifestyle and exposome databases, which take diet, drugs, and environmental exposure into account in individualized analyses, will allow for dynamic monitoring of metabolic variation and therapeutic response. Validation of modulating the microbiome–metabolite–epigenetic network in cancers must be done on large cohorts. Bringing together multi‐omics and exposomics will enable researchers to investigate the causal link between metabolism and epigenetic regulation, thereby advancing this axis from correlative insight to mechanistic and translational precision oncology.

#### Challenges and future directions in clinical translation

Although microbiome–epigenetic combinatorial interventions have demonstrated promising antitumor potential in preclinical studies and early‐phase clinical trials, significant challenges must be overcome before their translation into routine clinical practice. Substantial interindividual variability in gut microbiota composition, dietary patterns, genetic background, and metabolic status leads to highly heterogeneous responses to interventions, making it difficult to construct reliable efficacy prediction models [980]. This complexity is further compounded by the spatiotemporal specificity of metabolite regulation—differences in dosage, tissue microenvironment, and host metabolic conditions can all alter the direction and magnitude of their effects on epigenetic markers. Evidence also suggests that even minor variations in donor microbiota composition may influence the intensity and durability of recipient immune responses, posing major challenges for personalized clinical implementation [980].

Technical limitations and the lack of standardization also constrain research reproducibility. Variations among research centers in formulation preparation, dosage regimens, routes of administration, and quality monitoring hinder cross‐study comparability. Conventional 16S rRNA sequencing can only resolve bacterial taxonomy at the genus level and thus fails to capture strain‐specific functional features, whereas high‐resolution metagenomic sequencing, culturomics, and single‐cell multi‐omics platforms are beginning to fill this gap [981]. At the same time, limited sample sizes remain a major issue: most clinical studies are single‐arm or small‐scale exploratory trials with short follow‐up periods, lacking multicenter randomized validation and long‐term safety assessments [982]. To advance clinical translation in this field, it is essential to establish unified quality control systems for donor selection, metabolite quantification, and endpoint definition, along with standardized research frameworks that enable cross‐platform comparison.

The integration of multi‐omics technologies and AI is transforming the research landscape. By integrating metabolomic, epigenomic, immunomic, and metagenomic datasets, researchers can elucidate the causal chains linking the microbiome‐metabolite‐epigenetic axis, thereby improving precision stratification and therapeutic prediction [983]. AI‐driven multi‐omics models are increasingly capable of identifying key signaling nodes between the host and microbiota, showing promise in risk classification, treatment response evaluation, and drug sensitivity prediction. With the accumulation of high‐quality datasets and improvements in algorithmic interpretability, this field is expected to progress from mechanistic investigation toward dynamic monitoring of therapeutic efficacy.

The clinical translation of interventions that combine the microbiome and epigenetics will require effective regulatory and ethical frameworks to be successful. One of the major future challenges will be to balance efficacy validation with safety assessment while integrating multi‐omics and AI‐based models into regulatory evaluation. The major barriers and possible solutions to clinical translation in this field are summarized in Figure 20 to serve as a reference for research and practice. Although largely theoretical at present, precision microbiome–epigenetic medicine is taking shape and real‐world applicability through collaboration and interdisciplinary integration.

**Figure 20:**
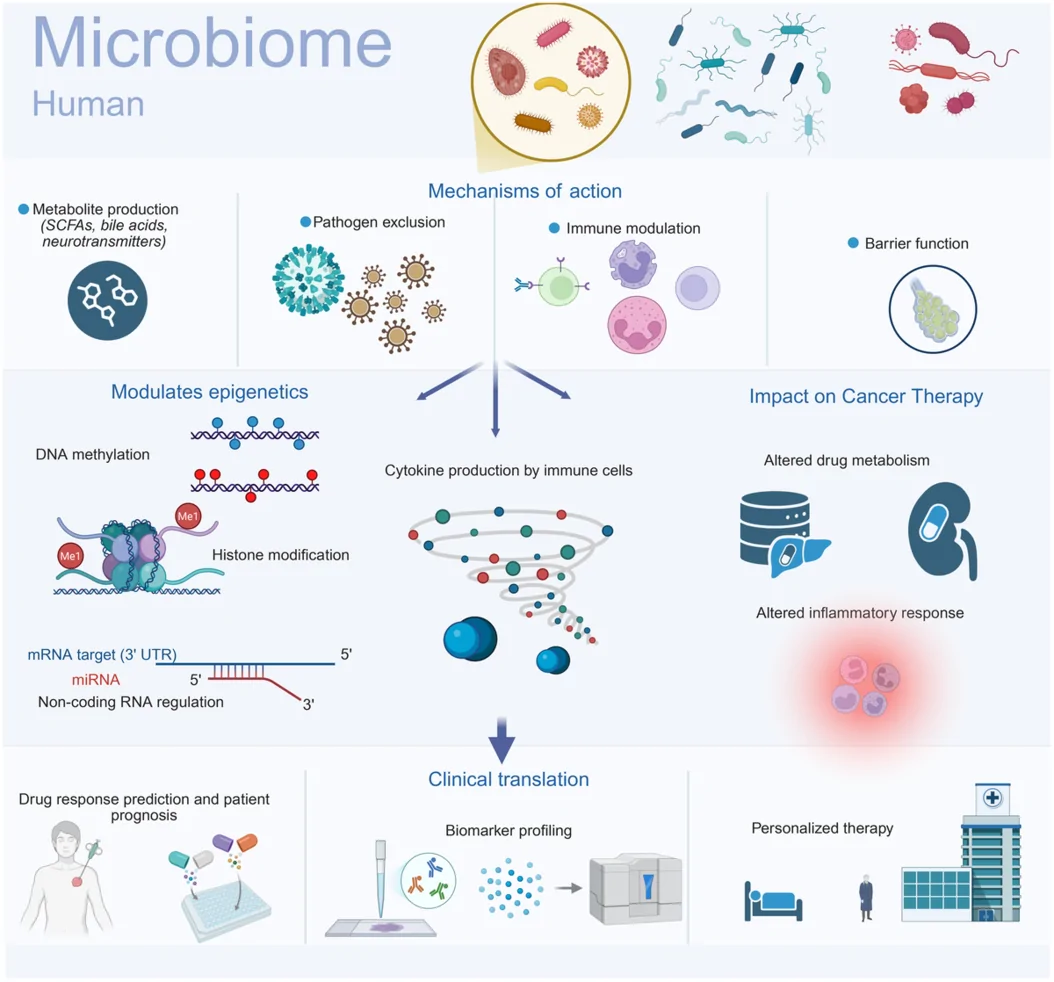
Multilayered mechanisms linking the human microbiome to cancer therapy and clinical translation. The figure illustrates how microbial metabolites such as SCFAs, BAs, and neurotransmitters influence host physiology through multiple mechanisms, including immune modulation, pathogen exclusion, and barrier maintenance. These microbial actions remodel host epigenetic programs—by regulating DNA methylation, histone modification, and non‐coding RNA expression—and shape cytokine production by immune cells. Such epigenetic–immune interactions impact cancer therapy by altering drug metabolism and inflammatory responses. Translational applications include microbiome‐based biomarker profiling, prediction of therapeutic response, and development of personalized precision interventions. This conceptual framework highlights both the translational promise and current challenges in integrating microbiome–epigenetic insights into clinical oncology.

## CONTROVERSIES, CHALLENGES, AND FUTURE DIRECTIONS

### Core scientific controversies

#### Spatiotemporal confirmation of causality

Currently, the most pressing challenge in research is to define accurately the spatiotemporal causal chain linking microbial metabolites to epigenetic changes in the host. Many existing studies rely on cross‐sectional analyses that do not capture the evolving dynamics of microbes, metabolites, and epigenetics factors throughout disease progression. A major challenge is to distinguish between direct regulatory actions of metabolites and feedback regulation by host epigenetic states [984]. The TME is a complex, heterogeneous environment that is difficult to monitor. These factors complicate the accurate reporting of metabolite concentrations and their co‐localization with epigenetic enzymes, from systemic circulation to the cell nucleus.

To overcome these limitations, future research should adopt multimodal, multiscale integrative frameworks. Dynamic tracing can be performed using isotope labeling, which, when combined with high spatiotemporal resolution mass spectrometry imaging in living models can effectively track the migration and accumulation of metabolites across organs in real time. Metabolomic analysis delineates the distribution of metabolites under physiological and pathological conditions, enabling the precise mapping of metabolite dynamics and their temporal relationship to host epigenetics [985]. Temporal inference can be achieved by integrating longitudinal multi‐omics datasets with time‐series network inference to identify the sequential relationships among fluctuations in metabolites, epigenetics, and gene transcription. This approach enhances our understanding of microbe–epigenetic interactions by revealing cumulative and delayed effects [986]. Causal validation involves using CRISPR–Cas9 to edit metabolic pathways and subsequently employing synthetic biology approaches under stringent conditions to test the functional necessity of these pathways for epigenetic regulation, offering direct evidence linking specific pathways or metabolites to the epigenetic control of the host [679, 987]. Clinical integration further combines these technologies with longitudinal clinical cohort studies to trace metabolite–epigenetic causal chains in real time during disease progression [988]. Sampling at multiple timepoints from patients helps capture the immediate consequences of microbial metabolites on host epigenetic states, information that can assist in designing personalized treatments.

The combined effects of microbial metabolites and epigenetic enzyme gene remodeling on different tumor biological processes will jointly delineate the complete causal chain. It may offer a theoretical basis for optimizing precision medicine and tumor treatment strategies.

#### Nonlinear dose–response characteristics

The biological effects of microbial metabolites often exhibit nonlinear or even biphasic (hormetic) dose–response relationships, constituting another major scientific controversy. For example, at a physiological concentration, the SCFA butyrate inhibits HDAC activity and activates TSGs, whereas at supraphysiological levels, it may induce nonspecific cytotoxicity through excessive modulation of the chromatin structure or energy metabolism [842, 989]. Similarly, low doses of lactate promote immune repair via histone lactylation, whereas at higher doses it exacerbates genomic instability by disrupting DNA repair [990].

The spatiotemporal distribution of metabolites is shaped in a tumor type‐specific and microenvironmental threshold‐dependent manner. To delineate these intricate dose–response relationships, high‐precision metabolite profiling across gut, plasma, and tumor compartments can be employed, supplemented by dynamic microfluidic and organoid simulations of epigenetic enzyme activity. The non‐linear use of mathematical models that take into account a multitude of influencing factors as well as multi‐dose in vivo testing together with multi‐omics monitoring will over time help delineate safe and efficacious therapeutic windows.

Collectively, these strategies will enable the accurate characterization of the nonlinear effects of microbial metabolites. This, in turn, will provide a scientific basis for precision nutrition and epigenetic therapy.

#### Host genetic background interference

Host genetic polymorphisms play a vital role in determining how microbial metabolites impact host epigenetic regulation. While microbial metabolites may be similar across individuals, their biological effects can vary substantially depending on the host's genotype. For instance, variations in SCFA transporters such as SLC16A1 (MCT1) and SLC16A3 (MCT4) can affect the uptake of butyrate by tumor and immune cells, and thus the extent of HDAC inhibition [991, 992]. Variants in the CYP450 and SIRT gene families affect the capacity of the host to efficiently use NAD⁺ precursors (such as NR). Consequently, availability and activity levels of epigenetic enzymes controlling gene expression will also be affected [993]. In addition, polymorphisms that occur in innate immune receptors, such as TLRs and NOD2, mediate the detection of microbial signals and inflammatory cascades, with potential interactions with epigenetic modifications and immune regulation that are induced by metabolites [994].

Future studies should integrate metagenomic, genotypic, and epigenomic information from large cohorts to enable personalized therapy and model host genetic effects on microbe–epigenetic interactions. We can identify genetic loci that modify sensitivity to epigenetic regulation via gene‐environment interaction analyses. Measurable biomarkers can be identified for specific interventions. We will provide functional validation for therapies that can be either nutritional or epigenetic, and are based on the genotype, through either gene editing or humanized models. This will contribute to precision medicine.

### Technical breakthroughs

#### Real‐time in vivo monitoring

A major technical challenge is the ability to monitor, at the in vivo level, the dynamic and continuous interactions between microbial metabolites and host epigenetic marks. However, emerging technologies are starting to fix this problem. As an example, nanomaterial‐based biosensors enable highly sensitive detection of circulating tumor DNA and specific metabolites (e.g., α‐KG, 1‐MNA) [995, 996, 997]. Moreover, genetically encoded fluorescent probes, such as HyPer and RhoA‐FRET biosensors, coupled with multimodal live imaging strategies, enable the tracking at cellular resolution of oxidative stress and signaling within the TME [995, 996, 998].

Despite these improvements in sensitivity and resolution, challenges regarding signal stability, tissue penetration depth and long‐term biocompatibility. Future research should focus on designing highly specific molecular probes, integrating nanosensors with imaging systems, and employing AI algorithms for accurate data analysis. These advancements will facilitate the rapid investigation of the microbiome–host interactions in vivo.

#### Single‐cell metabolism–epigenetics integration

Tumor heterogeneity is a key contributor to cancer progression and treatment resistance. For understanding and explaining this complexity, metabolic and epigenetic profiling at a single‐cell level is crucial. Current strategies encompass microfluidics‐mass spectrometry coupling, single‐cell multi‐omics sequencing (e.g., scNMT‐seq), and spatial imaging (e.g., MALDI‐MSI). These methods can be used to link the levels of metabolites with chromatin states at either single‐cell or subcellular levels. They have been successfully used in many kinds of solid tumors. The combination of scRNA‐seq with spatial transcriptomics has revealed that nucleotide metabolism is reprogrammed in CRC tumor cores, which is linked to intercellular communication [999]. In BrCa, cells in clusters were found to switch from glycolysis to OXPHOS [1000]. Similar approaches uncovered that there exist metabolically heterogeneous subpopulations for the treatment response and prognosis in LUAD [1001]. As these technologies advance, they will enable a deeper functional stratification of the TME.

#### CRISPR‐Cas13‐based biosensing platforms for spatiotemporal analysis of metabolite–epigenetic crosstalk in the TME

Recent advances in understanding how microbial metabolites mediate epigenetic regulation of intestinal pathology have not fully clarified their causal mechanism, as spatiotemporal dynamics within heterogeneous TME remain a major theoretical and methodological challenge. The interactions between those metabolites and cells vary depending on the type of cell, metabolic state of the host and genetic background of the recipient cells. Moreover, finely tuned local microenvironmental parameters—such as oxygen tension, pH, and nutrient levels—also critically influence these cellular interactions.

Recent pan‐cancer studies have revealed that PTMs—including phosphorylation, acetylation, ubiquitination, and glycosylation—regulate immune checkpoint stability, antigen presentation, and immune cell activation through multilayered signaling networks, thereby directly influencing tumor immune evasion and therapeutic responses [1002]. Meanwhile, the human microbiome exerts critical influence on tumor initiation and progression; yet, the functional patterns of microbial metabolites vary markedly across cell types and metabolic contexts [1003], suggesting that their regulatory effects are highly dependent on host cell state and the surrounding microenvironment.

Research on the caspase family has put forth the concept of a “spatiotemporal‐activity continuum,” which implies that the functioning of a protease in dynamic microenvironments relies on its enzymatic activity gradient and spatial localization [1004]. The model provides a dynamic framework of metabolite–epigenetic regulation, indicating that epigenetic enzymes and their substrates work along a continuum rather than in a static manner. Nonetheless, current techniques offer limited temporal and spatial resolution, impeding real‐time characterization and systematic understanding of these rapid molecular events [730].

As such, CRISPR‐Cas13‐based biosensing represents a disruptive conceptual and technological advance to overcome existing limitations. Cas13 is a class II, RNA‐guided, RNA‐targeting effector protein. The catalytically inactive form (dCas13) of Cas13 is highly specific and therefore has applications in programmable RNA splicing, A‐to‐I and C‐to‐U editing, m^6^A mapping, and RNA–protein interaction profiling. Cas13 is also used for live‐cell imaging of RNA [1005].

Leveraging the precise RNA identification and cleavage capabilities of Cas13, highly quantitative, near‐real‐time monitoring of cellular metabolic signals can be achieved through highly quantitative approaches. By incorporating metabolite‐responsive probes, these platforms facilitate real‐time monitoring of microbial metabolites within the TME, as well as their effects on DNA methylation, histone modification, and ncRNA networks. Treatment with NaB causes apoptosis and modulates the HECTD2–EHMT2–TNFAIP1 signaling axis, which suggests that microbiota SCFAs regulate tumor epigenetic networks using metabolism–epigenetic crosstalk mechanisms [1003].

This strategy enables high‐spatial‐resolution, and dynamic visualization of metabolism–epigenetic interaction processes, providing a powerful tool for deciphering how microbial metabolites shape the epigenetic landscape of tumors. Looking ahead, the integration of CRISPR‐Cas13 based RNA targeting approaches with single‐cell transcriptomics, spatial omics, and multi‐omics analyses holds strong potential to enable system‐level and spatiotemporal dissection of microbial metabolite‐driven epigenetic regulation within the TME [1006, 1007, 1008]. Such convergence will deepen and expand our understanding of tumorigenesis and progression to an unprecedented degree.

#### Cross‐scale data integration

Research on microbiome–host interactions spans multiple scales, from molecular to organismal levels, and encompasses various modalities, including genomes and clinical phenotypes. This complexity poses a significant computational bottleneck for integrative analysis. To address this challenge, establishing unified data standards and semantic ontologies is essential, as is developing advanced multi‐omics integration algorithms such as MOFA and graph neural networks to extract cross‐scale associations [1009]. Notably, circulating free RNA (cfRNA) may provide more comprehensive information than cfDNA in cancer detection, serving as a more sensitive tool for integrative analyses [1010]. Future studies will depend on scalable cloud computing, automated analytical pipelines, and interactive visualization tools. These components will be integrated with iterative feedback from functional experiments to construct and validate robust regulatory network models of “microbiome–metabolite–epigenetic” interactions, ultimately guiding precision interventions.

#### Limitations and complementarity of multi‐omics technologies

Despite the promise of multi‐omics approaches for elucidating microbiome–metabolite–epigenetic interactions, major methodological hurdles remain. Dissimilarities in platform sensitivity, quantitative accuracy, and spatial resolution reduces data comparability. Variability in chemical compound measurements, often stemming from technical inconsistencies or batch effects, can distort correlations and mislead mechanistic reasoning. Furthermore, protein abundance often better reflects subtype activity than copy number variations or mutations, underscoring the predominant influence of non‐genetic factors on oncogenic protein regulation [1009]. Even when global methylation levels correlate strongly with reference standards (PCC > 0.95), significant methodological discrepancies may persist, and sequencing depth variability can introduce site‐specific bias, compromising interpretation of key genomic regions [1011]. Single‐cell data frequently exhibit high dropout rates, accumulated noise, and computational bottlenecks due to massive dataset sizes—often encompassing millions of cells—collectively complicating data integration [968].

Each omics layer provides complementary information: the metabolome delineates the functional end state, the epigenome reflects the regulatory plasticity, and the transcriptome serves as a dynamic bridge between them. Single‐cell and spatial transcriptomics have been applied across multiple cancer types to resolve tumor ecosystem architecture and immune dynamics [999, 1012]. To reduce uncertainty, multi‐level cross‐validation should be implemented at both experimental and computational levels. This may include repeated sampling within the same cohort, heterogeneity correction models, cross‐platform comparisons, and integration of time‐series data to minimize biases introduced by asynchrony. Multimodal fusion algorithms—such as MOFA, DIABLO, and Bayesian Network models—can, to some extent, correct for these biases. Among them, MOFA+ utilizes variational inference and sparse regularization to jointly model multi‐modal variance, thereby enhancing consistency across samples and modalities [968].

Future multi‐omics studies must prioritize standardized data formats and quality control. Normalization, batch correction, and bias detection should be implemented at the raw‐signal level with clear model assumptions and uncertainty definitions. Key findings require validation across different platforms, cohorts, and timepoints. Standardized quantification of metabolites such as SCFAs will ensure inter‐study comparability. Integrating exposomic data with clinical endpoints can strengthen causal inference across metabolic, epigenetic, and functional layers. To ensure the translational success of research employing multi‐omics integration of microbiome–metabolite–epigenetic networks, methodological constraints must be fully addressed.

### Future directions

#### Synthetic biology‐driven metabolite engineering

Synthetic biology offers revolutionary tools for precisely modulating epigenetics in cancer therapy. By engineering programmable microbes or cell therapy vectors, researchers can achieve on‐demand, localized synthesis and release of epigenetic regulators, such as SCFAs or indole derivatives. For instance, engineered probiotics that produce elevated levels of l‐arginine within the TME have been shown to significantly enhance the efficacy of anti–PD‐L1 therapy [1013]. Similarly, synthetic gene circuits can be designed to convert inert prodrugs into active anticancer agents specifically within tumors [1014]. This approach can also be used to optimize CAR‐T therapies by targeting their metabolic pathways and epigenetic states, thereby enhancing antitumor activity and persistence [1015]. With advances in gene editing, metabolic modeling, and precision delivery, synthetic biology‐driven metabolite engineering is poised to emerge as an innovative strategy for reprogramming tumor epigenetics and overcoming drug resistance.

#### Application of epigenetic editing technologies

Technologies for epigenetic editing based on dCas9 enable accurate “writing” or “erasing” of epigenetic marks by fusing dCas9 to histone‐modifying enzymes (e.g., Ezh2 or p300), which are then guided by sgRNAs to specific genomic loci. This approach allows for the targeted activation or silencing of genes, demonstrating considerable efficacy across various tumor types. For instance, polysaccharide A has been used to target oncogenes and inhibit PCa cell proliferation [1016]. In BrCa therapy, it has been applied to reactivate TSGs. Coupling these editing systems with advanced delivery platforms, such as mesoporous silica nanoparticles [1017] and synthetic biology gene circuits [1018], enables precise spatiotemporal control over editing activity with minimal off‐target effects. These advances indicate that epigenetic editing could emerge as a potent and highly controllable strategy for tumor gene therapy [1019, 1020].

#### AI‐assisted personalized therapy

AI is transforming personalized cancer treatment by integrating large multi‐omics datasets with clinical information. Deep learning models can automatically identify biomarker combinations associated with therapeutic response and drug resistance from complex, heterogeneous data, enabling accurate predictions of patient survival subtypes [1021] and treatment outcomes [1022]. For example, ML models based on clinical and laboratory data have shown performance superior to the standard of care for early LuCa diagnosis [1023]. Likewise, AI frameworks incorporating radiomics have enhanced the molecular subtyping of BrCa [1024]. Furthermore, AI‐based screening systems have proven highly effective and efficient for cervical cancer diagnosis [1025]. Moving forward, the development of explainable AI and privacy‐preserving ML techniques, such as federated learning, will better facilitate individualized cancer treatment strategies based on microbiome–metabolism–epigenetics networks while ensuring data security and decision‐making transparency.

## DISCUSSION

The notion that growing tumors serve as symbiotic microecosystems, rather than mere aggregates of malignant cells, could reshape our understanding of cancer. Microbial metabolites are not only external perturbations but also integral components of the tumor's endogenous epigenetic regulatory network. While tumor phenotypes and biological behaviors are shaped by heritable genetic modifications, they also exhibit significant plasticity in response to environmental and dietary signals.

In the TME, metabolites from microbes and the host epigenetic machinery continuously form complex positive feedback loops that not only drive malignant progression but also provide insights into therapeutic resistance mechanisms. Cancer cells undergo metabolic reprogramming via the Warburg effect, creating a hypoxic, acidic, and nutrient‐deprived niche. These conditions favor the proliferation of acid‐tolerant, anaerobic microbes. Microbial products, along with tumor‐derived lactate and succinate induce histone lactylation and inhibit HDAC activity, hence strengthening expression of glycolytic and proliferative genes and worsening epigenetic dysregulation [802]. Emerging evidence indicates that therapeutic resistance arises not only from innate mutational changes in cancer cells but also from microbial‐induced epigenetic self‐regulation. Therefore, future strategies should regard tumor cells and their microbes as a unit and employ multi‐level targeting of metabolism and epigenetics to disrupt this alliance.

Tumors are not merely masses of cells but complex ecosystems comprising diverse life forms. Microbial metabolites play a role in initiation and progression of tumors and modulation of host epigenetic states. These effects can be protective, as seen with SCFAs that inhibit HDAC activity to maintain metabolic and immune homeostasis, exerting anti‐inflammatory and antitumor effects. Conversely, they can be oncogenic by inducing DNA methylation and histone modifications that silence TSGs or activate oncogenes, thereby promoting malignant transformation [562]. In GCa, for instance, a gastric microbiota resistant to *Helicobacter pylori* eradication may persist. Even in patients without *H. pylori* infection, carcinogenesis risk remains due to infection‐ or injury‐induced epigenetic modifications [1026]. In addition, the remodeling of microbial community structure plays a key role in tumor immunotherapy, as microbial metabolites can modulate immune cell polarization and immune checkpoint molecule expression. These factors can affect the evasion of immunity and the efficacy of the therapy.

Despite increasing recognition of the role of microbial metabolites in tumor epigenetic regulation, several key challenges remain. One major challenge is unresolved causality, as much is still unknown about how these factors are related to one another. Most studies on tumors are cross‐sectional and therefore fail to provide insight into metabolite–epigenetic temporal changes. Additionally, while noncanonical histone acylations, such as propionylation and butyrylation, can affect chromatin accessibility, further work is needed to confirm their in vivo mechanisms and context‐specific effects in different tumor types and microbes [1027].

Another challenge lies in host variability, because metabolic responses vary greatly between individuals. Changes in the activity of a metabolic enzyme or the expression of a transporter can alter the uptake of a metabolite and the downstream signals that are transmitted. As a result, the observed epigenetic outcomes resulting from any given metabolite may diverge significantly, and these divergences can compound uncertainties in predicting personalized therapeutic responses [1028].

A further challenge involves technological and model limitations. Although many studies have demonstrated the importance of microbial metabolites in tumor epigenetic regulation, technical and modeling bottlenecks still limit clinical translation. Systems for precise microbiome detection and dynamic monitoring, as well as clinically integrated evaluation linking microbiota with epigenetic changes, remain insufficient. Existing experimental models also face limitations, including complexity, scalability, and cost.

Current research is also faced with methodological inconsistencies and biological contradictions. Targeted interventions focused on the microbiome can have very different outcomes even within the same type of cancer. The outcomes are variable likely due to interindividual differences in microbiota composition, dietary habits, and metabolic state, among other factors [980]. Current experimental models are still limited in depicting interactions of tumor, microbes, metabolism, immunity and vasculature. The taxonomic and metabolic profiles of murine microbiota differ significantly from those of humans, and 2D cultures cannot reproduce the spatial competition and vascular proximity of the TME. Even organoid systems do not have integrated assessment of immune–microbial–metabolic coupling. Single‐cell spatial metabolomics techniques provide insights into protein–metabolite interactions in human tissues, capturing alterations in lineage and competitive cell type states during differentiation processes [1029]. Future studies should seek to develop multidimensional frameworks to elucidate the causal and spatiotemporal dynamics of microbiome–metabolite–epigenetic–tumor interactions systematically, which encompasses spatial metabolomics and single‐cell epigenomics, transcriptomics, and immunomics.

As this field advances, future studies will need to answer fundamental scientific questions, including (1) how to delineate causative links between specific microbial metabolites and key epigenetic enzymes (e.g., DNMTs, TETs, and HDACs) across tumor types; (2) how to derive patient‐specific metabolic–epigenetic signatures that predict the efficacy and safety of microbe‐based and epigenetic therapies; and (3) how to establish safety limits for the long‐term modulation of microbial metabolism or epigenetic states to avoid off‐target effects in normal tissues. To address these questions, humanized organoid systems, spatiotemporal multi‐omics studies, and AI‐based causal inference systems can help transform descriptive findings into falsifiable, clinically actionable strategies.

The complex relationship between microbes and cancer can help doctors make better treatment choices. Subsequent research should critically evaluate current inconsistencies regarding the issues of causality, predictability and safety while integrating advanced experimental models and technological platforms. This will speed up the accurate clinical translation of the microbiome‐metabolite‐epigenetic axis towards next‐generation personalized cancer therapies.

## AUTHOR CONTRIBUTIONS

Wangzheqi Zhang, Haoling Zhang, Yan Liao, Shuya Jiang, Haolong Zhang, Chenwei Huang, Zhijing Song, Rui Zhao, Ping Lu, Xiaohong Kang, Eryan Kong, Yalin Zhu, Wei Wang, Xuesong Liu, Yisheng Chen, Zhiwen Luo, Zhaoyu Li, Di Wang, Na Xing, Yadong Guo, Jingjing Zhang, Xiaojing Wang, Wenyi Wang, Mowaffaq Adam, Bakiah Shaharuddin and Muhamad Yusri Musa contributed to the manuscript writing and figure preparation. Doblin Sandai, Chenglong Zhu, Aimin Jiang, Peng Luo and Haoling Zhang supervised the work. Wangzheqi Zhang, Zhijie Zhao and Zui Zou reviewed the manuscript and proofed the figures. All authors have read the final manuscript and approved it for publication.

## CONFLICT OF INTEREST STATEMENT

The authors declare no conflicts of interest.

## ETHICS STATEMENT

No animals or humans were involved in this study.

## Data Availability

Data sharing not applicable to this article as no datasets were generated or analyzed during the current study. Supplementary materials (graphical abstract, slides, videos, Chinese translated version and update materials) may be found in the online DOI or iMeta Science http://www.imeta.science/.
